# Genome-Wide fitness analysis of group B *Streptococcus* in human amniotic fluid reveals a transcription factor that controls multiple virulence traits

**DOI:** 10.1371/journal.ppat.1009116

**Published:** 2021-03-08

**Authors:** Allison N. Dammann, Anna B. Chamby, Andrew J. Catomeris, Kyle M. Davidson, Hervé Tettelin, Jan-Peter van Pijkeren, Kathyayini P. Gopalakrishna, Mary F. Keith, Jordan L. Elder, Adam J. Ratner, Thomas A. Hooven

**Affiliations:** 1 Department of Pediatrics, New York University School of Medicine, New York, New York, United States of America; 2 University of Vermont Larner College of Medicine, Burlington, Vermont, United States of America; 3 Georgetown University School of Medicine, Washington, District of Columbia, United States of America; 4 Department of Pediatrics, University of Pittsburgh School of Medicine, Pittsburgh, Pennsylvania, United States of America; 5 Department of Microbiology and Immunology, University of Maryland School of Medicine, Baltimore, Maryland, United States of America; 6 Institute for Genome Sciences, University of Maryland School of Medicine, Baltimore, Maryland, United States of America; 7 Department of Food Science, University of Wisconsin, Madison, Wisconsin, United States of America; 8 Department of Microbiology, New York University, New York, New York, United States of America; 9 Richard King Mellon Institute for Pediatric Research, UPMC Children’s Hospital of Pittsburgh, Pittsburgh, Pennsylvania, United States of America; University of Texas Medical School at Houston, UNITED STATES

## Abstract

*Streptococcus agalactiae* (group B *Streptococcus*; GBS) remains a dominant cause of serious neonatal infections. One aspect of GBS that renders it particularly virulent during the perinatal period is its ability to invade the chorioamniotic membranes and persist in amniotic fluid, which is nutritionally deplete and rich in fetal immunologic factors such as antimicrobial peptides. We used next-generation sequencing of transposon-genome junctions (Tn-seq) to identify five GBS genes that promote survival in the presence of human amniotic fluid. We confirmed our Tn-seq findings using a novel CRISPR inhibition (CRISPRi) gene expression knockdown system. This analysis showed that one gene, which encodes a GntR-class transcription factor that we named MrvR, conferred a significant fitness benefit to GBS in amniotic fluid. We generated an isogenic targeted deletion of the *mrvR* gene, which had a growth defect in amniotic fluid relative to the wild type parent strain. The *mrvR* deletion strain also showed a significant biofilm defect *in vitro*. Subsequent *in vivo* studies showed that while the mutant was able to cause persistent murine vaginal colonization, pregnant mice colonized with the *mrvR* deletion strain did not develop preterm labor despite consistent GBS invasion of the uterus and the fetoplacental units. In contrast, pregnant mice colonized with wild type GBS consistently deliver prematurely. In a sepsis model the *mrvR* deletion strain showed significantly decreased lethality. In order to better understand the mechanism by which this newly identified transcription factor controls GBS virulence, we performed RNA-seq on wild type and *mrvR* deletion GBS strains, which revealed that the transcription factor affects expression of a wide range of genes across the GBS chromosome. Nucleotide biosynthesis and salvage pathways were highly represented among the set of differentially expressed genes, suggesting that MrvR may be involved in regulating nucleotide availability.

## Author summary

Group B *Streptococcus* (GBS) is a species of Gram-positive bacteria that often colonizes the healthy adult intestinal and reproductive tracts without causing serious symptoms. During pregnancy, however, GBS can invade the pregnant uterus, where it can cause infection of the placenta, fetal membranes, and fetus—a condition known as chorioamnionitis. Chorioamnionitis is associated with serious adverse pregnancy outcomes, including stillbirth, preterm labor, and severe infection of the newborn. GBS can survive in human amniotic fluid, which is low in bacterial nutrients and contains immune molecules that limit microbial persistence, and this ability likely contributes to GBS chorioamnionitis. This study is focused on a single GBS gene that encodes a genetic regulator we called MrvR, which we show is important for GBS resistance to human amniotic fluid. Using a series of genetic techniques combined with animal models of GBS colonization and infection, we show that MrvR also plays a key role in allowing GBS to invade the bloodstream and trigger the inflammatory responses that lead to preterm labor and stillbirth. The study concludes with a survey of other GBS genes whose activity is regulated by MrvR, which seems to be an important contributor to GBS virulence.

## Introduction

*Streptococcus agalactiae* (group B *Streptococcus*; GBS) is a cause of chorioamnionitis, stillbirth, and neonatal infections including bacteremia, pneumonia, and meningitis [1–10]. GBS is a common commensal of the intestinal and reproductive tracts in healthy adults, among whom invasive disease is rare [11,12]. In the pregnant or neonatal host, however, GBS can be highly invasive, breaching anatomic and immunologic barriers with potentially severe consequences [13,14].

GBS is known to express a number of virulence factors such as adhesins [15–17], IgA binding proteins [18–20], and the cytotoxic ornithine-rhamnopolyene β-hemolysin/cytolysin [13,21–30]. Many of these are regulated by transcription factors, some of which have been studied and described in detail [31–40], yet many of the predicted transcription factors encoded by GBS remain minimally characterized.

We have previously described development of a GBS saturated transposon mutant library and its use in Tn-seq experiments to identify essential and conditionally essential genes [41,42]. Tn-seq permits genome-wide quantification of bacterial gene fitness through highly parallel sequencing of a saturated transposon library in which transposon insertion within a given gene is expected to result in loss of that gene product [43–46]. In Tn-seq, the saturated mutant library is grown under two conditions: control outgrowth in which there is minimal selection pressure on the bacterial population (for instance, in rich media) and an experimental outgrowth condition that introduces a selective bottleneck [46]. Following DNA purification from the two conditions, next-generation sequencing is performed in a manner that allows quantification of genome-wide transposon insertion sites. By identifying transposon insertion sites that are relatively absent from the experimental outgrowth condition, it is possible to infer which genes (or potentially intergenic regions) are essential for bacterial survival under those outgrowth circumstances [43,46–53]. Tn-seq has been widely used across a variety of bacterial species to identify genes and pathways that enable microbial survival under environmental stress [47,48,54–62]. In this study, we performed Tn-seq on GBS grown in human amniotic fluid in order to identify bacterial genes that promote survival in the nutrient-poor, unhospitable growth conditions of the amniotic sac.

The top candidate genes from our Tn-seq screen were further studied using a newly developed CRISPR interference (CRISPRi) system. Whereas wild type components of the CRISPR-Cas family of bacterial immune molecules can be used for RNA-guided, programmable double stranded DNA cleavage [63,64], two targeted mutations of Cas9 endonuclease active sites result in a catalytically inactive isoform (dCas9). Rather than introducing double stranded breaks in DNA at the site of guide RNA recognition, dCas9 can be programmed to occupy a particular region of DNA, creating steric hindrance that disrupts transcriptional initiation or elongation [65,66]. This approach allows rapid, flexible generation of functional gene expression knockdowns that permit preliminary study of a gene’s phenotypic contribution without requiring generation of a targeted chromosomal deletion.

Our CRISPRi system builds on similar approaches that have been developed for other bacteria [65–69], using a plasmid-based single guide RNA (sgRNA) expression system delivered to a GBS strain in which the native *cas9* gene has been altered to render it catalytically inactive. As with CRISPRi approaches based on the canonical Cas9 enzyme from *Streptococcus pyogenes*, CRISPRi in GBS requires existence of a 20-bp DNA region complementary to the sgRNA sequence and followed by a protospacer adjacent motif (PAM) with a NGG sequence, which is the same in GBS as in *S*. *pyogenes* [70].

For each of the five genes found by Tn-seq to be conditionally essential for GBS survival in amniotic fluid, we identified two sgRNA sequences that met the requirements for dCas9 targeting. We then used these sequences, cloned into a sgRNA expression plasmid, to generate a pair of knockdown strains for each candidate gene. We screened these CRISPRi knockdown strains for survival defects in amniotic fluid, which allowed one form of validation of our Tn-seq data. There is evidence that the Cas9 enzyme has regulatory properties beyond its role as a bacterial immune defender against bacteriophages, and that—in GBS—some Cas9-regulated genes affect virulence [71,72]. For this reason, results of CRISPRi knockdown experiments must be viewed as provisional evidence for a particular gene’s role in the bacterial phenotype, requiring further validation with a targeted gene deletion approach.

Our CRISPRi assessment of candidate genes suggested a significant survival advantage in amniotic fluid mediated by a transcription factor with domain features marking it as a member of the GntR protein superfamily. GntR regulators have a conserved helix-turn-helix DNA binding motif at the N-terminus but vary in the structure of the C-terminal effector-binding domain [73]. Based on shared similarities within the effector-binding domain, the GntR superfamily is further subdivided into four main subfamilies (FadR, HutC, MocR, and YtrA). Two minor subfamilies, AraR and PlmA, have also been described, the latter expressed only in cyanobacteria [73–77]. Conformational changes induced by ligand binding to the C-terminus of a GntR regulator alters the configuration of the N-terminus, modulating the protein’s DNA binding affinity [75,77,78]. As a result of the diversity of C-terminus structures within the GntR superfamily, these transcription factors respond to a wide variety of effector ligands. Most experimentally proven GntR effectors are small molecules that are generated or consumed by central metabolic pathways [73,75,77].

We named the GBS GntR-class transcription factor with a suspected fitness role in amniotic fluid MrvR (multiple regulator of virulence). This report offers results of our whole genome Tn-seq screen, CRISPRi knockdown studies, and experiments performed with an allelic exchange gene deletion mutant strain (and an appropriate complement control strain) to provide an initial description of the role of MrvR in several GBS virulence-related phenotypes, including survival in human amniotic fluid, *in vitro* biofilm formation, and invasive bacteremia and preterm labor in murine models. We also report transcriptomic data from RNA-seq performed with wild type GBS and a mutant strain lacking the *mrvR* gene. The transcriptomic results suggest a role in modifying expression of genes integral to nucleotide metabolism.

## Results

### Five GBS genes are conditionally essential for GBS survival in human amniotic fluid

Using a previously described *Himar1* transposon mutant library in an A909 (serotype Ia) background [41], we performed Tn-seq after growth challenge in eight human amniotic fluid samples from amniocentesis.

The A909 library was originally created using a temperature sensitive shuttle plasmid-based system for delivery of the *Himar1* mini-transposon and a transposase-encoding gene to electrocompetent GBS. Prior to its use in GBS, the terminal ends of the mini-transposon were modified so that they contained MmeI restriction sites. MmeI is an unusual type II restriction enzyme that cuts 20 bp downstream from its recognition site [79]. The separation between the recognition and cut sites has been exploited in Tn-seq studies, since it permits retention of transposon-adjacent host genomic DNA sequence information following MmeI digestion of transposon library DNA [45,58,80,81]. This segment of host DNA provides enough sequence specificity to pinpoint the site of transposon insertion in the source cell, which is fundamental to the Tn-seq approach.

Initial development, testing, and preliminary Tn-seq studies using our A909 library were described in detail in a prior publication [41]. Our approach to creating and characterizing the library was guided by similar work performed in *S*. *pyogenes* and *Streptococcus pneumoniae* backgrounds by Le Breton et al., van Opijnen, and Camilli [44,80,81]. When creating the A909 Tn-seq system, we originally produced three separate libraries, each with 36,000–100,000 unique transposon insertions. As in our prior work using Tn-seq to identify conditionally essential GBS genes in human blood [42], for this study we used a pooled mixture of the three separate libraries shown previously to have 167,000 unique mutations, which corresponds to 45% saturation of available TA-dinucleotides sites available to the *Himar1* mini-transposon in A909 [41].

Before inoculation with the GBS transposon library, the amniotic fluid used for outgrowth was filter sterilized to remove any contaminating microorganisms and human cells that may have been present. In pilot experiments with wild type A909 maintained in human amniotic fluid, we observed that an initial inoculation density of 10^8^ CFU/mL decreased to 10^7^ CFU/mL after overnight incubation. This decrease in CFU density suggested to us that overnight incubation in amniotic fluid exerted selective pressure against GBS without creating a severe bottleneck that would prohibit accurate calculation of gene contributions to fitness. We therefore used an overnight incubation strategy in our Tn-seq library selection experiment.

The eight amniotic fluid samples used for outgrowth were comprised of two or three equal-volume aliquots from three separate women. The purpose of this design was to ensure that neither donor-specific (such as antibiotic exposure or chorioamnionitis at the time of amniocentesis) nor sample-specific (microbial contamination, for instance) factors influenced the Tn-seq outcome. Each amniotic fluid aliquot was seeded independently with the A909 transposon library. The same pooled mutant library was grown in rich media as an input control. Following 16-hour experimental or control incubation and a subsequent outgrowth phase in rich media to increase biomass, DNA was purified from each sample and processed for Tn-seq by MmeI digestion and subsequent ligation of separate, barcoded double-stranded DNA adapters to the resultant DNA sticky ends. The final step was PCR amplification of transposon-genome junctions, followed by next-generation sequencing of the resulting amplicon library.

**Fig 1:**
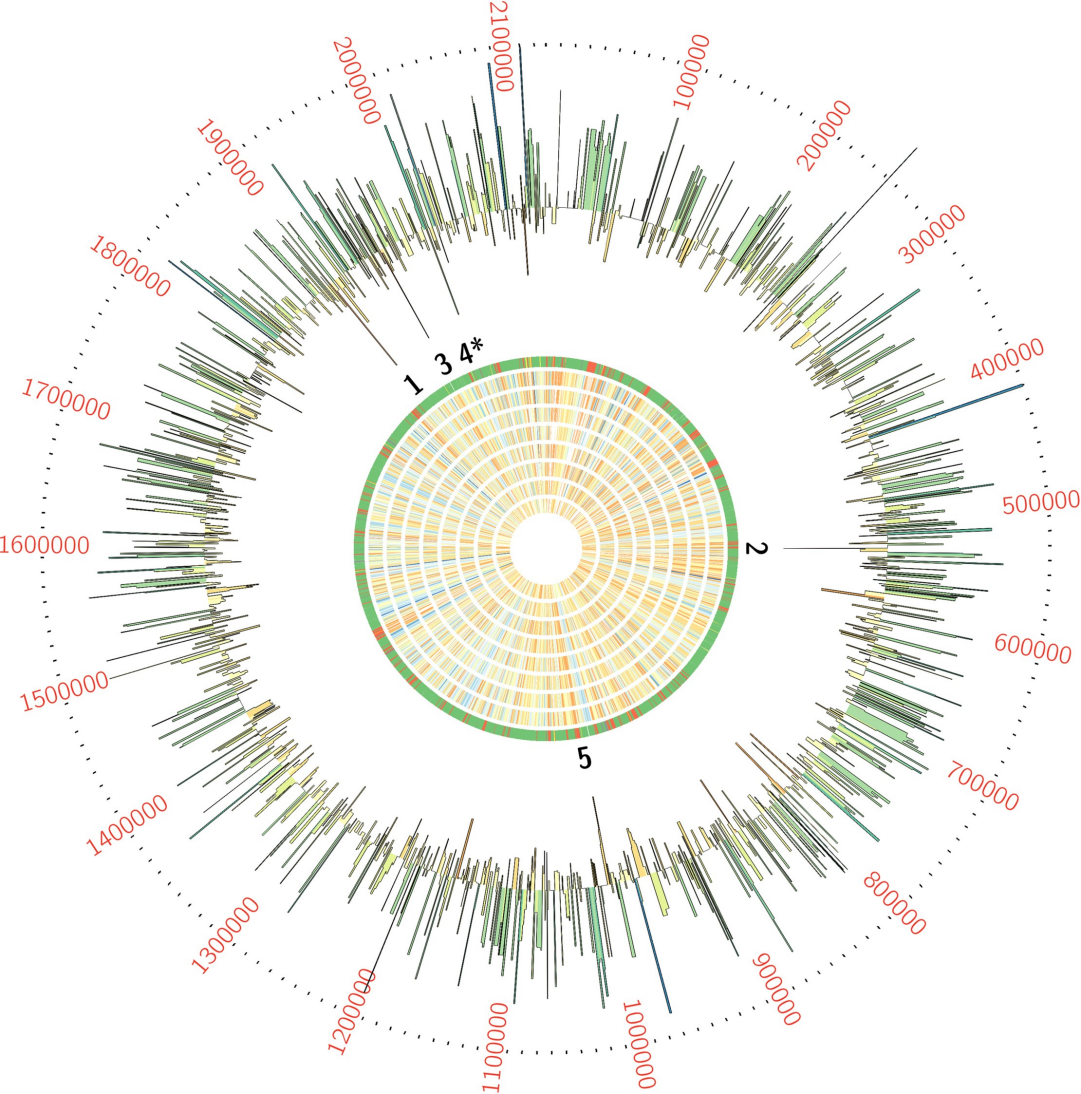
Tn-seq screening of GBS grown in human amniotic fluid revealed 5 conditionally essential genes. GBS transposon library in a background of strain A909 was grown in eight human amniotic fluid samples from three individuals (see main text), then subjected to Tn-seq analysis. Each ring of the Circos plot shows a different representation of the A909 circular chromosome. From the center of the plot, rings 1–8 show transposon insertion density detected in the eight amniotic fluid outgrowth samples (lowest detection: red; highest detection: blue). Ring 9 shows essential genes (red), nonessential genes (green), intermediate genes (yellow), and those for which baseline fitness cannot be determined (white; see reference [41]). The outermost ring shows log_2_ fold-change values for each gene in amniotic fluid outgrowth as determined by ESSENTIALS. Genes with inward-pointing peaks had fewer than expected transposon insertions. Outward-pointing peaks had more than expected transposon insertions. The peaks are color coded for statistical significance (low adjusted p value: red; high adjusted p value: green). The five labeled peaks are described in the text and are listed in **Table 1**. Peak 4 is the GntR-class transcription factor, *mrvR*.

We used ESSENTIALS, a publicly available Tn-seq bioinformatics package, to analyze sequencing data from the Tn-seq experiment [50]. ESSENTIALS takes sequencing reads or alignment files as input; in this instance, we performed initial alignment of our Tn-seq data to the A909 genome using Bowtie 2 [82]. Following data upload, ESSENTIALS normalizes read counts across control and experimental replicates using the trimmed mean of M values (TMM), which corrects for differential sequencing depth and potential compositional biases resulting from experimental depletion of the mutant library [83]. Following normalization, statistical differences in gene transposon insertion are determined using an exact test performed within the negative binomial distribution statistical model in EdgeR [84]. Following this statistical testing, each nonessential gene’s contribution to fitness can be assessed by the log_2_ transformed ratio of transposon insertions detected in the experimental condition relative to control. This ranked list, with associated p values from the exact test, is a useful way to compare relative fitness contributions (**S1 Data**). However, ESSENTIALS also generates a kernel density plot of experimental-versus-control insertions and calculates local minima on that plot (**S1 Data, bottom**), which can provide a more stringent separation of conditionally essential genes from those with less of a contribution to fitness [50]. We used this local minimum calculation to identify five candidate conditionally essential genes expected to have the most significant contribution to GBS survival in amniotic fluid (**Fig 1 and Table 1**).

**Table 1:** GBS genes conditionally essential for survival in human amniotic fluid.

Gene locus	log_2_ Fold-Change^a^	Expected product
SAK_RS09545	-4.3690439	DeoR family transcriptional regulator
SAK_RS02930	-4.3341605	hypothetical protein
SAK_RS09890	-4.1865043	ABC-class transporter
SAK_RS10120	-3.8123495	GntR family transcriptional regulator (mrvR)
SAK_RS05160	-3.7270772	membrane-spanning protein of unknown function

a Fold-change between expected and observed transposon insertions in amniotic fluid experimental outgrowth conditions

One of the candidate genes, SAK_RS10120, which we termed *mrvR*, had conserved functional domains suggesting its membership in the GntR superfamily of transcriptional regulators. The other candidate genes encoded an expected DeoR family transcriptional regulator (SAK_RS09545), a predicted membrane-spanning protein of unknown function (SAK_RS05160), an ABC-class transporter (SAK_RS09890), and one protein (SAK_RS02930) with a single domain of unknown function whose cellular localization and role are also unknown.

### CRISPRi knockdown and targeted knockout competition assays validate Tn-seq findings

To further validate the five candidate genes conditionally essential for GBS survival in amniotic fluid, we developed a CRISPRi gene expression knockdown system, which uses natively expressed, catalytically inactive Cas9 (dCas9) encoded on the GBS chromosome along with a single guide RNA (sgRNA) expression plasmid to generate efficient and flexible gene expression inhibition in GBS. We competed knockdowns of the five candidate conditionally essential genes against a sham targeting control dCas9 strain in human amniotic fluid and assayed survival using quantitative PCR analysis of knockdown and control strain persistence.

Development of a GBS CRISPRi system was aided by the fact that the GBS *cas9* gene shows high sequence similarity to the canonical *cas9* endonuclease first described in *S*. *pyogenes* [64,85–87] (**S1 Fig**). This homology allowed us to predict the two missense mutations that would establish a catalytically inactive isoform in GBS: D10A and H840A. Lopez-Sanchez et al. have previously shown that the protospacer adjacent motif (PAM) necessary for GBS Cas9 genomic target recognition follows the same NGG pattern as in *S*. *pyogenes* [70]. We used a previously described sucrose-counterselectable mutagenesis plasmid, pMBsacB, to generate point mutations within the coding sequence of the *cas9* gene in GBS strain CNCTC 10/84 (serotype V) [88], which is the strain with which our animal models of vaginal colonization and ascending chorioamnionitis have been optimized.

We developed a sgRNA expression shuttle plasmid, p3015b, which accepts ligation of a 20-bp protospacer against a target gene between two BsaI restriction enzyme recognition sites. We used the Broad Institute’s Genetic Perturbation Platform sgRNA design tool (https://portals.broadinstitute.org/gpp/public/analysis-tools/sgrna-design) to select optimized protospacers [89,90]. After confirming successful protospacer cloning in an *E*. *coli* background, the targeting plasmids were purified and transformed into competent 10/84 modified to express dCas9.

**Fig 2:**
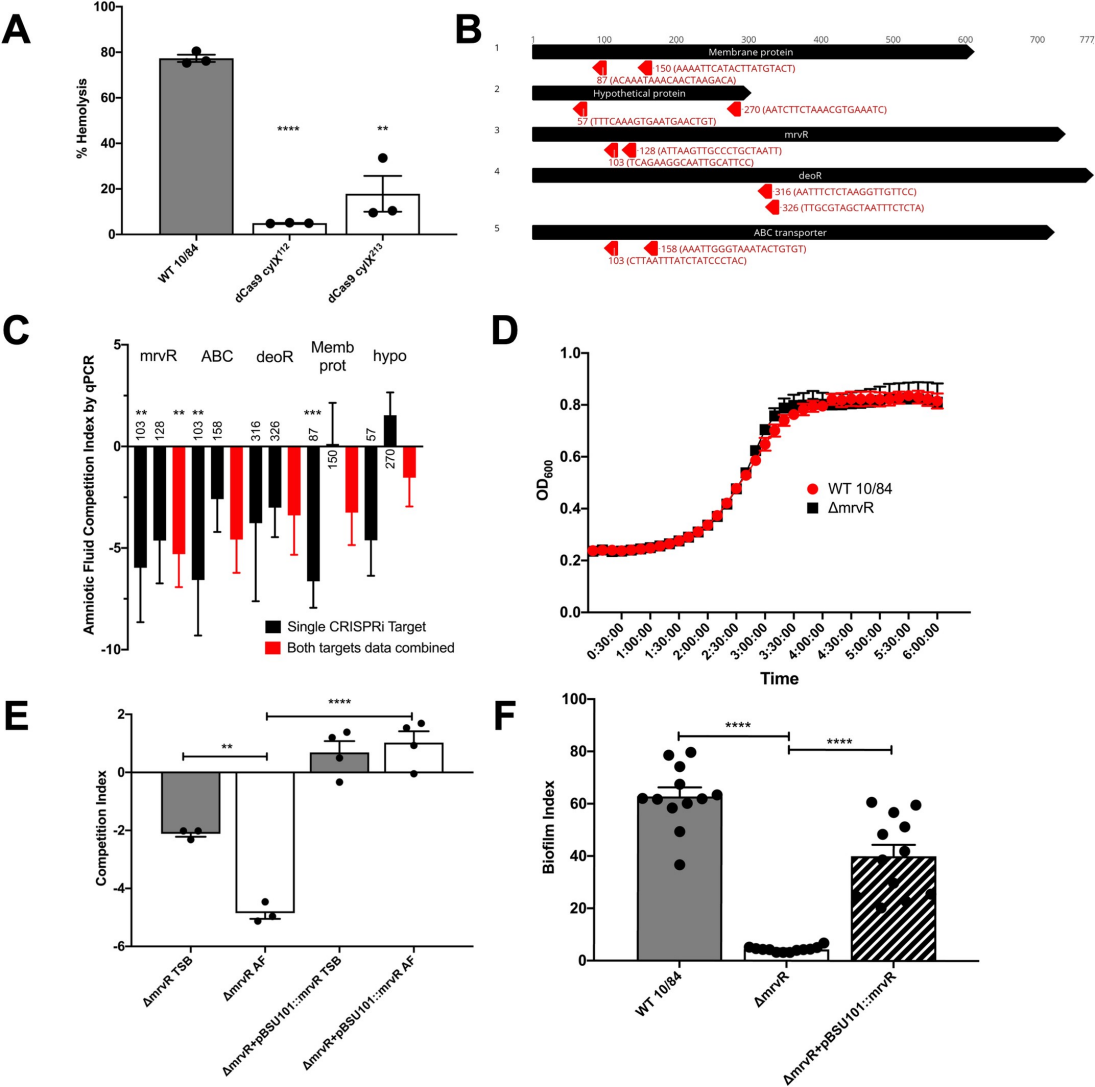
CRISPRi knockdowns of candidate genes and phenotypic characterization of GBS lacking MrvR in amniotic fluid, rich media, and biofilm-promoting growth conditions. Two CRISPRi targeting protospacers (targeting nucleotides 112 and 213 of *cylx*, the first gene in the operon) against the *cyl* operon were developed to knockdown expression of β-hemolysin/cytolysin. The resultant knockdown strains (in a 10/84 background) were compared to wild type in a human erythrocyte hemolysis assay (**A**). Targeting protospacers against the five candidate conditionally essential genes from amniotic fluid Tn-seq. For each gene, the number indicates the targeted nucleotide; the protospacer sequence is shown in parentheses (**B**). A qPCR-based multiplex CRISPRi screen using knockdowns of all five candidate genes revealed decreased fitness in amniotic fluid compared to tryptic soy (TS) broth (**C**); black columns show the competition index of strains bearing individual targeting protospacer plasmids, while red columns show pooled results from both targeting plasmids for each gene. Statistical significance of each knockdown phenotype was determined relative to growth of the knockdowns in TS broth. Growth curves of the Δ*mrvR* and wild type (WT) 10/84 in broth (**D**). Colony forming unit enumeration-based competition assays between Δ*mrvR* and Δ*mrvR*+pBSU101::*mrvR* in TS broth (TSB) and amniotic fluid (AF); **E**). Biofilm assay results comparing WT 10/84, Δ*mrvR*, and Δ*mrvR*+pBSU101::*mrvR*. (*p<0.05, ** p<0.01, *** p<0.005, **** p<0.001; t test, error bars show standard error surrounding mean).

To establish proof-of-concept for this approach we knocked down the first open reading frame (*cylX*) in the *cyl* operon that encodes genes responsible for synthesis of the pigmented cytotoxin β-hemolysin/cytolysin [91]. p3015b encoding anti-*cylX* sgRNA could not be transformed into wild type GBS, presumably because Cas9 targeting to the chromosome resulted in lethal double-stranded DNA cleavage. The dCas9 strain, however, had high transformation efficiency and generated non-pigmented, non-hemolytic knockdowns when transformed with plasmid expressing anti-*cylX* sgRNA (**Fig 2A**).

Next, we created plasmids targeting two different sites in each of the five candidate amniotic fluid conditionally essential genes **(Fig 2B**) and transformed dCas9-expressing 10/84. The Broad Institute’s Genetic Perturbation Platform allows prioritization of sgRNA-complementary target sites with certain characteristics [89,90]. Where possible, we selected targets in the first half of the candidate gene coding sequence, without polynucleotide repeats greater than four bases, and without overlap of the two target sequences. These criteria were met for three of the five candidate genes, but were not universally achievable: overlap of the two target sequences was required for the *deoR* gene and one of the two hypothetical protein targets was in the second half of the coding sequence.

The resulting knockdown strains, plus a control dCas9 strain transformed with p3015b bearing a sham protospacer, were tested through qRT-PCR for target gene silencing. After growth to stationary phase, reverse transcribed cDNA from each knockdown and the non-targeted control was normalized to *recA*, a stably expressed GBS housekeeping gene [92]. For each knockdown strain, the targeted gene’s normalized expression was compared to the non-targeting control strain using gene-specific PCR primers (**S1 Table**). This analysis showed significant silencing of the target genes in eight of the ten knockdown strains (**S2 Fig**). The ABC transporter gene did not show significant silencing at stationary phase by qRT-PCR. We did not rule out the possibility that off-target amplification of another chromosomal region accounted for this apparent escape from silencing.

In order to use our knockdown strains for initial validation of our Tn-seq results, they were combined and used to inoculate erythromycin-selective amniotic fluid (one of the three samples that was used for Tn-seq) and control broth. At the time of inoculation (T_0_), an aliquot of each combined sample was used for plasmid DNA extraction. After 24 hours of competition (T_24_), plasmid DNA was purified from each broth and amniotic fluid sample. We used protospacer-specific quantitative PCR primers to characterize T_0_ and T_24_ GBS as a CRISPRi knockdown or non-targeting control. We used the qPCR results to calculate competition indices for the knockdowns, then compared competition indices in amniotic fluid to broth. The experimental design is diagrammed in **S3 Fig**. **Fig 2C** shows the results from two replicates of this experiment and demonstrates an expected competition defect in amniotic fluid for the five candidate knockdown strains.

### A targeted knockout of mrvR shows a growth defect in human amniotic fluid

*MrvR* showed a consistent CRISPRi knockdown pattern, where both targeting protospacers generated a fitness defect in amniotic fluid. We selected this gene for further study based on these results and the hypothesis that this transcription factor may bridge microenvironmental changes associated with exposure to amniotic fluid and gene expression responses that promote amniotic fluid survival and possibly other GBS virulence traits.

Using pMBsacB, we generated an isogenic knockout of W903_RS09645, the *mrvR* ortholog in 10/84. We also complemented the gene deletion by cloning the full coding sequence of W903_RS09645 and its upstream promoter sequence into the shuttle vector pBSU101 (replacing the green fluorescent protein and *cfb* promoter sequence originally cloned into pBSU101) [93] and transforming the knockout with this plasmid.

We tested the knockout and complemented strains for survival defects in human amniotic fluid and in broth. **Fig 2D** shows growth curves of the wild type and knockout strains in tryptic soy broth, indicating that growth kinetics between the two strains in rich media are identical. **Fig 2E** shows results of competition assays in which amniotic fluid and broth were inoculated with a mixture of wild type 10/84 and the knockout strain or its complement. After 24 hours of outgrowth, these mixtures were plated for CFU enumeration on selective and nonselective media, permitting determination of the relative survival of the strains in competition.

We observed that in broth the knockout strain had a mild survival defect relative to wild type—despite similar growth kinetics in monoculture conditions—but that the defect was more severe in amniotic fluid, and that both defects could be complemented through expression of the full-length *mrvR* gene.

### MrvR controls GBS biofilm formation

We observed phenotypic differences in growth of the *mrvR* knockout as compared to wild type 10/84. In liquid media, the mutant seemed to aggregate in clumps with less coating of the outgrowth vessel, although there were no obvious morphologic differences when the cells were examined by light microscopy after Gram stain (**S4 Fig**). We suspected that the knockout was biofilm deficient, which we confirmed using a biofilm assay. The biofilm index is the ratio of crystal-violet stained biofilm (following overnight growth in a multi-well plate) divided by the planktonic-phase optical density from the same sample. **Fig 2F** confirms that the knockout mutant is biofilm deficient compared to wild type 10/84, and that the biofilm phenotype is restored in the complemented strain.

### MrvR activity does not affect vaginal colonization

To test whether *mrvR* is necessary for persistent vaginal colonization, we colonized healthy adult female mice with knockout GBS or wild type control and performed serial surveillance swabs using an established protocol [13]. After estrus synchronization with β-estradiol administration, nonpregnant adult C57BL/6J mice were colonized with equal inoculums of stationary-phase wild type 10/84 or *mrvR* knockout GBS. Following colonization, the mice were single-housed and screened for colonization persistence three times weekly (starting 48 hours after colonization) by plating vaginal swab samples on GBS-specific chromogenic agar. We did not include the complemented knockout in this experiment because of past observations that, without ongoing *in vivo* antibiotic selection, plasmid DNA is rapidly cured by colonizing GBS.

**Fig 3:**
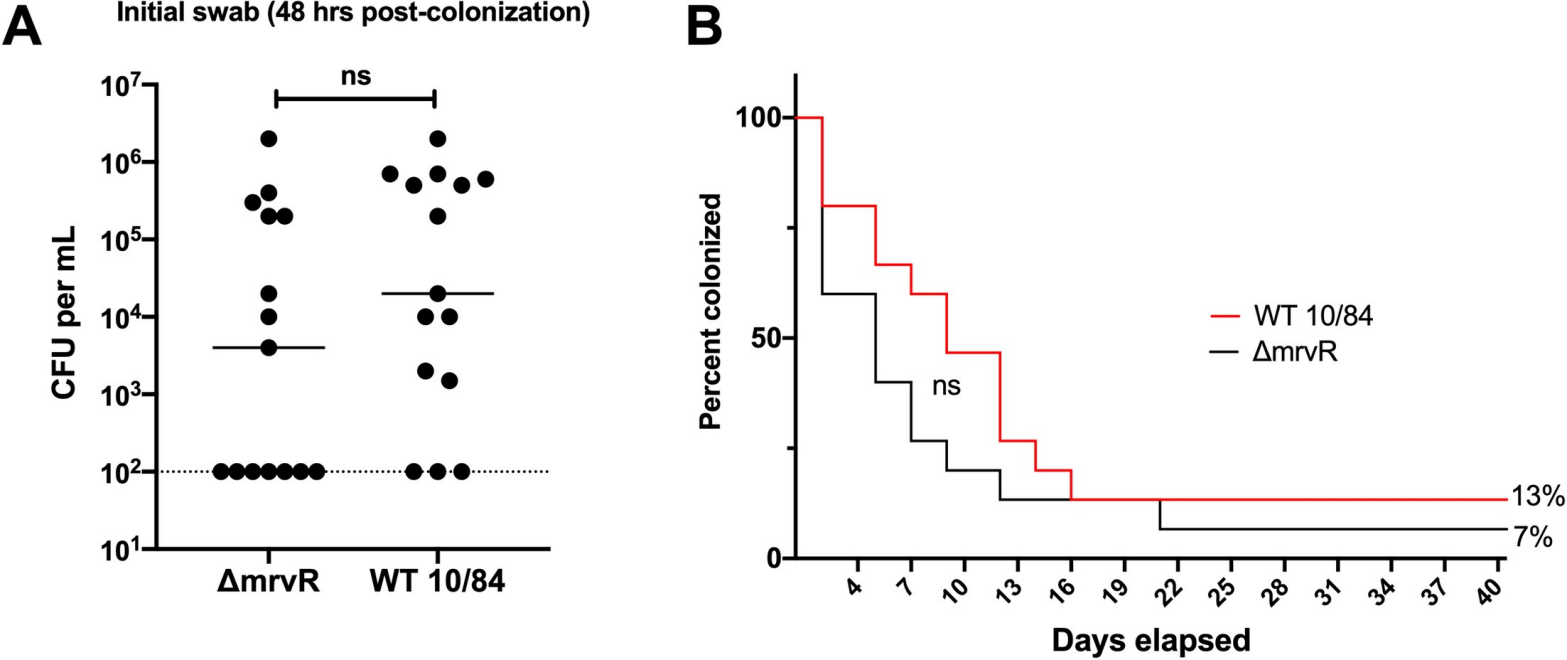
MrvR does not affect establishment or maintenance of vaginal colonization. Adult nonpregnant C57/BLJ mice were vaginally colonized with wild type (WT) 10/84 or Δ*mrvR*. Neither the colonization density at the initial swab 48 hours after inoculation (**A**; t test) nor duration of colonization (**B**; Mantel-Cox test) showed significant differences (CFU: colony forming units, ns: not significant).

While there was a trend toward earlier clearance among the mice colonized with the mutant strain, neither initial colonization density nor eventual clearance among the two groups differed significantly (**Fig 3**).

### The MrvR knockout causes chorioamnionitis without preterm delivery in a mouse model

To test the effect of *mrvR* in ascending chorioamnionitis, we used an established mouse model, vaginally colonizing pregnant female mice on pregnancy day 13 with either wild type 10/84 or the *mrvR* knockout.

Colonized pregnant mice were monitored through pregnancy day 17. Preterm delivery between day 13 and day 17 by a colonized mouse was one experimental endpoint, while animals that remained pregnant on day 17 were sacrificed and assessed for persistent vaginal colonization and ascending chorioamnionitis. Chorioamnionitis was established based on any of the following: GBS present in placental homogenates, GBS present in fetal homogenates, or GBS present in amniotic fluid. Data from mice that were no longer vaginally colonized on day 17 were not included in the analysis; none of the mice who cleared their vaginal colonization had chorioamnionitis.

**Fig 4:**
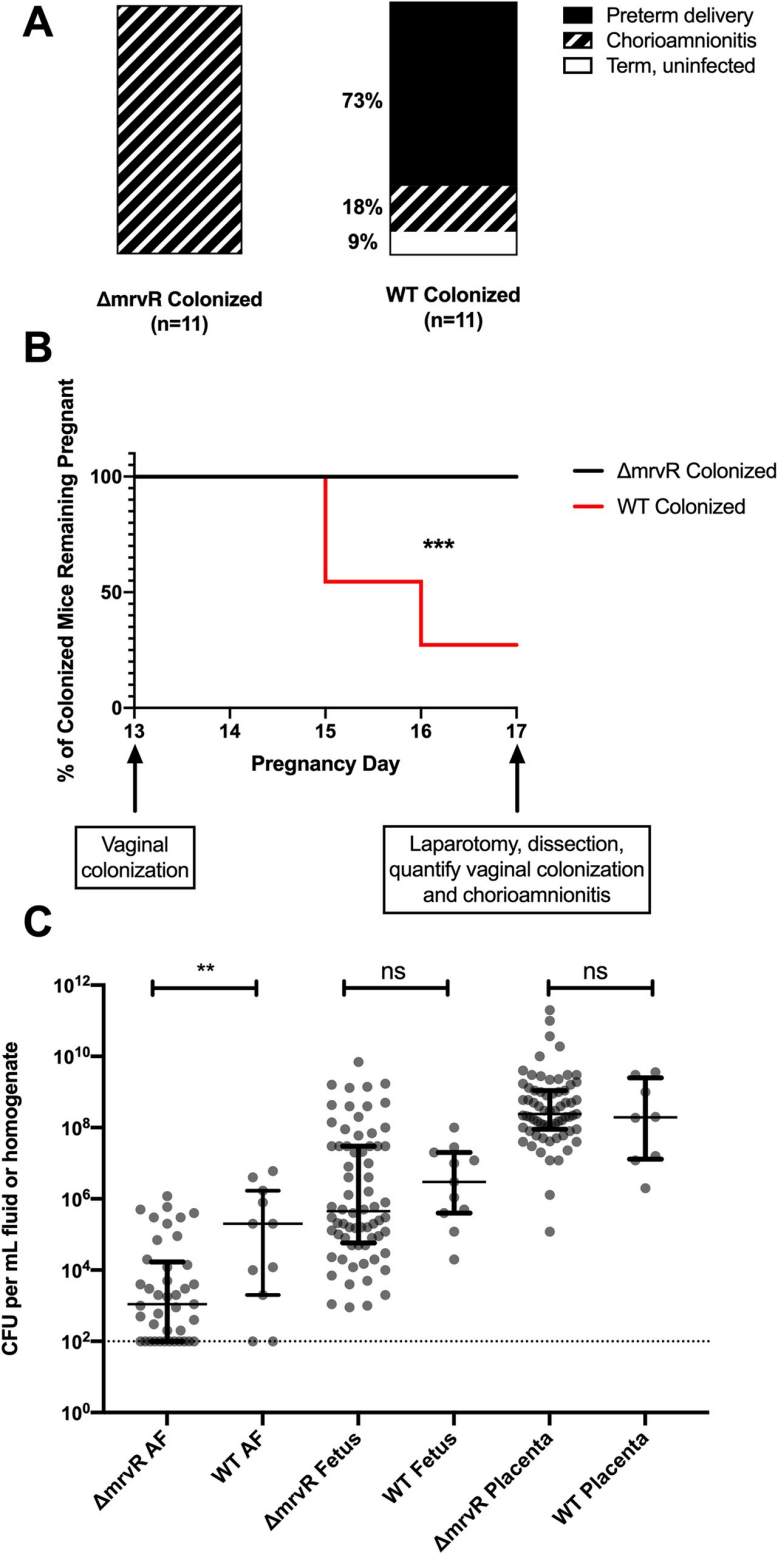
MrvR has a significant effect on the outcome of chorioamnionitis. Pregnant BALB/c mice were vaginally colonized with wild type (WT) or knockout (KO) 10/84 on pregnancy day 13. Pregnancies were monitored until day 17 or preterm delivery, whichever came first. On day 17, mice that remained pregnant were dissected and evaluated for vaginal colonization and chorioamnionitis. While none of the KO-colonized mice with chorioamnionitis delivered preterm, 8 of 11 WT-colonized mice delivered early. One WT-colonized mouse did not develop chorioamnionitis (**A-B**; *** p<0.005; Mantel-Cox test). Dissected pregnancy tissue was homogenized and quantified, showing a difference in invasion density in amniotic fluid (AF) but not in fetal or placental tissue (**C**, ** p<0.01; t test, error bars show interquartile ranges surrounding medians; ns: not significant).

There was a significant difference between pregnancy outcomes for mice colonized with wild type GBS and those colonized with the knockout. Whereas eight of eleven mice colonized with wild type GBS delivered preterm, none of the eleven mice colonized with the knockout strain did. However, all of the knockout colonized mice had chorioamnionitis with widespread GBS dissemination throughout the placentas, fetuses, and amniotic fluid. Of the mice colonized with wild type GBS, two of the three that carried their pregnancies through day 17 had chorioamnionitis (**Fig 4A and 4B**).

Colony counts from tissue and amniotic fluid samples collected at laparotomy showed a significant difference between wild type and knockout GBS density in amniotic fluid but not placental or fetal tissue. Bacterial density was higher in placental homogenates than in fetal tissue homogenates and amniotic fluid for both GBS strains, suggesting the placenta as the portal of infection (**Fig 4C**).

### MrvR is necessary for lethal invasive bacteremia in a murine sepsis model

To assess the role of the transcription factor in bloodstream invasion and systemic infection, we performed intraperitoneal injections of knockout and wild type GBS in healthy adult BALB-c mice. Both bacterial strains were transformed with a toxin-antitoxin stabilized plasmid expression system that generates constitutive luciferase, permitting *in vivo* monitoring of bacterial spread upon bacterial exposure to luciferin cofactor.

The infected mice were photographed using an IVIS *in vivo* imaging system at the time of initial intraperitoneal infection, then daily afterward until either death or complete clearance of the luciferase signal. Surviving mice that had cleared their infection based on imaging were subsequently observed for the following five days, and all remained well-appearing with normal behavior, eating, and stable weights throughout this post-imaging observation period.

**Fig 5:**
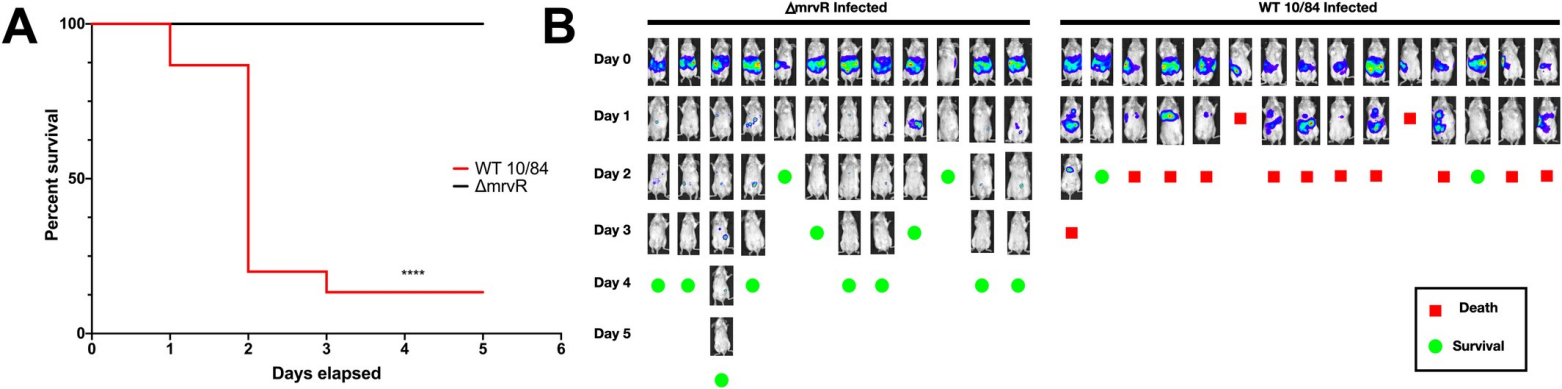
MrvR affects GBS invasiveness and lethality in a sepsis model. BALB/c mice were infected intraperitoneally with wild type (WT) 10/84 or Δ*mrvR*, both bearing a plasmid to allow *in vivo* tracking of a luciferase signal. There was a significant difference in outcome between the two strains (**A**, **** p<0.001 Mantel-Cox test). Mice were imaged daily (**B**) until they either died (red boxes) or completely cleared the luciferase signal (green circles). Mice that cleared the luciferase signal were monitored for the following five days and remained well.

This experiment revealed a significant difference in the ability of infected mice to clear mutant and wild type GBS. Whereas 87% of mice infected with wild type GBS died of the infection within three days, none of the mice infected with the knockout strain died. *In vivo* imaging showed that the mice were able to contain and clear the knockout strain, while the wild type strain tended to invade beyond the peritoneum such as into the thorax, a finding that was invariably followed by death (**Fig 5**).

### Whole-genome transcriptomics shows that MrvR affects a widespread genetic network including multiple operons involved in nucleotide metabolism

We performed whole-genome RNA-seq on wild type GBS and the *mrvR* knockout in order to gain a preliminary understanding of the transcription factor’s regulon. We purified RNA from liquid cultures of the two strains at three growth time points: early log phase, late log phase, and stationary phase.

**Fig 6:**
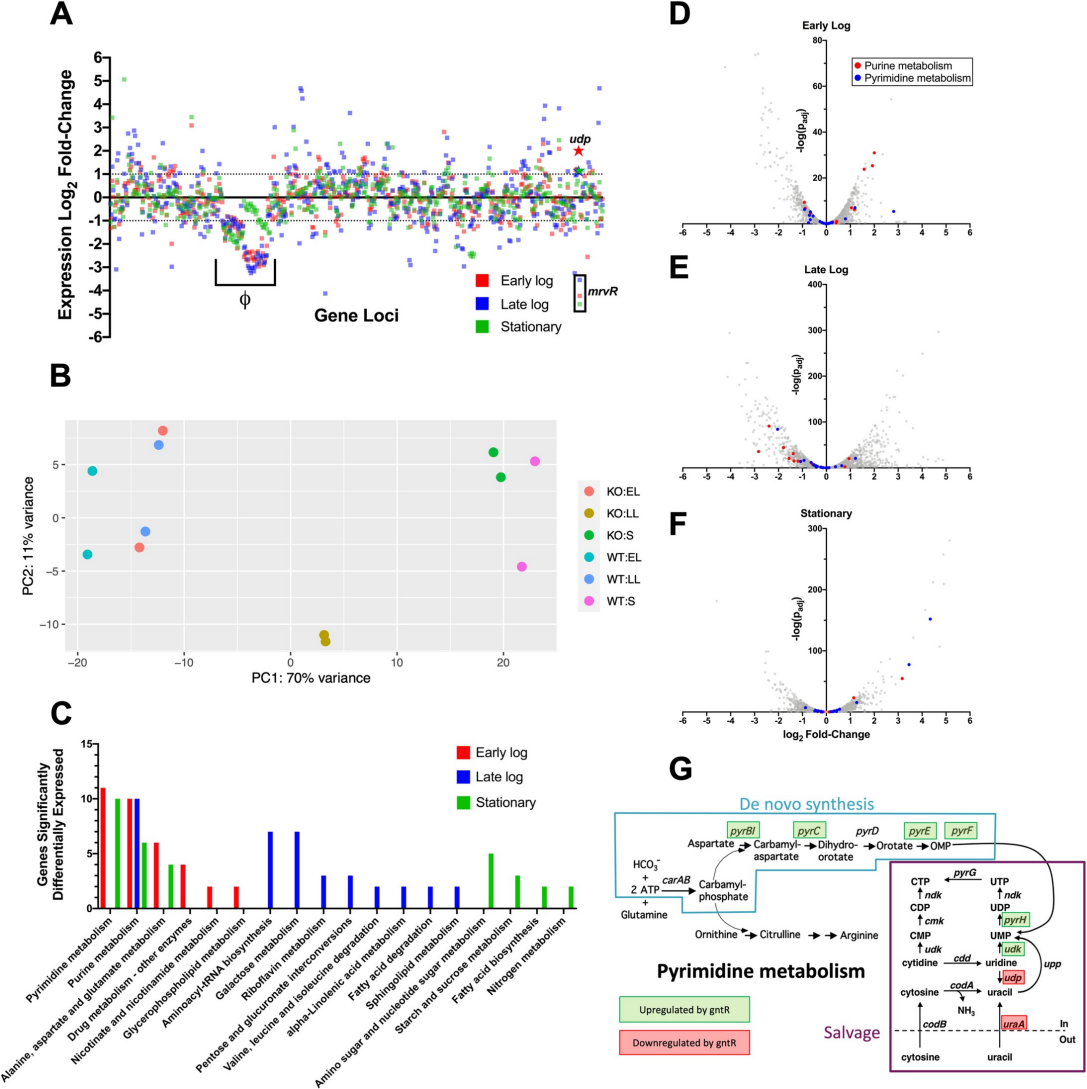
Analysis of the MrvR regulon reveals widespread effects with emphasis on genes involved in nucleotide metabolism. RNA-seq analysis of wild type (WT) and Δ*mrvR* 10/84 at three growth timepoints showed extensive regulation across the chromosome in all phases of growth (**A**, expression log_2_ fold-change shown relative to WT). A phage island (Φ) with significant differences from WT is noted, as are the *udp* gene (stars) and *mrvR*, which was deleted in the knockout strain. Principal component analysis showed isolated clustering of the late log phase replicates of the knockout strain (**B**). Gene set enrichment analysis showed that, across all three bacterial growth phases tested, the KEGG pathways most influenced by the presence of MrvR were pyrimidine and purine metabolism (**C**), which is also shown in volcano plots (**D-F**). Panel **G** shows specific genes of the de novo and salvage pathways of pyrimidine metabolism in GBS whose expression is significantly affected by the presence of MrvR (KO = Δ*mrvR* knockout; EL = early log; LL = late log; S = stationary).

Comparing gene expression between the knockout and wild type GBS demonstrated that a substantial fraction of GBS transcription is controlled directly or indirectly by MrvR (**Fig 6A and S2 Data**). 16.8 percent of genes showed at least a 2-fold change in expression in the knockout relative to wild type during early logarithmic growth. This total increased to 35.7 percent in late logarithmic phase before dropping to 19.9 percent in stationary phase. A principal component analysis of the RNA-seq data showed clustering of the two knockout late log samples apart from both strains in early log phase and stationary phase (**Fig 6B**), reinforcing the concept that MrvR exerts its major regulatory influence during the transition from late log to stationary growth. A correlation analysis using sequence counts across the entire genome showed reproducibility between technical replicates and also that the knockout late log expression profile occupied its own clade on hierarchical clustering (**S5A Fig**). Heatmaps of the top 500 differentially expressed genes (**S5B Fig**) demonstrate distinct but reproducible profiles across conditions.

Notably, a prophage island from W903_RS03075 through W903_RS03520 showed significant downregulation in the knockout at all three phases of growth. In order to confirm that this prophage DNA remained present in the knockout and had not undergone excision, we performed PCR amplification of three prophage regions using genomic DNA template from the wild type and mutant GBS strains (**S6A and S6B Fig**). We also used qRT-PCR on cDNA from separate cultures to validate our RNA-seq data that showed decreased expression of genes within the prophage island in the mutant (**S6C Fig**).

We used a publicly available gene set enrichment search tool [94] to assess the differential gene expression data for functional patterns. This analysis revealed that genes involved in purine and pyrimidine metabolism were significantly enriched among the set of differentially expressed genes and that these nucleotide metabolism genes showed differential expression across all three growth phases. Other functional categories that showed significant differential expression tended to only be affected in one or, at most, two phases of growth (**Fig 6C–6G**).

The *mrvR* gene is located next to a uridine phosphorylase gene (W903_RS09640), which encodes the Udp enzyme that converts uridine to uracil. The intergenic region between *mrvR* and *udp* is 158 nt. The *udp* gene appears coregulated by MrvR, based on RNA-seq data indicating significant upregulation of *udp* expression in the knockout strain relative to wild type. Intriguingly, the arrangement of MrvR adjacent to nucleotide metabolic genes—particularly uridine phosphorylase genes—is highly conserved across Gram-positive bacteria (**S7 Fig**).

## Discussion

GBS can survive and grow in diverse environments within the human host. It can persist in the intestine, in the male and female reproductive tracts, within the placenta, the amniotic fluid, human blood, joint spaces, the neonatal lung, and the central nervous system [1,9,12,33,95–99]. This environmental tolerance contributes to GBS virulence, allowing it to invade and grow within compartments that are prohibitive to other bacteria.

This study began with a Tn-seq examination of genes that promote GBS fitness in amniotic fluid. GBS survival within human amniotic fluid contributes to its ability to cause ascending chorioamnionitis, which can lead to preterm labor and early-onset sepsis, sometimes in combination—a circumstance that can result in delivery of a severely infected preterm newborn with multi-organ system dysfunction [14,100–103]. Intraamniotic infection can also result in stillbirth [10]. Amniotic fluid has chemical and immunological characteristics that are antimicrobial. The fetus and membranes produce antimicrobial peptides that inhibit bacterial survival, and amniotic fluid is intrinsically poor in nutrients required for bacterial proliferation [104–112].

We identified five GBS genes required for survival in human amniotic fluid. Two of these were predicted to encode transcriptional regulators, two encode surface-associated proteins, and one remains uncharacterized. A previous study that used a proteomic approach to evaluate differential gene expression in GBS strain A909 (the same background used for our Tn-seq library) during conditions associated with colonization and fetal invasion did not identify any of the same genes [113]. Intriguingly, however, that investigation found two differentially expressed proteins (encoded by SAK_RS10135 and SAK_RS09535) whose genes are both two loci away from members of our set.

Our analysis also suggests that a significant set of gene deletions increase GBS fitness in amniotic fluid. We speculate that, when GBS enters the stringent conditions of human amniotic fluid, nonessential accessory gene expression may carry a high cost due to the limited metabolic resources available. If this is the case, removal of genetic pathways that expend energy without enhancing immune defense or required biosynthetic capacity may promote enhanced survival.

This study introduces a novel CRISPRi gene expression knockdown system, which we paired with qPCR to further interrogate the candidates suggested by our Tn-seq screen (65, 68, 69).

Our GBS CRISPRi system—which permits flexible and rapid phenotypic discovery without the challenge and time requirements associated with generating gene deletions on the chromosome—could be used for diverse experimental purposes. In this case, it allowed us to validate Tn-seq results and to decide which candidate gene to further study. We envision additional uses, however, including rapid generation of knockdown libraries for multiplex testing of gene contributions to fitness in GBS and exploration of the roles of essential genes whose expression cannot be eliminated entirely.

In this study, the CRISPRi screen pointed our attention to a GntR-class transcription factor that seemed to play an important role in bacterial fitness in amniotic fluid. While GntR transcription factors are widely distributed throughout the bacterial kingdom, and some have been shown to play a role in virulence [73,77,114–120], this gene’s role in GBS host-pathogen interactions has not been explored. Interestingly, an orthologous gene was previously shown to influence group A *Streptococcus* susceptibility to the mouse cathelicidin CRAMP during murine skin infection [121]. We investigated whether our knockout GBS had altered CRAMP sensitivity, but did not observe an effect. Instead we focused on various GBS phenotypic features that are regulated by this transcription factor.

We confirmed our CRISPRi knockdown findings with survival assays in broth and human amniotic fluid, using an in-frame deletion of the transcription factor, which we competed against the wild type parent strain. Unexpectedly, the MrvR knockout was visibly deficient at forming biofilms, which we subsequently confirmed with formal biofilm assays. GBS biofilms have been posited to influence colonization and host immune evasion in GBS and other bacteria [16,122–124].

In a murine model of chorioamnionitis, absence of MrvR had a major impact on the ability of GBS to trigger preterm birth, with mice colonized on pregnancy day 13 showing consistent carriage of their pregnancies through day 17, despite extensive uterine invasion of the knockout strain. This is in contrast to the high rates of preterm delivery seen when pregnant mice are vaginally colonized with wild type GBS, which was observed in this study and in prior work [13] and has been documented in other animal models and clinical practice [14,29].

This finding adds to accumulating evidence that GBS invasion of the fetoplacental unit is not, in itself, sufficient to trigger preterm delivery. A previous study demonstrated similar findings in an examination of the role of β-hemolysin/cytolysin in chorioamnionitis and preterm birth [13]. Several others have shown that consequential inflammatory cascades are triggered in the choriodecidua following GBS invasion [14,24,30,125]. We hypothesize that these inflammatory cascades are enhanced by expression of virulence factors regulated by MrvR, such that its absence ameliorates the inflammation that leads to preterm birth without preventing tissue invasion; this hypothesis will be the focus of additional future work.

Our work also shows that pathways regulated by MrvR are critical for bloodstream invasion and host immune evasion. In an adult mouse model of sepsis, dramatic differences were seen between wild type and knockout GBS in terms of systemic spread and eventual mortality.

Our transcriptomic analysis of the *mrvR* knockout suggests that this transcription factor is involved in mediating nucleotide metabolism. Purine and pyrimidine sensing, biosynthesis, and salvage all play key roles in modulation of virulence traits in other bacterial pathogens [126–128]. Furthermore, intracellular nucleotide homeostasis is highly regulated through diverse signaling pathways [126,129,130], lending plausibility to the prospect that the transcription factor under study here also participates in maintaining nucleotide concentrations.

It is interesting and potentially informative that the major virulence effects of MrvR seem to be manifest in anatomic compartments that are normally sterile (the uterus, peritoneum, and bloodstream), whereas its effects in the nonsterile vaginal colonization model were minimal or altogether absent. We speculate that the presence of a diverse microbiota in the vaginal milieu may complement whatever nucleotide biosynthetic pathway or pathways MrvR serves to modulate in the sterile environments associated with invasive disease.

We used two GBS strains for this study: A909 and CNCTC 10/84. A909 is a serotype Ia strain that was a clinical isolate originally collected by Rebecca Lancefield [131]. 10/84 is a serotype V strain, also a clinical isolate [132], with a hyperpigmented, hypervirulent phenotype observed in a subset of disease-causing GBS [25]. Both strains have been used in numerous GBS pathogenesis studies. The MrvR protein sequence is identical in the two strains except for a single phenylalanine (10/84) to leucine (A909) difference at position 191 of 245. Prior work demonstrated that the orange pigmentation and hypervirulence of 10/84 result from overexpression of the ornithine-rhamnopolyene β-hemolysin/cytolysin, which has cytotoxic properties [26,28–30,133]. Shortly before completion of the *in vivo* experiments for this study, Zhu et al. published data showing that 10/84’s overexpression of β-hemolysin/cytolysin results from a single nucleotide polymorphism in the noncoding promoter region of the *covR*/*covS* two-component regulatory system, resulting in relative under-expression compared to GBS strains without this mutation [134]. The fact that *mrvR* had an effect on GBS survival in amniotic fluid in A909 and 10/84 suggests that its role in 10/84 is not due to the altered *covR*/*covS* expression, but future work will be applied toward determining the role of *mrvR* across multiple GBS strains, as well as seeking to better understand its exact molecular mechanism, including its DNA binding sites and effector molecule.

In conclusion, this study applied genome-wide screens, novel and established molecular techniques, and multiple murine models of GBS colonization and invasive disease to identify and characterize a GntR-class transcription factor, MrvR, with key roles in multiple virulence-related phenotypes. This newly described transcription factor presents an appealing subject for further mechanistic study and may be a target for novel pharmaceutical approaches to limit GBS virulence through modulation of this molecule’s activities.

## Methods

### Ethics statement

Animal experiments were performed under approved IACUC protocols at NYU (s16-01356) and University of Pittsburgh (20016575). Adult phlebotomy for hemolysis assays was conducted under a University of Pittsburgh approved IRB protocol (19110106). Collection of anonymous, discarded amniotic fluid was conducted under an approved exemption from the NYU IRB.

### Statistical analyses

Statistics for Tn-seq were determined with ESSENTIALS software [50] and RNA-seq statistical tests were performed with DESeq 2 [135], using visualization packages in R to generate principal component analysis and heatmap images. Remaining statistics for figures were calculated using Prism for macOS v. 8.4.3 (Graphpad Software, San Diego, CA).

### Bacterial strains and growth conditions

GBS strains A909 (serotype Ia, sequence type 7) and CNCTC 10/84 (serotype V, sequence type 26) and their derivatives were grown at 37°C (or 28°C when the temperature-sensitive pMBsacB plasmid was present and extrachromosomal) under stationary conditions in tryptic soy (TS) medium (Fisher Scientific cat. # DF0370-17-3), supplemented with 5 μg/ml erythromycin, 150 μg/ml spectinomycin, or rifampin 10 μg/ml as needed for selection. *Escherichia coli* was grown at 37°C (or 28°C with extrachromosomal pMBsacB present) with shaking in Luria-Bertani (LB) medium (Fisher Scientific cat. # DF9446-07-5) supplemented with 300 μg/ml erythromycin and 150 μg/ml spectinomycin as needed for selection.

### Tn-seq on GBS grown in human amniotic fluid

Discarded, anonymous amniotic fluid from amniocentesis was obtained from the NYU-Langone Medical Center Department of Obstetrics and Gynecology. Samples were stored frozen at -20°C, then thawed on ice. Before use in outgrowth experiments, the amniotic fluid was passed through a 0.2 μm sterile filter.

A previously described saturated transposon mutant library in an A909 background [41] was thawed from frozen stock. The library was washed twice in sterile PBS to remove glycerol cryoprotectant, then resuspended in 3 mL PBS and seeded into 100 mL of TS broth with erythromycin selection. After overnight outgrowth, 20 mL of the library was seeded into fresh TS broth with erythromycin and allowed to grow to mid-log phase (OD_600_ = 1). 120 mL of this culture was then pelleted and washed twice with PBS, then resuspended in 1 mL of PBS.

50 μL of this concentrated library was then used to seed either 5 mL of amniotic fluid pre-warmed to 37°C or control TS broth. The seeded amniotic fluid and TS was allowed to grow overnight. Unseeded control amniotic fluid samples were also incubated and plated the following morning to ensure that there was no contamination present.

After 16-hour outgrowth, the TS and amniotic fluid samples were transferred into 250 mL aliquots of TS. These were grown overnight, then genomic DNA was purified from 90 mL of each culture using the MoBio Powersoil DNA Extraction Kit (cat. # 128888) according to manufacturer instructions. Purified DNA was subsequently digested with MmeI and used for barcoded terminal adapter ligation, PCR, and sequencing on a 150-nt paired-end run of the Illumina (San Diego, CA, USA) HiSeq 4000 platform, with a target number of reads per library of 50 million and subsequent demultiplexing as previously described [41,42].

We used ESSENTIALS, a publicly available Tn-seq analysis server, to analyze our data [50]. ESSENTIALS generates a plot of genome-wide log_2_ fold-change values for actual-versus-expected transposon detection. Bimodality in this plot indicates separate gene populations—those with approximately the expected number of transposon insertions detected in the experimental condition, and those with fewer than expected detected insertions. The latter group represents the set of conditionally essential genes, and the local minimum between the two modal peaks can be used to generate a cutoff value between nonessential and conditionally essential genes. In the case of our amniotic fluid Tn-seq experiment, the local minimum was at a log_2_ fold-change value of -3.22. The five candidate genes evaluated had log_2_ fold-change values below this cutoff (**S1 Data**).

### Development of a GBS CRISPRi system for gene expression knockdown

To generate GBS expressing dCas9, we used the mutagenesis plasmid pMBsacB in GBS strain 10/84 as previously described [88]. Two separate synthetic, double-stranded DNA fragments, 800 to 1000 nt in length, were designed and ordered from Genscript (Piscataway, NJ, USA). The fragments matched the native 10/84 *cas9* coding sequence except for the two missense mutations, D10A and H840A, needed to render the enzyme catalytically inactive. These fragments were cloned into pMBsacB using Gibson assembly and used to transform chemically competent DH5α *E*. *coli* (New England Biolabs, Ipswich, MA, USA) according to manufacturer instructions. We confirmed proper cloning by Sanger sequencing of miniprepped plasmid DNA, which we then used to transform electrocompetent GBS as previously described [88,136,137]. We performed the C-terminal (H840A) mutagenesis first, following the protocol we have described previously [88]. Once this mutation was generated and confirmed by Sanger sequencing, we made those GBS competent and created the second mutation using identical techniques.

p3015b is a shuttle vector originally derived from pVPL3004 [67]. We used PCR, synthetic DNA, and Gibson assembly approaches to make multiple changes to this plasmid for use in GBS. The kanamycin resistance cassette was changed to the erythromycin resistance gene; the *cas9* gene from *S*. *pyogenes* was removed; and the tracrRNA sequence downstream of the dual BsaI restriction sites used for protospacer introduction was replaced with a new sequence that conforms to the predicted folding structure of the native GBS tracrRNA sequence (**S1 Fig**). We also sought to introduce anhydrotetracycline inducibility of sgRNA expression by cloning a *tetR* coding sequence and a 2x tetO inducible promoter upstream of the sgRNA coding sequence. Despite extensive efforts, however, we could not suppress baseline sgRNA expression in GBS to the point that gene knockdown was inducible, so the plasmid was used for constitutive sgRNA expression in GBS without anhydrotetracycline addition.

Targeting protospacers were designed using the Broad Institute’s online sgRNA design tool [89,90]. Target sequences were selected based on their antisense orientation and, where possible, position in the first half of the coding sequence. Protospacer oligonucleotide sequences were ordered from Integrated DNA Technologies (Coralville, IA, USA) and cloned into BsaI-digested 3015b as originally described by Jiang et al. in their description of pCas9, from which pVPL3004 was developed [138].

### qRT-PCR testing for gene silencing

Each of the ten CRISPRi knockdown GBS strains and the non-targeted control strain were grown in TS broth to stationary phase. RNA was purified using the Ribopure Bacteria RNA Purification Kit (Thermo cat. # AM1925), then DNA-depleted with DNase treatment as per kit instructions. The DNase treatment was repeated twice to eliminate any trace contamination, then cDNA was reverse transcribed using the Applied Biosystems High-Capacity Reverse Transcription Kit (cat. # 4368814). A reverse transcriptase-negative control condition was performed for each sample. qRT-PCR was performed with Bio-Rad SSOAdvanced Universal SYBR Green Supermix (cat. # 1725270) and oligonucleotide primers specific for each of the five targeted genes. The GBS *recA* gene was used as a normalization control. Reactions were performed on a Bio-Rad CFX96 real time PCR thermocycler. After normalization to *recA* expression, each target gene’s expression was compared between the knockdown strain and the non-target control strain. This experiment was performed with triplicate samples across two independent replicates.

### CRISPRi multiplex competition assay

Cultures of 10/84 dCas9 transformed with p3015b plasmid bearing targeting protospacers against candidate conditionally essential genes were grown overnight in selective broth. A control strain with p3015b plasmid containing a sham protospacer with no matching sequence in the GBS chromosome was grown as a control. The 11 cultures were spun down and washed twice in PBS, then resuspended and normalized to an OD_600_ of 0.8. These suspensions were then plated as serial dilutions to confirm that the starting CFU concentrations were approximately equal.

500 μL from each of the 11 normalized GBS suspensions were then combined to make a starting mixed culture. A sample of this mixed culture was used for plasmid DNA purification using the Thermo MagJET plasmid DNA Kit (cat. # K2791) according to manufacturer instructions.

Amniotic fluid or TS broth containing erythromycin for continued plasmid selection was pre-warmed to 37°C and seeded 1:50 with the mixed culture, then allowed to grow stationary overnight. 24 hours after seeding, plasmid DNA was extracted from the amniotic fluid and TS samples.

At both the starting and 24-hour timepoints, prior to purification, DNA from dead bacteria was removed using ethidium monoazide using techniques described by Soejima et al. [139]. Ethidium monoazide—which binds to extracellular DNA or DNA within dead bacteria, but not to DNA within live cells—was added to a final concentration of 10 μg/mL, after which the cultures were allowed to incubate at 4°C for five minutes. They were then placed under a high intensity white light source, which destroys DNA bound to ethidium monoazide. The treated samples were then spun down, washed once in PBS, then used for plasmid purification.

qPCR on purified plasmid samples from the initial and 24-hour timepoints was performed using Applied Biosystems Power SYBR Green Master Mix (Thermo cat. #4368577) on a BioRad (Hercules, CA, USA) CFX384 real-time PCR thermocycler. The different plasmids present were detected using a conserved reverse primer and a forward primer complementary to either one of the targeting protospacer sequences or the sham protospacer sequence. We also used a pair of normalization primers complementary to plasmid sequences distant from the protospacer cloning site. This strategy allowed determination of relative abundances of each of the targeting protospacers (and the sham control) relative to the total plasmid concentration in each sample.

Competition indices for each knockdown strain were calculated using the formula: ln((NE^target^_T24_/NE^target^_T0_)/(NE^sham^_T24_/NE^sham^_T0_)) where NE is normalized expression scaled to total plasmid quantity, target and sham reflect targeting and control plasmids, and T0 and T24 represent samples from the initial mixed culture or from the 24-hour timepoint, respectively. qPCR was performed twice, with two to three technical replicates per primer pair. Competition indices for the knockdown strains were normalized to survival for each strain in TS broth.

### Generation of the mrvR knockout and its complemented control strain

A synthetic double-stranded DNA fragment was designed to contain the chloramphenicol acetyltransferase gene surrounded by 500-nt homology arms that match upstream and downstream sequences flanking the *mrvR* open reading frame in 10/84. This mutagenesis cassette was cloned into pMBsacB using Gibson assembly, then used to transform electrocompetent wild type 10/84. Temperature- and sucrose-based selection and counterselection were used to isolate single-cross and double-cross plasmid insertion and excision mutants, which were confirmed using targeted PCR and Sanger sequencing as previously described [88].

To complement the gene deletion, the wild type 10/84 *mrvR* gene and 75 nt of upstream promoter sequence were amplified using PCR and cloned into pBSU101 in place of the *cfb* promoter and green fluorescent protein gene present in the original plasmid [93]. This plasmid was then used to transform the knockout strain, using spectinomycin for positive selection. Photographs and light micrographs of wild type 10/84, the *mrvR* knockout, and the complemented control strain at stationary phase are included in **S4 Fig**.

### Amniotic fluid competition assays with CFU quantification

In order to identify wild type and knockout genetic backgrounds following competition in amniotic fluid, we used strains with common antibiotic resistance phenotypes that emerge from isolated missense mutations from single nucleotide polymorphisms. Amniotic fluid or TS broth was seeded with either a) a 10/84 strain with a defined mutation in the *rpoB* gene that confers rifampin resistance [140] and a spontaneous streptomycin-resistant *mrvR* knockout clone or b) the rifampin-resistant 10/84 strain transformed with empty pBSU101 (for spectinomycin resistance) and the complemented, streptomycin-resistant knockout strain. After 24 hours of outgrowth, these mixtures were plated for CFU enumeration on nonselective media, rifampin-containing media, and streptomycin-containing media. This permitted determination of total growth and allowed discrimination between the knockout and wild type strains.

Competition indices were calculated using colony forming unit (CFU) density per mL, where competition index = ln((CFU^expt^_T24_/CFU^expt^_T0_)/ (CFU^WT^_T24_/CFU^WT^_T0_)); here, expt represents the experimental strain (either knockout or control) and WT is wild type 10/84. Assays were performed with three replicates for the knockout and four replicates for the complemented strain. The experiment was repeated twice for each strain; data in **Fig 2** is from a representative replicate.

### Hemolysis assays

Whole cell GBS was used in hemolysis assays on washed human erythrocytes as previously described [42]. The assay was performed in triplicate and the experiment was repeated twice. Data in **Fig 2** is from a one of the experimental replicates.

### Growth curve analysis

Wild type and *mrvR* knockout GBS were grown to late logarithmic phase in TS broth. The two cultures were then normalized to an OD_600_ of 1.0, then diluted 1:50 in broth. Triplicate samples were then instilled into a clear, flat-bottom 96-well plate, the lid of which had been treated with sterile defogging solution. OD_600_ absorbance readings were taken every 10 minutes, with a brief shaking step before each read, using a Molecular Devices (San Jose, CA, USA) SpectraMax M4 plate reader set to 37°C. The experiment was repeated twice with triplicate growth wells for each strain; data shown are from one experimental replicate.

### Biofilm assays

10 μL of stationary-phase GBS was used to seed 1 mL of fresh broth with appropriate antibiotic selection and 5% glucose supplementation in a 12-well, clear-bottom plate, which was then allowed to grow for 24 hours at 37°C. Preparations were then photographed and 10 μL of supernatant was used to prepare a Gram stained slide for microscopy following standard protocols (**S4 Fig**). The plate was rocked gently on an orbital shaker for 1 minute to resuspend non-adherent bacteria, then the media was carefully removed and transferred to a fresh plate. OD_600_ absorbance readings of the planktonic phase bacteria were recorded.

The plate that grew overnight was then used to stain the residual biofilms. 500 μL of 1% crystal violet was carefully added to each well, being cautious not to disturb the biofilm. After 10 minutes of gentle shaking at room temperature, the stain was removed and each well was washed three times in 1 mL of sterile PBS. After the final wash, the stained biofilm was allowed to dry, then solubilized with 1 mL of a 20% acetone/80% ethanol solution. Absorbance at 550 nm was measured. Biofilm indices were calculated as OD_550_/OD_600_. The experiment was performed three times with twelve technical replicates for each condition. Data in **Fig 2** is from a representative experimental replicate.

### Murine vaginal colonization model

Vaginal colonization of nonpregnant C57BL/6J mice was performed as previously described by Randis et al. [13] with minor modifications.

6- to 8-week old mice were injected subcutaneously with 0.5 mg β-estradiol on two successive days to synchronize estrus. On the third day, stationary phase GBS was pelleted and resuspended in a 1:1 mixture of PBS and a sterile 10% gelatin solution to enhance viscosity. The mixtures were diluted and plated for CFU enumeration. Mice were vaginally colonized with 50 μL, corresponding to 10^7^ CFU (n = 15 per condition).

Following colonization, mice were single-housed. After 48 hours, an initial swab to detect colonization was performed. A sterile nasopharyngeal swab was moistened in 300 μL sterile PBS, then inserted into the murine vagina and rotated three times, after which it was replaced in the PBS and swirled to release adherent GBS. The PBS was diluted and plated for CFU enumeration on GBS-specific Chromagar plates (Chromagar, Paris, France, cat. # SB282), which were then grown overnight at 37°C.

Mice with established vaginal colonization, based on the 48-hour swab, were then swabbed three times weekly (Monday, Wednesday, and Friday) following the above protocol. Colonization clearance was defined as two sequential swabs with no detectable GBS.

### Chorioamnionitis and preterm delivery model

The murine chorioamnionitis/early-onset sepsis model was performed as previously described [13] with minor modifications. Two- to four-month old BALB/c mice were mated and plug-positive female mice were monitored for pregnancy by physical examination and serial weights.

On pregnancy day 13, mice were vaginally colonized with 10^7^ CFU of either wild type 10/84 or the *mrvR* knockout as in the vaginal colonization model (without the preceding estradiol injections). Following colonization, they were single-housed and monitored daily for preterm delivery. If delivery occurred prior to day 17, this outcome was considered chorioamnionitis with preterm birth. Mice that remained pregnant on day 17 were sacrificed and dissected. Each fetus was examined for signs of intrauterine fetal demise (significant pallor or hyperemia, visible skin sloughing or purulence, or evidence of ongoing fetal resorption). An insulin needle was used to withdraw amniotic fluid from within the fetal membranes, after which placentas and fetuses were isolated and homogenized.

Amniotic fluid and tissue homogenates were diluted and plated on Chromagar plates for CFU enumeration, which was performed after overnight plate growth at 37°C.

The experiment was continued until 11 mice had been successfully colonized with both strains. Four mice inoculated with wild type GBS did not colonize and three mice inoculated with the knockout strain did not colonize. Pregnancy tissue data from these mice are not included in the analysis.

### Generation of luciferase reporter GBS

A toxin-antitoxin stabilized shuttle vector expressing the firefly luciferase gene, pLZ12Km2-P23R:TA:ffluc, was a gift from Thomas Proft (Addgene plasmid # 88900; http://n2t.net/addgene:88900; RRID:Addgene_88900). We used Gibson assembly techniques to excise the kanamycin resistance marker on pLZ12Km2-P23R:TA:Ffluc and replace it with an erythromycin resistance gene. The new plasmid, called pFfluc-Erm, was purified from *E*. *coli* and used to transform electrocompetent wild type 10/84 and the *mrvR* knockout.

In order to fluoresce, GBS bearing the pFfluc-Erm plasmid must be exposed to the cofactor D-luciferin (Millipore Sigma cat. # L9504), which was prepared in a filter-sterilized stock solution at a concentration of 28 mg/mL in water. We performed *in vitro* imaging of serial dilutions of liquid cultures of GBS transformed with pFfluc-Erm combined 1:1 with D-luciferin stock and determined the level of fluorescence detection at 290,000 CFU/mL.

### Adult mouse sepsis model with in vivo imaging

Six- to eight-week old female BALB/c mice were injected intraperitoneally under general anesthesia with 10^8^ CFU of PBS-washed, stationary phase WT 10/84 or *mrvR* knockout 10/84 bearing the pFfluc-Erm plasmid and 500 μg of D-luciferin in 500 μL PBS. Immediately after the injection, mice were imaged on a Perkin-Elmer (Waltham, MA, USA) IVIS Lumina Series II imaging instrument with a 1-minute exposure time.

On subsequent days, surviving mice were injected intraperitoneally with 560 μg D-luciferin in sterile water then imaged after 10–15 minutes using a 1-minute exposure time. Mice that cleared their luciferase signal were subsequently observed for five days.

For both strains, 15 mice were injected. Three mice in the knockout condition died within several minutes of intraperitoneal bacterial injection on the first day of the experiment, with evidence of internal hemorrhage. These mice were assumed to have suffered organ injury related to the injection procedure and were not included in the analysis.

### Whole-genome RNA-seq transcriptomic analyses

Wild type 10/84 and the *mrvR* knockout 10/84 strain were grown in TS broth overnight. 13-mL overnight culture samples were used for stationary phase RNA extraction. This culture was also used to seed pre-warmed broth, which was then grown to an OD_600_ 0.3–0.6 (early log phase) or 0.9–1.1 (late log phase), at which point RNA extraction was performed.

We used the Ribopure Bacteria RNA Purification Kit (Thermo cat. # AM1925) according to manufacturer specifications with the following exceptions: the initial bead beating step was performed for 20 minutes; the early log bacterial RNA was extracted from an 85-mL starting culture while late log and stationary phases were extracted from 13-mL cultures. RNA samples were then treated with the DNase and DNase inactivation reagents supplied with the kit. Two independent cultures for each strain/growth phase combination were used for RNA purification, sequencing, and analysis.

Sequencing library preparation was conducted with the Illumina TruSeq total RNA kit according to manufacturer instructions. rRNA depletion was performed using the Ribo-Zero Plus rRNA Depletion kit. Next, random primers initiated first and second strand cDNA synthesis. Adenylation of 3’ ends was followed by adapter ligation and library amplification with indexing. Sequencing was performed on an Illumina NextSeq500 platform with paired-end 75-nt reads over 150 cycles.

Trimmed and demultiplexed reads were aligned to the 10/84 genome using Bowtie 2 [82]. These alignments were then used for statistical analysis using DESeq 2 [135], which allowed determination of expression fold-change and p values. Gene set enrichment analyses were performed with Genome2D [94].

Confirmation that the 10/84 prophage region was downregulated in the *mrvR* knockout strain was performed in two steps. Initially, PCR of three segments within the prophage region was performed on genomic DNA from separate cultures of wild type and knockout GBS. Gel electrophoresis of these reactions indicated that the entire region remained in the knockout (**S5A Fig**). Next, qRT-PCR on prophage regions 1 and 2 (using primers 1084 Phage qPCR R1 F/R and 1084 Phage qPCR R2 F/R) was performed as described above.

Principal component analysis and generation of the correlation matrix and heatmaps in **S6 Fig** were performed in R.

## Supporting information

S1 Data GBS amniotic fluid conditionally essential gene analysis. Output from ESSENTIALS analysis of eight A909 library aliquots grown in human amniotic fluid. The table is sorted by log_2_ fold-change (D), and the five conditionally essential genes described in the main text are highlighted in yellow. The index (column A) allows sorting by gene locus if set from smallest to largest. At the end of the table there is a kernel density plot, generated by the ESSENTIALS bioinformatic package, which shows log_2_ fold-change values for experimental-versus-control transposon insertions. The leftmost local minimum on that plot (-3.22) provides a stringent criterion for conditionally essential genes and was used to select the five candidate genes discussed in the main text.
(PDF)

S2 Data RNA-seq comparing Δ*mrvR* with wild type 10/84 genome-wide gene expression. Output from DESeq 2 analysis of RNA-seq read alignments. Log_2_ fold-change and adjusted p values are provided for three growth phases as described in the text.
(PDF)

S1 Table Oligonucleotides used in this study. (PDF)

S1 Fig GBS CRISPRi sequence data. Alignments of GBS and *S*. *pyogenes* genomic DNA regions encoding CRISPR-Cas complex components (**A-C**). Predicted folding of gRNA complexes (**D**) generated using the mfold server [141]. Sequence of sgRNA complex used to target candidate genes using GBS CRISPRi system (**E**). Sanger sequence showing the two targeted mutations used to generate dCas9 on the 10/84 chromosome (**F**).
(TIF)

S2 Fig qRT-PCR evaluation of CRISPRi targeted gene expression. Each silenced strain was assessed for normalized expression of the targeted gene relative to a non-targeted control strain. This experiment was performed with triplicate samples across two independent replicates. (*p<0.05, **** p<0.001, ns = not significant; t test against a single value of 1, error bars show standard error surrounding the mean).
(TIF)

S3 Fig Schematic of qPCR-based competition assays between CRISPRi knockdown strains and a non-targeted control strain. Image created with BioRender.com.
(TIF)

S4 Fig Stationary phase growth of wild type, knockout, and complemented strains. Strains were grown in TS broth (with spectinomycin selection for the complemented control strain) and photographed after overnight growth in a 24-well plate (**top**). Microscopic examination of Gram-stained preparations did not reveal obvious differences in cellular morphology (**bottom**).
(TIF)

S5 Fig RNA-seq heatmaps. Genome wide correlation matrix with hierarchical clustering (**A**). Top 500 regulated genes across all RNA-seq samples with hierarchical clustering (**B**).
(TIF)

S6 Fig PCR targeting three regions within the prophage island found to be downregulated in Δ*mrvR*. Three regions within the prophage island spanning gene loci W903_RS03075 through W903_RS03520 were amplified from wild type and *mrvR* knockout GBS genomic DNA. The PCR products from the two strains were the same size when assessed by gel electrophoresis (**A**). The schematic shows the three amplified regions of the chromosome in red (**B**). qPCR validation of RNA-seq data showing decreased expression of RNA from regions 1 and 2 in the *mrvR* knockout compared to wild type (**C**).
(TIF)

S7 Fig *mrvR* conservation. Gene synteny plot showing orthologs of the GBS *mrvR* gene in 16 other bacterial species. The plot was generated using the Integrated Microbial Genomes & Microbiomes server on the JGI genome portal (https://img.jgi.doe.gov). Genes are color coded by COG functional prediction. COG categories related to nucleotide metabolism present on the diagram are noted (**A**). Conservation of the *mrvR* coding sequence among 138 sequenced GBS genomes aligned through NCBI BLAST (**B**). The top of the panel shows percent conservation at each nucleotide sequence along the coding sequence. Mismatches in individual sequences (gray lines) are highlighted and color-coded for nucleotide identity.
(TIF)
