# Supplementary material for: Genome-Wide fitness analysis of group B Streptococcus in human amniotic fluid reveals a transcription factor that controls multiple virulence traits
Source: PLoS Pathog. 2021 Mar 8;17(3):e1009116. doi: 10.1371/journal.ppat.1009116 (PMC7971860; doi:10.1371/journal.ppat.1009116)
Supplement: S1 Data — Output from ESSENTIALS analysis of eight A909 library aliquots grown in human amniotic fluid. The table is sorted by log2 fold-change (D), and the five conditionally essential genes described in the main text are highlighted in yellow. The index (column A) allows sorting by gene locus if set from smallest to largest. At the end of the table there is a kernel density plot, generated by the ESSENTIALS bioinformatic package, which shows log2 fold-change values for experimental-versus-control transposon insertions. The leftmost local minimum on that plot (-3.22) provides a stringent criterion for conditionally essential genes and was used to select the five candidate genes discussed in the main text. (PDF) [file ppat.1009116.s001.pdf]

Index	New Locus Tag	Old Locus Tag	logFC	adj.P.Val	Start	Stop	Strand	Length	Gene
1896	SAK_RS09545	SAK_1896	-4.369043935	2.07E-14	1896329	1897105	+	777	-
588	SAK_RS02930	SAK_0588	-4.334160498	6.17E-14	531495	531797	-	303	-
1965	SAK_RS09890	SAK_1965	-4.186504256	1.08E-11	1955676	1956398	-	723	-
2012	SAK_RS10120	SAK_2012	-3.812349482	1.39E-11	2006402	2007139	+	738	mrvR
1031	SAK_RS05160	SAK_1031	-3.727077207	7.04E-10	1000406	1001017	+	612	-
853	SAK_RS04270	SAK_0853	-2.914281284	0.001172189	800893	802503	+	1611	ftsY
2114	SAK_RS10650	SAK_2114	-2.814015133	0.008276941	2104985	2106094	+	1110	recF
835	SAK_RS04180	SAK_0835	-2.750161096	0.010132895	780699	781697	+	999	-
914	SAK_RS04570	SAK_0914	-2.646573421	0.005020334	864068	864919	+	852	-
648	SAK_RS03245	SAK_0648	-2.625489661	0.005894511	580503	582506	+	2004	-
1774	SAK_RS08930	SAK_1774	-2.5803376	0.001172189	1767695	1768231	-	537	-
1175	SAK_RS05880	SAK_1175	-2.575797821	0.000190588	1152800	1154800	-	2001	-
840	SAK_RS04205	SAK_0840	-2.471913997	0.005020334	787644	788210	+	567	-
1032	SAK_RS05165	SAK_1032	-2.310372707	0.001924826	1001054	1001836	-	783	-
1795	SAK_RS09035	SAK_1795	-2.267894167	0.005048137	1787967	1788908	-	942	-
1900	SAK_RS09565	SAK_1900	-2.230948465	0.005894511	1901627	1903843	-	2217	relA
1556	SAK_RS07845	SAK_1556	-2.16980131	0.008276941	1553936	1554862	-	927	mtsA
1004	SAK_RS05025	SAK_1004	-2.098788616	0.016106467	967197	968954	+	1758	-
85	SAK_RS00420	SAK_0085	-2.038812798	0.049204984	66913	68691	+	1779	-
290	SAK_RS01430	SAK_0290	-2.00828642	0.095981098	250257	250547	+	291	-
84	SAK_RS00415	SAK_0084	-1.950174441	0.082269681	66536	66916	+	381	-
1003	SAK_RS05020	SAK_1003	-1.925319292	0.049887517	965749	967137	+	1389	-
1554	SAK_RS07835	SAK_1554	-1.908204399	0.032039909	1552214	1553047	-	834	mtsC
1510	SAK_RS07605	SAK_1510	-1.88165868	0.036861887	1506407	1507795	+	1389	cycA
1240	SAK_RS06220	SAK_1240	-1.845243257	0.054129749	1210062	1210292	-	231	-
1743	SAK_RS08765	SAK_1743	-1.819369789	0.053769112	1732904	1733563	+	660	-
1306	SAK_RS06550	SAK_1306	-1.791049337	0.104483905	1283629	1284231	-	603	-
1688	SAK_RS08500	SAK_1688	-1.785016451	0.053769112	1674328	1675539	-	1212	-
901	SAK_RS04505	SAK_0901	-1.774037861	0.054129749	850398	851228	+	831	-
1102	SAK_RS05510	SAK_1102	-1.758512696	0.096516319	1075382	1076410	-	1029	-
1033	SAK_RS05170	SAK_1033	-1.707120157	0.082269681	1001848	1002546	-	699	-
1094	SAK_RS05470	SAK_1094	-1.650490124	0.11149771	1065277	1065708	-	432	-
2022	SAK_RS10175	SAK_2022	-1.641370227	0.118937627	2016537	2017028	-	492	-
1555	SAK_RS07840	SAK_1555	-1.631320049	0.114092804	1553049	1553765	-	717	mtsB
303	SAK_RS01495	SAK_0303	-1.629874464	0.11149771	258189	259334	-	1146	-
222	SAK_RS01100	SAK_0222	-1.62701866	0.095981098	186394	188691	+	2298	-
583	SAK_RS02905	SAK_0583	-1.589920056	0.104483905	525869	526474	+	606	-
955	SAK_RS04785	SAK_0955	-1.589056744	0.115099042	910378	912639	+	2262	-
1945	SAK_RS09795	SAK_1945	-1.583339386	0.11297899	1942416	1943444	-	1029	-
301	SAK_RS01485	SAK_0301	-1.577807802	0.11149771	256625	257551	-	927	-
1091	SAK_RS05455	SAK_1091	-1.574983149	0.11149771	1062900	1063313	-	414	spxA
536	#N/A	SAK_0536	-1.568116314	0.104483905	479378	480550	+	1173	galK
688	SAK_RS03440	SAK_0688	-1.545420919	0.104483905	623359	624381	+	1023	add
1002	SAK_RS05015	SAK_1002	-1.542737172	0.13513782	964624	965622	+	999	-
1585	SAK_RS07995	SAK_1585	-1.519661637	0.130362767	1581422	1581970	-	549	-
1524	SAK_RS07680	SAK_1524	-1.505296991	0.127312727	1521303	1521704	-	402	-
1195	SAK_RS05980	SAK_1195	-1.495883658	0.129734883	1173488	1174282	-	795	potB
512	#N/A	SAK_0512	-1.490199363	0.125726808	454032	455068	-	1037	-
1373	SAK_RS06910	SAK_1373	-1.480486043	0.12947717	1359488	1360336	-	849	-
691	SAK_RS03455	SAK_0691	-1.453113846	0.129734883	625228	626424	+	1197	-
1414	SAK_RS07115	SAK_1414	-1.452580096	0.138762275	1397171	1399315	+	2145	-
2076	SAK_RS10445	SAK_2076	-1.436390507	0.145369724	2071253	2071876	-	624	-
1264	#N/A	SAK_1264	-1.433785776	0.145369724	1235051	1235818	-	768	-
154	SAK_RS00760	SAK_0154	-1.412145687	0.138762275	112295	113137	-	843	-
1105	SAK_RS05525	SAK_1105	-1.410911863	0.132975096	1078201	1079163	-	963	-
993	SAK_RS04970	SAK_0993	-1.407315552	0.132975096	948079	948600	+	522	-
65	SAK_RS00320	SAK_0065	-1.396142377	0.138762275	47331	48635	+	1305	sip
302	SAK_RS01490	SAK_0302	-1.395074447	0.129734883	257554	258189	-	636	-
1001	SAK_RS05010	SAK_1001	-1.39054359	0.169868628	963581	964549	+	969	-
989	SAK_RS04950	SAK_0989	-1.387343564	0.138762275	944268	945539	+	1272	murA
1611	SAK_RS08125	SAK_1611	-1.385300118	0.138762275	1604407	1605147	-	741	xerD
679	SAK_RS03395	SAK_0679	-1.375483102	0.138762275	614037	615020	-	984	-
1661	#N/A	SAK_1661	-1.375376509	0.157720711	1652015	1652128	+	114	-
1367	SAK_RS06880	SAK_1367	-1.336590789	0.155716174	1351616	1352125	+	510	-
83	SAK_RS00410	SAK_0083	-1.335647016	0.145369724	66092	66529	+	438	-
1432	SAK_RS07205	SAK_1432	-1.329171331	0.140920456	1414035	1415369	-	1335	-
1913	SAK_RS09635	SAK_1913	-1.328895211	0.145369724	1916590	1917498	-	909	-
1222	SAK_RS06130	SAK_1222	-1.327673464	0.145369724	1196121	1196318	-	198	-
506	SAK_RS02525	SAK_0506	-1.324230925	0.14117843	451781	452239	-	459	-
1794	SAK_RS09030	SAK_1794	-1.319824945	0.145369724	1787058	1787870	-	813	purR
369	SAK_RS01830	SAK_0369	-1.313874938	0.155716174	316472	317806	+	1335	pepC
1828	SAK_RS09205	SAK_1828	-1.301603797	0.155716174	1823461	1824444	-	984	-
1123	SAK_RS05615	SAK_1123	-1.294141396	0.143058407	1093406	1097815	-	4410	-
772	SAK_RS03850	SAK_0772	-1.28865065	0.147719162	707515	708264	-	750	-
1005	SAK_RS05030	SAK_1005	-1.286361828	0.145369724	969052	970041	+	990	lplA
153	SAK_RS00755	SAK_0153	-1.285290027	0.145369724	111689	112258	+	570	-
2101	SAK_RS10585	SAK_2101	-1.28322615	0.145369724	2093487	2094098	-	612	-
1935	SAK_RS09745	SAK_1935	-1.277596245	0.163741103	1935837	1936145	-	309	-
662	SAK_RS03310	SAK_0662	-1.272689115	0.161487887	592426	593742	-	1317	-
1983	SAK_RS09980	SAK_1983	-1.269780026	0.147719162	1969227	1969994	-	768	cfb
2098	SAK_RS10570	SAK_2098	-1.253585144	0.155716174	2088909	2089361	-	453	rplI
188	SAK_RS00935	SAK_0188	-1.238722344	0.155716174	149900	151240	-	1341	-
1234	SAK_RS06190	SAK_1234	-1.236544174	0.147719162	1206716	1207411	+	696	-
1886	SAK_RS09495	SAK_1886	-1.22914635	0.156913217	1887081	1887977	-	897	-
74	SAK_RS00365	SAK_0074	-1.227833141	0.172669706	55716	56693	+	978	-
1015	SAK_RS05080	SAK_1015	-1.226034318	0.169956616	978499	979269	+	771	-
998	SAK_RS04995	SAK_0998	-1.224611449	0.155716174	959067	959984	+	918	-
1128	SAK_RS05640	SAK_1128	-1.224099198	0.180357604	1102906	1103196	-	291	-
1718	SAK_RS08640	SAK_1718	-1.218348031	0.155716174	1708250	1708921	-	672	-
1233	SAK_RS06185	SAK_1233	-1.209787289	0.17074747	1206343	1206735	+	393	-
1728	SAK_RS08690	SAK_1728	-1.203076224	0.158610161	1717491	1718033	-	543	-
1437	SAK_RS07230	SAK_1437	-1.199255996	0.156818512	1418626	1419225	-	600	-

341	SAK_RS01690	SAK_0341	-1.197657247	0.180357604	284801	285442	+	642	-
1326	SAK_RS06660	SAK_1326	-1.186879183	0.166416628	1309068	1310249	-	1182	-
1612	SAK_RS08130	SAK_1612	-1.184829731	0.172669706	1605137	1605610	-	474	-
437	SAK_RS02170	SAK_0437	-1.182643563	0.163741103	384996	385580	+	585	-
1046	SAK_RS05230	SAK_1046	-1.180964844	0.163741103	1014769	1015689	-	921	coaA
576	SAK_RS02870	SAK_0576	-1.175383376	0.170990433	518281	518526	+	246	-
1145	SAK_RS05725	SAK_1145	-1.173348688	0.17239133	1123189	1124208	-	1020	lplA
1893	SAK_RS09530	SAK_1893	-1.170228205	0.169868628	1893852	1895300	-	1449	-
1651	SAK_RS08320	SAK_1651	-1.169306253	0.174705686	1642475	1643530	+	1056	-
397	SAK_RS01970	SAK_0397	-1.162120599	0.170990433	346258	347241	+	984	-
1660	SAK_RS08365	SAK_1660	-1.156826013	0.17239133	1651415	1651951	-	537	-
1435	SAK_RS07220	SAK_1435	-1.155240109	0.166416628	1417136	1417777	-	642	-
1440	SAK_RS07245	SAK_1440	-1.152859258	0.169868628	1420792	1422300	-	1509	-
553	SAK_RS02750	SAK_0553	-1.151510975	0.17239133	496116	496565	-	450	-
469	SAK_RS02335	SAK_0469	-1.150848116	0.163741103	414492	415232	-	741	-
1184	SAK_RS05925	SAK_1184	-1.150267809	0.184091563	1162110	1162457	+	348	-
590	SAK_RS02940	SAK_0590	-1.143942923	0.17239133	532502	534763	-	2262	clpE
1409	SAK_RS07090	SAK_1409	-1.141324963	0.18748101	1392455	1392790	-	336	-
1496	SAK_RS07530	SAK_1496	-1.138127261	0.169868628	1491473	1492393	-	921	-
609	SAK_RS03035	SAK_0609	-1.137617049	0.169868628	550495	550875	-	381	-
1442	#N/A	SAK_1442	-1.136796835	0.184091563	1425799	1426641	-	843	-
140	SAK_RS00690	SAK_0140	-1.13343611	0.174968901	99890	100309	+	420	-
389	SAK_RS01930	SAK_0389	-1.123348852	0.18958454	338179	340134	+	1956	stk1
2134	SAK_RS10750	SAK_2134	-1.113379892	0.189174312	2124951	2125430	-	480	-
1586	#N/A	SAK_1586	-1.105354361	0.174968901	1582038	1583130	-	1093	serC
1067	SAK_RS05335	SAK_1067	-1.105307905	0.174968901	1037659	1038147	-	489	-
328	SAK_RS01620	SAK_0328	-1.10483702	0.187140104	273285	273863	+	579	-
163	SAK_RS00805	SAK_0163	-1.101632964	0.184091563	121207	122526	+	1320	-
173	SAK_RS00855	SAK_0173	-1.096695269	0.184091563	132770	133456	+	687	-
1752	SAK_RS08810	SAK_1752	-1.0947966	0.174968901	1744576	1745475	-	900	-
703	SAK_RS03515	SAK_0703	-1.091863331	0.188667224	636258	636845	-	588	-
1354	SAK_RS06815	SAK_1354	-1.090156487	0.184091563	1336645	1337640	-	996	fni
379	SAK_RS01880	SAK_0379	-1.089565725	0.189335928	328345	329085	+	741	-
1487	SAK_RS07480	SAK_1487	-1.088966164	0.180357604	1476583	1477779	-	1197	-
1709	SAK_RS08605	SAK_1709	-1.085206833	0.174309267	1699638	1699868	-	231	-
564	SAK_RS02810	SAK_0564	-1.084425328	0.186437602	507187	508677	-	1491	-
339	SAK_RS01680	SAK_0339	-1.082649715	0.196880977	283032	283574	+	543	-
1623	SAK_RS08185	SAK_1623	-1.082491255	0.21112807	1612031	1612963	+	933	-
1441	#N/A	SAK_1441	-1.082009324	0.184091563	1422341	1425622	-	3282	-
1383	SAK_RS06960	SAK_1383	-1.081106469	0.178270806	1369959	1371029	-	1071	-
186	SAK_RS00925	SAK_0186	-1.074681808	0.180357604	145690	149184	-	3495	bag
1903	SAK_RS09580	SAK_1903	-1.073363903	0.184091563	1907128	1908165	+	1038	-
1714	#N/A	SAK_1714	-1.070949133	0.189062541	1701570	1702637	-	1068	pepP
826	SAK_RS04135	SAK_0826	-1.068332409	0.209867819	769867	770484	+	618	eda
1146	SAK_RS05730	SAK_1146	-1.06704191	0.189335928	1124235	1125113	-	879	-
1951	#N/A	SAK_1951	-1.066891121	0.203123056	1947346	1948100	-	755	-
2053	SAK_RS10325	SAK_2053	-1.063074959	0.198989025	2045239	2046654	+	1416	-
2126	SAK_RS10710	SAK_2126	-1.053113469	0.198989025	2117043	2118068	-	1026	trpS
1203	SAK_RS06020	SAK_1203	-1.047111918	0.196880977	1180017	1180904	-	888	rarD
478	SAK_RS02385	SAK_0478	-1.043994059	0.189062541	422216	423259	+	1044	-
582	SAK_RS02900	SAK_0582	-1.043168221	0.186437602	525183	525857	+	675	-
948	SAK_RS04740	SAK_0948	-1.04175284	0.186437602	901887	903140	+	1254	-
1392	SAK_RS07005	SAK_1392	-1.03685998	0.189335928	1376017	1377264	-	1248	-
2017	SAK_RS10150	SAK_2017	-1.036401097	0.202067542	2011446	2012408	-	963	-
1549	SAK_RS07810	SAK_1549	-1.033551836	0.196880977	1545552	1546088	+	537	-
1967	SAK_RS09900	SAK_1967	-1.032438849	0.202774204	1957698	1958552	-	855	-
604	SAK_RS03010	SAK_0604	-1.030342034	0.197739438	547082	547921	+	840	-
1096	SAK_RS05480	SAK_1096	-1.030251319	0.186437602	1067026	1067616	-	591	-
1044	SAK_RS05220	SAK_1044	-1.029882045	0.200328935	1013550	1014383	+	834	-
1011	SAK_RS05060	SAK_1011	-1.028757386	0.186437602	975759	976130	+	372	-
977	SAK_RS04890	SAK_0977	-1.026951523	0.189335928	933565	934704	+	1140	glgC
1748	SAK_RS08790	SAK_1748	-1.024161428	0.189335928	1739087	1740805	-	1719	cydD
1823	SAK_RS09180	SAK_1823	-1.021982338	0.198989025	1817089	1818606	-	1518	-
682	#N/A	SAK_0682	-1.021492125	0.196880977	616918	617694	+	777	-
1904	SAK_RS09585	SAK_1904	-1.014205176	0.193916283	1908165	1908662	+	498	-
1469	SAK_RS07385	SAK_1469	-1.013945972	0.19854204	1451297	1452283	-	987	-
461	SAK_RS02295	SAK_0461	-1.010371101	0.196408987	405695	406309	+	615	-
470	SAK_RS02340	SAK_0470	-1.005653859	0.198989025	415419	416561	+	1143	tgt
353	SAK_RS01750	SAK_0353	-1.005178603	0.197046947	298513	300117	+	1605	-
1366	SAK_RS06875	SAK_1366	-1.00371058	0.198989025	1350099	1351448	-	1350	gdhA
1877	SAK_RS09450	SAK_1877	-1.003321104	0.204619869	1875572	1877041	-	1470	-
183	SAK_RS00905	SAK_0183	-1.002783792	0.198989025	142732	143616	+	885	-
671	SAK_RS03355	SAK_0671	-1.001472151	0.204619869	604247	605695	-	1449	-
1405	SAK_RS07070	SAK_1405	-1.000091083	0.210300662	1387827	1389041	-	1215	thiI
300	SAK_RS01480	SAK_0300	-0.999664559	0.198981738	255974	256624	-	651	-
504	SAK_RS02515	SAK_0504	-0.998394239	0.199504128	450375	450911	+	537	-
354	SAK_RS01755	SAK_0354	-0.995522938	0.210934582	300325	301710	-	1386	-
1235	SAK_RS06195	SAK_1235	-0.994315897	0.203643304	1207480	1208103	+	624	-
1242	SAK_RS06230	SAK_1242	-0.993676405	0.208367958	1211510	1213969	-	2460	parC
1508	SAK_RS07595	SAK_1508	-0.992604573	0.198989025	1504831	1505223	+	393	gloA
1836	SAK_RS09245	SAK_1836	-0.991688972	0.201357963	1830094	1830711	-	618	-
941	SAK_RS04705	SAK_0941	-0.989538239	0.209510112	892696	894147	+	1452	-
958	SAK_RS04795	SAK_0958	-0.983282249	0.216258089	913647	914213	+	567	-
1698	SAK_RS08550	SAK_1698	-0.983191902	0.198989025	1686150	1687157	-	1008	-
320	SAK_RS01580	SAK_0320	-0.98157816	0.21112807	265841	267331	+	1491	-
233	SAK_RS01150	SAK_0233	-0.980792039	0.212001013	200981	201955	+	975	-
1151	SAK_RS05755	SAK_1151	-0.975675075	0.203643304	1128433	1129266	-	834	-
1042	SAK_RS05210	SAK_1042	-0.975574751	0.21112807	1012256	1012897	+	642	-
1223	SAK_RS06135	SAK_1223	-0.974333911	0.210934582	1196365	1196907	-	543	-
1948	SAK_RS09810	SAK_1948	-0.97374758	0.254233211	1944822	1945316	+	495	-
1158	SAK_RS05795	SAK_1158	-0.970834959	0.202774204	1136826	1137428	-	603	-
1313	SAK_RS06585	SAK_1313	-0.970134093	0.221542766	1291572	1292168	+	597	-
2015	SAK_RS10140	SAK_2015	-0.967239187	0.212456086	2009726	2010529	-	804	-

1194	SAK_RS05975	SAK_1194	-0.965471448	0.209510112	1172715	1173491	-	777	potC
1750	SAK_RS08800	SAK_1750	-0.963000806	0.21112807	1741825	1743252	-	1428	cydA
1140	SAK_RS05700	SAK_1140	-0.962293089	0.210538919	1116194	1116532	-	339	-
1700	SAK_RS08560	SAK_1700	-0.959242989	0.212001013	1689920	1690867	-	948	manA
1621	SAK_RS08175	SAK_1621	-0.958516777	0.215204656	1610893	1611636	-	744	-
327	SAK_RS01615	SAK_0327	-0.955141792	0.209510112	272625	273185	+	561	-
1629	SAK_RS08215	SAK_1629	-0.953594313	0.209510112	1618877	1619380	-	504	-
613	SAK_RS03055	SAK_0613	-0.949566937	0.212213114	552166	552501	-	336	-
1168	SAK_RS05845	SAK_1168	-0.948260891	0.21519047	1144913	1146148	+	1236	-
867	SAK_RS04335	SAK_0867	-0.946926844	0.215204656	812037	812948	+	912	-
1861	#N/A	SAK_1861	-0.945730012	0.227732614	1857986	1859126	-	1141	-
1382	SAK_RS06955	SAK_1382	-0.945542347	0.215204656	1369023	1369946	-	924	panE
1188	SAK_RS05945	SAK_1188	-0.944467876	0.21112807	1165263	1166699	-	1437	arb
2070	SAK_RS10415	SAK_2070	-0.941784451	0.255198773	2063651	2066191	-	2541	-
854	SAK_RS04275	SAK_0854	-0.9414358	0.21112807	802540	803352	-	813	-
812	SAK_RS04065	SAK_0812	-0.941047485	0.242071372	754648	755319	+	672	-
1684	SAK_RS08480	SAK_1684	-0.940978962	0.217198349	1670972	1671469	-	498	-
1213	SAK_RS06075	SAK_1213	-0.939186988	0.248542053	1189245	1189931	-	687	-
885	SAK_RS04425	SAK_0885	-0.939077126	0.21112807	829735	832530	+	2796	ppc
329	SAK_RS01625	SAK_0329	-0.938642934	0.214828534	273906	274586	+	681	-
1747	SAK_RS08785	SAK_1747	-0.935501195	0.215204656	1737346	1739094	-	1749	cydC
589	SAK_RS02935	SAK_0589	-0.935135603	0.212001013	531861	532316	-	456	-
404	SAK_RS02005	SAK_0404	-0.927871077	0.239137121	354031	354957	-	927	-
1635	SAK_RS08240	SAK_1635	-0.927729497	0.266994845	1626200	1627102	-	903	dnaI
142	SAK_RS00700	SAK_0142	-0.927687216	0.230277253	101110	101802	+	693	-
1352	SAK_RS06805	SAK_1352	-0.92582636	0.218821478	1335459	1336313	+	855	-
1756	SAK_RS08830	SAK_1756	-0.921056476	0.220157415	1751340	1753325	-	1986	tkt
919	SAK_RS04595	SAK_0919	-0.918974967	0.230277253	870721	871977	+	1257	-
352	SAK_RS01745	SAK_0352	-0.918754947	0.217175681	297775	298509	+	735	-
257	SAK_RS01265	SAK_0257	-0.918159049	0.230277253	220089	222119	+	2031	-
206	SAK_RS01020	SAK_0206	-0.915798258	0.227290649	169047	170702	+	1656	-
1553	SAK_RS07830	SAK_1553	-0.912557755	0.219354656	1551359	1552162	+	804	-
939	SAK_RS04695	SAK_0939	-0.905236451	0.240729169	890955	891713	+	759	-
1336	SAK_RS06725	SAK_1336	-0.905150307	0.227290649	1320422	1322530	+	2109	clpL
1268	SAK_RS06360	SAK_1268	-0.904619043	0.217383842	1238627	1239034	-	408	arsC
1644	SAK_RS08285	SAK_1644	-0.90064557	0.229172068	1634280	1635659	+	1380	-
923	SAK_RS04615	SAK_0923	-0.898464737	0.239137121	874902	875582	+	681	-
2093	SAK_RS10545	SAK_2093	-0.897888466	0.230277253	2083694	2084659	+	966	-
393	SAK_RS01950	SAK_0393	-0.895876626	0.230277253	342682	344082	+	1401	-
715	SAK_RS03575	SAK_0715	-0.892861874	0.237386666	648187	649470	+	1284	aroA
537	SAK_RS02670	SAK_0537	-0.889269215	0.239137121	480563	482044	+	1482	galT
2033	SAK_RS10230	SAK_2033	-0.888006739	0.233731862	2025345	2026604	-	1260	cinA
326	SAK_RS01610	SAK_0326	-0.885524489	0.247354993	271834	272052	+	219	-
1353	SAK_RS06810	SAK_1353	-0.884730477	0.244276007	1336359	1336577	-	219	-
1365	SAK_RS06870	SAK_1365	-0.884557017	0.230277253	1349619	1350029	-	411	def
1771	SAK_RS08915	SAK_1771	-0.882332999	0.23112401	1764952	1766142	+	1191	cfa
1842	SAK_RS09275	SAK_1842	-0.88144307	0.244627648	1837633	1838451	+	819	-
1157	SAK_RS05790	SAK_1157	-0.881052515	0.231453519	1135096	1136814	-	1719	-
940	SAK_RS04700	SAK_0940	-0.880600276	0.227732614	891798	892361	-	564	-
1749	SAK_RS08795	SAK_1749	-0.871249202	0.242022895	1740805	1741824	-	1020	cydB
2007	SAK_RS10095	SAK_2007	-0.870412499	0.242071899	2000452	2002092	-	1641	-
1535	SAK_RS07735	SAK_1535	-0.870225923	0.263815242	1530741	1531595	-	855	-
1280	SAK_RS06420	SAK_1280	-0.86662199	0.266164941	1252098	1253324	-	1227	-
1029	SAK_RS05155	SAK_1029	-0.866256576	0.243200988	999057	999695	-	639	-
1193	SAK_RS05970	SAK_1193	-0.860825259	0.249943584	1171649	1172722	-	1074	potD
1768	SAK_RS08905	SAK_1768	-0.859684222	0.266063731	1763152	1763562	-	411	-
355	SAK_RS01760	SAK_0355	-0.857944162	0.242286709	301851	302654	+	804	proB
1784	SAK_RS08980	SAK_1784	-0.851754357	0.24114476	1776787	1777296	-	510	-
1733	SAK_RS08715	SAK_1733	-0.851237382	0.249909389	1722624	1723106	+	483	-
1219	#N/A	SAK_1219	-0.851155293	0.255198773	1193976	1194738	-	763	-
560	SAK_RS02790	SAK_0560	-0.850260699	0.244627648	502296	502811	+	516	-
387	SAK_RS01920	SAK_0387	-0.846099177	0.242504335	336082	337404	+	1323	sun
1178	SAK_RS05895	SAK_1178	-0.845726209	0.257690003	1156776	1157660	-	885	-
1763	#N/A	SAK_1763	-0.840726104	0.264006972	1759082	1759565	-	484	-
460	SAK_RS02290	SAK_0460	-0.839169584	0.244627648	405379	405585	+	207	-
786	SAK_RS03920	SAK_0786	-0.834667929	0.298535565	723252	724403	+	1152	-
79	SAK_RS00390	SAK_0079	-0.825737631	0.253003725	60969	62360	+	1392	-
927	SAK_RS04635	SAK_0927	-0.82369308	0.258110751	878350	879501	+	1152	-
1699	SAK_RS08555	SAK_1699	-0.823669798	0.263815242	1687283	1689811	-	2529	secA
929	SAK_RS04645	SAK_0929	-0.823668576	0.258110751	880516	882321	+	1806	pepB
1646	SAK_RS08295	SAK_1646	-0.822846717	0.263815942	1636378	1637208	-	831	-
518	#N/A	SAK_0518	-0.820077185	0.278581315	462673	463375	+	703	-
1846	SAK_RS09295	SAK_1846	-0.819664164	0.255364611	1841801	1842442	+	642	-
1103	SAK_RS05515	SAK_1103	-0.817712172	0.261634673	1076472	1077233	-	762	-
647	SAK_RS03240	SAK_0647	-0.817022343	0.266063731	576381	580502	+	4122	-
1012	SAK_RS05065	SAK_1012	-0.816315293	0.266063731	976155	976535	+	381	-
805	SAK_RS04015	SAK_0805	-0.815315688	0.292945589	743195	746419	+	3225	-
1557	SAK_RS07850	SAK_1557	-0.814813085	0.263815242	1555029	1555676	+	648	-
1080	SAK_RS05400	SAK_1080	-0.814350899	0.271605064	1051800	1052480	-	681	ciaR
1821	SAK_RS09170	SAK_1821	-0.81324603	0.258110751	1814563	1816170	-	1608	-
597	SAK_RS02975	SAK_0597	-0.810494293	0.260062546	540785	542125	+	1341	xseA
1789	SAK_RS09005	SAK_1789	-0.808653108	0.274842775	1781408	1782277	-	870	-
1928	SAK_RS09710	SAK_1928	-0.803547385	0.266063731	1931360	1931920	-	561	-
1138	SAK_RS05690	SAK_1138	-0.803537277	0.266063731	1113250	1114470	-	1221	-
904	SAK_RS04520	SAK_0904	-0.798574004	0.279696141	853493	854257	-	765	-
1847	SAK_RS09300	SAK_1847	-0.798050839	0.279696141	1842456	1842947	+	492	-
1655	SAK_RS08340	SAK_1655	-0.797900471	0.283096027	1646841	1647665	-	825	-
1624	SAK_RS08190	SAK_1624	-0.794870942	0.266063731	1613163	1613828	-	666	-
1739	SAK_RS08745	SAK_1739	-0.793880519	0.266063731	1729021	1729980	+	960	-
599	SAK_RS02985	SAK_0599	-0.792829788	0.278581315	542318	543190	+	873	-
673	SAK_RS03365	SAK_0673	-0.792128065	0.282108519	606547	607245	-	699	-
1317	#N/A	SAK_1317	-0.790163413	0.293571626	1293992	1295098	+	1107	-
1478	SAK_RS07430	SAK_1478	-0.789639724	0.279696141	1463653	1465125	+	1473	-

1931	#N/A	SAK_1931	-0.787463467	0.283723286	1933607	1934065	-	459	-
1745	SAK_RS08775	SAK_1745	-0.78559312	0.280903297	1735876	1736235	+	360	-
1613	SAK_RS08135	SAK_1613	-0.780467171	0.300124568	1605607	1606128	-	522	-
1081	SAK_RS05405	SAK_1081	-0.779959802	0.279696141	1052642	1055191	-	2550	pepN
1991	SAK_RS10020	SAK_1991	-0.779901745	0.294807681	1979631	1984346	+	4716	cspA
1479	SAK_RS07435	SAK_1479	-0.777624506	0.296193317	1465170	1465634	-	465	-
1908	SAK_RS09610	SAK_1908	-0.777338429	0.280903297	1912497	1913324	-	828	-
150	SAK_RS00740	SAK_0150	-0.776607578	0.296680325	109721	110497	+	777	truA
1020	SAK_RS05105	SAK_1020	-0.776080481	0.291985895	985611	986276	+	666	-
2128	SAK_RS10720	SAK_2128	-0.77530512	0.278501194	2119225	2119983	+	759	-
375	SAK_RS01860	SAK_0375	-0.774239612	0.282428037	323367	324830	+	1464	-
1528	SAK_RS07700	SAK_1528	-0.771606472	0.283723286	1524153	1524959	-	807	-
1116	SAK_RS05580	SAK_1116	-0.769853315	0.312810888	1088829	1089479	-	651	-
1891	SAK_RS09520	SAK_1891	-0.769453109	0.280116719	1891163	1893316	-	2154	-
1729	SAK_RS08695	SAK_1729	-0.768336841	0.280903297	1718036	1718347	-	312	-
1147	SAK_RS05735	SAK_1147	-0.76579255	0.281628814	1125082	1125900	-	819	-
378	SAK_RS01875	SAK_0378	-0.761649492	0.325649365	327462	328358	+	897	-
1115	SAK_RS05575	SAK_1115	-0.755394176	0.289155078	1088525	1088818	-	294	-
1843	SAK_RS09280	SAK_1843	-0.753606976	0.300124568	1838448	1839704	+	1257	-
382	SAK_RS01895	SAK_0382	-0.751074466	0.296680325	330809	331513	+	705	-
724	SAK_RS03620	SAK_0724	-0.750904169	0.314120856	663365	663934	+	570	-
1596	SAK_RS08050	SAK_1596	-0.746167708	0.309186324	1590704	1591657	-	954	livM
1079	SAK_RS05395	SAK_1079	-0.742754162	0.302376522	1050503	1051816	-	1314	ciaH
1309	SAK_RS06565	SAK_1309	-0.736866454	0.306331783	1288166	1288933	+	768	-
1628	SAK_RS08210	SAK_1628	-0.733796386	0.337156514	1616988	1618790	-	1803	-
468	SAK_RS02330	SAK_0468	-0.733746373	0.307124171	413441	414478	+	1038	-
1331	SAK_RS06700	SAK_1331	-0.7329157	0.302376522	1313445	1314650	-	1206	-
771	SAK_RS03845	SAK_0771	-0.732060269	0.312810888	707062	707394	+	333	-
569	SAK_RS02835	SAK_0569	-0.731301673	0.329037947	512072	513301	+	1230	-
1192	SAK_RS05965	SAK_1192	-0.727292985	0.314120856	1170008	1171540	-	1533	-
1104	SAK_RS05520	SAK_1104	-0.726441149	0.314120856	1077230	1078204	-	975	-
882	SAK_RS04410	SAK_0882	-0.726269797	0.311620626	826771	827295	+	525	-
1972	SAK_RS09925	SAK_1972	-0.724902685	0.307885744	1961014	1961349	-	336	-
2001	SAK_RS10070	SAK_2001	-0.721126878	0.309186324	1995471	1995860	+	390	-
1370	SAK_RS06895	SAK_1370	-0.719966195	0.314120856	1355746	1356219	-	474	-
855	SAK_RS04280	SAK_0855	-0.719387825	0.310271221	803352	804254	-	903	-
1406	SAK_RS07075	SAK_1406	-0.71822779	0.332109337	1389043	1390188	-	1146	-
967	SAK_RS04840	SAK_0967	-0.715351071	0.316713977	921200	921772	+	573	-
1438	SAK_RS07235	SAK_1438	-0.712344399	0.318368259	1419218	1419889	-	672	-
467	SAK_RS02325	SAK_0467	-0.711345077	0.322082233	412753	413439	+	687	-
1150	SAK_RS05750	SAK_1150	-0.711272108	0.318368259	1127237	1128436	-	1200	-
1226	SAK_RS06150	SAK_1226	-0.710271786	0.324383904	1197763	1198011	-	249	-
674	SAK_RS03370	SAK_0674	-0.705722931	0.334457577	607413	608177	+	765	-
356	SAK_RS01765	SAK_0356	-0.700604602	0.319858607	302664	303917	+	1254	proA
1546	SAK_RS07790	SAK_1546	-0.700382778	0.333925851	1541300	1542682	-	1383	-
775	SAK_RS03865	SAK_0775	-0.700290374	0.319858607	709858	711066	-	1209	-
1572	SAK_RS07930	SAK_1572	-0.699587642	0.333925851	1567338	1568771	-	1434	-
1061	SAK_RS05305	SAK_1061	-0.69863221	0.320325033	1031449	1031838	+	390	-
8	SAK_RS00035	SAK_0008	-0.695579084	0.322082233	6244	9741	+	3498	mfd
1980	SAK_RS09965	SAK_1980	-0.694161135	0.333925851	1966867	1967427	+	561	-
1095	SAK_RS05475	SAK_1095	-0.693132957	0.333925851	1065721	1066992	-	1272	-
1433	SAK_RS07210	SAK_1433	-0.689800949	0.338891996	1415549	1416115	-	567	-
1529	SAK_RS07705	SAK_1529	-0.688289198	0.348691911	1525281	1525757	-	477	-
80	SAK_RS00395	SAK_0080	-0.68822025	0.337156514	62382	63680	+	1299	purB
602	SAK_RS03000	SAK_0602	-0.68450704	0.357672905	544482	546140	+	1659	recN
1160	SAK_RS05805	SAK_1160	-0.684006505	0.338371963	1138412	1139668	-	1257	glyA
1303	SAK_RS06535	SAK_1303	-0.683256693	0.331194488	1280746	1281480	-	735	-
920	SAK_RS04600	SAK_0920	-0.683205712	0.347745702	872078	873142	+	1065	-
1968	#N/A	SAK_1968	-0.679506048	0.337156514	1958731	1959282	-	552	-
1208	SAK_RS06045	SAK_1208	-0.677703241	0.333925851	1184888	1185277	-	390	-
1064	SAK_RS05320	SAK_1064	-0.675272067	0.338891996	1034491	1035825	+	1335	gid
2120	SAK_RS10680	SAK_2120	-0.673635147	0.361401132	2110786	2111490	+	705	-
495	SAK_RS02470	SAK_0495	-0.67103416	0.350222564	436296	437060	+	765	-
516	SAK_RS02565	SAK_0516	-0.670855655	0.341207848	457819	459012	-	1194	-
717	SAK_RS03585	SAK_0717	-0.670698671	0.337371643	650032	651405	+	1374	-
2062	SAK_RS10375	SAK_2062	-0.670361186	0.355988703	2053828	2055192	+	1365	-
190	SAK_RS00945	SAK_0190	-0.670061689	0.337865636	152094	152483	-	390	-
768	SAK_RS03825	SAK_0768	-0.668210589	0.359463977	704649	705386	+	738	aphA
541	SAK_RS02690	SAK_0541	-0.663957788	0.341207848	484457	484846	+	390	-
401	SAK_RS01990	SAK_0401	-0.662659197	0.35618514	349589	352045	+	2457	-
2060	#N/A	SAK_2060	-0.661923631	0.359463977	2052557	2053087	+	531	-
1804	SAK_RS09080	SAK_1804	-0.66185982	0.34235069	1795257	1796039	-	783	-
1463	SAK_RS07355	SAK_1463	-0.660697693	0.348691911	1446539	1446916	+	378	mscL
933	SAK_RS04665	SAK_0933	-0.660684845	0.348691911	885155	885640	+	486	-
189	SAK_RS00940	SAK_0189	-0.660354347	0.353715599	151237	151890	-	654	-
723	SAK_RS03615	SAK_0723	-0.659662279	0.349408151	661088	662956	+	1869	-
505	SAK_RS02520	SAK_0505	-0.658203008	0.347724174	450887	451720	+	834	-
857	SAK_RS04290	SAK_0857	-0.654696797	0.357672905	804521	805564	+	1044	-
1888	SAK_RS09505	SAK_1888	-0.649058133	0.361849546	1889010	1889942	-	933	lacC
1477	SAK_RS07425	SAK_1477	-0.64890957	0.359463977	1462586	1463422	+	837	-
1864	SAK_RS09385	SAK_1864	-0.648173087	0.363265583	1860646	1861647	-	1002	-
1230	SAK_RS06170	SAK_1230	-0.645166309	0.369303033	1201619	1202944	-	1326	uraA
1503	SAK_RS07570	SAK_1503	-0.644789007	0.36260743	1499232	1499810	-	579	-
299	SAK_RS01475	SAK_0299	-0.642744321	0.355988703	254731	255912	+	1182	-
1547	SAK_RS07795	SAK_1547	-0.642539881	0.359463977	1542690	1543904	-	1215	-
502	SAK_RS02505	SAK_0502	-0.640575723	0.359836159	446733	449900	+	3168	-
1767	SAK_RS08900	SAK_1767	-0.640112622	0.36260743	1762351	1763142	-	792	-
273	SAK_RS01345	SAK_0273	-0.638621021	0.359871121	238007	238525	+	519	-
2112	SAK_RS10640	SAK_2112	-0.637267996	0.377045864	2103146	2104390	-	1245	-
1737	SAK_RS08735	SAK_1737	-0.63720527	0.360887437	1727278	1728207	-	930	-
1969	SAK_RS09910	SAK_1969	-0.634815025	0.359463977	1959323	1959712	-	390	-
158	SAK_RS00780	SAK_0158	-0.634525409	0.362173577	117327	118253	+	927	-
1614	SAK_RS08140	SAK_1614	-0.633177489	0.359836159	1606110	1607084	-	975	rdgB

584	SAK_RS02910	SAK_0584	-0.632272785	0.392230658	526477	526731	+	255	-
823	SAK_RS04120	SAK_0823	-0.630518862	0.363265583	766209	767234	+	1026	-
1109	SAK_RS05545	SAK_1109	-0.62609849	0.38710259	1081846	1082418	-	573	-
2043	SAK_RS10275	SAK_2043	-0.625036454	0.370243472	2036653	2036961	+	309	-
1139	SAK_RS05695	SAK_1139	-0.624582176	0.36260743	1114522	1116063	-	1542	-
653	SAK_RS03270	SAK_0653	-0.62368496	0.374613962	583718	585049	+	1332	-
2039	SAK_RS10255	SAK_2039	-0.622573389	0.363265583	2031322	2031525	+	204	-
175	SAK_RS00865	SAK_0175	-0.621695071	0.369303033	134151	135221	+	1071	-
1885	SAK_RS09490	SAK_1885	-0.620111081	0.369303033	1886135	1886986	-	852	-
820	SAK_RS04105	SAK_0820	-0.619788369	0.389673739	762322	763380	+	1059	-
220	SAK_RS01090	SAK_0220	-0.616222284	0.389673739	183414	184445	-	1032	-
1550	SAK_RS07815	SAK_1550	-0.615147105	0.373480294	1546064	1546897	+	834	-
907	SAK_RS04535	SAK_0907	-0.612002675	0.381308016	855869	858106	+	2238	-
1468	SAK_RS07380	SAK_1468	-0.611669171	0.374613962	1450133	1451260	+	1128	-
769	SAK_RS03830	SAK_0769	-0.611283531	0.374364869	705707	706225	+	519	-
1949	SAK_RS09815	SAK_1949	-0.610752176	0.383912208	1945603	1946553	-	951	-
1118	SAK_RS05590	SAK_1118	-0.606816742	0.380758797	1090513	1090827	-	315	-
1985	SAK_RS09990	SAK_1985	-0.603617691	0.389673739	1971503	1972588	+	1086	-
1962	SAK_RS09875	SAK_1962	-0.602425957	0.389503316	1953613	1953921	+	309	-
895	SAK_RS04475	SAK_0895	-0.602217441	0.386708435	844282	844968	+	687	-
1610	SAK_RS08120	SAK_1610	-0.601491542	0.383424343	1603700	1604407	-	708	scpA
1143	SAK_RS05715	SAK_1143	-0.60042731	0.437406181	1119716	1121191	-	1476	-
144	SAK_RS00710	SAK_0144	-0.600397352	0.383424343	102548	103123	+	576	-
2016	SAK_RS10145	SAK_2016	-0.599408083	0.389673739	2010534	2011430	-	897	-
1870	SAK_RS09415	SAK_1870	-0.599079494	0.393182791	1868987	1869571	-	585	-
1349	SAK_RS06790	SAK_1349	-0.597160448	0.397705743	1333259	1333708	+	450	-
139	SAK_RS00685	SAK_0139	-0.594337662	0.393182791	99119	99670	+	552	-
444	SAK_RS02205	SAK_0444	-0.591693241	0.396827784	390584	391003	-	420	-
672	SAK_RS03360	SAK_0672	-0.5907607	0.397050486	605840	606550	-	711	-
1564	SAK_RS07885	SAK_1564	-0.590138611	0.390415625	1559774	1560730	-	957	-
1241	SAK_RS06225	SAK_1241	-0.589909957	0.389673739	1210375	1211397	-	1023	ilvE
143	SAK_RS00705	SAK_0143	-0.589465028	0.386719411	101799	102551	+	753	-
1647	SAK_RS08300	SAK_1647	-0.589160674	0.389673739	1637201	1638877	-	1677	-
1302	SAK_RS06530	SAK_1302	-0.587503882	0.389673739	1276819	1280577	-	3759	-
1666	SAK_RS08390	SAK_1666	-0.587115484	0.389894774	1655092	1655496	-	405	-
1702	SAK_RS08570	SAK_1702	-0.585428079	0.393182791	1691934	1693853	-	1920	-
439	SAK_RS02180	SAK_0439	-0.584893825	0.390415625	386573	387805	+	1233	-
1389	SAK_RS06990	SAK_1389	-0.584399105	0.389673739	1373721	1375232	-	1512	-
376	SAK_RS01865	SAK_0376	-0.581750894	0.391880121	325055	325537	-	483	luxS
76	SAK_RS00375	SAK_0076	-0.581552861	0.393261066	57802	59064	+	1263	purD
1484	SAK_RS07465	SAK_1484	-0.580954714	0.392230658	1472120	1473604	-	1485	asp2
1266	SAK_RS06350	SAK_1266	-0.580681628	0.393261066	1236521	1237777	-	1257	-
1075	SAK_RS05375	SAK_1075	-0.580513209	0.392230658	1046983	1047756	-	774	-
1368	SAK_RS06885	SAK_1368	-0.579611443	0.393046772	1352166	1353935	-	1770	-
1519	SAK_RS07645	SAK_1519	-0.578573152	0.403729819	1515703	1516524	-	822	mutM
464	SAK_RS02310	SAK_0464	-0.577672507	0.402048856	409921	410361	+	441	-
1551	SAK_RS07820	SAK_1551	-0.574046929	0.397322395	1546946	1548661	-	1716	pabB
1606	SAK_RS08100	SAK_1606	-0.568159074	0.397322395	1600693	1602132	+	1440	-
1351	SAK_RS06800	SAK_1351	-0.567833994	0.397322395	1334444	1335358	+	915	-
666	SAK_RS03330	SAK_0666	-0.566554521	0.407384536	598289	600220	+	1932	fbp
1166	SAK_RS05835	SAK_1166	-0.564757289	0.40553778	1143322	1144269	+	948	-
852	SAK_RS04265	SAK_0852	-0.563971454	0.403729819	800069	800893	+	825	-
238	SAK_RS01170	SAK_0238	-0.563075512	0.404184446	204299	204964	+	666	-
184	SAK_RS00915	SAK_0184	-0.562822619	0.404184446	144017	145039	+	1023	-
540	SAK_RS02685	SAK_0540	-0.560818283	0.402048856	484041	484424	+	384	-
1070	SAK_RS05350	SAK_1070	-0.560067074	0.399828584	1040055	1042010	+	1956	-
2059	SAK_RS10360	SAK_2059	-0.557643169	0.401016245	2050792	2052057	+	1266	-
395	SAK_RS01960	SAK_0395	-0.556280724	0.410665403	344487	345263	-	777	-
2000	SAK_RS10065	SAK_2000	-0.553401009	0.409530285	1995055	1995438	+	384	-
1381	SAK_RS06950	SAK_1381	-0.55261246	0.406829589	1367177	1368811	-	1635	-
325	SAK_RS01605	SAK_0325	-0.551466546	0.407384536	271250	271831	+	582	-
66	SAK_RS00325	SAK_0066	-0.550513087	0.42275996	48882	49580	+	699	-
1548	SAK_RS07800	SAK_1548	-0.548546121	0.416134775	1544162	1545067	+	906	-
284	SAK_RS01400	SAK_0284	-0.545655962	0.409530285	245277	246152	+	876	-
630	#N/A	SAK_0630	-0.545493232	0.412963943	563613	563987	+	375	-
252	SAK_RS01240	SAK_0252	-0.544952303	0.416126638	214845	216473	+	1629	-
513	SAK_RS02550	SAK_0513	-0.544749143	0.418555119	455178	456020	-	843	-
716	SAK_RS03580	SAK_0716	-0.543790983	0.412963943	649463	649975	+	513	aroK
949	SAK_RS04745	SAK_0949	-0.543212372	0.428155813	903173	904255	-	1083	-
274	SAK_RS01350	SAK_0274	-0.54169843	0.419985019	238618	239478	+	861	-
191	SAK_RS00950	SAK_0191	-0.540019138	0.418901076	152516	152899	-	384	-
725	SAK_RS03625	SAK_0725	-0.539782899	0.4426257	663973	665928	-	1956	-
1970	SAK_RS09915	SAK_1970	-0.539437224	0.419109253	1959745	1960128	-	384	-
1780	SAK_RS08960	SAK_1780	-0.53469351	0.416126638	1773382	1773789	-	408	rimI
438	SAK_RS02175	SAK_0438	-0.529277039	0.429366831	385670	386281	+	612	-
1504	SAK_RS07575	SAK_1504	-0.5274548	0.419985019	1499920	1501962	-	2043	-
1255	SAK_RS06295	SAK_1255	-0.52737341	0.437268869	1226719	1227858	-	1140	cpsH
1060	SAK_RS05300	SAK_1060	-0.526059142	0.428215382	1031033	1031416	+	384	-
1587	SAK_RS08005	SAK_1587	-0.523932544	0.42491063	1583263	1583898	+	636	-
1618	SAK_RS08160	SAK_1618	-0.523593802	0.434509782	1609051	1609572	-	522	-
974	SAK_RS04875	SAK_0974	-0.521221017	0.453339242	928126	929145	+	1020	-
2040	SAK_RS10260	SAK_2040	-0.518728843	0.437406181	2031582	2034158	-	2577	hexA
746	SAK_RS03725	SAK_0746	-0.518246189	0.428155813	682200	683489	+	1290	-
1295	SAK_RS06495	SAK_1295	-0.513044123	0.437406181	1270846	1271607	-	762	-
685	SAK_RS03425	SAK_0685	-0.509967613	0.437406181	620156	621676	+	1521	adcA
1133	SAK_RS05665	SAK_1133	-0.50988279	0.437406181	1108212	1109288	-	1077	carA
1806	SAK_RS09090	SAK_1806	-0.508384342	0.444472343	1796512	1796904	-	393	-
1289	SAK_RS06465	SAK_1289	-0.507607277	0.437406181	1262931	1263719	-	789	-
1332	SAK_RS06705	SAK_1332	-0.507030146	0.443650852	1314702	1315193	-	492	-
164	SAK_RS00810	SAK_0164	-0.50621638	0.437406181	122833	123999	-	1167	-
975	SAK_RS04880	SAK_0975	-0.504013449	0.438924756	929149	931449	+	2301	pulA
1058	SAK_RS05290	SAK_1058	-0.503350144	0.437406181	1028621	1029583	+	963	-
1668	SAK_RS08400	SAK_1668	-0.502470628	0.440310941	1656441	1657550	-	1110	-

1883	SAK_RS09480	SAK_1883	-0.501454142	0.437406181	1883165	1884772	-	1608	dexB
1755	SAK_RS08825	SAK_1755	-0.501419557	0.437406181	1749430	1751202	-	1773	-
865	SAK_RS04325	SAK_0865	-0.501321193	0.45236022	810935	811366	+	432	-
1272	SAK_RS06380	SAK_1272	-0.50127168	0.441295796	1242062	1242850	-	789	estA
1394	SAK_RS07015	SAK_1394	-0.499770544	0.444703166	1377992	1379236	-	1245	-
1209	SAK_RS06050	SAK_1209	-0.498814544	0.444703166	1185310	1185693	-	384	-
707	SAK_RS03535	SAK_0707	-0.496686398	0.450634609	639241	639801	+	561	-
1592	SAK_RS08030	SAK_1592	-0.491572243	0.450520948	1587120	1588286	+	1167	-
868	SAK_RS04340	SAK_0868	-0.491127979	0.444703166	813078	814364	+	1287	-
405	SAK_RS02010	SAK_0405	-0.490976031	0.452216367	355048	355692	-	645	-
915	SAK_RS04575	SAK_0915	-0.490197193	0.457529636	864912	866780	+	1869	bglF
1901	SAK_RS09570	SAK_1901	-0.489444292	0.453116083	1904103	1906505	+	2403	cpdB
2071	SAK_RS10420	SAK_2071	-0.48882584	0.453435595	2066175	2066918	-	744	-
1333	SAK_RS06710	SAK_1333	-0.487624677	0.450520948	1315207	1318818	-	3612	-
1486	SAK_RS07475	SAK_1486	-0.485654589	0.458649575	1475230	1476459	-	1230	secY
574	SAK_RS02860	SAK_0574	-0.4851891	0.463008407	515783	516163	+	381	-
1393	SAK_RS07010	SAK_1393	-0.482800246	0.45733362	1377279	1377989	-	711	-
804	SAK_RS04010	SAK_0804	-0.482281831	0.450634609	740435	743092	+	2658	-
1480	SAK_RS07445	SAK_1480	-0.479641794	0.463008407	1465917	1467242	-	1326	-
1907	SAK_RS09605	SAK_1907	-0.47823809	0.45733362	1910710	1912359	-	1650	-
970	SAK_RS04855	SAK_0970	-0.475630722	0.458649575	923933	924853	+	921	-
264	SAK_RS01300	SAK_0264	-0.475370506	0.45733362	229768	231027	+	1260	-
1807	SAK_RS09095	SAK_1807	-0.471464986	0.461785074	1796895	1798187	-	1293	-
1027	SAK_RS05145	SAK_1027	-0.47079102	0.466148493	995041	997833	+	2793	-
931	SAK_RS04655	SAK_0931	-0.467370715	0.466148493	883252	883923	+	672	-
1293	SAK_RS06485	SAK_1293	-0.465782007	0.460483732	1265935	1268499	-	2565	-
1545	SAK_RS07785	SAK_1545	-0.465519568	0.463008407	1539800	1540966	+	1167	-
462	SAK_RS02300	SAK_0462	-0.465396936	0.469526376	406324	407136	+	813	-
442	SAK_RS02195	SAK_0442	-0.464733635	0.463008407	388920	390227	+	1308	-
1565	#N/A	SAK_1565	-0.461982394	0.475217088	1560727	1561178	-	452	-
739	SAK_RS03685	SAK_0739	-0.461804711	0.468042129	677023	678276	+	1254	-
605	SAK_RS03015	SAK_0605	-0.459978642	0.480237822	547896	548498	+	603	-
832	SAK_RS04165	SAK_0832	-0.459150723	0.471980658	776757	777842	-	1086	pepQ
884	SAK_RS04420	SAK_0884	-0.457649032	0.469042005	827727	829526	-	1800	pepF
2130	SAK_RS10730	SAK_2130	-0.456955861	0.468042129	2121791	2124370	+	2580	-
1171	SAK_RS05860	SAK_1171	-0.455886711	0.472597599	1148404	1148985	-	582	xpt
1695	SAK_RS08535	SAK_1695	-0.455754547	0.468042129	1683038	1684576	-	1539	-
567	#N/A	SAK_0567	-0.455502949	0.485252851	510460	510954	+	495	-
1017	SAK_RS05090	SAK_1017	-0.452664652	0.468042129	980303	984415	+	4113	-
258	SAK_RS01270	SAK_0258	-0.450690381	0.490942785	222341	223966	+	1626	-
1641	SAK_RS08270	SAK_1641	-0.447881305	0.471521343	1631959	1632849	-	891	htpX
2005	SAK_RS10085	SAK_2005	-0.44660197	0.476356331	1997178	1999499	-	2322	pbp2A
813	SAK_RS04070	SAK_0813	-0.446195835	0.480237822	755376	756794	-	1419	-
1288	SAK_RS06460	SAK_1288	-0.44289733	0.476488284	1261819	1262922	-	1104	-
1602	SAK_RS08080	SAK_1602	-0.437776831	0.486830579	1595755	1596924	-	1170	-
1712	#N/A	SAK_1712	-0.435544956	0.486583435	1700640	1700804	-	165	-
254	SAK_RS01250	SAK_0254	-0.434997999	0.489124242	217560	218381	+	822	-
2116	SAK_RS10660	SAK_2116	-0.433505977	0.487863691	2107028	2107690	-	663	-
385	SAK_RS01910	SAK_0385	-0.432659805	0.497852608	332720	335110	+	2391	priA
1341	SAK_RS06750	SAK_1341	-0.431647401	0.489124242	1326482	1327111	+	630	-
766	SAK_RS03815	SAK_0766	-0.428373444	0.489124242	702564	704069	+	1506	-
1772	SAK_RS08920	SAK_1772	-0.428031702	0.487863691	1766212	1766937	-	726	-
1472	SAK_RS07400	SAK_1472	-0.424582132	0.493924087	1454694	1456958	-	2265	glgP
819	SAK_RS04100	SAK_0819	-0.422827072	0.497962232	761245	762129	-	885	-
449	SAK_RS02235	SAK_0449	-0.421932723	0.524571353	394760	395395	+	636	trmB
1149	SAK_RS05745	SAK_1149	-0.421751771	0.493966213	1126254	1127240	-	987	-
995	SAK_RS04980	SAK_0995	-0.418922229	0.499498032	951128	952030	-	903	-
370	SAK_RS01835	SAK_0370	-0.41820611	0.497962232	317852	320098	-	2247	-
680	SAK_RS03400	SAK_0680	-0.417072075	0.497962232	615266	615679	-	414	-
1782	SAK_RS08970	SAK_1782	-0.416077419	0.5009368	1774662	1774892	+	231	-
1422	SAK_RS07155	SAK_1422	-0.415187668	0.497962232	1402873	1404093	-	1221	pepT
1897	SAK_RS09550	SAK_1897	-0.414061411	0.497880737	1897387	1899969	-	2583	-
1443	SAK_RS07255	SAK_1443	-0.41360975	0.497962232	1426660	1427229	-	570	lepB
1475	SAK_RS07415	SAK_1475	-0.412523316	0.497962232	1459871	1461118	+	1248	-
458	SAK_RS02280	SAK_0458	-0.412228291	0.497962232	402675	403091	+	417	copY
936	SAK_RS04680	SAK_0936	-0.411867996	0.497962232	889160	889978	+	819	-
1686	SAK_RS08490	SAK_1686	-0.411519678	0.49758321	1672802	1673350	-	549	-
1515	SAK_RS07625	SAK_1515	-0.409951044	0.497962232	1511293	1512462	-	1170	-
1217	SAK_RS06100	SAK_1217	-0.408765481	0.497962232	1192750	1193244	-	495	tpx
1793	SAK_RS09025	SAK_1793	-0.404801998	0.532636863	1786426	1786839	-	414	rpsL
149	SAK_RS00735	SAK_0149	-0.403570493	0.499584173	108403	109650	+	1248	-
2023	SAK_RS10180	SAK_2023	-0.402972969	0.499205114	2017037	2017969	-	933	-
1021	SAK_RS05115	SAK_1021	-0.402621577	0.501545822	987960	988376	+	417	ndk
573	SAK_RS02855	SAK_0573	-0.399885648	0.503657759	514803	515771	+	969	-
1088	SAK_RS05440	SAK_1088	-0.398904726	0.501545822	1060494	1061804	-	1311	-
1787	SAK_RS08995	SAK_1787	-0.398432083	0.502682512	1779275	1779814	-	540	-
1820	SAK_RS09165	SAK_1820	-0.397947719	0.506137551	1813155	1814246	-	1092	-
2006	SAK_RS10090	SAK_2006	-0.396454375	0.501778249	1999543	2000427	+	885	-
1218	SAK_RS06105	SAK_1218	-0.394174809	0.509985189	1193317	1193976	-	660	-
1936	SAK_RS09750	SAK_1936	-0.391194235	0.509402173	1936188	1936658	-	471	-
2061	SAK_RS10370	SAK_2061	-0.390348572	0.506587663	2053173	2053844	+	672	-
1560	SAK_RS07865	SAK_1560	-0.386893333	0.513600141	1556684	1557238	-	555	-
2135	SAK_RS10755	SAK_2135	-0.385574149	0.513600141	2125631	2126860	+	1230	htrA
1625	SAK_RS08195	SAK_1625	-0.382202099	0.514353126	1613852	1614709	-	858	-
1310	SAK_RS06570	SAK_1310	-0.379220511	0.516188383	1288992	1290332	-	1341	-
1532	SAK_RS07720	SAK_1532	-0.378493111	0.515435879	1527953	1529725	-	1773	-
678	SAK_RS03390	SAK_0678	-0.377438514	0.518575552	613483	614040	-	558	-
1575	SAK_RS07945	SAK_1575	-0.376166232	0.519259366	1570565	1571902	-	1338	brnQ
965	SAK_RS04830	SAK_0965	-0.375290759	0.519259366	919059	919730	+	672	thiE
1862	SAK_RS09375	SAK_1862	-0.375281463	0.519259366	1859250	1859894	+	645	-
1259	SAK_RS06315	SAK_1259	-0.374976252	0.518449108	1230310	1231008	-	699	cpsD
821	SAK_RS04110	SAK_0821	-0.371172559	0.520767451	763394	764386	+	993	ldhA
1000	SAK_RS05005	SAK_1000	-0.370180249	0.520859367	961521	963431	+	1911	-

294	SAK_RS01450	SAK_0294	-0.368875666	0.523208403	252254	252640	+	387	-
1490	SAK_RS07495	SAK_1490	-0.367794018	0.521763611	1479882	1481123	-	1242	-
620	SAK_RS03100	SAK_0620	-0.36773083	0.527759204	558375	559244	+	870	-
972	SAK_RS04865	SAK_0972	-0.359329456	0.528012075	925507	926016	+	510	-
1107	SAK_RS05535	SAK_1107	-0.358974695	0.52891986	1079971	1080732	-	762	rnhB
1227	SAK_RS06155	SAK_1227	-0.357509399	0.53366742	1198059	1198304	-	246	-
1380	SAK_RS06945	SAK_1380	-0.357182696	0.533783615	1365798	1367033	-	1236	-
1838	SAK_RS09255	SAK_1838	-0.354117328	0.532636863	1831366	1832658	-	1293	purA
1271	SAK_RS06375	SAK_1271	-0.346124368	0.539716597	1241262	1241972	-	711	-
1506	SAK_RS07585	SAK_1506	-0.345176269	0.542197233	1502960	1503802	-	843	-
517	SAK_RS02570	SAK_0517	-0.34458493	0.542197233	459256	462318	+	3063	-
1723	SAK_RS08665	SAK_1723	-0.343202217	0.542285989	1712228	1713352	+	1125	mutY
1678	SAK_RS08450	SAK_1678	-0.342142084	0.544008878	1663081	1663968	-	888	-
1989	SAK_RS10010	SAK_1989	-0.341779716	0.545689223	1978303	1978626	-	324	-
938	SAK_RS04690	SAK_0938	-0.338491393	0.54890596	890737	890952	+	216	-
1187	SAK_RS05940	SAK_1187	-0.336709464	0.547151512	1164039	1165172	-	1134	-
1369	SAK_RS06890	SAK_1369	-0.334841913	0.550221141	1353940	1355679	-	1740	-
195	SAK_RS00965	SAK_0195	-0.327337447	0.554895886	155947	157821	+	1875	-
799	SAK_RS03985	SAK_0799	-0.318648488	0.561984187	735195	737390	+	2196	cylI
1800	SAK_RS09060	SAK_1800	-0.314342479	0.567346933	1792496	1793347	-	852	-
1744	SAK_RS08770	SAK_1744	-0.312307349	0.568216735	1733587	1735872	+	2286	pepX
161	SAK_RS00795	SAK_0161	-0.310099844	0.570306978	119056	120420	+	1365	radA
1404	SAK_RS07065	SAK_1404	-0.307232424	0.573476498	1386547	1387725	-	1179	-
1802	SAK_RS09070	SAK_1802	-0.306595293	0.573503238	1794251	1794700	-	450	-
570	SAK_RS02840	SAK_0570	-0.305580492	0.573476498	513414	514046	+	633	nth
556	SAK_RS02770	SAK_0556	-0.303425751	0.575797889	498010	499083	+	1074	-
1019	SAK_RS05100	SAK_1019	-0.302988015	0.575797889	985283	985624	+	342	-
400	SAK_RS01985	SAK_0400	-0.302888205	0.575797889	348107	349408	+	1302	-
1583	SAK_RS07985	SAK_1583	-0.30004237	0.578408334	1579647	1580123	-	477	-
2106	SAK_RS10610	SAK_2106	-0.296707189	0.579666834	2097997	2098536	-	540	-
1817	SAK_RS09150	SAK_1817	-0.293582404	0.581973303	1807342	1809069	-	1728	proWX
2034	SAK_RS10235	SAK_2034	-0.292502849	0.5814861	2026693	2027244	-	552	tag
1261	SAK_RS06325	SAK_1261	-0.29187914	0.582872033	1231720	1232451	-	732	cpsB
1375	SAK_RS06920	SAK_1375	-0.289999572	0.584622965	1360788	1361321	-	534	-
338	SAK_RS01675	SAK_0338	-0.288747312	0.584927671	281765	282913	+	1149	nagA
634	SAK_RS03170	SAK_0634	-0.288141795	0.587220305	566972	567160	+	189	-
837	SAK_RS04190	SAK_0837	-0.287974171	0.616490813	783490	785433	+	1944	thrS
2026	SAK_RS10195	SAK_2026	-0.285957228	0.587100557	2020496	2021983	-	1488	-
2037	SAK_RS10250	SAK_2037	-0.285277015	0.587220305	2029123	2031096	-	1974	mutL
1705	SAK_RS08585	SAK_1705	-0.279769562	0.607926001	1696528	1696962	-	435	nusB
1224	SAK_RS06140	SAK_1224	-0.270800047	0.61265392	1196963	1197157	-	195	-
1225	SAK_RS06145	SAK_1225	-0.269419035	0.601167844	1197167	1197748	-	582	-
667	SAK_RS03335	SAK_0667	-0.26460897	0.606149569	600310	601434	+	1125	-
1541	SAK_RS07765	SAK_1541	-0.263693166	0.60341954	1535384	1536328	-	945	nikB
784	#N/A	SAK_0784	-0.261637861	0.607926001	721133	721803	+	671	-
1566	SAK_RS07900	SAK_1566	-0.261248473	0.607926001	1561417	1562115	-	699	fabG
1493	#N/A	SAK_1493	-0.261091311	0.608091239	1483672	1487448	-	3777	-
697	#N/A	SAK_0697	-0.259901455	0.595259316	629936	630097	-	162	-
1854	SAK_RS09335	SAK_1854	-0.259738399	0.608859416	1849628	1851160	+	1533	ahpF
735	#N/A	SAK_0735	-0.258299749	0.600771152	673376	673477	+	102	-
445	SAK_RS02210	SAK_0445	-0.254528514	0.612067594	391339	391842	+	504	-
1410	SAK_RS07095	SAK_1410	-0.252906902	0.61268019	1392874	1394040	-	1167	aroC
477	SAK_RS02380	SAK_0477	-0.252643877	0.61268019	421413	422084	+	672	-
1657	SAK_RS08350	SAK_1657	-0.250255502	0.613572805	1648741	1649430	-	690	-
1507	SAK_RS07590	SAK_1507	-0.246420175	0.613572805	1504069	1504743	+	675	-
414	SAK_RS02055	SAK_0414	-0.240871457	0.622226103	363873	365225	-	1353	-
702	SAK_RS03510	SAK_0702	-0.239282902	0.621791	634905	636206	+	1302	-
690	SAK_RS03450	SAK_0690	-0.236403453	0.626520219	624960	625235	+	276	-
1253	SAK_RS06285	SAK_1253	-0.232926255	0.618316546	1224764	1225711	-	948	-
1065	SAK_RS05325	SAK_1065	-0.231357681	0.629771049	1035903	1036646	+	744	-
208	SAK_RS01030	SAK_0208	-0.230825613	0.63032143	171745	172776	+	1032	-
1626	SAK_RS08200	SAK_1626	-0.229290592	0.63047464	1614870	1616330	-	1461	-
1398	SAK_RS07035	SAK_1398	-0.228887831	0.598337748	1382915	1383805	-	891	-
1307	SAK_RS06555	SAK_1307	-0.224921736	0.633311761	1284549	1286183	+	1635	-
1538	SAK_RS07750	SAK_1538	-0.224297806	0.636817348	1533135	1533815	-	681	nikE
665	#N/A	SAK_0665	-0.222104407	0.633388531	597277	598137	-	861	-
1135	SAK_RS05675	SAK_1135	-0.221649503	0.633388531	1110390	1111682	-	1293	pyrC
1170	SAK_RS05855	SAK_1170	-0.221221404	0.633388531	1147130	1148404	-	1275	pbuX
1024	SAK_RS05130	SAK_1024	-0.221091991	0.633283275	993347	993982	+	636	-
1016	SAK_RS05085	SAK_1016	-0.220633867	0.634665719	979244	979915	+	672	-
1994	SAK_RS10035	SAK_1994	-0.220498069	0.633311761	1986743	1987351	+	609	-
1619	SAK_RS08165	SAK_1619	-0.219721577	0.633388531	1609640	1610329	-	690	-
1439	SAK_RS07240	SAK_1439	-0.219241792	0.633388531	1419879	1420754	-	876	-
1742	SAK_RS08760	SAK_1742	-0.218785495	0.633283275	1731546	1732748	-	1203	-
374	SAK_RS01855	SAK_0374	-0.218353816	0.63374025	322200	323354	+	1155	-
2045	SAK_RS10285	SAK_2045	-0.215645972	0.634665719	2037968	2038912	-	945	-
756	SAK_RS03770	SAK_0756	-0.21498597	0.634665719	687988	691107	+	3120	-
431	SAK_RS02140	SAK_0431	-0.211822806	0.63897958	379501	380493	+	993	-
872	SAK_RS04360	SAK_0872	-0.206693872	0.642932998	817571	818680	+	1110	ribD
1278	SAK_RS06410	SAK_1278	-0.206630472	0.639571324	1249573	1250292	-	720	budA
1654	SAK_RS08335	SAK_1654	-0.20652259	0.64350579	1645331	1646707	-	1377	-
1495	SAK_RS07525	SAK_1495	-0.204180632	0.64350579	1489421	1491412	-	1992	uvrB
1860	SAK_RS09365	SAK_1860	-0.202570216	0.64350579	1856957	1857940	+	984	-
676	SAK_RS03380	SAK_0676	-0.199224809	0.646971279	610883	612076	+	1194	-
1631	SAK_RS08225	SAK_1631	-0.198328915	0.647978304	1620807	1621424	-	618	-
1204	SAK_RS06025	SAK_1204	-0.197207931	0.647978304	1180891	1181757	-	867	thrB
1325	SAK_RS06655	SAK_1325	-0.196552079	0.647031011	1307631	1308866	+	1236	-
1026	SAK_RS05140	SAK_1026	-0.195282518	0.649850644	994455	994889	+	435	msrB
1530	SAK_RS07710	SAK_1530	-0.19393147	0.650369234	1525949	1526752	+	804	-
1872	SAK_RS09425	SAK_1872	-0.191915932	0.650587689	1871641	1872900	-	1260	-
351	SAK_RS01740	SAK_0351	-0.191511054	0.649850644	297450	297755	+	306	-
463	SAK_RS02305	SAK_0463	-0.19128827	0.647978304	407249	409891	+	2643	polA
443	SAK_RS02200	SAK_0443	-0.190103363	0.647978304	390291	390587	-	297	-

776	SAK_RS03870	SAK_0776	-0.189646196	0.648432892	711448	713112	+	1665	-
1704	SAK_RS08580	SAK_1704	-0.189302214	0.650587689	1695479	1696441	+	963	scrR
1751	SAK_RS08805	SAK_1751	-0.18899753	0.647978304	1743355	1744563	-	1209	-
1481	SAK_RS07450	SAK_1481	-0.187007074	0.653554576	1467235	1468743	-	1509	-
1766	SAK_RS08895	SAK_1766	-0.186520652	0.650720644	1761779	1762309	-	531	-
1284	SAK_RS06440	SAK_1284	-0.184074381	0.654578158	1255748	1258993	+	3246	hylB
559	SAK_RS02785	SAK_0559	-0.184064775	0.654578158	501190	502296	+	1107	-
493	SAK_RS02460	SAK_0493	-0.182899316	0.653637317	433797	434573	-	777	recX
494	SAK_RS02465	SAK_0494	-0.181391548	0.653554576	434672	436027	+	1356	rumA
801	SAK_RS03995	SAK_0801	-0.181283749	0.653554576	738614	739150	+	537	cylK
1717	SAK_RS08635	SAK_1717	-0.178827609	0.654578158	1705290	1708118	-	2829	uvrA
1841	SAK_RS09270	SAK_1841	-0.178630812	0.656245803	1835257	1837509	+	2253	-
681	SAK_RS03405	SAK_0681	-0.177069102	0.656245803	615833	616723	+	891	-
636	SAK_RS03180	SAK_0636	-0.176033319	0.656453622	568764	569228	+	465	-
1853	SAK_RS09330	SAK_1853	-0.175596478	0.650587689	1849050	1849610	+	561	ahpC
243	SAK_RS01195	SAK_0243	-0.174746928	0.650369234	207758	208081	+	324	-
1542	SAK_RS07770	SAK_1542	-0.174434391	0.658832859	1536315	1537931	-	1617	nikA
782	SAK_RS03900	SAK_0782	-0.172876688	0.659404293	719698	720303	+	606	-
1304	SAK_RS06540	SAK_1304	-0.172559995	0.659260455	1281495	1282079	-	585	-
501	SAK_RS02500	SAK_0501	-0.170390388	0.660223813	444502	446670	+	2169	-
714	SAK_RS03570	SAK_0714	-0.16898553	0.660430834	646894	647958	-	1065	-
1413	SAK_RS07110	SAK_1413	-0.168977855	0.660223813	1395879	1397036	-	1158	-
1260	SAK_RS06320	SAK_1260	-0.165767582	0.659404293	1231019	1231711	-	693	cpsC
1996	SAK_RS10045	SAK_1996	-0.162858194	0.668875802	1990319	1991566	+	1248	-
1429	SAK_RS07190	SAK_1429	-0.159485582	0.670007976	1411414	1412067	-	654	-
269	SAK_RS01325	SAK_0269	-0.159462407	0.667446107	235251	235433	+	183	-
711	SAK_RS03555	SAK_0711	-0.156213856	0.670007976	644328	644813	-	486	-
1298	SAK_RS06510	SAK_1298	-0.155780266	0.67138155	1273220	1274458	-	1239	-
1966	SAK_RS09895	SAK_1966	-0.154432884	0.670007976	1956402	1957019	-	618	-
207	SAK_RS01025	SAK_0207	-0.150677798	0.676716444	170821	171735	+	915	oppD
1489	SAK_RS07490	SAK_1489	-0.149301317	0.67138155	1478708	1479892	-	1185	-
1305	SAK_RS06545	SAK_1305	-0.146687291	0.6772854	1282176	1283582	-	1407	pepV
850	SAK_RS04255	SAK_0850	-0.141114127	0.668875802	795642	799181	+	3540	smc
1436	SAK_RS07225	SAK_1436	-0.138844243	0.650587689	1417758	1418342	-	585	-
1567	SAK_RS07905	SAK_1567	-0.13867276	0.688848576	1562108	1562344	-	237	-
1670	SAK_RS08410	SAK_1670	-0.135681102	0.669650812	1657821	1658555	-	735	-
1320	SAK_RS06630	SAK_1320	-0.133634627	0.691219602	1298934	1302386	-	3453	scpB
523	SAK_RS02600	SAK_0523	-0.133228676	0.656453622	465273	467333	+	2061	-
1212	SAK_RS06070	SAK_1212	-0.133132197	0.691902938	1188130	1189137	-	1008	-
913	SAK_RS04565	SAK_0913	-0.132237908	0.685080408	863122	863730	+	609	sodA
89	SAK_RS00440	SAK_0089	-0.130780633	0.686174933	74513	75793	+	1281	-
598	SAK_RS02980	SAK_0598	-0.126178293	0.71158824	542103	542318	+	216	xseB
881	SAK_RS04405	SAK_0881	-0.122619469	0.694814146	825766	826614	-	849	-
2036	SAK_RS10245	SAK_2036	-0.121341476	0.682297755	2027859	2029091	-	1233	-
210	SAK_RS01040	SAK_0210	-0.118843404	0.697497622	173835	174767	+	933	oppF
897	SAK_RS04485	SAK_0897	-0.118547687	0.695032281	846862	848406	+	1545	prfC
1574	SAK_RS07940	SAK_1574	-0.118343406	0.695507173	1569505	1570200	-	696	-
1022	SAK_RS05120	SAK_1022	-0.116025769	0.701437319	988512	990344	+	1833	lepA
2087	#N/A	SAK_2087	-0.114254372	0.702908417	2079069	2079638	-	570	-
1599	SAK_RS08065	SAK_1599	-0.113236608	0.698411751	1593956	1594201	-	246	-
951	SAK_RS04755	SAK_0951	-0.111485388	0.697322328	905116	905613	+	498	-
773	SAK_RS03855	SAK_0773	-0.109257252	0.703892491	708384	708659	-	276	-
906	SAK_RS04530	SAK_0906	-0.098608024	0.716779715	855232	855885	+	654	-
858	SAK_RS04295	SAK_0858	-0.096322328	0.720840527	805669	807831	+	2163	-
1921	SAK_RS09675	SAK_1921	-0.093488055	0.717597902	1924888	1926543	-	1656	-
1584	SAK_RS07990	SAK_1584	-0.088249627	0.725150213	1580178	1581359	-	1182	-
1588	SAK_RS08010	SAK_1588	-0.08320107	0.725010628	1584168	1585031	-	864	-
930	SAK_RS04650	SAK_0930	-0.079695503	0.725612393	882516	883142	+	627	-
848	SAK_RS04245	SAK_0848	-0.076327632	0.735891975	794404	794772	+	369	-
1865	SAK_RS09390	SAK_1865	-0.072150841	0.729509685	1861727	1862671	-	945	-
1173	SAK_RS05870	SAK_1173	-0.066935743	0.720597296	1150426	1151766	-	1341	-
1919	SAK_RS09665	SAK_1919	-0.061908763	0.725612393	1921678	1922508	-	831	rgfB
767	SAK_RS03820	SAK_0767	-0.058411758	0.725010628	704319	704531	+	213	-
1043	SAK_RS05215	SAK_1043	-0.053842209	0.746216659	1012907	1013536	+	630	-
1337	SAK_RS06730	SAK_1337	-0.049261157	0.756013166	1322624	1323568	-	945	mmuM
12	SAK_RS00055	SAK_0012	-0.049175939	0.729830767	10799	12085	+	1287	-
1156	SAK_RS05785	SAK_1156	-0.047044112	0.744868906	1133362	1135095	-	1734	-
88	SAK_RS00435	SAK_0088	-0.04571302	0.755394479	72918	74408	+	1491	thrC
330	SAK_RS01630	SAK_0330	-0.045265404	0.749248104	274846	275793	-	948	-
1732	SAK_RS08710	SAK_1732	-0.043261158	0.756013166	1720089	1722509	+	2421	-
1997	SAK_RS10050	SAK_1997	-0.042511239	0.75494372	1991609	1992418	-	810	-
1066	SAK_RS05330	SAK_1066	-0.042220739	0.759935601	1036779	1037627	+	849	-
498	SAK_RS02485	SAK_0498	-0.040236728	0.758261297	441613	442521	+	909	-
1167	SAK_RS05840	SAK_1167	-0.036690912	0.762004457	1144287	1144889	+	603	-
2111	SAK_RS10635	SAK_2111	-0.036559653	0.75494372	2101861	2103144	-	1284	-
496	SAK_RS02475	SAK_0496	-0.03483266	0.742144265	437094	437522	+	429	-
535	SAK_RS02660	SAK_0535	-0.03422256	0.754165972	477087	479288	+	2202	-
847	SAK_RS04240	SAK_0847	-0.032870371	0.767716542	793592	794401	+	810	-
899	SAK_RS04495	SAK_0899	-0.028108489	0.768457736	848993	849727	+	735	-
2094	SAK_RS10550	SAK_2094	-0.027652743	0.75494372	2084998	2086164	+	1167	-
1494	#N/A	SAK_1494	-0.023576462	0.758489942	1487831	1489323	+	1493	-
718	SAK_RS03590	SAK_0718	-0.018710185	0.768846129	651506	652861	+	1356	rumA
1292	SAK_RS06480	SAK_1292	-0.018048786	0.758608044	1265294	1265812	-	519	apt
1172	SAK_RS05865	SAK_1172	-0.015632779	0.768466917	1149240	1150223	-	984	guaC
1483	SAK_RS07460	SAK_1483	-0.014744646	0.77734695	1471131	1472123	-	993	asp3
1622	SAK_RS08180	SAK_1622	-0.014274868	0.774224194	1611668	1611946	+	279	-
1990	SAK_RS10015	SAK_1990	-0.008448113	0.783946375	1978616	1979308	-	693	-
1920	SAK_RS09670	SAK_1920	-0.005466183	0.790842059	1922547	1924730	-	2184	-
1053	SAK_RS05265	SAK_1053	-0.000783161	0.767716542	1022111	1023481	+	1371	nox
827	SAK_RS04140	SAK_0827	0.006778494	0.803281809	770501	771901	+	1401	uxaC
1690	SAK_RS08510	SAK_1690	0.009496105	0.768535779	1676314	1677696	-	1383	-
737	SAK_RS03670	SAK_0737	0.013246374	0.790842059	674690	675055	+	366	-
722	#N/A	SAK_0722	0.013491596	0.80123095	654904	660228	+	5325	-

218	SAK_RS01080	SAK_0218	0.014173891	0.801399041	181691	182401	+	711	adcC
1269	SAK_RS06365	SAK_1269	0.016561348	0.798575081	1239085	1240296	-	1212	deoB
362	SAK_RS01795	SAK_0362	0.016615119	0.790842059	310141	310953	+	813	-
1205	SAK_RS06030	SAK_1205	0.017902944	0.808557912	1181759	1183042	-	1284	hom
1431	SAK_RS07200	SAK_1431	0.023907149	0.754165972	1413179	1413967	-	789	pflA
1211	SAK_RS06065	SAK_1211	0.024530554	0.813648074	1186680	1188056	-	1377	-
710	SAK_RS03550	SAK_0710	0.031296895	0.806112633	643666	644307	+	642	serB
1598	#N/A	SAK_1598	0.031491128	0.809601261	1592635	1593801	-	1167	livK
482	SAK_RS02405	SAK_0482	0.034581454	0.820363131	425814	426629	+	816	-
660	SAK_RS03300	SAK_0660	0.034987949	0.815439636	590323	591543	-	1221	-
324	SAK_RS01600	SAK_0324	0.038688395	0.787363092	270039	270389	+	351	-
1014	SAK_RS05075	SAK_1014	0.04172686	0.815439636	977761	978498	+	738	-
1207	#N/A	SAK_1207	0.043482882	0.813648074	1184129	1184866	-	738	-
1912	SAK_RS09630	SAK_1912	0.045764087	0.808018227	1915327	1916379	-	1053	-
1653	SAK_RS08330	SAK_1653	0.045784301	0.823482079	1644268	1645338	-	1071	-
457	SAK_RS02275	SAK_0457	0.050593189	0.824716875	401507	402511	-	1005	-
1358	SAK_RS06835	SAK_1358	0.058038438	0.824716875	1340542	1341771	-	1230	-
1321	SAK_RS06635	SAK_1321	0.05864016	0.813710336	1302923	1303948	-	1026	-
244	SAK_RS01200	SAK_0244	0.058645393	0.831771161	208114	208740	+	627	-
1283	SAK_RS06435	SAK_1283	0.059978048	0.813759978	1255047	1255529	-	483	mutX
1727	SAK_RS08685	SAK_1727	0.06058739	0.837397003	1715067	1717406	-	2340	-
1559	SAK_RS07860	SAK_1559	0.063681056	0.768846129	1556415	1556684	-	270	-
209	SAK_RS01035	SAK_0209	0.065684913	0.813710336	172789	173835	+	1047	oppD
2069	SAK_RS10410	SAK_2069	0.067996187	0.842249492	2062058	2063572	+	1515	-
1933	SAK_RS09735	SAK_1933	0.069105753	0.837987743	1934944	1935414	-	471	-
1656	SAK_RS08345	SAK_1656	0.071651311	0.835884371	1647806	1648639	-	834	-
1601	SAK_RS08075	SAK_1601	0.072715782	0.824716875	1595016	1595645	-	630	upp
1652	SAK_RS08325	SAK_1652	0.072844969	0.836057263	1643579	1644271	-	693	metI
546	SAK_RS02715	SAK_0546	0.073168196	0.791821529	486768	487460	+	693	-
2122	SAK_RS10690	SAK_2122	0.075768657	0.837987743	2113016	2113426	+	411	-
877	SAK_RS04385	SAK_0877	0.077470491	0.843925747	822747	823649	+	903	-
1301	SAK_RS06525	SAK_1301	0.080402841	0.844224164	1275768	1276685	-	918	-
950	SAK_RS04750	SAK_0950	0.080665286	0.813648074	904400	905029	+	630	udk
511	SAK_RS02540	SAK_0511	0.081493685	0.837987743	453701	454016	-	316	-
1777	SAK_RS08945	SAK_1777	0.084486755	0.824716875	1770522	1770791	+	270	rpsN
1274	SAK_RS06390	SAK_1274	0.089467652	0.852242343	1244864	1245625	-	762	-
1191	SAK_RS05960	SAK_1191	0.101062103	0.844026277	1169022	1169951	+	930	-
1713	SAK_RS08615	SAK_1713	0.105131823	0.86485475	1701106	1701558	-	453	-
531	SAK_RS02640	SAK_0531	0.107578594	0.866941123	472910	473740	-	831	-
687	SAK_RS03435	SAK_0687	0.108798334	0.868220847	622158	623093	-	936	-
1875	SAK_RS09440	SAK_1875	0.111782044	0.857851698	1874658	1874999	-	342	yajC
194	SAK_RS00960	SAK_0194	0.117512411	0.871012075	154261	155811	-	1551	-
990	SAK_RS04955	SAK_0990	0.117855872	0.744868906	945541	945732	+	192	-
822	SAK_RS04115	SAK_0822	0.121695069	0.880862072	764592	766142	+	1551	-
1427	SAK_RS07180	SAK_1427	0.128199326	0.844224164	1409362	1410387	+	1026	fhuB
1491	SAK_RS07500	SAK_1491	0.128771989	0.868220847	1481120	1482325	-	1206	-
9	SAK_RS00040	SAK_0009	0.130201961	0.877799791	10032	10304	+	273	-
261	SAK_RS01285	SAK_0261	0.130243916	0.878494612	226518	227873	+	1356	ulaA
1923	SAK_RS09685	SAK_1923	0.13094789	0.865709617	1927213	1927869	-	657	phoU
2008	SAK_RS10100	SAK_2008	0.131508246	0.884241512	2002262	2003473	-	1212	deoB
2075	SAK_RS10440	SAK_2075	0.133054552	0.813648074	2070494	2070790	-	297	-
1169	SAK_RS05850	SAK_1169	0.136237429	0.844026277	1146247	1147035	+	789	-
1938	SAK_RS09760	SAK_1938	0.139422529	0.884166911	1937468	1938736	+	1269	-
497	#N/A	SAK_0497	0.144050382	0.893244474	437701	441403	+	3703	scpA
1013	SAK_RS05070	SAK_1013	0.145103661	0.882439255	976627	977757	+	1131	-
2115	SAK_RS10655	SAK_2115	0.146474027	0.893244474	2106106	2106972	-	867	-
1258	SAK_RS06310	SAK_1258	0.14859937	0.874294761	1228909	1230297	-	1389	cpsE
2079	SAK_RS10455	SAK_2079	0.148740463	0.875889185	2072866	2073252	-	387	-
1256	SAK_RS06300	SAK_1256	0.149557676	0.865801714	1227954	1228436	-	483	-
1796	SAK_RS09040	SAK_1796	0.15176649	0.871012075	1788898	1790172	-	1275	-
878	SAK_RS04390	SAK_0878	0.151790988	0.870973876	823685	824326	-	642	-
1183	SAK_RS05920	SAK_1183	0.152700909	0.871167183	1160993	1162108	+	1116	-
86	SAK_RS00425	SAK_0086	0.153231056	0.725612393	68961	71603	+	2643	-
1294	SAK_RS06490	SAK_1294	0.154663053	0.893244474	1268651	1270849	-	2199	recJ
262	SAK_RS01290	SAK_0262	0.154730201	0.871919603	227876	228733	+	858	-
237	SAK_RS01165	SAK_0237	0.159764971	0.890748321	203775	204230	+	456	-
1074	SAK_RS05370	SAK_1074	0.162748148	0.888249397	1045289	1046950	+	1662	-
1813	SAK_RS09130	SAK_1813	0.172426136	0.882439255	1803206	1804393	-	1188	dltS
1642	SAK_RS08275	SAK_1642	0.177320394	0.879569896	1632870	1633424	-	555	-
81	SAK_RS00400	SAK_0081	0.177333208	0.899028989	63833	64741	+	909	-
2051	SAK_RS10315	SAK_2051	0.180120041	0.890748321	2044224	2044838	+	615	cadD
935	SAK_RS04675	SAK_0935	0.180940165	0.87602693	888356	889072	+	717	-
205	SAK_RS01015	SAK_0205	0.181888614	0.871167183	167571	168806	-	1236	-
1978	SAK_RS09955	SAK_1978	0.188528322	0.917868692	1965725	1966339	-	615	-
962	SAK_RS04815	SAK_0962	0.190743806	0.910846078	916794	917474	+	681	-
1858	SAK_RS09355	SAK_1858	0.191286009	0.888249397	1853911	1854864	+	954	-
552	SAK_RS02745	SAK_0552	0.199219037	0.932749146	495079	496071	+	993	asnA
193	SAK_RS00955	SAK_0193	0.200715698	0.910846078	153511	154251	-	741	-
1419	SAK_RS07140	SAK_1419	0.203369236	0.922003891	1401599	1402123	-	525	-
1930	SAK_RS09720	SAK_1930	0.205032118	0.899028989	1932657	1933610	-	954	prmA
1474	SAK_RS07410	SAK_1474	0.205971008	0.922888442	1458587	1459615	-	1029	-
5	#N/A	SAK_0005	0.206834276	0.922888442	4113	4229	+	117	-
1773	SAK_RS08925	SAK_1773	0.209114726	0.940662421	1767064	1767651	-	588	-
1137	SAK_RS05685	SAK_1137	0.210888485	0.930371688	1112336	1113037	-	702	pyrF
1633	SAK_RS08230	SAK_1633	0.213318173	0.899028989	1621589	1624687	-	3099	-
1465	SAK_RS07365	SAK_1465	0.214212726	0.935910013	1447293	1447796	-	504	-
1275	SAK_RS06395	SAK_1275	0.216122026	0.930371688	1245625	1246488	-	864	-
216	SAK_RS01070	SAK_0216	0.216358469	0.922985095	180308	181159	+	852	ipk
1819	SAK_RS09160	SAK_1819	0.221060494	0.860634817	1810687	1813065	-	2379	xpkA
1279	SAK_RS06415	SAK_1279	0.226845972	0.938026098	1250306	1251988	-	1683	budB
1482	SAK_RS07455	SAK_1482	0.227688247	0.899977066	1468757	1471144	-	2388	secA
1775	SAK_RS08935	SAK_1775	0.229998032	0.946168569	1768366	1769538	+	1173	-
1411	SAK_RS07100	SAK_1411	0.230417915	0.956185751	1394041	1395108	-	1068	aroB

1512	SAK_RS07615	SAK_1512	0.249320982	0.930566507	1508349	1510754	-	2406	rnr
1502	SAK_RS07565	SAK_1502	0.251221827	0.939960034	1497956	1499197	+	1242	pepS
777	SAK_RS03875	SAK_0777	0.256796733	0.958727998	713201	714124	+	924	-
1159	SAK_RS05800	SAK_1159	0.264329642	0.97211807	1137430	1138407	-	978	-
1360	SAK_RS06845	SAK_1360	0.270887129	0.956185751	1342459	1343085	-	627	-
1257	SAK_RS06305	SAK_1257	0.271763938	0.899028989	1228436	1228885	-	450	cpsF
1569	SAK_RS07915	SAK_1569	0.273458005	0.979664465	1562880	1563380	-	501	-
337	SAK_RS01670	SAK_0337	0.278756491	0.958703294	280780	281487	+	708	-
251	SAK_RS01235	SAK_0251	0.279187056	0.983877718	213874	214602	+	729	-
1232	SAK_RS06180	SAK_1232	0.292846306	0.998271181	1204949	1206184	+	1236	-
2104	SAK_RS10600	SAK_2104	0.302569038	0.965660819	2096180	2097052	+	873	sdhA
1231	SAK_RS06175	SAK_1231	0.305380234	0.997385155	1203540	1204886	+	1347	-
843	SAK_RS04220	SAK_0843	0.313045587	0.905796423	789629	790429	-	801	-
1206	SAK_RS06035	SAK_1206	0.314496131	0.965660819	1183219	1184106	+	888	-
1018	SAK_RS05095	SAK_1018	0.314747076	0.991984176	984417	985286	+	870	cas1
705	SAK_RS03525	SAK_0705	0.3148114	0.902602462	637304	637492	+	189	-
656	SAK_RS03280	SAK_0656	0.317298108	0.967027568	586485	586682	+	198	-
796	SAK_RS03970	SAK_0796	0.322325784	0.888511869	731370	732248	+	879	cylB
1814	SAK_RS09135	SAK_1814	0.32541865	0.992070522	1804393	1805067	-	675	dltr
1246	SAK_RS06250	SAK_1246	0.326522086	0.982904517	1217594	1218079	-	486	-
1818	SAK_RS09155	SAK_1818	0.331202029	0.998356824	1809088	1810311	-	1224	proV
1922	SAK_RS09680	SAK_1922	0.332489594	0.977018403	1926536	1927213	-	678	-
1210	#N/A	SAK_1210	0.335156839	0.899028989	1185698	1186537	-	840	-
1164	SAK_RS05825	SAK_1164	0.33674133	0.96381863	1142293	1142862	-	570	tdk
1759	SAK_RS08845	SAK_1759	0.33919626	0.983695175	1754737	1755840	-	1104	-
1981	SAK_RS09970	SAK_1981	0.340814035	0.971385761	1967732	1968595	+	864	-
2030	SAK_RS10215	SAK_2030	0.348638631	0.836057263	2023050	2023316	-	267	-
256	SAK_RS01260	SAK_0256	0.348705436	0.975266079	219180	219806	+	627	-
249	SAK_RS01225	SAK_0249	0.360351174	0.998271181	212507	213247	+	741	-
991	SAK_RS04960	SAK_0991	0.365309586	0.962349065	945807	946664	+	858	endA
1434	SAK_RS07215	SAK_1434	0.369921442	0.940662421	1416108	1417043	-	936	-
1028	SAK_RS05150	SAK_1028	0.370128866	0.940662421	997833	998936	+	1104	-
834	SAK_RS04175	SAK_0834	0.373676764	0.965660819	779188	780654	+	1467	-
838	SAK_RS04195	SAK_0838	0.374930517	1	785655	786359	+	705	-
797	SAK_RS03975	SAK_0797	0.386555381	0.956185751	732245	734248	+	2004	cylE
248	SAK_RS01220	SAK_0248	0.389329462	0.946168569	210781	212526	+	1746	-
380	SAK_RS01885	SAK_0380	0.390517908	0.946168569	329082	330167	+	1086	-
236	#N/A	SAK_0236	0.393014153	0.935910013	203597	203734	+	138	-
1805	SAK_RS09085	SAK_1805	0.395032805	0.941025278	1796221	1796493	-	273	-
922	SAK_RS04610	SAK_0922	0.39735452	0.949139564	873721	874749	-	1029	queA
1245	SAK_RS06245	SAK_1245	0.402066689	0.956185751	1216842	1217495	-	654	ung
794	SAK_RS03960	SAK_0794	0.405969146	0.925643667	729982	730458	+	477	cylZ
1516	SAK_RS07630	SAK_1516	0.413850719	0.930157007	1512658	1514304	-	1647	-
87	SAK_RS00430	SAK_0087	0.420003928	0.946168569	71782	72798	+	1017	adhA
1122	SAK_RS05610	SAK_1122	0.425643421	0.916316381	1093058	1093393	-	336	-
1485	SAK_RS07470	SAK_1485	0.430265981	0.977594383	1473686	1475230	-	1545	asp1
52	SAK_RS00255	SAK_0052	0.431563812	0.905796423	31399	32574	+	1176	-
1190	SAK_RS05955	SAK_1190	0.436166114	0.928467796	1167577	1168398	-	822	-
1982	SAK_RS09975	SAK_1982	0.437224339	0.897979654	1968752	1969054	-	303	-
472	SAK_RS02350	SAK_0472	0.443136378	0.998027208	416986	417525	+	540	-
1117	SAK_RS05585	SAK_1117	0.449994092	0.945372619	1089485	1090321	-	837	-
558	SAK_RS02780	SAK_0558	0.450831664	0.902044497	500561	501139	+	579	-
349	SAK_RS01730	SAK_0349	0.471544444	0.883480502	293697	295127	+	1431	-
167	SAK_RS00825	SAK_0167	0.471729245	0.893244474	126800	127732	-	933	rbsC
10	SAK_RS00045	SAK_0010	0.476455647	0.954369217	10291	10662	+	372	-
1578	SAK_RS07960	SAK_1578	0.480452507	0.888511869	1575271	1575966	-	696	-
1609	SAK_RS08115	SAK_1609	0.480548542	0.868220847	1603119	1603703	-	585	scpB
1892	SAK_RS09525	SAK_1892	0.491959076	0.889086844	1893377	1893652	-	276	-
607	SAK_RS03025	SAK_0607	0.492126964	0.86485475	548964	550106	-	1143	-
1106	SAK_RS05530	SAK_1106	0.492465278	0.905796423	1079402	1079950	-	549	-
1940	SAK_RS09770	SAK_1940	0.498519984	0.87602693	1939326	1940435	-	1110	-
1311	SAK_RS06575	SAK_1311	0.50132566	0.866653114	1290433	1290843	-	411	-
800	SAK_RS03990	SAK_0800	0.502941736	0.999769997	737395	738606	+	1212	cylJ
1577	SAK_RS07955	SAK_1577	0.518675713	0.874412315	1574238	1575113	+	876	-
219	SAK_RS01085	SAK_0219	0.522725571	0.864807312	182391	183203	+	813	adcB
483	SAK_RS02410	SAK_0483	0.525687622	0.897979654	426637	427458	+	822	-
1636	SAK_RS08245	SAK_1636	0.529470459	0.893244474	1627099	1628274	-	1176	-
1531	SAK_RS07715	SAK_1531	0.534283565	0.868220847	1526843	1527829	-	987	-
1281	SAK_RS06425	SAK_1281	0.538214062	0.899028989	1253314	1254504	-	1191	-
1047	SAK_RS05235	SAK_1047	0.539966789	0.852176022	1015795	1016385	+	591	-
1059	SAK_RS05295	SAK_1059	0.543622645	0.837987743	1029646	1030773	-	1128	-
3	SAK_RS00015	SAK_0003	0.546222411	0.877513756	2823	3704	+	882	-
611	SAK_RS03045	SAK_0611	0.54815175	0.871012075	551413	551631	+	219	-
1263	SAK_RS06335	SAK_1263	0.549481319	0.87602693	1234103	1235026	+	924	cpsY
1734	SAK_RS08720	SAK_1734	0.557578534	0.820363131	1723177	1724271	-	1095	dinB
271	SAK_RS01335	SAK_0271	0.568121871	0.893244474	236766	237152	+	387	-
1643	SAK_RS08280	SAK_1643	0.569401948	0.824162512	1633552	1634265	+	714	gidB
675	SAK_RS03375	SAK_0675	0.569925493	0.866914988	608290	610797	+	2508	-
1581	SAK_RS07975	SAK_1581	0.572052581	0.85476476	1578422	1579249	+	828	xth
1262	SAK_RS06330	SAK_1262	0.572916305	0.831771161	1232457	1233914	-	1458	-
657	SAK_RS03285	SAK_0657	0.581722995	0.816677979	586726	587658	-	933	pyrD
1121	SAK_RS05605	SAK_1121	0.583047473	0.844026277	1092693	1093049	-	357	-
1676	SAK_RS08440	SAK_1676	0.585561243	0.893244474	1661319	1662437	-	1119	-
817	SAK_RS04090	SAK_0817	0.591406641	0.842249492	759453	760226	+	774	-
2103	SAK_RS10595	SAK_2103	0.592207888	0.824716875	2095497	2096165	+	669	sdhB
170	SAK_RS00840	SAK_0170	0.593082767	0.824716875	129610	130521	-	912	rbsK
890	SAK_RS04450	SAK_0890	0.593519504	0.830553415	837389	839434	+	2046	-
909	SAK_RS04545	SAK_0909	0.595715251	0.796388389	859052	859996	+	945	-
1282	SAK_RS06430	SAK_1282	0.598550946	0.946168569	1254596	1255057	-	462	-
179	SAK_RS00885	SAK_0179	0.599813939	0.937198569	139079	139996	-	918	-
1316	SAK_RS06600	SAK_1316	0.600166739	0.822652106	1293687	1293977	+	291	-
1880	SAK_RS09465	SAK_1880	0.600841045	0.790842059	1879853	1881379	+	1527	-
1650	#N/A	SAK_1650	0.600852289	0.824716875	1640995	1642361	+	1367	brnQ

1778	SAK_RS08950	SAK_1778	0.60571468	0.834926041	1771152	1772177	-	1026	ltaE
2065	SAK_RS10390	SAK_2065	0.611720824	0.822089608	2057885	2058883	-	999	argF
1971	SAK_RS09920	SAK_1971	0.626145487	0.813648074	1960419	1961027	-	609	-
1379	SAK_RS06940	SAK_1379	0.626363543	0.837987743	1364924	1365667	-	744	-
978	SAK_RS04895	SAK_0978	0.636068943	0.779316833	934694	935827	+	1134	glgD
554	SAK_RS02755	SAK_0554	0.64209237	0.835884371	496697	497236	+	540	-
706	SAK_RS03530	SAK_0706	0.646146725	0.833442828	637917	639122	+	1206	-
1604	SAK_RS08090	SAK_1604	0.657255992	0.77734695	1597781	1599133	-	1353	-
1119	SAK_RS05595	SAK_1119	0.659105949	0.790842059	1090856	1091401	-	546	-
757	SAK_RS03775	SAK_0757	0.660119851	0.869641502	691104	691829	+	726	-
197	SAK_RS00975	SAK_0197	0.6603321	0.803895886	158826	159581	+	756	-
979	SAK_RS04900	SAK_0979	0.666051959	0.843762494	935824	937254	+	1431	glgA
902	SAK_RS04510	SAK_0902	0.666635335	0.758261297	851463	853049	+	1587	-
432	SAK_RS02145	SAK_0432	0.67224345	0.864453578	380531	380833	-	303	-
1025	SAK_RS05135	SAK_1025	0.673077612	0.802526818	993991	994455	+	465	-
1252	SAK_RS06280	SAK_1252	0.676625069	0.765550026	1223724	1224680	-	957	-
788	SAK_RS03930	SAK_0788	0.67964261	0.884453236	725598	726416	+	819	-
487	#N/A	SAK_0487	0.681275326	0.791177397	432606	432721	+	116	rrfG
754	SAK_RS03765	SAK_0754	0.684575264	0.893244474	687274	687693	+	420	-
846	SAK_RS04235	SAK_0846	0.688288725	0.785157832	792239	793588	+	1350	-
19	#N/A	SAK_0019	0.689040649	0.768846129	21206	21321	+	116	rrfA
1844	SAK_RS09285	SAK_1844	0.689451793	0.732136333	1839844	1840719	+	876	hslO
988	SAK_RS04945	SAK_0988	0.69162582	0.994537468	943969	944205	+	237	-
1918	#N/A	SAK_1918	0.694812656	0.861852711	1921118	1921462	-	345	-
596	SAK_RS02970	SAK_0596	0.708017991	0.747348133	539823	540659	+	837	-
1738	SAK_RS08740	SAK_1738	0.724561823	0.724726709	1728204	1728959	-	756	-
810	SAK_RS04045	SAK_0810	0.728335105	0.756013166	751971	753200	+	1230	-
32	#N/A	SAK_0032	0.733515846	0.756013166	27038	27153	+	116	rrfB
816	SAK_RS04085	SAK_0816	0.738030992	0.755394479	758800	759456	+	657	-
2073	SAK_RS10430	SAK_2073	0.753875947	0.756859352	2067475	2069793	-	2319	-
548	SAK_RS02725	SAK_0548	0.754628792	0.701437319	490372	491301	-	930	-
1038	SAK_RS05195	SAK_1038	0.757035669	0.77734695	1008989	1009546	+	558	lepB
911	SAK_RS04555	SAK_0911	0.766715213	0.725010628	861223	861951	+	729	-
75	SAK_RS00370	SAK_0075	0.768712291	0.725747584	56716	57519	-	804	-
1679	SAK_RS08455	SAK_1679	0.771515624	0.824716875	1664125	1665567	-	1443	gatB
1694	SAK_RS08530	SAK_1694	0.773471552	0.691219602	1680943	1682958	-	2016	recG
798	SAK_RS03980	SAK_0798	0.773978485	0.691219602	734245	735198	+	954	cylF
371	SAK_RS01840	SAK_0371	0.775508033	0.748284612	320085	320684	-	600	recU
1248	SAK_RS06260	SAK_1248	0.775920211	0.718575521	1219445	1220074	-	630	neuD
180	SAK_RS00890	SAK_0180	0.778537356	0.729830767	140235	140423	+	189	rpmB
1984	SAK_RS09985	SAK_1984	0.785375883	0.713411063	1970763	1971302	+	540	-
415	SAK_RS02060	SAK_0415	0.794205675	0.748284612	365319	365969	+	651	-
107	SAK_RS00530	SAK_0107	0.802177002	0.925086882	84000	84356	+	357	rplR
510	SAK_RS02535	SAK_0510	0.806231491	0.67138155	453222	453617	-	396	-
1857	SAK_RS09350	SAK_1857	0.807587258	0.740554071	1853508	1853744	-	237	-
1229	SAK_RS06165	SAK_1229	0.807775352	0.765569942	1201100	1201486	-	387	-
1089	SAK_RS05445	SAK_1089	0.80947129	0.654578158	1061872	1062603	-	732	-
1816	SAK_RS09145	SAK_1816	0.812000461	0.790762022	1805741	1807096	-	1356	-
239	SAK_RS01175	SAK_0239	0.814015364	0.668248292	204985	205755	-	771	proC
1110	SAK_RS05550	SAK_1110	0.819627701	0.67138155	1082619	1084103	-	1485	cstA
1308	SAK_RS06560	SAK_1308	0.819689076	0.660223813	1286225	1288006	-	1782	uvrC
259	SAK_RS01275	SAK_0259	0.828418384	0.700957184	224182	226218	+	2037	-
1881	SAK_RS09470	SAK_1881	0.834742767	0.656453622	1881381	1882061	+	681	-
412	#N/A	SAK_0412	0.835158064	0.72096857	363463	363578	+	116	rrfF
968	SAK_RS04845	SAK_0968	0.835314012	0.647978304	921765	923048	+	1284	-
521	SAK_RS02590	SAK_0521	0.83672504	0.735731221	463959	464297	-	339	-
1364	SAK_RS06865	SAK_1364	0.844627986	0.820363131	1347510	1349582	+	2073	-
2072	SAK_RS10425	SAK_2072	0.850708392	0.649850644	2067052	2067408	-	357	-
1973	SAK_RS09930	SAK_1973	0.852311645	0.716481238	1961878	1962366	-	489	-
1247	SAK_RS06255	SAK_1247	0.855102314	0.656245803	1218193	1219434	-	1242	neuA
695	SAK_RS03475	SAK_0695	0.856275145	0.725612393	628380	628784	+	405	-
795	SAK_RS03965	SAK_0795	0.856346478	0.660430834	730448	731377	+	930	cylA
2002	SAK_RS10075	SAK_2002	0.860509176	0.650587689	1995857	1996456	-	600	-
335	SAK_RS01660	SAK_0335	0.868233673	0.630738135	279573	280256	-	684	-
1859	SAK_RS09360	SAK_1859	0.871459778	0.638611901	1854914	1856809	-	1896	pepO
1182	SAK_RS05915	SAK_1182	0.881931142	0.697497622	1160015	1160989	+	975	prs
357	SAK_RS01770	SAK_0357	0.887387333	0.633283275	304000	304947	+	948	mraW
322	SAK_RS01590	SAK_0322	0.892210771	0.620735109	268104	269738	+	1635	-
2117	SAK_RS10665	SAK_2117	0.900218595	0.627395082	2107765	2109246	-	1482	guaB
1856	SAK_RS09345	SAK_1856	0.901723181	0.609411175	1851437	1853506	-	2070	-
1667	SAK_RS08395	SAK_1667	0.904751942	0.639978426	1655635	1656348	-	714	-
1740	SAK_RS08750	SAK_1740	0.907683613	0.804292535	1730059	1730886	-	828	-
1509	SAK_RS07600	SAK_1509	0.91698521	0.616657046	1505352	1506278	+	927	-
1100	SAK_RS05500	SAK_1100	0.917412516	0.691219602	1072187	1074307	-	2121	topA
266	SAK_RS01310	SAK_0266	0.920332063	0.612990002	231765	233894	+	2130	pnp
1839	SAK_RS09260	SAK_1839	0.925417134	0.743336033	1833029	1834051	-	1023	pfoR
204	SAK_RS01010	SAK_0204	0.931039699	0.608091239	166231	167418	-	1188	-
793	SAK_RS03955	SAK_0793	0.937986298	0.623514367	729693	729998	+	306	-
562	SAK_RS02800	SAK_0562	0.944904904	0.630738135	504742	506481	+	1740	-
447	SAK_RS02225	SAK_0447	0.949126697	0.62967992	392878	393912	+	1035	-
1848	SAK_RS09305	SAK_1848	0.95074918	0.578877528	1843167	1845614	-	2448	clpC
2077	SAK_RS10450	SAK_2077	0.951873469	0.720324263	2071866	2072372	-	507	-
1576	SAK_RS07950	SAK_1576	0.953229772	0.683437921	1572098	1574095	-	1998	metG
1520	SAK_RS07650	SAK_1520	0.954759894	0.716481238	1516673	1517521	-	849	-
121	#N/A	SAK_0121	0.955520599	0.640637297	95729	95844	+	116	rrfC
436	SAK_RS02165	SAK_0436	0.95596499	0.635320124	384095	384907	-	813	-
1399	SAK_RS07040	SAK_1399	0.956365548	0.656245803	1383789	1384253	-	465	lspA
635	SAK_RS03175	SAK_0635	0.957482687	0.750527819	567268	568683	+	1416	-
2068	SAK_RS10405	SAK_2068	0.957862878	0.848884859	2061333	2062055	+	723	-
561	SAK_RS02795	SAK_0561	0.96803594	0.584927671	503007	504752	+	1746	-
1830	SAK_RS09215	SAK_1830	0.970356601	0.712332252	1825266	1825982	-	717	araD
629	SAK_RS03145	SAK_0629	0.973367839	0.61268019	562613	563041	+	429	-
921	#N/A	SAK_0921	0.974706465	0.582090748	873197	873640	-	444	-

1523	#N/A	SAK_1523	0.978833485	0.579666834	1520319	1520573	-	255	-
241	SAK_RS01185	SAK_0241	0.987506079	0.636071861	207077	207316	-	240	-
1746	SAK_RS08780	SAK_1746	0.991545073	0.644989952	1736281	1737261	+	981	-
694	#N/A	SAK_0694	1.001162657	0.56517131	627496	628380	-	885	-
1692	SAK_RS08520	SAK_1692	1.001818297	0.547943296	1678762	1679640	-	879	aroE
2136	SAK_RS10760	SAK_2136	1.004227517	0.676716444	2126958	2127731	+	774	-
1362	SAK_RS06855	SAK_1362	1.005505872	0.595976611	1344937	1346754	-	1818	-
1009	SAK_RS05050	SAK_1009	1.006177808	0.661481534	973271	974230	+	960	-
1620	SAK_RS08170	SAK_1620	1.033895912	0.581973303	1610352	1610855	-	504	-
1492	SAK_RS07505	SAK_1492	1.035989658	0.61191597	1482334	1483341	-	1008	-
550	SAK_RS02735	SAK_0550	1.047164131	0.573649839	492838	493740	+	903	-
2009	SAK_RS10105	SAK_2009	1.047279775	0.553727419	2003540	2004211	-	672	deoC
288	SAK_RS01420	SAK_0288	1.061418727	0.514964712	249138	249473	-	336	-
1408	SAK_RS07085	SAK_1408	1.066056776	0.509402173	1390931	1392280	-	1350	gor
519	#N/A	SAK_0519	1.069745521	0.668875802	463511	463581	-	71	-
1505	SAK_RS07580	SAK_1505	1.075426042	0.591281446	1502047	1502769	-	723	-
700	SAK_RS03500	SAK_0700	1.078218909	0.501778249	632782	634158	+	1377	-
1648	SAK_RS08305	SAK_1648	1.078276729	0.591281446	1638894	1639442	-	549	-
1153	SAK_RS05765	SAK_1153	1.086011298	0.546293936	1130099	1130641	+	543	coaC
1165	SAK_RS05830	SAK_1165	1.088279185	0.519259366	1143000	1143182	+	183	-
1395	SAK_RS07020	SAK_1395	1.088454926	0.499498032	1379445	1381043	-	1599	-
214	#N/A	SAK_0214	1.09097673	0.56517131	179974	180089	+	116	rrfD
1318	SAK_RS06620	SAK_1318	1.093757333	0.530560764	1295297	1297765	-	2469	-
331	SAK_RS01635	SAK_0331	1.096413696	0.52891986	275790	276281	-	492	-
346	SAK_RS01715	SAK_0346	1.102465147	0.522873182	289742	291571	+	1830	glpO
1072	SAK_RS05360	SAK_1072	1.102915036	0.66722164	1042785	1043723	+	939	-
1344	#N/A	SAK_1344	1.111935985	0.501778249	1328986	1329132	+	147	-
1376	SAK_RS06925	SAK_1376	1.126215388	0.513085097	1361372	1361866	-	495	-
1359	SAK_RS06840	SAK_1359	1.148925155	0.519259366	1341771	1342457	-	687	-
1956	SAK_RS09855	SAK_1956	1.15287761	0.635320124	1951499	1951789	+	291	-
1964	SAK_RS09885	SAK_1964	1.153386894	0.633388531	1955121	1955522	-	402	-
446	SAK_RS02220	SAK_0446	1.154216049	0.729509685	392151	392876	+	726	ecsA
1277	SAK_RS06405	SAK_1277	1.16690793	0.49735054	1247864	1249519	+	1656	fbpA
1073	SAK_RS05365	SAK_1073	1.167327039	0.687351986	1043766	1044836	-	1071	xerS
1476	SAK_RS07420	SAK_1476	1.197789376	0.479647524	1461216	1462586	+	1371	-
272	SAK_RS01340	SAK_0272	1.201062082	0.56030962	237255	238010	+	756	-
62	SAK_RS00305	SAK_0062	1.214706172	0.444703166	43773	44525	+	753	-
698	SAK_RS03490	SAK_0698	1.218306423	0.576174127	630839	632116	+	1278	-
1488	SAK_RS07485	SAK_1488	1.220464891	0.49518538	1477840	1478715	-	876	-
2064	SAK_RS10385	SAK_2064	1.225330238	0.494427487	2056947	2057873	-	927	arcC
307	#N/A	SAK_0307	1.240718322	0.501778249	264561	264676	+	116	rrfE
1377	SAK_RS06930	SAK_1377	1.241268187	0.444703166	1362055	1364019	-	1965	-
1837	#N/A	SAK_1837	1.246676493	0.472597599	1830888	1831343	+	456	-
1093	SAK_RS05465	SAK_1093	1.251765361	0.471521343	1064301	1065185	-	885	truB
879	SAK_RS04395	SAK_0879	1.251780294	0.460347135	824348	824821	-	474	-
155	SAK_RS00765	SAK_0155	1.252413636	0.595965164	113294	114577	+	1284	tig
240	SAK_RS01180	SAK_0240	1.255086632	0.499498032	205825	206892	-	1068	pepA
1993	SAK_RS10030	SAK_1993	1.257569182	0.497962232	1985262	1986650	+	1389	-
1144	SAK_RS05720	SAK_1144	1.263526499	0.467722354	1121430	1123100	-	1671	fhs
910	SAK_RS04550	SAK_0910	1.269625683	0.435308424	860055	861047	-	993	-
863	SAK_RS04315	SAK_0863	1.272490202	0.519259366	809752	810525	+	774	lgt
1249	SAK_RS06265	SAK_1249	1.274316461	0.475217088	1220071	1221225	-	1155	neuC
953	SAK_RS04770	SAK_0953	1.281052688	0.609978251	907541	908476	-	936	-
1683	SAK_RS08475	SAK_1683	1.284667559	0.437406181	1670131	1670961	-	831	-
966	SAK_RS04835	SAK_0966	1.288261642	0.635320124	919857	921116	+	1260	murA
1071	SAK_RS05355	SAK_1071	1.293710127	0.450634609	1042120	1042788	+	669	-
247	SAK_RS01215	SAK_0247	1.297489863	0.557645418	210110	210754	+	645	-
1884	SAK_RS09485	SAK_1884	1.3080423	0.463008407	1884901	1886034	-	1134	msmK
551	SAK_RS02740	SAK_0551	1.327081691	0.393261066	493900	494964	+	1065	-
1329	#N/A	SAK_1329	1.328071024	0.466148493	1311820	1312896	-	1077	-
1339	SAK_RS06740	SAK_1339	1.329489702	0.437406181	1325079	1325729	-	651	-
1959	SAK_RS09865	SAK_1959	1.330227965	0.523407731	1952530	1952913	+	384	-
1397	SAK_RS07030	SAK_1397	1.340598837	0.463008407	1382218	1382739	-	522	pyrR
815	SAK_RS04080	SAK_0815	1.342479724	0.452966568	757945	758583	-	639	-
434	SAK_RS02155	SAK_0434	1.353379212	0.852242343	381938	382750	-	813	-
2044	SAK_RS10280	SAK_2044	1.356310634	0.393261066	2036988	2037860	-	873	-
2113	SAK_RS10645	SAK_2113	1.361312198	0.635320124	2104629	2104982	+	354	-
53	SAK_RS00260	SAK_0053	1.361506775	0.475217088	32564	33325	+	762	recO
1992	SAK_RS10025	SAK_1992	1.365611141	0.394720246	1984579	1985265	+	687	-
1340	SAK_RS06745	SAK_1340	1.37523863	0.520767451	1325887	1326210	-	324	-
2127	SAK_RS10715	SAK_2127	1.375383927	0.374613962	2118176	2118868	-	693	-
1176	SAK_RS05885	SAK_1176	1.387085666	0.49327433	1154935	1155696	-	762	-
1371	SAK_RS06900	SAK_1371	1.399202795	0.497962232	1356216	1358084	-	1869	-
2063	SAK_RS10380	SAK_2063	1.411929827	0.466148493	2055300	2056853	-	1554	-
918	SAK_RS04590	SAK_0918	1.436699683	0.47814981	869554	870696	+	1143	-
1130	SAK_RS05650	SAK_1130	1.439679883	0.453775717	1103770	1104552	-	783	-
1131	SAK_RS05655	SAK_1131	1.450865533	0.404184446	1104549	1104872	-	324	-
1710	#N/A	SAK_1710	1.45216392	0.513600141	1699881	1700051	-	171	-
1937	SAK_RS09755	SAK_1937	1.454064101	0.537718916	1936894	1937475	+	582	-
1905	SAK_RS09595	SAK_1905	1.460174471	0.389673739	1908884	1909921	-	1038	-
1111	SAK_RS05555	SAK_1111	1.469474951	0.36599435	1084259	1084993	-	735	-
1425	SAK_RS07170	SAK_1425	1.47232507	0.369303033	1407596	1408390	+	795	fhuC
459	SAK_RS02285	SAK_0459	1.472927783	0.729509685	403104	405338	+	2235	copA
1363	SAK_RS06860	SAK_1363	1.474231392	0.451489799	1346801	1347241	-	441	-
765	SAK_RS03810	SAK_0765	1.474805565	0.424994396	701777	702550	+	774	-
1466	SAK_RS07370	SAK_1466	1.477724424	0.527759204	1447879	1448685	-	807	-
321	SAK_RS01585	SAK_0321	1.485129579	0.393261066	267582	268040	+	459	-
803	SAK_RS04005	SAK_0803	1.488579593	0.530560764	739868	740383	+	516	-
278	SAK_RS01365	SAK_0278	1.493460745	0.377688025	241346	242500	-	1155	-
896	SAK_RS04480	SAK_0896	1.508736172	0.345542684	845147	846685	+	1539	-
407	SAK_RS02020	SAK_0407	1.526530837	0.55204352	357037	357702	+	666	-
345	SAK_RS01710	SAK_0345	1.538167991	0.389503316	288221	289729	+	1509	glpK
1976	SAK_RS09945	SAK_1976	1.545892667	0.314120856	1964192	1964398	-	207	-

522	SAK_RS02595	SAK_0522	1.558483933	0.321467013	464318	465082	-	765	-
964	SAK_RS04825	SAK_0964	1.563201562	0.497962232	918275	919045	+	771	thiM
396	SAK_RS01965	SAK_0396	1.567026371	0.893244474	345384	346139	+	756	-
348	SAK_RS01725	SAK_0348	1.567923223	0.391345805	292369	293706	+	1338	-
1999	#N/A	SAK_1999	1.573584992	0.319858607	1993337	1995052	-	1716	-
585	SAK_RS02915	SAK_0585	1.580226444	0.445352595	526733	527521	+	789	-
996	SAK_RS04985	SAK_0996	1.592047946	0.45733362	952208	955441	+	3234	rexB
2090	SAK_RS10525	SAK_2090	1.59839187	0.489124242	2081211	2081813	-	603	-
1250	SAK_RS06270	SAK_1250	1.608363955	0.300816021	1221302	1222327	-	1026	neuB
1946	SAK_RS09800	SAK_1946	1.610921478	0.418434995	1943447	1943866	-	420	-
1582	SAK_RS07980	SAK_1582	1.613345257	0.337156514	1579289	1579645	-	357	-
1845	SAK_RS09290	SAK_1845	1.621781414	0.371866911	1840703	1841680	+	978	-
408	SAK_RS02025	SAK_0408	1.622605717	0.359871121	357779	358333	+	555	yfiA
549	SAK_RS02730	SAK_0549	1.629050975	0.312810888	491718	492677	+	960	-
571	SAK_RS02845	SAK_0571	1.634082666	0.397322395	514047	514436	-	390	-
1338	SAK_RS06735	SAK_1338	1.635388329	0.704047122	1323573	1324949	-	1377	-
719	SAK_RS03595	SAK_0719	1.642839645	0.359463977	653383	653901	+	519	-
1497	SAK_RS07535	SAK_1497	1.681481817	0.36599435	1492576	1494759	+	2184	-
1693	SAK_RS08525	SAK_1693	1.686397485	0.320325033	1679738	1680652	-	915	-
1181	SAK_RS05910	SAK_1181	1.692789953	0.346946656	1159249	1159839	+	591	-
1276	SAK_RS06400	SAK_1276	1.696487646	0.36260743	1246501	1247505	-	1005	-
253	SAK_RS01245	SAK_0253	1.716649823	0.320171411	216586	217563	+	978	-
1254	SAK_RS06290	SAK_1254	1.736444506	0.480237822	1225745	1226719	-	975	cpsI
874	SAK_RS04370	SAK_0874	1.745782323	0.242286709	819329	820522	+	1194	ribBA
1957	SAK_RS09860	SAK_1957	1.747157176	0.370250573	1951890	1952264	+	375	-
1327	SAK_RS06670	SAK_1327	1.750213897	0.450520948	1310504	1311070	-	567	-
435	SAK_RS02160	SAK_0435	1.753408635	0.682297755	382783	383793	-	1011	-
1889	SAK_RS09510	SAK_1889	1.754277476	0.278581315	1889953	1890468	-	516	lacB
1708	SAK_RS08600	SAK_1708	1.75812841	0.391345805	1698921	1699220	-	300	-
780	SAK_RS03890	SAK_0780	1.762122348	0.333925851	715933	718605	+	2673	-
1963	#N/A	SAK_1963	1.768111618	0.314986183	1954690	1954893	-	204	-
1906	SAK_RS09600	SAK_1906	1.797201302	0.244276007	1909933	1910706	-	774	-
2031	SAK_RS10220	SAK_2031	1.801813303	0.428215382	2023518	2023916	-	399	spxA
1426	SAK_RS07175	SAK_1426	1.813092635	0.59736893	1408414	1409346	+	933	fhuD
997	SAK_RS04990	SAK_0997	1.837741785	0.356220247	955431	959054	+	3624	rexA
1803	SAK_RS09075	SAK_1803	1.839383567	0.36260743	1794704	1795270	-	567	-
225	SAK_RS01115	SAK_0225	1.847391711	0.322082233	196671	197036	+	366	-
1941	SAK_RS09775	SAK_1941	1.849431986	0.409953467	1940575	1940784	-	210	-
2018	SAK_RS10155	SAK_2018	1.861923157	0.307885744	2012873	2013670	-	798	-
245	SAK_RS01205	SAK_0245	1.86296088	0.340397624	208794	209510	-	717	-
1715	SAK_RS08625	SAK_1715	1.865813522	0.217383842	1702737	1703453	-	717	-
1412	SAK_RS07105	SAK_1412	1.879805063	0.419985019	1395202	1395879	-	678	aroD
1251	SAK_RS06275	SAK_1251	1.880126531	0.232234038	1222327	1223727	-	1401	cpsL
515	SAK_RS02560	SAK_0515	1.916488653	0.230277253	457100	457624	+	525	-
1608	SAK_RS08110	SAK_1608	1.920360921	0.254454943	1602407	1603129	-	723	rluB
1754	SAK_RS08820	SAK_1754	1.923847228	0.314120856	1747226	1748806	+	1581	hsdM
971	SAK_RS04860	SAK_0971	1.938055333	0.36260743	924870	925325	-	456	-
1760	SAK_RS08850	SAK_1760	1.950255724	0.274842775	1755853	1756302	-	450	-
1987	SAK_RS10000	SAK_1987	1.956885061	0.226079692	1973807	1975651	-	1845	-
633	SAK_RS03165	SAK_0633	1.957473361	0.230277253	565752	566972	+	1221	-
886	SAK_RS04430	SAK_0886	1.969177749	0.355988703	832636	833904	+	1269	-
344	SAK_RS01705	SAK_0344	1.9742165	0.306614774	287843	288106	+	264	-
789	SAK_RS03935	SAK_0789	1.982943569	0.243942227	726418	727203	+	786	-
824	SAK_RS04125	SAK_0824	1.985728541	0.359836159	767251	769050	+	1800	-
347	SAK_RS01720	SAK_0347	1.986886787	0.269276372	291583	292281	+	699	glpF
1324	SAK_RS06650	SAK_1324	2.004997097	0.238330535	1305401	1307215	-	1815	-
2083	SAK_RS10475	SAK_2083	2.006616092	0.212001013	2075461	2076903	-	1443	-
1605	SAK_RS08095	SAK_1605	2.014368007	0.332599435	1599339	1600688	+	1350	trkA
733	SAK_RS03655	SAK_0733	2.01525575	0.397050486	671392	672768	+	1377	-
696	#N/A	SAK_0696	2.016831797	0.202774204	628946	629428	+	483	-
473	SAK_RS02355	SAK_0473	2.017813875	0.198989025	417664	418440	+	777	-
2032	SAK_RS10225	SAK_2032	2.019567341	0.325649365	2024132	2025229	-	1098	recA
508	#N/A	SAK_0508	2.022735976	0.289486263	452633	452794	-	162	-
712	SAK_RS03560	SAK_0712	2.026550076	0.404184446	644826	645281	-	456	-
433	SAK_RS02150	SAK_0433	2.029229405	0.700754622	381012	381923	-	912	-
255	SAK_RS01255	SAK_0255	2.031606927	0.220443657	218393	219196	+	804	-
1428	SAK_RS07185	SAK_1428	2.048596131	0.241295652	1410384	1411385	+	1002	-
1460	SAK_RS07340	SAK_1460	2.049664332	0.213939594	1442974	1443315	-	342	-
2084	SAK_RS10480	SAK_2084	2.060986069	0.222864927	2076983	2077840	-	858	-
1757	SAK_RS08835	SAK_1757	2.078646735	0.254233211	1753374	1754018	-	645	-
2066	SAK_RS10395	SAK_2066	2.087522466	0.312810888	2058986	2060281	+	1296	-
1568	SAK_RS07910	SAK_1568	2.103900266	0.406289713	1562434	1562682	-	249	-
1216	SAK_RS06095	SAK_1216	2.10623917	0.389673739	1192141	1192674	+	534	-
1765	#N/A	SAK_1765	2.108402417	0.212001013	1760509	1761370	+	862	-
263	SAK_RS01295	SAK_0263	2.112438312	0.210300662	228730	229659	+	930	-
1498	SAK_RS07540	SAK_1498	2.116292048	0.216258089	1494759	1495499	+	741	-
839	SAK_RS04200	SAK_0839	2.128799175	0.184091563	786457	787476	+	1020	-
1112	SAK_RS05560	SAK_1112	2.129498823	0.21112807	1085005	1086744	-	1740	-
1579	SAK_RS07965	SAK_1579	2.130160752	0.175018813	1576302	1577519	+	1218	-
2118	SAK_RS10670	SAK_2118	2.141156416	0.768510042	2109403	2109888	-	486	-
646	SAK_RS03235	SAK_0646	2.144138781	0.220443657	574848	576380	+	1533	-
174	SAK_RS00860	SAK_0174	2.150581095	0.199504128	133487	134158	+	672	-
818	SAK_RS04095	SAK_0818	2.150756787	0.21112807	760390	761208	+	819	-
563	SAK_RS02805	SAK_0563	2.151898027	0.264006972	506609	507175	+	567	-
1638	SAK_RS08255	SAK_1638	2.160504324	0.219683069	1628902	1630407	-	1506	-
1180	SAK_RS05905	SAK_1180	2.163652655	0.219354656	1158468	1159139	-	672	-
924	SAK_RS04620	SAK_0924	2.17236957	0.173440403	875733	876434	+	702	nagB
372	SAK_RS01845	SAK_0372	2.191024529	0.181755726	320759	321277	+	519	-
2089	SAK_RS10520	SAK_2089	2.196017298	0.198989025	2080337	2080975	-	639	-
1711	#N/A	SAK_1711	2.202390815	0.239137121	1700504	1700629	-	126	-
916	SAK_RS04580	SAK_0916	2.21651681	0.21519047	866797	868224	+	1428	-
298	SAK_RS01470	SAK_0298	2.217632209	0.196880977	253798	254658	+	861	-
1977	SAK_RS09950	SAK_1977	2.219454877	0.184091563	1964400	1965476	-	1077	-

829	SAK_RS04150	SAK_0829	2.222309702	0.245811457	773089	773928	+	840	-
230	SAK_RS01135	SAK_0230	2.232218961	0.261607823	199803	200102	+	300	-
229	SAK_RS01130	SAK_0229	2.234374052	0.359463977	199493	199831	+	339	-
1361	SAK_RS06850	SAK_1361	2.234744737	0.173440403	1343193	1344947	-	1755	-
1162	SAK_RS05815	SAK_1162	2.235663743	0.244276007	1140349	1141179	-	831	-
1835	SAK_RS09240	SAK_1835	2.242582044	0.244439264	1828487	1829926	-	1440	ulaA
2025	SAK_RS10190	SAK_2025	2.249180491	0.264042606	2018200	2020371	-	2172	nrdD
917	SAK_RS04585	SAK_0917	2.249541642	0.198989025	868293	869387	-	1095	-
628	SAK_RS03140	SAK_0628	2.251305984	0.266063731	561959	562225	+	267	-
171	SAK_RS00845	SAK_0171	2.257862826	0.202774204	130514	131500	-	987	rbsR
1136	SAK_RS05680	SAK_1136	2.257926459	0.212001013	1111694	1112323	-	630	pyrE
1521	SAK_RS07655	SAK_1521	2.259477094	0.173440403	1517738	1519330	+	1593	-
1228	SAK_RS06160	SAK_1228	2.272084019	0.212001013	1198715	1200994	-	2280	pcrA
78	SAK_RS00385	SAK_0078	2.274431154	0.186437602	59825	60916	+	1092	purK
224	SAK_RS01110	SAK_0224	2.28084457	0.307885744	192907	196557	+	3651	rpoC
908	SAK_RS04540	SAK_0908	2.287822312	0.185392775	858232	859041	+	810	-
655	SAK_RS03275	SAK_0655	2.29696406	0.299007809	585900	586079	+	180	-
1396	SAK_RS07025	SAK_1396	2.298397542	0.222864927	1381099	1382169	-	1071	carA
1952	SAK_RS09835	SAK_1952	2.302302562	0.199504128	1948097	1948483	-	387	-
1125	SAK_RS05625	SAK_1125	2.303290505	0.291985895	1099099	1099341	-	243	-
291	SAK_RS01435	SAK_0291	2.3090944	0.174968901	250649	251056	+	408	-
976	SAK_RS04885	SAK_0976	2.311711632	0.189199204	931655	933523	+	1869	glgB
663	SAK_RS03315	SAK_0663	2.312447916	0.198989025	593818	594204	+	387	-
586	SAK_RS02920	SAK_0586	2.319848295	0.341207848	527531	528301	+	771	-
217	SAK_RS01075	SAK_0217	2.325501936	0.36260743	181245	181688	+	444	adcR
1975	SAK_RS09940	SAK_1975	2.329146978	0.174968901	1963293	1964195	-	903	-
260	SAK_RS01280	SAK_0260	2.337973527	0.19938339	226221	226505	+	285	-
619	SAK_RS03095	SAK_0619	2.340175126	0.21112807	556553	558106	+	1554	-
1236	#N/A	SAK_1236	2.341334627	0.231453519	1208372	1208443	-	72	-
391	SAK_RS01940	SAK_0391	2.342183903	0.184091563	340987	342006	+	1020	-
870	SAK_RS04350	SAK_0870	2.345977678	0.283096027	814827	815447	+	621	-
1898	SAK_RS09555	SAK_1898	2.347417927	0.244638428	1899996	1900919	-	924	-
1099	SAK_RS05495	SAK_1099	2.352723804	0.219683069	1071349	1072050	-	702	-
791	SAK_RS03945	SAK_0791	2.35978842	0.170990433	728133	728981	+	849	cylD
1943	SAK_RS09785	SAK_1943	2.362539194	0.186437602	1941036	1942166	-	1131	-
292	SAK_RS01440	SAK_0292	2.367287743	0.17239133	251056	251616	+	561	-
1724	SAK_RS08670	SAK_1724	2.367600962	0.17239133	1713529	1714122	+	594	-
814	SAK_RS04075	SAK_0814	2.369292356	0.279696141	757082	757867	+	786	-
402	SAK_RS01995	SAK_0402	2.373410609	0.199504128	352055	352723	+	669	-
1068	SAK_RS05340	SAK_1068	2.37430831	0.202774204	1038313	1039275	+	963	-
1840	SAK_RS09265	SAK_1840	2.38280305	0.189335928	1834413	1835087	+	675	-
1098	SAK_RS05490	SAK_1098	2.389659589	0.206957978	1068707	1071337	-	2631	-
1762	SAK_RS08860	SAK_1762	2.398510643	0.239137121	1756641	1758530	-	1890	-
1142	SAK_RS05710	SAK_1142	2.398554399	0.390415625	1117985	1119025	-	1041	-
928	SAK_RS04640	SAK_0928	2.399031689	0.254454943	879553	880500	+	948	-
1801	SAK_RS09065	SAK_1801	2.413914015	0.174265958	1793351	1794223	-	873	ksgA
2054	SAK_RS10330	SAK_2054	2.417977003	0.21112807	2046975	2047424	+	450	-
1974	SAK_RS09935	SAK_1974	2.424337512	0.165179892	1962535	1963296	-	762	-
1706	SAK_RS08590	SAK_1706	2.425951364	0.256553996	1696955	1697344	-	390	-
1132	SAK_RS05660	SAK_1132	2.428373758	0.174968901	1104999	1108181	-	3183	carB
1300	SAK_RS06520	SAK_1300	2.430363195	0.264888798	1275550	1275726	+	177	-
1517	SAK_RS07635	SAK_1517	2.437188856	0.174705686	1514294	1514995	-	702	-
1917	SAK_RS09655	SAK_1917	2.440007996	0.173440403	1919379	1920713	-	1335	rgfC
390	SAK_RS01935	SAK_0390	2.444065158	0.188667224	340295	340990	+	696	-
1924	SAK_RS09690	SAK_1924	2.445241783	0.221203875	1927866	1928615	-	750	pstB
72	SAK_RS00355	SAK_0072	2.447296213	0.17239133	53893	54810	+	918	-
557	SAK_RS02775	SAK_0557	2.455513855	0.170990433	499158	500492	+	1335	-
1662	SAK_RS08370	SAK_1662	2.456058486	0.17239133	1652125	1653114	+	990	-
963	SAK_RS04820	SAK_0963	2.45642816	0.219487391	917476	918273	+	798	thiD
1809	SAK_RS09105	SAK_1809	2.45668794	0.244276007	1798754	1800016	-	1263	dltD
1267	SAK_RS06355	SAK_1267	2.466041591	0.199821077	1237779	1238588	-	810	punA
297	SAK_RS01460	SAK_0297	2.467243187	0.256553996	253572	253676	+	105	-
608	SAK_RS03030	SAK_0608	2.467922755	0.221436801	550234	550443	-	210	-
287	SAK_RS01415	SAK_0287	2.471493708	0.213939594	248363	248833	+	471	-
1685	SAK_RS08485	SAK_1685	2.472316699	0.170990433	1671622	1672755	+	1134	-
572	SAK_RS02850	SAK_0572	2.47919324	0.209510112	514597	514806	+	210	-
894	SAK_RS04470	SAK_0894	2.485349496	0.258110751	842933	844153	+	1221	-
617	SAK_RS03085	SAK_0617	2.48577218	0.199504128	554684	556048	+	1365	-
60	SAK_RS00295	SAK_0060	2.486865879	0.155716174	42012	43034	+	1023	purM
1097	SAK_RS05485	SAK_1097	2.489260388	0.266063731	1067723	1068601	-	879	-
2024	SAK_RS10185	SAK_2024	2.499936688	0.184091563	2017982	2018125	-	144	-
1953	SAK_RS09840	SAK_1953	2.514240277	0.169868628	1948590	1949162	-	573	-
2019	SAK_RS10160	SAK_2019	2.529049756	0.184091563	2013856	2014716	+	861	-
1573	SAK_RS07935	SAK_1573	2.544736433	0.306331783	1568776	1569453	-	678	-
71	SAK_RS00350	SAK_0071	2.546885593	0.198989025	53234	53896	+	663	-
1343	SAK_RS06760	SAK_1343	2.561039737	0.188442309	1327699	1328925	-	1227	clpX
830	SAK_RS04155	SAK_0830	2.561067899	0.264339605	774081	774893	+	813	-
187	SAK_RS00930	SAK_0187	2.561419815	0.21423225	149264	149791	-	528	-
2067	SAK_RS10400	SAK_2067	2.563704137	0.17239133	2060278	2061111	+	834	-
1141	SAK_RS05705	SAK_1141	2.572473088	0.173809905	1116702	1117778	-	1077	asd
1471	SAK_RS07395	SAK_1471	2.575062884	0.202067542	1452906	1454279	-	1374	-
787	SAK_RS03925	SAK_0787	2.575866945	0.163741103	724603	725595	+	993	-
403	SAK_RS02000	SAK_0403	2.576257188	0.163741103	352791	353879	+	1089	gldA
250	SAK_RS01230	SAK_0250	2.578352142	0.198989025	213417	213872	+	456	-
1649	SAK_RS08310	SAK_1649	2.581309298	0.145369724	1639611	1640819	-	1209	ygjU
932	SAK_RS04660	SAK_0932	2.585154108	0.660223813	883984	884913	+	930	prsA
1669	SAK_RS08405	SAK_1669	2.586239634	0.189335928	1657547	1657798	-	252	-
1562	SAK_RS07875	SAK_1562	2.598676073	0.169868628	1558882	1559295	-	414	-
2013	SAK_RS10130	SAK_2013	2.609922117	0.198989025	2007549	2009171	-	1623	groEL
661	SAK_RS03305	SAK_0661	2.612959226	0.312810888	591543	592355	-	813	-
2125	SAK_RS10705	SAK_2125	2.613192078	0.184091563	2115966	2116922	+	957	arcC
177	SAK_RS00875	SAK_0177	2.614577026	0.145369724	136584	137972	+	1389	argH
790	SAK_RS03940	SAK_0790	2.617115951	0.241501982	727828	728133	+	306	cylX

176	SAK_RS00870	SAK_0176	2.624849894	0.145369724	135375	136565	+	1191	argG
1850	SAK_RS09315	SAK_1850	2.625269795	0.173632464	1846248	1846709	+	462	-
1932	SAK_RS09730	SAK_1932	2.627158558	0.145369724	1934189	1934905	+	717	-
59	SAK_RS00290	SAK_0059	2.63111072	0.170990433	40530	41984	+	1455	purF
514	SAK_RS02555	SAK_0514	2.633847816	0.145369724	456105	456968	-	864	-
1189	SAK_RS05950	SAK_1189	2.642094444	0.184091563	1166862	1167476	-	615	-
1887	SAK_RS09500	SAK_1887	2.648598305	0.189335928	1888031	1889008	-	978	lacD
166	SAK_RS00820	SAK_0166	2.653278895	0.184091563	125779	126747	-	969	rbsB
1214	#N/A	SAK_1214	2.658505261	0.186437602	1190350	1191693	+	1344	-
763	SAK_RS03800	SAK_0763	2.668865658	0.22553654	699217	700458	+	1242	-
1265	SAK_RS06345	SAK_1265	2.679939215	0.175018813	1235827	1236537	-	711	deoD
1986	SAK_RS09995	SAK_1986	2.681415212	0.199504128	1972753	1973460	-	708	-
781	#N/A	SAK_0781	2.685154308	0.198989025	718636	719449	+	814	-
758	SAK_RS03780	SAK_0758	2.68580473	0.200140125	691829	694744	+	2916	-
579	SAK_RS02885	SAK_0579	2.688180609	0.21112807	521094	522230	+	1137	divIB
727	SAK_RS03630	SAK_0727	2.69009331	0.21112807	666159	666722	-	564	-
2129	SAK_RS10725	SAK_2129	2.690501201	0.159466405	2120049	2121668	+	1620	-
1911	SAK_RS09625	SAK_1911	2.691584012	0.184091563	1914655	1915080	-	426	-
807	SAK_RS04030	SAK_0807	2.699558641	0.220443657	748666	749697	+	1032	-
1054	SAK_RS05270	SAK_1054	2.708268742	0.282938996	1023602	1024591	-	990	ldh
526	SAK_RS02615	SAK_0526	2.712729045	0.23648522	468206	469543	+	1338	-
871	SAK_RS04355	SAK_0871	2.717543837	0.161699253	815502	816842	-	1341	-
638	SAK_RS03190	SAK_0638	2.720027271	0.312810888	570130	570345	+	216	-
1716	SAK_RS08630	SAK_1716	2.720172849	0.161699253	1703455	1704954	-	1500	-
333	SAK_RS01650	SAK_0333	2.736663656	0.169868628	277493	278590	-	1098	-
828	SAK_RS04145	SAK_0828	2.736689468	0.170990433	771919	772965	+	1047	uxuA
67	SAK_RS00330	SAK_0067	2.744712723	0.166416628	49627	50943	+	1317	-
1988	SAK_RS10005	SAK_1988	2.748370258	0.145369724	1975696	1977933	-	2238	metE
701	SAK_RS03505	SAK_0701	2.749034502	0.188143552	634255	634908	+	654	vncR
465	SAK_RS02315	SAK_0465	2.764333961	0.170990433	410443	410922	+	480	-
293	SAK_RS01445	SAK_0293	2.766366834	0.140920456	251589	252269	+	681	-
2028	SAK_RS10205	SAK_2028	2.77021259	0.157497934	2022288	2022605	-	318	-
684	SAK_RS03420	SAK_0684	2.773434315	0.147719162	618617	620014	+	1398	pepDA
1594	SAK_RS08040	SAK_1594	2.775909301	0.167893773	1589229	1589939	-	711	livF
1925	SAK_RS09695	SAK_1925	2.783219444	0.153054468	1928608	1929486	-	879	pstA
455	SAK_RS02265	SAK_0455	2.783485383	0.186437602	398181	400964	+	2784	infB
1063	SAK_RS05315	SAK_1063	2.783767367	0.138762275	1033679	1034377	+	699	-
1127	SAK_RS05635	SAK_1127	2.791443327	0.145369724	1099807	1102824	-	3018	-
632	SAK_RS03160	SAK_0632	2.792443406	0.199504128	564491	565759	+	1269	-
1534	SAK_RS07730	SAK_1534	2.795404031	0.18748101	1530094	1530603	-	510	msrA
761	#N/A	SAK_0761	2.795639162	0.18704725	697031	698041	+	1011	-
1374	SAK_RS06915	SAK_1374	2.804761669	0.151534397	1360336	1360716	-	381	-
1890	SAK_RS09515	SAK_1890	2.806278276	0.153272897	1890489	1890914	-	426	lacA
534	SAK_RS02655	SAK_0534	2.806755336	0.17239133	476244	477077	+	834	-
1595	SAK_RS08045	SAK_1595	2.807212826	0.138762275	1589939	1590703	-	765	livG
1879	SAK_RS09460	SAK_1879	2.808748935	0.17239133	1878362	1879699	-	1338	-
1129	SAK_RS05645	SAK_1129	2.811506847	0.215204656	1103363	1103653	-	291	-
566	SAK_RS02820	SAK_0566	2.816589962	0.205408608	509343	510335	-	993	bioB
748	SAK_RS03735	SAK_0748	2.819503186	0.183472396	684194	685402	+	1209	-
226	SAK_RS10775	SAK_0226	2.826690434	0.186437602	197209	198180	+	972	comGA
2121	SAK_RS10685	SAK_2121	2.833350747	0.21112807	2111757	2112989	+	1233	arcA
499	SAK_RS02490	SAK_0499	2.835785491	0.146980978	443076	444086	+	1011	nrdF
172	SAK_RS00850	SAK_0172	2.842118752	0.158546106	131682	132770	+	1089	-
760	SAK_RS03790	SAK_0760	2.854424474	0.263815942	696631	697029	+	399	-
130	#N/A	SAK_0130	2.85690147	0.203767353	96542	96615	+	74	-
1825	SAK_RS09190	SAK_1825	2.861127822	0.155716174	1819683	1821134	-	1452	-
744	SAK_RS03715	SAK_0744	2.866432158	0.167893773	681525	681830	+	306	-
1758	SAK_RS08840	SAK_1758	2.871005483	0.169868628	1754036	1754725	-	690	-
859	SAK_RS04300	SAK_0859	2.871764511	0.184091563	807824	808252	+	429	-
232	SAK_RS01145	SAK_0232	2.879928929	0.205747703	200495	200866	+	372	-
809	SAK_RS04040	SAK_0809	2.882399921	0.21112807	750786	751847	+	1062	-
45	#N/A	SAK_0045	2.890318997	0.189199204	28215	28288	+	74	-
738	SAK_RS03680	SAK_0738	2.894551613	0.187140104	675788	677026	+	1239	-
568	SAK_RS02830	SAK_0568	2.898781015	0.174705686	510964	512079	+	1116	-
1701	SAK_RS08565	SAK_1701	2.900234463	0.21112807	1690985	1691866	-	882	scrK
169	SAK_RS00835	SAK_0169	2.900606234	0.155716174	129237	129635	-	399	rbsD
873	SAK_RS04365	SAK_0873	2.900813388	0.138762275	818661	819311	+	651	ribE
332	SAK_RS01645	SAK_0332	2.90221838	0.145369724	276861	277469	-	609	-
476	SAK_RS02375	SAK_0476	2.906315118	0.151534397	420889	421416	+	528	-
1467	SAK_RS07375	SAK_1467	2.908579878	0.186437602	1449010	1450029	-	1020	-
1570	SAK_RS07920	SAK_1570	2.910531185	0.156913217	1563620	1565032	-	1413	-
1603	SAK_RS08085	SAK_1603	2.924267525	0.189335928	1597060	1597608	-	549	-
64	SAK_RS00315	SAK_0064	2.937942747	0.142383873	46285	47184	+	900	zooA
2057	SAK_RS10345	SAK_2057	2.939783334	0.14117843	2049045	2049650	+	606	-
645	SAK_RS03230	SAK_0645	2.945573329	0.174968901	572842	574854	+	2013	-
168	SAK_RS00830	SAK_0168	2.945591738	0.134569919	127743	129221	-	1479	rbsA
1664	SAK_RS08380	SAK_1664	2.965103601	0.197739438	1653730	1654104	+	375	-
1120	SAK_RS05600	SAK_1120	2.966706291	0.158546106	1091407	1092693	-	1287	-
1445	SAK_RS07265	SAK_1445	2.967398586	0.175018813	1427724	1428872	-	1149	-
196	SAK_RS00970	SAK_0196	2.968529394	0.271103117	157867	158706	+	840	uppP
228	SAK_RS01125	SAK_0228	2.968692013	0.19854204	199114	199443	+	330	-
286	SAK_RS01410	SAK_0286	2.971379126	0.170990433	246939	248195	+	1257	pre
1950	SAK_RS09820	SAK_1950	2.971525707	0.208367958	1946624	1947214	+	591	-
1328	#N/A	SAK_1328	2.972439319	0.189335928	1311205	1311818	-	614	-
1039	#N/A	SAK_1039	2.977960265	0.174968901	1009560	1009703	-	144	-
1041	SAK_RS05205	SAK_1041	2.982635552	0.180357604	1011792	1012121	+	330	phnA
1691	SAK_RS08515	SAK_1691	2.993795088	0.13610747	1677765	1678727	+	963	-
70	SAK_RS00345	SAK_0070	2.996278257	0.163741103	52771	53214	+	444	-
529	SAK_RS02630	SAK_0529	2.998787092	0.173440403	471008	472339	+	1332	-
1050	SAK_RS05250	SAK_1050	3.000128279	0.138762275	1018359	1019894	+	1536	-
528	SAK_RS02625	SAK_0528	3.005430995	0.198989025	470531	470995	+	465	-
1319	SAK_RS06625	SAK_1319	3.00918625	0.189335928	1297778	1298698	-	921	lmb
898	SAK_RS04490	SAK_0898	3.009785451	0.202333988	848492	848872	+	381	-

730	SAK_RS03645	SAK_0730	3.017270423	0.145369724	668561	670846	+	2286	-
1607	SAK_RS08105	SAK_1607	3.017892578	0.184091563	1602156	1602407	-	252	-
137	SAK_RS00680	SAK_0137	3.01961608	0.220443657	97532	97963	+	432	-
642	SAK_RS03215	SAK_0642	3.020017524	0.163741103	571654	572211	+	558	-
1735	SAK_RS08725	SAK_1735	3.024024691	0.142383873	1724456	1726768	+	2313	pflB
1909	SAK_RS09615	SAK_1909	3.029827278	0.201513422	1913321	1914130	-	810	-
851	SAK_RS04260	SAK_0851	3.033300367	0.48494263	799272	800069	+	798	-
82	SAK_RS00405	SAK_0082	3.038119928	0.436170801	64942	65940	+	999	ruvB
1764	#N/A	SAK_1764	3.044320498	0.26500319	1759685	1760503	+	819	-
759	SAK_RS03785	SAK_0759	3.048435753	0.170990433	694757	696613	+	1857	-
1663	SAK_RS08375	SAK_1663	3.054888624	0.162222213	1653152	1653730	+	579	-
808	SAK_RS04035	SAK_0808	3.062525317	0.156913217	749732	750751	+	1020	-
2081	SAK_RS10465	SAK_2081	3.077543212	0.138762275	2073989	2074477	-	489	-
825	SAK_RS04130	SAK_0825	3.082306463	0.145369724	769079	769750	+	672	-
1849	SAK_RS09310	SAK_1849	3.083811468	0.271103117	1845611	1846075	-	465	ctsR
869	SAK_RS04345	SAK_0869	3.091093616	0.198989025	814491	814703	+	213	-
503	SAK_RS02510	SAK_0503	3.091289101	0.179638018	449913	450302	+	390	-
1954	SAK_RS09845	SAK_1954	3.098200234	0.208367958	1949164	1949496	-	333	-
734	SAK_RS03660	SAK_0734	3.108717491	0.166416628	672761	673258	+	498	-
1899	SAK_RS09560	SAK_1899	3.110225586	0.169868628	1901174	1901617	-	444	dtd
720	#N/A	SAK_0720	3.110937477	0.222864927	654026	654124	-	99	-
601	SAK_RS02995	SAK_0601	3.112811493	0.138762275	543997	544470	+	474	-
1910	SAK_RS09620	SAK_1910	3.116864922	0.21112807	1914146	1914637	-	492	-
1675	SAK_RS08435	SAK_1675	3.12170216	0.199504128	1660909	1661226	-	318	-
381	SAK_RS01890	SAK_0381	3.125272738	0.138762275	330169	330759	+	591	-
1444	SAK_RS07260	SAK_1444	3.135428115	0.169868628	1427213	1427485	-	273	-
533	SAK_RS02650	SAK_0533	3.139122933	0.170990433	475340	476230	+	891	-
1078	SAK_RS05390	SAK_1078	3.144433187	0.201780026	1050082	1050390	-	309	-
1051	SAK_RS05255	SAK_1051	3.144884289	0.114092804	1019887	1020948	+	1062	-
592	SAK_RS02950	SAK_0592	3.146932978	0.130158907	535433	536125	+	693	-
538	SAK_RS02675	SAK_0538	3.15431797	0.181924349	482046	483041	+	996	galE
792	SAK_RS03950	SAK_0792	3.156114395	0.142383873	728978	729700	+	723	cylG
1023	SAK_RS05125	SAK_1023	3.159508158	0.147719162	991064	993223	+	2160	-
1979	SAK_RS09960	SAK_1979	3.160764994	0.17239133	1966553	1966870	+	318	-
69	SAK_RS00340	SAK_0069	3.161068357	0.138762275	51928	52758	+	831	-
1672	SAK_RS08420	SAK_1672	3.1681725	0.160327046	1658979	1659500	-	522	-
242	SAK_RS01190	SAK_0242	3.169169865	0.170990433	207477	207761	+	285	-
742	SAK_RS03705	SAK_0742	3.174650676	0.145369724	679575	681167	+	1593	-
1407	SAK_RS07080	SAK_1407	3.175846746	0.145369724	1390435	1390887	+	453	-
313	#N/A	SAK_0313	3.176060654	0.170990433	265134	265207	+	74	-
1682	SAK_RS08470	SAK_1682	3.183403875	0.145369724	1667473	1670118	-	2646	ppdK
1895	SAK_RS09540	SAK_1895	3.209706747	0.155716174	1895647	1896111	-	465	-
429	SAK_RS02130	SAK_0429	3.210733943	0.145369724	377180	377812	+	633	-
1134	SAK_RS05670	SAK_1134	3.218777034	0.18704725	1109302	1110225	-	924	pyrB
542	SAK_RS02695	SAK_0542	3.220394278	0.155716174	484866	485060	+	195	-
2123	SAK_RS10695	SAK_2123	3.222873124	0.138762275	2113442	2114455	+	1014	argF
670	SAK_RS03350	SAK_0670	3.231868473	0.589033218	603296	604225	+	930	-
1776	SAK_RS08940	SAK_1776	3.233180483	0.169868628	1769581	1770363	-	783	-
1868	SAK_RS09405	SAK_1868	3.23477503	0.184091563	1863906	1864331	-	426	-
1832	SAK_RS09225	SAK_1832	3.242518111	0.17429977	1826851	1827516	-	666	ulaD
1824	SAK_RS09185	SAK_1824	3.266386348	0.163918506	1818672	1819661	-	990	-
61	SAK_RS00300	SAK_0061	3.280736076	0.108384563	43202	43753	+	552	purN
2124	SAK_RS10700	SAK_2124	3.282496823	0.555188193	2114518	2115945	+	1428	-
2082	SAK_RS10470	SAK_2082	3.283726866	0.145369724	2074551	2075096	-	546	-
957	#N/A	SAK_0957	3.289141132	0.198989025	913411	913539	+	129	-
68	SAK_RS00335	SAK_0068	3.289559717	0.138762275	51031	51918	+	888	-
1808	SAK_RS09100	SAK_1808	3.295835385	0.174968901	1798187	1798579	-	393	-
544	SAK_RS02705	SAK_0544	3.30392563	0.13513782	485614	485925	+	312	-
1637	SAK_RS08250	SAK_1637	3.310969196	0.172717352	1628274	1628753	-	480	nrdR
856	SAK_RS04285	SAK_0856	3.313487975	0.196648029	804260	804388	-	129	-
1770	SAK_RS08910	SAK_1770	3.322344741	0.145369724	1764492	1764791	+	300	-
762	#N/A	SAK_0762	3.341816249	0.169868628	697996	698436	+	441	-
58	SAK_RS00285	SAK_0058	3.346746511	0.11297899	36685	40296	+	3612	-
1448	SAK_RS07280	SAK_1448	3.369228385	0.303440104	1431131	1432495	-	1365	-
54	SAK_RS00265	SAK_0054	3.37786828	0.134569919	33415	34266	+	852	-
246	SAK_RS01210	SAK_0246	3.380616398	0.148556245	209591	209986	+	396	ssb2
1929	SAK_RS09715	SAK_1929	3.381003852	0.147719162	1931917	1932657	-	741	-
2058	SAK_RS10350	SAK_2058	3.387647641	0.132975096	2049856	2050461	+	606	-
2020	SAK_RS10165	SAK_2020	3.387761514	0.132543065	2014759	2015490	+	732	-
1934	SAK_RS09740	SAK_1934	3.390303112	0.145369724	1935386	1935844	-	459	-
1299	SAK_RS06515	SAK_1299	3.392048112	0.11149771	1274549	1275439	-	891	miaA
532	SAK_RS02645	SAK_0532	3.393399928	0.147719162	473979	475256	+	1278	-
365	SAK_RS01810	SAK_0365	3.395403046	0.186437602	312716	312940	+	225	-
1645	SAK_RS08290	SAK_1645	3.396554223	0.122319158	1635672	1636343	+	672	-
811	#N/A	SAK_0811	3.40329892	0.138762275	753210	754436	+	1227	-
466	SAK_RS02320	SAK_0466	3.413776887	0.138762275	411075	412640	+	1566	-
406	SAK_RS02015	SAK_0406	3.427588768	0.163741103	355748	357037	+	1290	-
900	SAK_RS04500	SAK_0900	3.43528724	0.118691185	849720	850382	+	663	-
1048	SAK_RS05240	SAK_1048	3.454514877	0.103346728	1016711	1017100	+	390	cdd
227	SAK_RS01120	SAK_0227	3.456852807	0.145369724	198215	199117	+	903	-
683	SAK_RS03415	SAK_0683	3.458988125	0.11149771	617691	618602	+	912	-
2099	SAK_RS10575	SAK_2099	3.465557534	0.143058407	2089367	2091349	-	1983	-
1665	SAK_RS08385	SAK_1665	3.465724793	0.123356837	1654114	1654827	+	714	glpF
520	SAK_RS02585	SAK_0520	3.467248478	0.138762275	463652	463957	-	306	-
1961	SAK_RS09870	SAK_1961	3.474096757	0.175018813	1953193	1953537	+	345	-
527	SAK_RS02620	SAK_0527	3.474890987	0.155716174	469566	470396	+	831	rhaD
1894	SAK_RS09535	SAK_1894	3.486060837	0.145369724	1895340	1895645	-	306	-
2074	SAK_RS10435	SAK_2074	3.499991667	0.145369724	2069932	2070471	+	540	-
2056	SAK_RS10340	SAK_2056	3.504276064	0.158610161	2047948	2049048	+	1101	-
1597	SAK_RS08055	SAK_1597	3.507646804	0.118937627	1591660	1592529	-	870	livH
441	SAK_RS02190	SAK_0441	3.515797339	0.160561618	388390	388911	+	522	-
1539	SAK_RS07755	SAK_1539	3.517439287	0.173440403	1533802	1534590	-	789	nikD
875	SAK_RS04375	SAK_0875	3.526573517	0.104483905	820537	821007	+	471	ribH

704	SAK_RS03520	SAK_0704	3.533652257	0.138762275	637083	637262	+	180	-
152	SAK_RS00750	SAK_0152	3.542933407	0.163741103	111228	111692	+	465	-
1726	#N/A	SAK_1726	3.543565494	0.138762275	1714563	1714949	-	387	-
745	SAK_RS03720	SAK_0745	3.544360241	0.138762275	681847	682119	+	273	-
1571	SAK_RS07925	SAK_1571	3.545275619	0.138762275	1565172	1567331	-	2160	-
806	#N/A	SAK_0806	3.569689302	0.105722142	746690	748634	+	1945	-
500	SAK_RS02495	SAK_0500	3.569953466	0.142383873	444087	444500	+	414	nrdI
1312	SAK_RS06580	SAK_1312	3.582717245	0.132543065	1290853	1291350	-	498	-
1867	SAK_RS09400	SAK_1867	3.586399012	0.140920456	1863159	1863773	-	615	-
779	SAK_RS03885	SAK_0779	3.587227489	0.132543065	715000	715851	+	852	-
883	SAK_RS04415	SAK_0883	3.603198474	0.138762275	827279	827668	+	390	-
594	SAK_RS02960	SAK_0594	3.61816072	0.105722142	537139	538833	+	1695	-
1196	SAK_RS05985	SAK_1196	3.621998717	0.134569919	1174266	1175420	-	1155	potA
509	SAK_RS02530	SAK_0509	3.625867301	0.186169556	452881	453198	-	318	-
639	SAK_RS03195	SAK_0639	3.629810952	0.134569919	570359	570790	+	432	-
1753	SAK_RS10780	SAK_1753	3.631812955	0.116292066	1745884	1746807	-	924	-
1124	SAK_RS05620	SAK_1124	3.651601645	0.138762275	1097822	1099099	-	1278	-
1540	SAK_RS07760	SAK_1540	3.668218456	0.138762275	1534578	1535339	-	762	nikC
1659	#N/A	SAK_1659	3.671347515	0.104483905	1650419	1651207	-	789	-
764	SAK_RS03805	SAK_0764	3.684187095	0.123356837	700361	701707	+	1347	-
545	SAK_RS02710	SAK_0545	3.684205458	0.115099042	486193	486771	+	579	-
1101	SAK_RS05505	SAK_1101	3.692950235	0.13513782	1074402	1075244	-	843	-
192	#N/A	SAK_0192	3.702152482	0.138762275	153000	153170	-	171	-
637	SAK_RS03185	SAK_0637	3.717725618	0.138762275	569231	570133	+	903	-
728	SAK_RS03635	SAK_0728	3.728476084	0.169868628	666727	667848	-	1122	-
1458	SAK_RS07330	SAK_1458	3.731866027	0.145369724	1440759	1441913	-	1155	rgpAc
1878	SAK_RS09455	SAK_1878	3.738515026	0.142383873	1877174	1878337	-	1164	-
1876	SAK_RS09445	SAK_1876	3.740965856	0.145369724	1875076	1875420	-	345	-
961	SAK_RS04810	SAK_0961	3.743015443	0.123175947	916042	916710	+	669	-
283	SAK_RS01390	SAK_0283	3.759354677	0.158610161	244336	244977	+	642	-
880	SAK_RS04400	SAK_0880	3.764463295	0.158610161	825116	825733	+	618	-
63	SAK_RS00310	SAK_0063	3.767487991	0.145369724	44545	46092	+	1548	purH
1470	SAK_RS07390	SAK_1470	3.775794318	0.118937627	1452446	1452751	+	306	-
1388	#N/A	SAK_1388	3.780318988	0.140920456	1373542	1373613	-	72	-
956	SAK_RS04790	SAK_0956	3.780451529	0.122319158	912706	913251	+	546	-
1831	SAK_RS09220	SAK_1831	3.78220027	0.114045074	1825984	1826847	-	864	-
234	SAK_RS01155	SAK_0234	3.78807494	0.095981098	201987	203180	+	1194	ackA
1533	SAK_RS07725	SAK_1533	3.821469833	0.151534397	1529882	1530070	-	189	-
282	SAK_RS01385	SAK_0282	3.822138606	0.142383873	244006	244332	+	327	-
450	#N/A	SAK_0450	3.828125246	0.138762275	395439	395526	+	88	-
1330	SAK_RS06695	SAK_1330	3.832363498	0.145369724	1312896	1313210	-	315	-
1926	SAK_RS09700	SAK_1926	3.841757274	0.104483905	1929488	1930333	-	846	pstC
1736	SAK_RS08730	SAK_1736	3.851411948	0.13513782	1726869	1727243	+	375	-
925	SAK_RS04625	SAK_0925	3.866511882	0.142383873	876506	877462	-	957	-
740	SAK_RS03695	SAK_0740	3.879565502	0.123356837	678619	679068	+	450	-
2011	SAK_RS10115	SAK_2011	3.906303198	0.053769112	2005464	2006243	-	780	udp
669	SAK_RS03345	SAK_0669	3.932986106	0.64350579	602620	603312	+	693	ftsE
593	SAK_RS02955	SAK_0593	3.935776438	0.145369724	536118	536852	+	735	-
699	SAK_RS03495	SAK_0699	3.944713043	0.118937627	632126	632782	+	657	-
778	SAK_RS03880	SAK_0778	3.98471269	0.138762275	714126	715043	+	918	-
1593	SAK_RS08035	SAK_1593	3.990506508	0.174388443	1588551	1589210	-	660	-
864	SAK_RS04320	SAK_0864	3.99617556	0.140920456	810540	810938	+	399	-
1914	SAK_RS09640	SAK_1914	4.000139805	0.11149771	1917464	1917742	-	279	-
1083	#N/A	SAK_1083	4.015058598	0.237386666	1056024	1056581	-	558	-
1421	SAK_RS07150	SAK_1421	4.026165339	0.147719162	1402353	1402844	-	492	-
1689	SAK_RS08505	SAK_1689	4.042822368	0.145369724	1675806	1676258	+	453	-
860	SAK_RS04305	SAK_0860	4.101656474	0.11149771	808333	808596	+	264	-
73	SAK_RS00360	SAK_0073	4.106913945	0.11149771	54827	55708	+	882	-
1927	SAK_RS09705	SAK_1927	4.115992335	0.130362767	1930348	1931229	-	882	-
2055	#N/A	SAK_2055	4.127798593	0.129734883	2047627	2047833	+	207	-
866	SAK_RS04330	SAK_0866	4.130012168	0.146980978	811407	811682	-	276	-
774	SAK_RS03860	SAK_0774	4.154658601	0.130362767	709317	709664	-	348	-
747	SAK_RS03730	SAK_0747	4.15736791	0.132975096	683482	684180	+	699	-
2041	SAK_RS10265	SAK_2041	4.193107476	0.145369724	2034215	2034652	-	438	argR
831	SAK_RS04160	SAK_0831	4.215961278	0.104483905	774909	776699	+	1791	-
1617	SAK_RS08155	SAK_1617	4.237678465	0.104483905	1608447	1608989	-	543	-
721	SAK_RS03600	SAK_0721	4.266638529	0.130362767	654203	654733	-	531	-
631	SAK_RS03155	SAK_0631	4.27295764	0.123361093	564138	564494	+	357	-
1154	SAK_RS05770	SAK_1154	4.338350311	0.11297899	1130687	1131259	+	573	-
1297	SAK_RS06505	SAK_1297	4.340702029	0.093228622	1272580	1273227	-	648	-
575	SAK_RS02865	SAK_0575	4.358175161	0.095981098	516395	518236	+	1842	typA
1827	SAK_RS09200	SAK_1827	4.451111289	0.116292066	1822207	1823226	-	1020	-
1580	SAK_RS07970	SAK_1580	4.482156412	0.11297899	1577503	1578345	+	843	-
1186	SAK_RS05935	SAK_1186	4.498893647	0.16335217	1163263	1163943	-	681	radC
1090	SAK_RS05450	SAK_1090	4.518260285	0.095981098	1062626	1062907	-	282	-
1671	SAK_RS08415	SAK_1671	4.526852902	0.11149771	1658621	1658977	-	357	-
141	SAK_RS00695	SAK_0141	4.531193932	0.11297899	100509	100988	+	480	comX
959	SAK_RS04800	SAK_0959	4.543129435	0.097120997	914210	914764	+	555	-
285	SAK_RS01405	SAK_0285	4.552053848	0.155716174	246188	246622	+	435	-
2080	SAK_RS10460	SAK_2080	4.562076644	0.082269681	2073227	2073589	-	363	-
1049	SAK_RS05245	SAK_1049	4.587827647	0.115099042	1017165	1018214	+	1050	-
539	SAK_RS02680	SAK_0539	4.617071287	0.123175947	483167	484036	+	870	-
2010	SAK_RS10110	SAK_2010	4.635932619	0.006747214	2004241	2005443	-	1203	-
1514	#N/A	SAK_1514	4.662414336	0.104483905	1511150	1511293	-	144	rpmG1
377	SAK_RS01870	SAK_0377	4.700361977	0.11149771	325726	327333	+	1608	-
1501	SAK_RS07560	SAK_1501	4.73873366	0.145369724	1497650	1497778	-	129	-
1113	SAK_RS05570	SAK_1113	4.768508687	0.145369724	1087720	1088094	-	375	-
600	SAK_RS02990	SAK_0600	4.828824767	0.138762275	543183	544010	+	828	tlyA
1687	SAK_RS08495	SAK_1687	4.865983993	0.104483905	1673417	1674202	-	786	codY
2078	#N/A	SAK_2078	4.876618416	0.145369724	2072646	2072756	-	111	-
11	SAK_RS00050	SAK_0011	4.892444244	0.14117843	10665	10799	+	135	-
1378	SAK_RS06935	SAK_1378	4.901927333	0.138762275	1364016	1364927	-	912	-
1563	SAK_RS07880	SAK_1563	4.918911112	0.054129749	1559308	1559685	-	378	-

1052	SAK_RS05260	SAK_1052	5.060122638	0.051933248	1020950	1021906	+	957	-
1826	SAK_RS09195	SAK_1826	5.132035039	0.082269681	1821232	1822188	-	957	-
1915	#N/A	SAK_1915	5.428309724	0.036877032	1917748	1918595	-	848	-
926	SAK_RS04630	SAK_0926	5.446231406	0.11149771	877558	878277	+	720	-
1822	SAK_RS09175	SAK_1822	5.550707512	0.11149771	1816308	1817069	-	762	-
1221	SAK_RS06125	SAK_1221	5.741589625	0.082269681	1195548	1196099	-	552	-
2021	SAK_RS10170	SAK_2021	5.770760919	0.087192822	2015847	2016464	-	618	nrdG
1010	SAK_RS05055	SAK_1010	5.7979383	0.174265958	974284	975636	+	1353	glmM
2097	SAK_RS10565	SAK_2097	6.137811167	0.184091563	2087511	2088866	-	1356	dnaC
2119	SAK_RS10675	SAK_2119	6.689581231	0.369303033	2109898	2110578	-	681	-
475	SAK_RS02370	SAK_0475	6.766968361	0.155716174	419218	420567	+	1350	pgi
295	SAK_RS01455	SAK_0295	8.515919513	0.497962232	252674	252955	+	282	-
1	SAK_RS00005	SAK_0001	NA	NA	101	1462	+	1362	dnaA
2	SAK_RS00010	SAK_0002	NA	NA	1617	2753	+	1137	dnaN
4	SAK_RS00020	SAK_0004	NA	NA	3714	3911	+	198	-
6	SAK_RS00025	SAK_0006	NA	NA	4473	5588	+	1116	ychF
7	SAK_RS00030	SAK_0007	NA	NA	5672	6247	+	576	pth
13	SAK_RS00060	SAK_0013	NA	NA	12087	13361	+	1275	tilS
14	SAK_RS00065	SAK_0014	NA	NA	13366	13908	+	543	hpt
15	SAK_RS00070	SAK_0015	NA	NA	13931	15907	+	1977	ftsH
16	#N/A	SAK_0016	NA	NA	16406	17912	+	1507	rrsA
17	#N/A	SAK_0017	NA	NA	18002	18074	+	73	-
18	#N/A	SAK_0018	NA	NA	18229	21131	+	2903	rrlA
20	#N/A	SAK_0020	NA	NA	21325	21397	+	73	-
21	#N/A	SAK_0021	NA	NA	21400	21472	+	73	-
22	#N/A	SAK_0022	NA	NA	21475	21547	+	73	-
23	#N/A	SAK_0023	NA	NA	21557	21638	+	82	-
24	#N/A	SAK_0024	NA	NA	21649	21721	+	73	-
25	#N/A	SAK_0025	NA	NA	21755	21826	+	72	-
26	#N/A	SAK_0026	NA	NA	21835	21919	+	85	-
27	#N/A	SAK_0027	NA	NA	21931	22004	+	74	-
28	#N/A	SAK_0028	NA	NA	22012	22085	+	74	-
29	#N/A	SAK_0029	NA	NA	22238	23744	+	1507	rrsB
30	#N/A	SAK_0030	NA	NA	23834	23906	+	73	-
31	#N/A	SAK_0031	NA	NA	24061	26963	+	2903	rrlB
33	#N/A	SAK_0033	NA	NA	27157	27229	+	73	-
34	#N/A	SAK_0034	NA	NA	27232	27304	+	73	-
35	#N/A	SAK_0035	NA	NA	27307	27379	+	73	-
36	#N/A	SAK_0036	NA	NA	27389	27470	+	82	-
37	#N/A	SAK_0037	NA	NA	27481	27553	+	73	-
38	#N/A	SAK_0038	NA	NA	27587	27658	+	72	-
39	#N/A	SAK_0039	NA	NA	27667	27751	+	85	-
40	#N/A	SAK_0040	NA	NA	27763	27836	+	74	-
41	#N/A	SAK_0041	NA	NA	27844	27917	+	74	-
42	#N/A	SAK_0042	NA	NA	27933	28006	+	74	-
43	#N/A	SAK_0043	NA	NA	28026	28099	+	74	-
44	#N/A	SAK_0044	NA	NA	28115	28204	+	90	-
46	#N/A	SAK_0046	NA	NA	28291	28363	+	73	-
47	#N/A	SAK_0047	NA	NA	28383	28453	+	71	-
48	#N/A	SAK_0048	NA	NA	28488	28561	+	74	-
49	#N/A	SAK_0049	NA	NA	28575	28662	+	88	-
50	SAK_RS00245	SAK_0050	NA	NA	28898	30199	+	1302	pcsB
51	SAK_RS00250	SAK_0051	NA	NA	30323	31291	+	969	prs
55	SAK_RS00270	SAK_0055	NA	NA	34344	35336	+	993	plsX
56	SAK_RS00275	SAK_0056	NA	NA	35347	35586	+	240	-
57	SAK_RS00280	SAK_0057	NA	NA	35710	36417	+	708	purC
77	SAK_RS00380	SAK_0077	NA	NA	59350	59838	+	489	purE
90	SAK_RS00445	SAK_0090	NA	NA	75998	76306	+	309	rpsJ
91	SAK_RS00450	SAK_0091	NA	NA	76411	77037	+	627	rplC
92	SAK_RS00455	SAK_0092	NA	NA	77061	77684	+	624	rplD
93	SAK_RS00460	SAK_0093	NA	NA	77684	77980	+	297	rplW
94	SAK_RS00465	SAK_0094	NA	NA	77998	78831	+	834	rplB
95	SAK_RS00470	SAK_0095	NA	NA	78930	79208	+	279	rpsS
96	SAK_RS00475	SAK_0096	NA	NA	79224	79568	+	345	rplV
97	SAK_RS00480	SAK_0097	NA	NA	79581	80234	+	654	rpsC
98	SAK_RS00485	SAK_0098	NA	NA	80238	80651	+	414	rplP
99	SAK_RS00490	SAK_0099	NA	NA	80661	80867	+	207	rpmC
100	SAK_RS00495	SAK_0100	NA	NA	80893	81153	+	261	rpsQ
101	SAK_RS00500	SAK_0101	NA	NA	81178	81546	+	369	rplN
102	SAK_RS00505	SAK_0102	NA	NA	81626	81931	+	306	rplX
103	SAK_RS00510	SAK_0103	NA	NA	81955	82497	+	543	rplE
104	SAK_RS00515	SAK_0104	NA	NA	82515	82700	+	186	rpsN
105	SAK_RS00520	SAK_0105	NA	NA	82855	83253	+	399	rpsH
106	SAK_RS00525	SAK_0106	NA	NA	83363	83899	+	537	rplF
108	SAK_RS00535	SAK_0108	NA	NA	84375	84869	+	495	rpsE
109	SAK_RS00540	SAK_0109	NA	NA	84884	85063	+	180	rpmD
110	SAK_RS00545	SAK_0110	NA	NA	85188	85628	+	441	rplO
111	SAK_RS00550	SAK_0111	NA	NA	85649	86953	+	1305	secY
112	SAK_RS00555	SAK_0112	NA	NA	87048	87686	+	639	adk
113	SAK_RS00560	SAK_0113	NA	NA	87802	88020	+	219	infA
114	SAK_RS00565	SAK_0114	NA	NA	88180	88545	+	366	rpsM
115	SAK_RS00570	SAK_0115	NA	NA	88563	88946	+	384	rpsK
116	SAK_RS00575	SAK_0116	NA	NA	88996	89934	+	939	rpoA
117	SAK_RS00580	SAK_0117	NA	NA	89949	90335	+	387	rplQ
118	#N/A	SAK_0118	NA	NA	90927	92433	+	1507	rrsC
119	#N/A	SAK_0119	NA	NA	92523	92595	+	73	-
120	#N/A	SAK_0120	NA	NA	92752	95654	+	2903	rrlC
122	#N/A	SAK_0122	NA	NA	95848	95920	+	73	-
123	#N/A	SAK_0123	NA	NA	95923	95995	+	73	-
124	#N/A	SAK_0124	NA	NA	95998	96070	+	73	-
125	#N/A	SAK_0125	NA	NA	96080	96161	+	82	-
126	#N/A	SAK_0126	NA	NA	96172	96244	+	73	-
127	#N/A	SAK_0127	NA	NA	96273	96349	+	77	-

128	#N/A	SAK_0128	NA	NA	96357	96428	+	72	-
129	#N/A	SAK_0129	NA	NA	96442	96531	+	90	-
131	#N/A	SAK_0131	NA	NA	96618	96690	+	73	-
132	#N/A	SAK_0132	NA	NA	96702	96782	+	81	-
133	#N/A	SAK_0133	NA	NA	96789	96859	+	71	-
134	#N/A	SAK_0134	NA	NA	96874	96946	+	73	-
135	#N/A	SAK_0135	NA	NA	96953	97024	+	72	-
136	#N/A	SAK_0136	NA	NA	97035	97118	+	84	-
138	#N/A	SAK_0138	NA	NA	98033	98203	+	171	-
145	SAK_RS00715	SAK_0145	NA	NA	103266	104300	+	1035	hrcA
146	SAK_RS00720	SAK_0146	NA	NA	104342	104875	+	534	grpE
147	SAK_RS00725	SAK_0147	NA	NA	105056	106885	+	1830	dnaK
148	SAK_RS00730	SAK_0148	NA	NA	107174	108289	+	1116	dnaJ
151	SAK_RS00745	SAK_0151	NA	NA	110460	111218	+	759	-
156	SAK_RS00770	SAK_0156	NA	NA	114766	115341	+	576	rpoE
157	SAK_RS00775	SAK_0157	NA	NA	115614	117218	+	1605	pyrG
159	#N/A	SAK_0159	NA	NA	118300	118385	+	86	-
160	SAK_RS00790	SAK_0160	NA	NA	118448	118894	+	447	dut
162	SAK_RS00800	SAK_0162	NA	NA	120556	121053	+	498	-
165	SAK_RS00815	SAK_0165	NA	NA	124303	125739	+	1437	gltX
178	SAK_RS00880	SAK_0178	NA	NA	138113	138994	+	882	fba
181	SAK_RS00895	SAK_0181	NA	NA	140555	140920	+	366	-
182	SAK_RS00900	SAK_0182	NA	NA	140953	142584	+	1632	-
185	SAK_RS00920	SAK_0185	NA	NA	145294	145512	-	219	-
198	SAK_RS00980	SAK_0198	NA	NA	159583	160743	+	1161	-
199	SAK_RS00985	SAK_0199	NA	NA	160908	161678	+	771	sufC
200	SAK_RS00990	SAK_0200	NA	NA	161715	162977	+	1263	sufD
201	SAK_RS00995	SAK_0201	NA	NA	162979	164211	+	1233	sufS
202	SAK_RS01000	SAK_0202	NA	NA	164198	164641	+	444	-
203	SAK_RS01005	SAK_0203	NA	NA	164741	166159	+	1419	sufB
211	#N/A	SAK_0211	NA	NA	175172	176678	+	1507	rrsD
212	#N/A	SAK_0212	NA	NA	176768	176840	+	73	-
213	#N/A	SAK_0213	NA	NA	176997	179899	+	2903	rrlD
215	#N/A	SAK_0215	NA	NA	180095	180168	+	74	-
221	SAK_RS01095	SAK_0221	NA	NA	185024	186283	-	1260	tyrS
223	SAK_RS01105	SAK_0223	NA	NA	189215	192790	+	3576	rpoB
231	SAK_RS01140	SAK_0231	NA	NA	200125	200517	+	393	-
235	SAK_RS01160	SAK_0235	NA	NA	203332	203538	+	207	-
265	SAK_RS01305	SAK_0265	NA	NA	231115	231384	+	270	rpsO
267	SAK_RS01315	SAK_0267	NA	NA	233896	234648	+	753	-
268	SAK_RS01320	SAK_0268	NA	NA	234657	235241	+	585	cysE
270	SAK_RS01330	SAK_0270	NA	NA	235430	236773	+	1344	cysS
275	#N/A	SAK_0275	NA	NA	239823	239942	+	120	-
276	SAK_RS01355	SAK_0276	NA	NA	240359	240805	+	447	rplM
277	SAK_RS01360	SAK_0277	NA	NA	240826	241218	+	393	rpsI
279	SAK_RS01370	SAK_0279	NA	NA	242569	243120	-	552	-
280	SAK_RS01375	SAK_0280	NA	NA	243285	243578	+	294	-
281	SAK_RS01380	SAK_0281	NA	NA	243719	244000	+	282	-
289	SAK_RS01425	SAK_0289	NA	NA	249463	249750	-	288	-
296	#N/A	SAK_0296	NA	NA	253046	253144	+	99	-
304	#N/A	SAK_0304	NA	NA	259761	261267	+	1507	rrsE
305	#N/A	SAK_0305	NA	NA	261357	261429	+	73	-
306	#N/A	SAK_0306	NA	NA	261584	264486	+	2903	rrlE
308	#N/A	SAK_0308	NA	NA	264680	264752	+	73	-
309	#N/A	SAK_0309	NA	NA	264758	264828	+	71	-
310	#N/A	SAK_0310	NA	NA	264865	264941	+	77	-
311	#N/A	SAK_0311	NA	NA	264949	265020	+	72	-
312	#N/A	SAK_0312	NA	NA	265034	265123	+	90	-
314	#N/A	SAK_0314	NA	NA	265210	265282	+	73	-
315	#N/A	SAK_0315	NA	NA	265294	265374	+	81	-
316	#N/A	SAK_0316	NA	NA	265381	265451	+	71	-
317	#N/A	SAK_0317	NA	NA	265466	265538	+	73	-
318	#N/A	SAK_0318	NA	NA	265545	265616	+	72	-
319	#N/A	SAK_0319	NA	NA	265627	265710	+	84	-
323	SAK_RS01595	SAK_0323	NA	NA	269757	270029	+	273	-
334	SAK_RS01655	SAK_0334	NA	NA	278593	279309	-	717	-
336	SAK_RS01665	SAK_0336	NA	NA	280277	280588	-	312	-
340	SAK_RS01685	SAK_0340	NA	NA	283887	284801	+	915	glyQ
342	SAK_RS01695	SAK_0342	NA	NA	285446	287485	+	2040	glyS
343	SAK_RS01700	SAK_0343	NA	NA	287497	287754	+	258	-
350	SAK_RS01735	SAK_0350	NA	NA	295252	297237	+	1986	tkt
358	SAK_RS01775	SAK_0358	NA	NA	304962	305288	+	327	-
359	SAK_RS01780	SAK_0359	NA	NA	305397	307550	+	2154	pbpX
360	SAK_RS01785	SAK_0360	NA	NA	307552	308562	+	1011	mraY
361	SAK_RS01790	SAK_0361	NA	NA	308660	310003	+	1344	-
363	SAK_RS01800	SAK_0363	NA	NA	311048	311851	+	804	-
364	SAK_RS01805	SAK_0364	NA	NA	311851	312594	+	744	-
366	SAK_RS01815	SAK_0366	NA	NA	313009	313923	+	915	trxB
367	SAK_RS01820	SAK_0367	NA	NA	314081	315541	+	1461	-
368	SAK_RS01825	SAK_0368	NA	NA	315538	316359	+	822	nadE
373	SAK_RS01850	SAK_0373	NA	NA	321398	321730	+	333	-
383	SAK_RS01900	SAK_0383	NA	NA	331680	332309	+	630	gmk
384	SAK_RS01905	SAK_0384	NA	NA	332332	332646	+	315	rpoZ
386	SAK_RS01915	SAK_0386	NA	NA	335157	336092	+	936	fmt
388	SAK_RS01925	SAK_0388	NA	NA	337442	338179	+	738	stp1
392	SAK_RS01945	SAK_0392	NA	NA	341999	342640	+	642	-
394	SAK_RS01955	SAK_0394	NA	NA	344066	344440	+	375	-
398	SAK_RS01975	SAK_0398	NA	NA	347445	347768	+	324	-
399	SAK_RS01980	SAK_0399	NA	NA	347785	348105	+	321	-
409	#N/A	SAK_0409	NA	NA	358663	360169	+	1507	rrsF
410	#N/A	SAK_0410	NA	NA	360259	360331	+	73	-
411	#N/A	SAK_0411	NA	NA	360486	363388	+	2903	rrlF
413	#N/A	SAK_0413	NA	NA	363584	363657	+	74	-

416	SAK_RS02065	SAK_0416	NA	NA	366106	366897	+	792	-
417	SAK_RS02070	SAK_0417	NA	NA	366993	367427	+	435	-
418	SAK_RS02075	SAK_0418	NA	NA	367427	368398	+	972	fabH
419	SAK_RS02080	SAK_0419	NA	NA	368456	368680	+	225	acpP
420	SAK_RS02085	SAK_0420	NA	NA	368835	369794	+	960	fabK
421	SAK_RS02090	SAK_0421	NA	NA	369814	370740	+	927	fabD
422	SAK_RS02095	SAK_0422	NA	NA	370749	371483	+	735	fabG
423	SAK_RS02100	SAK_0423	NA	NA	371499	372731	+	1233	fabF
424	SAK_RS02105	SAK_0424	NA	NA	372733	373233	+	501	accB
425	SAK_RS02110	SAK_0425	NA	NA	373230	373652	+	423	fabZ
426	SAK_RS02115	SAK_0426	NA	NA	373690	375060	+	1371	accC
427	SAK_RS02120	SAK_0427	NA	NA	375069	375944	+	876	accD
428	SAK_RS02125	SAK_0428	NA	NA	375937	376710	+	774	accA
430	SAK_RS02135	SAK_0430	NA	NA	377858	379135	-	1278	serS
440	SAK_RS02185	SAK_0440	NA	NA	387888	388397	+	510	-
448	SAK_RS02230	SAK_0448	NA	NA	393970	394770	+	801	-
451	SAK_RS02245	SAK_0451	NA	NA	395978	396361	+	384	-
452	SAK_RS02250	SAK_0452	NA	NA	396397	397548	+	1152	nusA
453	SAK_RS02255	SAK_0453	NA	NA	397570	397866	+	297	ylxR
454	SAK_RS02260	SAK_0454	NA	NA	397859	398161	+	303	-
456	SAK_RS02270	SAK_0456	NA	NA	401073	401423	+	351	rbfA
471	SAK_RS02345	SAK_0471	NA	NA	416668	416979	+	312	-
474	SAK_RS02360	SAK_0474	NA	NA	418440	418946	+	507	-
479	SAK_RS02390	SAK_0479	NA	NA	423350	424249	-	900	galU
480	SAK_RS02395	SAK_0480	NA	NA	424286	425302	-	1017	gpsA
481	SAK_RS02400	SAK_0481	NA	NA	425472	425801	+	330	rnpA
484	#N/A	SAK_0484	NA	NA	427806	429312	+	1507	rrsG
485	#N/A	SAK_0485	NA	NA	429402	429474	+	73	-
486	#N/A	SAK_0486	NA	NA	429629	432531	+	2903	rrlG
488	#N/A	SAK_0488	NA	NA	432725	432797	+	73	-
489	#N/A	SAK_0489	NA	NA	432803	432873	+	71	-
490	#N/A	SAK_0490	NA	NA	432910	432986	+	77	-
491	#N/A	SAK_0491	NA	NA	432994	433065	+	72	-
492	SAK_RS02455	SAK_0492	NA	NA	433180	433713	-	534	-
507	#N/A	SAK_0507	NA	NA	452511	452633	-	123	-
524	SAK_RS02605	SAK_0524	NA	NA	467448	467897	+	450	-
525	SAK_RS02610	SAK_0525	NA	NA	467916	468194	+	279	-
530	SAK_RS02635	SAK_0530	NA	NA	472341	472625	+	285	-
543	SAK_RS02700	SAK_0543	NA	NA	485251	485505	+	255	-
547	SAK_RS02720	SAK_0547	NA	NA	487482	490136	+	2655	valS
555	SAK_RS02765	SAK_0555	NA	NA	497535	498020	+	486	coaD
565	SAK_RS02815	SAK_0565	NA	NA	508803	509342	-	540	-
577	SAK_RS02875	SAK_0577	NA	NA	518656	520011	+	1356	murD
578	SAK_RS02880	SAK_0578	NA	NA	520014	521090	+	1077	murG
580	SAK_RS02890	SAK_0580	NA	NA	522502	523875	+	1374	ftsA
581	SAK_RS02895	SAK_0581	NA	NA	523897	525177	+	1281	ftsZ
587	SAK_RS02925	SAK_0587	NA	NA	528586	531378	+	2793	ileS
591	SAK_RS02945	SAK_0591	NA	NA	535061	535291	+	231	-
595	SAK_RS02965	SAK_0595	NA	NA	538972	539826	+	855	folD
603	SAK_RS03005	SAK_0603	NA	NA	546253	547089	+	837	-
606	SAK_RS03020	SAK_0606	NA	NA	548601	548876	+	276	hup
610	SAK_RS03040	SAK_0610	NA	NA	550862	551221	-	360	-
612	SAK_RS03050	SAK_0612	NA	NA	551729	552061	+	333	-
614	#N/A	SAK_0614	NA	NA	553227	553376	+	150	-
615	SAK_RS03075	SAK_0615	NA	NA	553649	553939	+	291	-
616	SAK_RS03080	SAK_0616	NA	NA	553926	554609	+	684	-
618	SAK_RS03090	SAK_0618	NA	NA	556053	556535	+	483	-
621	SAK_RS03105	SAK_0621	NA	NA	559264	559545	+	282	-
622	SAK_RS03110	SAK_0622	NA	NA	559624	559920	+	297	-
623	#N/A	SAK_0623	NA	NA	560174	560338	+	165	-
624	#N/A	SAK_0624	NA	NA	560608	560751	+	144	-
625	SAK_RS03125	SAK_0625	NA	NA	560748	561260	+	513	-
626	SAK_RS03130	SAK_0626	NA	NA	561281	561580	+	300	-
627	SAK_RS03135	SAK_0627	NA	NA	561768	561962	+	195	-
640	SAK_RS03200	SAK_0640	NA	NA	570741	571079	+	339	-
641	SAK_RS03210	SAK_0641	NA	NA	571309	571644	+	336	-
643	SAK_RS03220	SAK_0643	NA	NA	572211	572456	+	246	-
644	SAK_RS03225	SAK_0644	NA	NA	572471	572842	+	372	-
649	SAK_RS03250	SAK_0649	NA	NA	582518	582910	+	393	-
650	#N/A	SAK_0650	NA	NA	582933	583061	+	129	-
651	SAK_RS03260	SAK_0651	NA	NA	583070	583372	+	303	-
652	SAK_RS03265	SAK_0652	NA	NA	583365	583592	+	228	-
654	#N/A	SAK_0654	NA	NA	585311	585514	-	204	-
658	SAK_RS03290	SAK_0658	NA	NA	587845	589080	-	1236	fibB
659	SAK_RS03295	SAK_0659	NA	NA	589099	590310	-	1212	-
664	SAK_RS03320	SAK_0664	NA	NA	594548	597232	+	2685	-
668	#N/A	SAK_0668	NA	NA	601503	602601	+	1099	prfB
677	SAK_RS03385	SAK_0677	NA	NA	612097	613443	+	1347	asnC
686	SAK_RS03430	SAK_0686	NA	NA	621789	622049	-	261	rpmE2
689	SAK_RS03445	SAK_0689	NA	NA	624440	624883	+	444	-
692	SAK_RS03460	SAK_0692	NA	NA	627004	627351	+	348	rplS
693	#N/A	SAK_0693	NA	NA	627462	627533	+	72	-
708	SAK_RS03540	SAK_0708	NA	NA	639802	641754	+	1953	gyrB
709	SAK_RS03545	SAK_0709	NA	NA	641848	643572	+	1725	ezrA
713	SAK_RS03565	SAK_0713	NA	NA	645479	646786	+	1308	eno
726	#N/A	SAK_0726	NA	NA	665978	666142	-	165	-
729	SAK_RS03640	SAK_0729	NA	NA	667841	668164	-	324	-
731	#N/A	SAK_0731	NA	NA	670867	670971	+	105	-
732	SAK_RS03650	SAK_0732	NA	NA	671130	671411	+	282	-
736	SAK_RS03665	SAK_0736	NA	NA	673651	674688	+	1038	metK
741	SAK_RS03700	SAK_0741	NA	NA	679165	679578	+	414	-
743	SAK_RS03710	SAK_0743	NA	NA	681223	681525	+	303	-
749	SAK_RS03740	SAK_0749	NA	NA	685399	685656	+	258	-

750	SAK_RS03745	SAK_0750	NA	NA	685656	685994	+	339	-
751	SAK_RS03750	SAK_0751	NA	NA	685987	686355	+	369	-
752	SAK_RS03755	SAK_0752	NA	NA	686364	686690	+	327	-
753	SAK_RS03760	SAK_0753	NA	NA	686693	687262	+	570	-
755	#N/A	SAK_0755	NA	NA	687762	687857	+	96	-
770	#N/A	SAK_0770	NA	NA	706354	706590	-	237	-
783	#N/A	SAK_0783	NA	NA	720824	720916	+	93	-
785	SAK_RS03915	SAK_0785	NA	NA	722922	723191	+	270	-
802	SAK_RS04000	SAK_0802	NA	NA	739493	739834	+	342	-
833	SAK_RS04170	SAK_0833	NA	NA	778052	779056	+	1005	ccpA
836	SAK_RS04185	SAK_0836	NA	NA	781699	783033	+	1335	-
841	SAK_RS04210	SAK_0841	NA	NA	788259	788909	-	651	-
842	SAK_RS04215	SAK_0842	NA	NA	788921	789616	-	696	-
844	SAK_RS04225	SAK_0844	NA	NA	790441	791196	-	756	-
845	SAK_RS04230	SAK_0845	NA	NA	791536	792246	+	711	-
849	SAK_RS04250	SAK_0849	NA	NA	794948	795634	+	687	rnc
861	#N/A	SAK_0861	NA	NA	808574	808708	+	135	-
862	SAK_RS04310	SAK_0862	NA	NA	808824	809759	+	936	hprK
876	SAK_RS04380	SAK_0876	NA	NA	821082	822572	-	1491	lysS
887	SAK_RS04435	SAK_0887	NA	NA	834256	835452	+	1197	tuf
888	SAK_RS04440	SAK_0888	NA	NA	835633	836391	+	759	tpiA
889	SAK_RS04445	SAK_0889	NA	NA	836568	837260	+	693	gpmA
891	SAK_RS04455	SAK_0891	NA	NA	839449	840045	+	597	recR
892	SAK_RS04460	SAK_0892	NA	NA	840186	841232	+	1047	ddl
893	SAK_RS04465	SAK_0893	NA	NA	841379	842746	+	1368	murF
903	SAK_RS04515	SAK_0903	NA	NA	853234	853500	-	267	-
905	SAK_RS04525	SAK_0905	NA	NA	854392	855132	+	741	-
912	SAK_RS04560	SAK_0912	NA	NA	862005	863042	+	1038	holA
934	SAK_RS04670	SAK_0934	NA	NA	885656	888274	+	2619	alaS
937	SAK_RS04685	SAK_0937	NA	NA	890399	890692	+	294	-
942	SAK_RS04710	SAK_0942	NA	NA	894163	895122	-	960	nrdF
943	SAK_RS04715	SAK_0943	NA	NA	895325	897484	-	2160	-
944	SAK_RS04720	SAK_0944	NA	NA	897562	897786	-	225	-
945	SAK_RS04725	SAK_0945	NA	NA	898169	898432	+	264	ptsH
946	SAK_RS04730	SAK_0946	NA	NA	898437	900170	+	1734	ptsI
947	SAK_RS04735	SAK_0947	NA	NA	900320	901747	+	1428	gapN
952	SAK_RS04760	SAK_0952	NA	NA	905613	907277	+	1665	dnaX
954	SAK_RS04775	SAK_0954	NA	NA	908661	909857	+	1197	metK
960	SAK_RS04805	SAK_0960	NA	NA	914768	916054	+	1287	-
969	SAK_RS04850	SAK_0969	NA	NA	923071	923931	+	861	map
973	SAK_RS04870	SAK_0973	NA	NA	926156	928114	+	1959	ligA
980	SAK_RS04905	SAK_0980	NA	NA	937586	937786	+	201	atpE
981	SAK_RS04910	SAK_0981	NA	NA	937819	938535	+	717	atpB
982	SAK_RS04915	SAK_0982	NA	NA	938553	939050	+	498	atpF
983	SAK_RS04920	SAK_0983	NA	NA	939050	939586	+	537	atpH
984	SAK_RS04925	SAK_0984	NA	NA	939602	941107	+	1506	atpA
985	SAK_RS04930	SAK_0985	NA	NA	941123	942004	+	882	atpG
986	SAK_RS04935	SAK_0986	NA	NA	942078	943484	+	1407	atpD
987	SAK_RS04940	SAK_0987	NA	NA	943497	943910	+	414	atpC
992	SAK_RS04965	SAK_0992	NA	NA	946956	947996	+	1041	pheS
994	SAK_RS04975	SAK_0994	NA	NA	948654	951059	+	2406	pheT
999	SAK_RS05000	SAK_0999	NA	NA	960017	961393	-	1377	trmE
1006	SAK_RS05035	SAK_1006	NA	NA	970149	970934	-	786	-
1007	SAK_RS05040	SAK_1007	NA	NA	970934	972277	-	1344	-
1008	SAK_RS05045	SAK_1008	NA	NA	972417	973268	+	852	-
1030	#N/A	SAK_1030	NA	NA	999967	1000313	-	347	-
1034	SAK_RS05175	SAK_1034	NA	NA	1002547	1002915	-	369	-
1035	SAK_RS05180	SAK_1035	NA	NA	1003060	1006164	+	3105	dnaE
1036	SAK_RS05185	SAK_1036	NA	NA	1006245	1007267	+	1023	pfkA
1037	SAK_RS05190	SAK_1037	NA	NA	1007316	1008818	+	1503	pyk
1040	SAK_RS05200	SAK_1040	NA	NA	1009815	1011629	+	1815	glmS
1045	SAK_RS05225	SAK_1045	NA	NA	1014467	1014700	-	234	rpsT
1055	SAK_RS05275	SAK_1055	NA	NA	1024830	1027289	+	2460	gyrA
1056	SAK_RS05280	SAK_1056	NA	NA	1027296	1028039	+	744	-
1057	SAK_RS05285	SAK_1057	NA	NA	1028055	1028468	+	414	-
1062	SAK_RS05310	SAK_1062	NA	NA	1031904	1033466	-	1563	guaA
1069	SAK_RS05345	SAK_1069	NA	NA	1039301	1040053	+	753	-
1076	SAK_RS05380	SAK_1076	NA	NA	1047746	1048432	-	687	satD
1077	SAK_RS05385	SAK_1077	NA	NA	1048499	1050064	-	1566	ffh
1082	SAK_RS05410	SAK_1082	NA	NA	1055337	1055990	-	654	phoU
1084	SAK_RS05420	SAK_1084	NA	NA	1056794	1057597	-	804	pstB
1085	SAK_RS05425	SAK_1085	NA	NA	1057609	1058496	-	888	pstA
1086	SAK_RS05430	SAK_1086	NA	NA	1058486	1059403	-	918	pstC
1087	SAK_RS05435	SAK_1087	NA	NA	1059449	1060309	-	861	-
1092	SAK_RS05460	SAK_1092	NA	NA	1063356	1064288	-	933	ribF
1108	SAK_RS05540	SAK_1108	NA	NA	1080719	1081570	-	852	rbgA
1114	#N/A	SAK_1114	NA	NA	1088185	1088328	-	144	-
1126	SAK_RS05630	SAK_1126	NA	NA	1099325	1099798	-	474	-
1148	SAK_RS05740	SAK_1148	NA	NA	1125893	1126225	-	333	-
1152	SAK_RS05760	SAK_1152	NA	NA	1129405	1130106	+	702	coaB
1155	SAK_RS05775	SAK_1155	NA	NA	1131369	1133087	+	1719	-
1161	SAK_RS05810	SAK_1161	NA	NA	1139760	1140356	-	597	-
1163	SAK_RS05820	SAK_1163	NA	NA	1141179	1142258	-	1080	prfA
1174	SAK_RS05875	SAK_1174	NA	NA	1152058	1152729	+	672	-
1177	SAK_RS05890	SAK_1177	NA	NA	1155758	1156750	-	993	eutD
1179	SAK_RS05900	SAK_1179	NA	NA	1157657	1158484	-	828	ppnK
1185	SAK_RS05930	SAK_1185	NA	NA	1162618	1163250	+	633	-
1197	SAK_RS05990	SAK_1197	NA	NA	1175469	1176371	-	903	murB
1198	SAK_RS05995	SAK_1198	NA	NA	1176515	1177003	-	489	folK
1199	SAK_RS06000	SAK_1199	NA	NA	1177000	1177362	-	363	folB
1200	SAK_RS06005	SAK_1200	NA	NA	1177364	1178167	-	804	folP
1201	SAK_RS06010	SAK_1201	NA	NA	1178171	1178734	-	564	folE
1202	SAK_RS06015	SAK_1202	NA	NA	1178753	1180015	-	1263	folC

1215	SAK_RS06090	SAK_1215	NA	NA	1191853	1192041	+	189	-
1220	SAK_RS06120	SAK_1220	NA	NA	1194743	1195384	-	642	-
1237	SAK_RS06205	SAK_1237	NA	NA	1208516	1209718	-	1203	rpsA
1238	#N/A	SAK_1238	NA	NA	1209841	1209915	-	75	-
1239	#N/A	SAK_1239	NA	NA	1209921	1210001	-	81	-
1243	SAK_RS06235	SAK_1243	NA	NA	1214103	1216031	-	1929	parE
1244	SAK_RS06240	SAK_1244	NA	NA	1216181	1216804	+	624	-
1270	SAK_RS06370	SAK_1270	NA	NA	1240353	1241024	-	672	rpiA
1273	SAK_RS06385	SAK_1273	NA	NA	1242907	1244568	-	1662	-
1285	SAK_RS06445	SAK_1285	NA	NA	1259045	1260091	-	1047	rfbB
1286	SAK_RS06450	SAK_1286	NA	NA	1260298	1260891	-	594	rmlC
1287	SAK_RS06455	SAK_1287	NA	NA	1260891	1261760	-	870	rfbA
1290	SAK_RS06470	SAK_1290	NA	NA	1263709	1264392	-	684	-
1291	SAK_RS06475	SAK_1291	NA	NA	1264496	1265176	-	681	-
1296	SAK_RS06500	SAK_1296	NA	NA	1271609	1272538	-	930	rnz
1314	SAK_RS06590	SAK_1314	NA	NA	1292358	1292648	+	291	-
1315	SAK_RS06595	SAK_1315	NA	NA	1292684	1293463	+	780	-
1322	SAK_RS06640	SAK_1322	NA	NA	1304188	1304478	+	291	-
1323	SAK_RS06645	SAK_1323	NA	NA	1304514	1305275	+	762	-
1334	SAK_RS06715	SAK_1334	NA	NA	1319059	1319424	-	366	rplL
1335	SAK_RS06720	SAK_1335	NA	NA	1319488	1319988	-	501	rplJ
1342	SAK_RS06755	SAK_1342	NA	NA	1327092	1327688	-	597	engB
1345	SAK_RS06770	SAK_1345	NA	NA	1329143	1329637	-	495	folA
1346	SAK_RS06775	SAK_1346	NA	NA	1329717	1330556	-	840	thyA
1347	SAK_RS06780	SAK_1347	NA	NA	1330710	1331882	+	1173	-
1348	SAK_RS06785	SAK_1348	NA	NA	1331884	1333167	+	1284	-
1350	SAK_RS06795	SAK_1350	NA	NA	1333713	1334357	+	645	-
1355	SAK_RS06820	SAK_1355	NA	NA	1337637	1338629	-	993	-
1356	SAK_RS06825	SAK_1356	NA	NA	1338622	1339566	-	945	mvaD
1357	SAK_RS06830	SAK_1357	NA	NA	1339548	1340426	-	879	mvk
1372	SAK_RS06905	SAK_1372	NA	NA	1358095	1359288	-	1194	papS
1384	SAK_RS06965	SAK_1384	NA	NA	1371166	1372158	-	993	-
1385	SAK_RS06970	SAK_1385	NA	NA	1372139	1372894	-	756	trmD
1386	SAK_RS06975	SAK_1386	NA	NA	1372881	1373399	-	519	rimM
1387	#N/A	SAK_1387	NA	NA	1373462	1373533	-	72	-
1390	SAK_RS06995	SAK_1390	NA	NA	1375364	1375606	-	243	-
1391	SAK_RS07000	SAK_1391	NA	NA	1375616	1375888	-	273	rpsP
1400	SAK_RS07045	SAK_1400	NA	NA	1384262	1385167	-	906	-
1401	SAK_RS07050	SAK_1401	NA	NA	1385383	1385676	-	294	rpmA
1402	SAK_RS07055	SAK_1402	NA	NA	1385698	1386036	-	339	-
1403	SAK_RS07060	SAK_1403	NA	NA	1386043	1386357	-	315	rplU
1415	SAK_RS07120	SAK_1415	NA	NA	1399557	1399916	-	360	rplT
1416	SAK_RS07125	SAK_1416	NA	NA	1399974	1400174	-	201	rpmI
1417	SAK_RS07130	SAK_1417	NA	NA	1400214	1400744	-	531	infC
1418	SAK_RS07135	SAK_1418	NA	NA	1400905	1401588	-	684	cmk
1420	SAK_RS07145	SAK_1420	NA	NA	1402169	1402366	+	198	-
1423	SAK_RS07160	SAK_1423	NA	NA	1404228	1405862	-	1635	-
1424	SAK_RS07165	SAK_1424	NA	NA	1405986	1407440	+	1455	murE
1430	SAK_RS07195	SAK_1430	NA	NA	1412127	1413062	-	936	ppaC
1446	SAK_RS07270	SAK_1446	NA	NA	1428862	1429710	-	849	-
1447	SAK_RS07275	SAK_1447	NA	NA	1429707	1431131	-	1425	-
1449	SAK_RS07285	SAK_1449	NA	NA	1432497	1433444	-	948	-
1450	SAK_RS07290	SAK_1450	NA	NA	1433453	1434388	-	936	-
1451	SAK_RS07295	SAK_1451	NA	NA	1434390	1435445	-	1056	-
1452	SAK_RS07300	SAK_1452	NA	NA	1435448	1436167	-	720	ispD
1453	SAK_RS07305	SAK_1453	NA	NA	1436170	1436994	-	825	licD2
1454	SAK_RS07310	SAK_1454	NA	NA	1437019	1438752	-	1734	-
1455	SAK_RS07315	SAK_1455	NA	NA	1438745	1439098	-	354	-
1456	SAK_RS07320	SAK_1456	NA	NA	1439095	1439823	-	729	-
1457	SAK_RS07325	SAK_1457	NA	NA	1439828	1440769	-	942	-
1459	SAK_RS07335	SAK_1459	NA	NA	1442030	1442884	-	855	rfbD
1461	SAK_RS07345	SAK_1461	NA	NA	1443424	1444533	-	1110	rpoD
1462	SAK_RS07350	SAK_1462	NA	NA	1444541	1446322	-	1782	dnaG
1464	SAK_RS07360	SAK_1464	NA	NA	1447013	1447189	-	177	rpsU
1473	SAK_RS07405	SAK_1473	NA	NA	1456970	1458466	-	1497	malQ
1499	SAK_RS07550	SAK_1499	NA	NA	1496083	1496250	-	168	-
1500	SAK_RS07555	SAK_1500	NA	NA	1496276	1497583	-	1308	obgE
1511	SAK_RS07610	SAK_1511	NA	NA	1507879	1508346	-	468	smpB
1513	SAK_RS07620	SAK_1513	NA	NA	1510867	1511103	-	237	secG
1518	SAK_RS07640	SAK_1518	NA	NA	1515119	1515706	-	588	coaE
1522	#N/A	SAK_1522	NA	NA	1519986	1520084	-	99	-
1525	SAK_RS07685	SAK_1525	NA	NA	1521941	1522840	-	900	era
1526	SAK_RS07690	SAK_1526	NA	NA	1522882	1523280	-	399	dgkA
1527	SAK_RS07695	SAK_1527	NA	NA	1523261	1523746	-	486	-
1536	SAK_RS07740	SAK_1536	NA	NA	1531713	1532270	-	558	frr
1537	SAK_RS07745	SAK_1537	NA	NA	1532286	1533014	-	729	pyrH
1543	SAK_RS07775	SAK_1543	NA	NA	1538320	1539009	-	690	rplA
1544	SAK_RS07780	SAK_1544	NA	NA	1539113	1539538	-	426	rplK
1552	SAK_RS07825	SAK_1552	NA	NA	1548743	1551184	-	2442	ftsK
1558	SAK_RS07855	SAK_1558	NA	NA	1555716	1556405	-	690	pfs
1561	SAK_RS07870	SAK_1561	NA	NA	1557259	1558638	-	1380	glmU
1589	SAK_RS08015	SAK_1589	NA	NA	1585037	1585363	-	327	-
1590	SAK_RS08020	SAK_1590	NA	NA	1585393	1586256	-	864	holB
1591	SAK_RS08025	SAK_1591	NA	NA	1586276	1586911	-	636	tmk
1600	SAK_RS08070	SAK_1600	NA	NA	1594301	1594891	-	591	clpP
1615	SAK_RS08145	SAK_1615	NA	NA	1607081	1607875	-	795	murI
1616	SAK_RS08150	SAK_1616	NA	NA	1608050	1608289	-	240	-
1627	SAK_RS08205	SAK_1627	NA	NA	1616433	1616915	-	483	greA
1630	SAK_RS08220	SAK_1630	NA	NA	1619466	1620797	-	1332	murC
1632	#N/A	SAK_1632	NA	NA	1621429	1621527	-	99	-
1634	SAK_RS08235	SAK_1634	NA	NA	1624842	1626152	-	1311	engA
1639	SAK_RS08260	SAK_1639	NA	NA	1630397	1631086	-	690	csrR
1640	SAK_RS08265	SAK_1640	NA	NA	1631321	1631854	-	534	-

1658	SAK_RS08355	SAK_1658	NA	NA	1649585	1650301	-	717	-
1673	SAK_RS08425	SAK_1673	NA	NA	1659563	1660150	-	588	-
1674	SAK_RS08430	SAK_1674	NA	NA	1660147	1660779	-	633	nadD
1677	SAK_RS08445	SAK_1677	NA	NA	1662437	1662970	-	534	-
1680	SAK_RS08460	SAK_1680	NA	NA	1665567	1667033	-	1467	gatA
1681	SAK_RS08465	SAK_1681	NA	NA	1667033	1667335	-	303	gatC
1696	SAK_RS08540	SAK_1696	NA	NA	1684669	1685769	-	1101	alr
1697	SAK_RS08545	SAK_1697	NA	NA	1685766	1686125	-	360	acpS
1703	SAK_RS08575	SAK_1703	NA	NA	1694038	1695477	+	1440	scrB
1707	SAK_RS08595	SAK_1707	NA	NA	1697433	1697993	-	561	efp
1719	SAK_RS08645	SAK_1719	NA	NA	1708946	1709890	-	945	-
1720	SAK_RS08650	SAK_1720	NA	NA	1710060	1710299	-	240	rpsR
1721	SAK_RS08655	SAK_1721	NA	NA	1710344	1710835	-	492	ssb1
1722	SAK_RS08660	SAK_1722	NA	NA	1710847	1711134	-	288	rpsF
1725	SAK_RS08675	SAK_1725	NA	NA	1714168	1714482	-	315	trx
1730	SAK_RS08700	SAK_1730	NA	NA	1718458	1719351	+	894	rnhC
1731	SAK_RS08705	SAK_1731	NA	NA	1719367	1719960	+	594	lepB
1741	SAK_RS08755	SAK_1741	NA	NA	1731076	1731528	-	453	-
1761	SAK_RS08855	SAK_1761	NA	NA	1756316	1756627	-	312	-
1769	#N/A	SAK_1769	NA	NA	1763970	1764068	-	99	-
1779	SAK_RS08955	SAK_1779	NA	NA	1772296	1773306	-	1011	gcp
1781	SAK_RS08965	SAK_1781	NA	NA	1773791	1774483	-	693	-
1783	SAK_RS08975	SAK_1783	NA	NA	1774946	1776625	+	1680	-
1785	SAK_RS08985	SAK_1785	NA	NA	1777444	1778790	-	1347	glnA
1786	SAK_RS08990	SAK_1786	NA	NA	1778824	1779195	-	372	glnR
1788	SAK_RS09000	SAK_1788	NA	NA	1780077	1781273	-	1197	pgk
1790	SAK_RS09010	SAK_1790	NA	NA	1782486	1783496	-	1011	gap
1791	SAK_RS09015	SAK_1791	NA	NA	1783701	1785779	-	2079	fusA
1792	SAK_RS09020	SAK_1792	NA	NA	1785934	1786404	-	471	rpsG
1797	SAK_RS09045	SAK_1797	NA	NA	1790174	1790806	-	633	-
1798	SAK_RS09050	SAK_1798	NA	NA	1790799	1791461	-	663	rpe
1799	SAK_RS09055	SAK_1799	NA	NA	1791468	1792340	-	873	-
1810	SAK_RS09110	SAK_1810	NA	NA	1800009	1800248	-	240	dltC
1811	SAK_RS09115	SAK_1811	NA	NA	1800263	1801528	-	1266	dltB
1812	SAK_RS09120	SAK_1812	NA	NA	1801525	1803060	-	1536	dltA
1815	SAK_RS09140	SAK_1815	NA	NA	1805423	1805557	-	135	rpmH
1829	SAK_RS09210	SAK_1829	NA	NA	1824608	1825255	-	648	-
1833	SAK_RS09230	SAK_1833	NA	NA	1827629	1828114	-	486	-
1834	SAK_RS09235	SAK_1834	NA	NA	1828181	1828459	-	279	-
1851	SAK_RS09320	SAK_1851	NA	NA	1846869	1847909	-	1041	tsf
1852	SAK_RS09325	SAK_1852	NA	NA	1848003	1848773	-	771	rpsB
1855	#N/A	SAK_1855	NA	NA	1851295	1851365	+	71	-
1863	SAK_RS09380	SAK_1863	NA	NA	1859960	1860574	+	615	def
1866	SAK_RS09395	SAK_1866	NA	NA	1862739	1862987	+	249	-
1869	SAK_RS09410	SAK_1869	NA	NA	1864458	1868864	-	4407	polC
1871	SAK_RS09420	SAK_1871	NA	NA	1869696	1871549	-	1854	proS
1873	SAK_RS09430	SAK_1873	NA	NA	1872931	1873725	-	795	cdsA
1874	SAK_RS09435	SAK_1874	NA	NA	1873740	1874492	-	753	uppS
1882	SAK_RS09475	SAK_1882	NA	NA	1882082	1883077	-	996	galE
1902	SAK_RS09575	SAK_1902	NA	NA	1906662	1907117	+	456	nrdI
1916	SAK_RS09650	SAK_1916	NA	NA	1919126	1919308	+	183	-
1939	#N/A	SAK_1939	NA	NA	1939018	1939093	+	76	-
1942	SAK_RS09780	SAK_1942	NA	NA	1940873	1941013	-	141	-
1944	SAK_RS09790	SAK_1944	NA	NA	1942170	1942355	-	186	-
1947	#N/A	SAK_1947	NA	NA	1944192	1944365	-	174	-
1955	#N/A	SAK_1955	NA	NA	1950930	1951058	+	129	-
1958	#N/A	SAK_1958	NA	NA	1952333	1952458	+	126	-
1960	#N/A	SAK_1960	NA	NA	1952910	1953014	+	105	-
1995	SAK_RS10040	SAK_1995	NA	NA	1987441	1989942	-	2502	leuS
1998	SAK_RS10055	SAK_1998	NA	NA	1992592	1993131	-	540	nusG
2003	SAK_RS10080	SAK_2003	NA	NA	1996768	1996941	-	174	secE
2004	#N/A	SAK_2004	NA	NA	1996977	1997123	-	147	rpmGB
2014	SAK_RS10135	SAK_2014	NA	NA	2009267	2009551	-	285	groES
2027	SAK_RS10200	SAK_2027	NA	NA	2022083	2022205	-	123	-
2029	SAK_RS10210	SAK_2029	NA	NA	2022631	2023041	-	411	-
2035	SAK_RS10240	SAK_2035	NA	NA	2027267	2027857	-	591	ruvA
2038	#N/A	SAK_2038	NA	NA	2031227	2031328	-	102	-
2042	SAK_RS10270	SAK_2042	NA	NA	2034874	2036565	+	1692	argS
2046	SAK_RS10290	SAK_2046	NA	NA	2038902	2040653	-	1752	aspS
2047	SAK_RS10295	SAK_2047	NA	NA	2040746	2042026	-	1281	hisS
2048	SAK_RS10300	SAK_2048	NA	NA	2042246	2042428	+	183	rpmF
2049	SAK_RS10305	SAK_2049	NA	NA	2042444	2042593	+	150	rpmG
2050	SAK_RS10310	SAK_2050	NA	NA	2042762	2043970	-	1209	-
2052	SAK_RS10320	SAK_2052	NA	NA	2044850	2045188	+	339	cadX
2085	SAK_RS10490	SAK_2085	NA	NA	2078116	2078445	-	330	-
2086	SAK_RS10500	SAK_2086	NA	NA	2078648	2078980	-	333	-
2088	SAK_RS10510	SAK_2088	NA	NA	2079662	2079835	-	174	-
2091	SAK_RS10535	SAK_2091	NA	NA	2082243	2082857	+	615	-
2092	#N/A	SAK_2092	NA	NA	2083250	2083354	-	105	-
2095	SAK_RS10555	SAK_2095	NA	NA	2086271	2086882	-	612	rpsD
2096	SAK_RS10560	SAK_2096	NA	NA	2087212	2087499	-	288	-
2100	SAK_RS10580	SAK_2100	NA	NA	2091416	2093317	-	1902	gidA
2102	SAK_RS10590	SAK_2102	NA	NA	2094130	2095251	-	1122	mnmA
2105	SAK_RS10605	SAK_2105	NA	NA	2097187	2097864	-	678	-
2107	SAK_RS10615	SAK_2107	NA	NA	2098752	2099546	-	795	-
2108	SAK_RS10620	SAK_2108	NA	NA	2099539	2100381	-	843	cbiO
2109	SAK_RS10625	SAK_2109	NA	NA	2100357	2101196	-	840	cbiO
2110	SAK_RS10630	SAK_2110	NA	NA	2101196	2101738	-	543	pgsA
2131	#N/A	SAK_2131	NA	NA	2124438	2124511	-	74	-
2132	#N/A	SAK_2132	NA	NA	2124541	2124612	-	72	-
2133	#N/A	SAK_2133	NA	NA	2124805	2124878	+	74	-

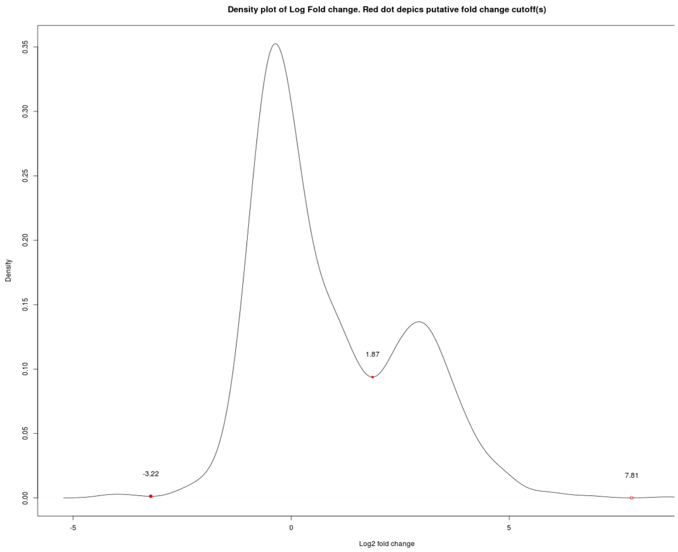
Density plot of Log Fold change. Red dot depics putative fold change cutoff(s)
Density
0.00
0.05
0.10
0.15
0.20
0.25
0.30
0.35
-3.22
1.87
7.81
-5
0
5
Log2 fold change
