# Supplementary material for: Genome-Wide fitness analysis of group B Streptococcus in human amniotic fluid reveals a transcription factor that controls multiple virulence traits
Source: PLoS Pathog. 2021 Mar 8;17(3):e1009116. doi: 10.1371/journal.ppat.1009116 (PMC7971860; doi:10.1371/journal.ppat.1009116)
Supplement: S2 Data — Output from DESeq 2 analysis of RNA-seq read alignments. Log2 fold-change and adjusted p values are provided for three growth phases as described in the text. (PDF) [file ppat.1009116.s002.pdf]

	new_locus_tag	old_locus_ta	start	end	name	description	log2FoldChange_earlyLog	padj_EarlyLog	log2FoldChange_lateLog	padj_lateLog	log2FoldChange_stat	padj_stat
1	W903_RS00005	W903_0001	54	1463	dnaA	chromosomal replication initiator protein DnaA	-0.192067193	0.272179322	-0.30511719	0.007573575	0.383229911	0.00414485
2	W903_RS00010	W903_0002	1618	2754	dnaN	DNA polymerase III, beta subunit	-0.088169456	0.629611169	-0.856579541	6.62975E-13	0.72121152	0.000000474
3	W903_RS00015	W903_0003	2812	3705	NA	diacylglycerol kinase catalytic domain protein	-0.806137191	8.1301E-07	0.391108678	0.005227457	-0.986466186	3.89E-08
4	W903_RS00020	W903_0004	3715	3912	NA	hypothetical protein	-1.31569835	9.20913E-13	-0.259427933	0.100153884	-1.062289874	0.00000691
5	NA	W903_0005	4037	4177	NA	helix-turn-helix family protein	-0.152996563	0.566833212	0.275656155	0.098943389	-1.05548621	0.00017744
6	W903_RS00030	W903_0006	4535	5650	NA	50S ribosome-binding GTPase family protein	-0.547306916	0.001019086	-1.170317955	7.36474E-32	0.091967435	0.610156915
7	W903_RS00035	W903_0007	5734	6309	pth	peptidyl-tRNA hydrolase	-0.648196454	0.006565025	-1.397500868	1.65455E-21	0.001436765	0.996252226
8	W903_RS00040	W903_0008	6306	9803	mfd	transcription-repair coupling factor	-0.812046428	2.16187E-05	-1.812506544	2.81494E-54	0.015987224	0.934056998
9	W903_RS00045	W903_0009	10094	10366	NA	S4 domain protein	-0.931606411	1.20706E-06	-1.075244929	1.19573E-14	0.228132981	0.500564049
10	W903_RS00050	W903_0010	10353	10724	NA	septum formation initiator family protein	-0.812343739	8.9586E-07	-0.726275204	1.67237E-08	0.044395388	0.85007182
11	W903_RS00055	W903_0011	10727	10861	NA	hypothetical protein	-1.457177723	3.378E-13	-1.043231159	1.15849E-08	0.070149016	0.868645392
12	W903_RS00060	W903_0012	10861	12147	NA	D-alanyl-D-alanine carboxypeptidase family protein	-0.899765758	1.17178E-08	-0.732746049	1.42937E-14	-0.297931201	0.099392439
13	W903_RS00065	W903_0013	12149	13423	tilS	tRNA(Ile)-lysidine synthetase	-0.595875518	5.69449E-05	0.112876081	0.276457054	-0.128087655	0.415784043
14	W903_RS00070	W903_0014	13428	13970	hpt	hypoxanthine phosphoribosyltransferase	0.1156808	0.503809141	-0.964722774	5.17484E-25	0.49978629	0.002139059
15	W903_RS00075	W903_0015	13993	15969	hflB	ATP-dependent metallopeptidase HflB family protein	0.566663928	0.000108051	-0.624648825	9.26956E-12	-0.052547932	0.750481516
16	W903_RS00080	W903_0016	16467	18003	rRNA	NA	2.410228946	0.376532116	-4.336131983	0.025327637	-0.680855949	0.71250481
17	W903_RS00085	W903_0017	18063	18135	rRNA	NA	-0.902731633	NA	-0.053706497	0.960968817	2.040378825	0.355180694
18	W903_RS00090	W903_0018	18292	21192	rRNA	NA	2.788501858	0.238231645	-4.49089962	0.024708989	0.145546075	0.926016995
19	W903_RS00095	W903_0019	21270	21383	rRNA	NA	-1.530553442	0.014692636	1.291313578	0.011584324	0.036262732	0.926016995
20	W903_RS00100	W903_0020	21386	21458	rRNA	NA	-0.008981392	NA	-1.412224254	0.550598186	2.762962331	0.354847058
21	W903_RS00105	W903_0021	21461	21533	rRNA	NA	-1.296189788	NA	0.21618932	0.882784086	4.579295302	0.094856967
22	W903_RS00110	W903_0022	21536	21608	rRNA	NA	-0.815028368	NA	1.375629996	0.368438014	3.936890559	0.068662702
23	W903_RS00115	W903_0023	21618	21699	NA	NA	NA	NA	1.048062688	0.86167603	1.876910281	0.77240887
24	W903_RS00120	W903_0024	21710	21782	rRNA	NA	-0.7358948	NA	-0.082981902	0.971479693	6.168457092	0.002016583
25	W903_RS00125	W903_0025	21816	21887	rRNA	NA	-0.834842062	NA	0.158238201	0.856110387	7.168086863	0.0000545
26	W903_RS00130	W903_0026	21896	21980	rRNA	NA	-0.517943311	NA	-0.08399439	0.945066339	0.426314479	0.65071206
27	W903_RS00135	W903_0027	21992	22065	tRNA	NA	-2.599052178	NA	2.130341804	0.443677967	4.84666148	0.061128759
28	W903_RS00140	W903_0028	22073	22146	rRNA	NA	0.707266024	NA	1.317858829	0.055468389	3.422406647	1.62E-16
29	W903_RS00145	W903_0029	22298	23834	rRNA	NA	2.403759877	0.377928802	-4.329608987	0.025957193	-0.688266293	0.710624211
30	W903_RS00150	W903_0030	23894	23966	rRNA	NA	-0.829079144	NA	-0.411633831	0.749853298	2.828783735	0.154492289
31	W903_RS00155	W903_0031	24123	27023	rRNA	NA	2.789290799	0.238231645	-4.46934484	0.025180409	0.14796923	0.925877523
32	W903_RS00160	W903_0032	27101	27214	rRNA	NA	-0.816388837	0.260142929	1.089765495	0.037412149	-0.022101721	0.95113423
33	W903_RS00165	W903_0033	27217	27289	tRNA	NA	-2.059390653	NA	1.833398181	0.400871853	2.847571588	0.305814721
34	W903_RS00170	W903_0034	27292	27364	rRNA	NA	-1.228152108	NA	0.596972864	0.765232568	5.377579846	0.020632542
35	W903_RS00175	W903_0035	27367	27439	rRNA	NA	-0.679289454	NA	0.817416983	0.496498369	6.864878324	0.000155506
36	W903_RS00180	W903_0036	27449	27530	rRNA	NA	1.170348617	NA	-1.943554221	0.737862841	NA	NA
37	W903_RS00185	W903_0037	27541	27613	rRNA	NA	-1.095681031	NA	1.470412164	0.420397259	3.708851248	0.024549784
38	W903_RS00190	W903_0038	27647	27718	rRNA	NA	-1.095309805	NA	0.639745024	0.384515144	6.979758964	0.00012804
39	W903_RS00195	W903_0039	27727	27811	rRNA	NA	0.018741414	NA	0.300571073	0.807643153	0.478027573	0.637429491
40	W903_RS00200	W903_0040	27823	27896	rRNA	NA	-0.088381731	NA	-0.364998529	0.946331025	4.281048951	0.0351132
41	W903_RS00205	W903_0041	27904	27977	rRNA	NA	-0.257947718	NA	0.910395492	0.402091417	4.6375439	0.000849281
42	W903_RS00210	W903_0042	27993	28066	rRNA	NA	-0.053834414	NA	0.979450433	0.627271987	6.882750202	0.000242294
43	W903_RS00215	W903_0043	28086	28159	rRNA	NA	-0.56930183	0.435886345	1.185054344	0.051335926	7.353616404	0.00000202
44	W903_RS00220	W903_0044	28175	28264	rRNA	NA	-0.284965672	0.733854288	0.636213859	0.401409142	5.695704732	2.63E-08
45	W903_RS00225	W903_0045	28275	28348	rRNA	NA	-1.500724356	NA	2.731406411	0.275636328	4.097697152	0.051982704
46	W903_RS00230	W903_0046	28351	28423	tRNA	NA	-1.98032567	NA	1.450158491	0.453048129	3.665156557	0.051982704
47	W903_RS00235	W903_0047	28443	28513	tRNA	NA	-3.751729086	NA	2.731406411	0.275636328	6.742564846	0.000278584
48	W903_RS00240	W903_0048	28548	28621	rRNA	NA	-1.705053102	NA	1.909600748	0.341223627	6.863397158	0.000155399
49	W903_RS00245	W903_0049	28635	28722	tRNA	NA	-4.136760643	NA	-0.040787376	0.986162366	1.661371839	0.194723208
50	W903_RS00250	W903_0050	28958	30295	NA	CHAP domain protein	-1.270039514	3.50543E-17	-0.946475236	1.14662E-26	-1.272645579	4.13E-26
51	W903_RS00255	W903_0051	30419	31387	prs	ribose-phosphate diphosphokinase family protein	-0.106069267	0.536398445	-0.015310527	0.8830912	0.123119056	0.408985759
52	W903_RS00260	W903_0052	31495	32670	NA	aminotransferase class-V family protein	0.022672516	0.909836741	-0.237594713	0.015101179	0.837912502	0.0000153
53	W903_RS00265	W903_0053	32660	33421	recO	DNA repair protein RecO	-0.346842884	0.058233186	-0.233046788	0.010470242	0.412876959	0.031728568
54	W903_RS00270	W903_0054	33484	34362	NA	CAAX protease self-immunity family protein	-0.339024387	0.365184946	-0.912893172	9.16251E-05	-0.607992699	0.14471533
55	W903_RS00275	W903_0055	34440	35432	plsX	fatty acid/phospholipid synthesis protein PlsX	-0.107411442	0.554005697	-0.43391214	8.80647E-05	0.996730258	4.44E-13
56	W903_RS00280	W903_0056	35443	35682	NA	phosphopantetheine attachment site family protein	0.942089407	1.15664E-09	-0.064272295	0.550454629	1.067335979	1.49E-12
57	W903_RS00285	W903_0057	35806	36510	purC	phosphoribosylaminoimidazolesuccinocarboxamide synthase	0.411949036	0.446374739	-2.389249605	1.03297E-26	-0.363166805	0.427394265
58	NA	W903_0058	36527	36640	NA	phosphoribosylformylglycinamidine synthase domain protein	-0.21814021	NA	-3.092522068	2.00702E-06	-0.180628404	0.926904618
59	W903_RS00290	W903_0059	36633	40358	NA	phosphoribosylformylglycinamidine synthase	1.167106317	2.44698E-06	-2.858703217	1.9143E-131	0.384796924	0.038309835
60	W903_RS00295	W903_0060	40398	40730	NA	glycerophosphoryl diester phosphodiesterase family protein	0.886425574	0.021623854	-2.910061941	5.06554E-53	0.732840797	0.006017318
61	W903_RS00300	W903_0061	40880	42334	purF	amidophosphoribosyltransferase	1.654871277	9.53326E-14	-2.795438952	2.8787E-113	0.509462215	0.005921543
62	W903_RS00305	W903_0062	42362	43384	purM	phosphoribosylformylglycinamidine cyclo-ligase	1.606658336	8.10917E-09	-2.801255502	3.60047E-63	0.323488557	0.088774933
63	W903_RS00310	W903_0063	43552	44100	purN	phosphoribosylglycinamide formyltransferase	1.880321467	3.28879E-11	-3.063149939	8.15491E-79	0.662653456	0.004497562
64	W903_RS00315	W903_0064	44123	44875	NA	acetyltransferase family protein	1.12142573	2.14802E-09	-2.347797941	1.9575E-108	0.626922122	0.00013174
65	W903_RS00320	W903_0065	44895	46442	purH	phosphoribosylaminoimidazolecarboxamide formyltransferase/IMP cyclohydrolase	1.368327432	1.22792E-14	-2.191444446	6.85435E-98	0.465883942	0.004365216
66	W903_RS00325	W903_0066	46635	47534	NA	peptidase M23 family protein	0.641402587	0.031426788	1.592106764	4.29666E-12	0.739924615	0.010264349
67	W903_RS00330	W903_0067	47681	48991	NA	lysM domain protein	-1.087298588	2.2691E-13	0.17465421	0.052700956	-1.238120491	2.63E-23
68	W903_RS00335	W903_0068	49238	49936	NA	putative N-acetylmannosamine-6-phosphate 2-epimerase	-0.184621958	0.735544957	0.904653999	0.000484969	-0.779720813	0.000703019
69	W903_RS00340	W903_0069	49983	51299	NA	bacterial extracellular solute-binding family protein	0.050958373	0.932692932	1.496473303	1.02642E-07	-0.988392009	3.19E-09
70	W903_RS00345	W903_0070	51387	52274	NA	binding--dependent transport system inner membrane component family protein	0.34256847	0.674964304	2.8739743	7.97891E-14	-1.109613939	0.000126326
71	W903_RS00350	W903_0071	52284	53114	NA	binding--dependent transport system inner membrane component family protein	0.386466145	0.641226111	2.613852952	9.39116E-12	-1.094941611	0.000925172
72	W903_RS00355	W903_0072	53127	53570	NA	hypothetical protein	0.564434474	0.484301603	1.148142693	0.013645992	-0.362388163	0.407447427
73	W903_RS00360	W903_0073	53590	54252	NA	hypothetical protein	-0.265296111	NA	2.039946432	1.83371E-05	-0.930125161	0.016821368
74	W903_RS00365	W903_0074	54249	55166	NA	dihydrodipicolinate synthetase family protein	0.853048963	0.187598324	1.51316635	0.000104185	-0.537961242	0.081179131
75	W903_RS00370	W903_0075	55183	56064	NA	ROK family protein	0.366956753	0.479805785	1.239508771	4.36243E-06	-0.475780841	0.054557738
76	W903_RS00375	W903_0076	56072	57049	axe1	xylan esterase 1	1.154567504	0.023352457	1.425857305	5.51175E-05	-0.318336998	0.30433167
77	W903_RS00380	W903_0077	57072	57875	NA	helix-turn-helix domain, rpiR family protein	-0.391298211	0.125079485	0.241257671	0.244943932	-0.709741471	0.0000903
78	W903_RS00385	W903_0078	58157	59419	purD	phosphoribosylamine--glycine ligase	1.941536917	1.97424E-20	-1.784649822	4.90521E-21	-0.222011271	0.31232465
79	W903_RS00390	W903_0079	60005	60493	purE	phosphoribosylaminoimidazole carboxylase, catalytic subunit	2.286587867	7.23174E-10	-1.181116753	1.27614E-05	-0.218220959	0.47604716
80	W903_RS00395	W903_0080	60480	61553	purK	phosphoribosylaminoimidazole carboxylase, ATPase subunit	1.944486874	1.16308E-25	-1.684198176	4.6227E-28	-0.077826322	0.690797222
81	W903_RS00400	W903_0081	61563	61820	NA	putative phosphoribosylaminoimidazole carboxylase ATPase subunit	0.351526065	0.213132413	-0.915264089	3.94738E-06	-0.223543369	0.370300334
82	W903_RS00405	W903_0082	62038	63288	NA	hypothetical protein	0.916899444	3.5071E-10	-0.27064574	0.002969583	-0.313463202	0.014127404
83	W903_RS00410	W903_0083	63374	64672	purB	adenylosuccinate lyase	0.961892979	3.90051E-11	-0.625305584	2.39043E-08	-0.071155742	0.655835799
84	W903_RS00415	W903_0084	64825	65736	NA	helix-turn-helix family protein	-1.133285473	3.08363E-11	-0.379904103	0.000692856	0.074075531	0.706454209
85	W903_RS00420	W903_0085	66033	67031	ruvB	holliday junction DNA helicase RuvB	-0.763387666	2.25231E-05	0.441438706	2.44039E-05	-0.420139977	0.005712937
86	W903_RS00425	W903_0086	67183	67620	NA	low molecular weight phosphotyrosine phosphatase family protein	-0.281236968	0.111988161	0.063854486	0.639063401	-0.186376007	0.251463744
87	W903_RS00430	W903_0087	67627	68007	NA	MORN repeat family protein	0.125206289	0.494839205	0.409891799	2.4831E-05	-0.121464695	0.390873083
88	W903_RS00435	W903_0088	68004	69782	NA	acyltransferase family protein	0.03928857	0.825599533	-0.006184993	0.955409198	-0.263043707	0.033895256
89	W903_RS00440	W903_0089	70052	72694	NA	aldehyde dehydrogenase family protein	0.091241701	0.717318267	1.889851898	1.11099E-30	0.096001204	0.49024491
90	W903_RS00445	W903_0090	72873	73889	NA	zinc-binding dehydrogenase family protein	-0.088017238	0.642192846	1.137431006	2.29547E-18	0.36040944	0.002832272
91	W903_RS00450	W903_0091	74009	75499	thrC	threonine synthase	0.212193945	0.240481793	-0.421350922	0.000280243	0.047620545	0.755876193
92	W903_RS00455	W903_0092	75604	76884	NA	MATE efflux family protein	0.271944086	0.100497184	0.208739721	0.05391016	-0.142972003	0.322913466
93	W903_RS00460	W903_0093	77090	77398	rpsJ	ribosomal protein S10	-0.091072933	0.624569854	-1.597909489	1.86253E-31	0.099755408	0.643102615
94	W903_RS00465	W903_0094	77503	78129	rplC	50S ribosomal protein L3	0.270709549	0.123894898	-1.36886168	1.59598E-22	-0.015132019	0.933895471
95	W903_RS00470	W903_0095	78153	78776	rplD	50S ribosomal protein L4	0.285810258	0.069411897	-1.307750072	8.65618E-26	-0.156866753	0.242128369
96	W903_RS00475	W903_0096	78776	79072	rplW	50S ribosomal protein L23	0.000499452	0.998097116	-1.449227018	2.33608E-20	-0.053479264	0.724791933
97	W903_RS00480	W903_0097	79090	79923	rplB	ribosomal protein L2	0.582015638	0.000116877	-1.167571401	4.04429E-21	-0.232998449	0.146967137
98	W903_RS00485	W903_0098	80022	80300	rpsS	ribosomal protein S19	0.502299316	0.004164815	-1.306616512	4.92072E-16	-0.065520925	0.71250481
99	W903_RS00490	W903_0099	80316	80660	rplV	ribosomal protein L22	0.469094721	0.002162424	-1.245454617	5.24886E-16	-0.000515357	0.997381444
100	W903_RS00495	W903_0100	80673	81326	rpsC	ribosomal protein S3	1.044675776	2.19789E-14	-0.938762656	4.13357E-12	-0.160165582	0.228903118
101	W903_RS00500	W903_0101	81330	81743	rplP	ribosomal protein L16	1.135350431	5.58782E-16	-0.934759465	3.7845E-17	-0.148036423	0.345521261
102	W903_RS00505	W903_0102	81753	81959	rpmC	ribosomal protein L29	0.635784192	9.02419E-06	-1.374764074	6.67953E-21	-0.194767244	0.202099681
103	W903_RS00510	W903_0103	81985	82245	rpsQ	30S ribosomal protein S17	1.066145609	4.10223E-14	-1.153399068	3.11651E-19	-0.273143425	0.06663029
104	W903_RS00515	W903_0104	82270	82638	rplN	ribosomal protein L14	0.991780954	4.60533E-11	-1.180780036	4.42373E-27	-0.474973359	0.000849281
105	W903_RS00520	W903_0105	82718	83023	rplX	ribosomal protein L24	1.208729824	3.50543E-17	-1.133261732	5.64714E-25	-0.481272373	0.000841273
106	W903_RS00525	W903_0106	83047	83589	rplE	50S ribosomal protein L5	0.856437639	3.87167E-09	-1.173170007	2.01199E-14	-0.372759633	0.003129468
107	W903_RS00530	W903_0107	83607	83792	rpsZ	30S ribosomal protein S14 type Z	0.478816266	0.012507613	-1.54102174	1.27323E-22	-0.517666439	0.0000766
108	W903_RS00535	W903_0108	83947	84345	rpsH	30S ribosomal protein S8	0.394070154	0.041698478	-1.451123341	3.00829E-18	-0.324904154	0.008348801
109	W903_RS00540	W903_0109	84455	84991	rplF	ribosomal protein L6	0.672410937	0.000197833	-1.474231564	2.1836E-35	-0.456216146	0.000706369
110	W903_RS00545	W903_0110	85092	85448	rplR	ribosomal protein L18	0.936234401	8.44101E-09	-1.168946797	1.37604E-25	-0.652692558	0.00000433
111	W903_RS00550	W903_0111	85467	85961	rpsE	ribosomal protein S5	1.120680619	5.50993E-14	-1.079756926	8.85838E-15	-0.544143354	0.000127231
112	W903_RS00555	W903_0112	85976	86155	rpmD	ribosomal protein L30	0.375931442	0.036890805	-1.700265953	1.93102E-21	-0.270603564	0.032597827
113	W903_RS00560	W903_0113	86280	86720	rplO	ribosomal protein L15	0.802834183	1.65981E-08	-1.353079492	8.29163E-16	-0.169234161	0.219907298
114	W903_RS00565	W903_0114	86741	88045	secY	preprotein translocase, SecY subunit	0.685891681	8.19376E-07	-1.470320624	1.05527E-25	-0.109664808	0.459271611
115	W903_RS00570	W903_0115	88140	88778	adk	adenylate kinase	-1.155177667	3.36045E-10	-1.78134431	2.25015E-36	-0.503456252	0.00080407
116	W903_RS00575	W903_0116	88894	89112	infA	translation initiation factor IF-1	-0.897309205	2.73892E-06	-1.265415532	1.46442E-49	0.017812736	0.918801321
117	W903_RS10205	W903_0117	89138	89254	rpmJ	ribosomal protein L36	0.178286822	0.387696786	-0.556024268	1.5094E-08	-0.133318653	0.457213502
118	W903_RS00580	W903_0118	89272	89637	rpsM	30S ribosomal protein S13	-0.644901309	0.003758254	-0.994480003	1.95817E-31	-0.166095595	0.295440105
119	W903_RS00585	W903_0119	89655	90038	rpsK	30S ribosomal protein S11	-0.1035415	0.568710442	-0.490957213	1.11683E-06	-0.60559367	0.0000368
120	W903_RS00590	W903_0120	90088	91026	rpoA	DNA-directed RNA polymerase, alpha subunit	-0.550420971	0.000587297	-0.41762386	9.16559E-06	-0.45537802	0.000385867
121	W903_RS00595	W903_0121	91041	91427	rplQ	ribosomal protein L17	-0.475642392	0.003955895	-0.169788124	0.070211636	-0.836575789	8.46E-09
122	W903_RS00600	W903_0122	92021	93557	rRNA	NA	2.395692532	0.295806045	-4.317340309	0.026629559	-0.696113738	0.706454209
123	W903_RS00605	W903_0123	93617	93689	rRNA	NA	-0.584801729	0.509464899	1.791107509	0.164699683	1.721440572	0.358121165
124	W903_RS00610	W903_0124	93846	96746	rRNA	NA	2.788151164	0.238231645	-4.489222951	0.024498859	0.145398326	0.926016995
125	W903_RS00615	W903_0125	96824	96937	rRNA	NA	-1.144082958	0.075629973	1.228334097	0.03701817	-0.044798606	0.904836992
126	W903_RS00620	W903_0126	96940	97012	tRNA	NA	-2.236685848	NA	1.333788115	0.608364086	4.136862744	0.189732569
127	W903_RS00625	W903_0127	97015	97087	rRNA	NA	-1.536025336	NA	1.784342926	0.289883319	4.482767294	0.11221854
128	W903_RS00630	W903_0128	97090	97162	rRNA	NA	-0.905761953	NA	1.095913821	0.413819862	2.839054549	0.10148024
129	W903_RS00635	W903_0129	97172	97253	rRNA	NA	-0.968406418	NA	NA	NA	NA	NA
130	W903_RS00640	W903_0130	97264	97336	rRNA	NA	-0.260110015	NA	1.317599062	0.388727127	3.702380774	0.022137
131	W903_RS00645	W903_0131	97365	97441	rRNA	NA	-0.386621625	0.641226111	0.118167919	0.888036667	5.317133987	0.000000637
132	W903_RS00650	W903_0132	97449	97520	rRNA	NA	0.498974692	NA	1.01511388	0.865322464	5.063156759	0.038785797
133	W903_RS00655	W903_0133	97534	97628	NA	NA	NA	NA	NA	NA	NA	NA

134	W903_RS00660	W903_0134	97639	97712	rRNA	NA	-1.533667816	NA	1.083226579	0.28974592	5.255882313	0.0000867
135	W903_RS00665	W903_0135	97715	97787	rRNA	NA	-0.07327521	NA	1.209131335	0.209207889	6.318413123	0.001109156
136	W903_RS00670	W903_0136	97799	97879	rRNA	NA	0.60090339	NA	0.472748079	0.581516414	8.024405991	0.00000146
137	W903_RS00675	W903_0137	97886	97956	rRNA	NA	-0.51151837	0.560572697	-1.41388743	0.087813097	5.923523669	0.00553976
138	W903_RS00680	W903_0139	97971	98043	NA	NA	NA	NA	NA	NA	NA	NA
139	W903_RS00685	W903_0140	98050	98121	rRNA	NA	0.778040693	NA	-0.395632635	0.943602143	NA	NA
140	W903_RS00690	W903_0141	98132	98215	tRNA	NA	2.193392017	NA	NA	NA	NA	NA
141	W903_RS10745	W903_0142	98697	99062	NA	putative lipoprotein BUG3	0.153021213	0.670231561	0.321349463	0.237011352	0.025185647	0.965445709
142	W903_RS00700	W903_0143	99132	99335	NA	hypothetical protein	-0.865655952	0.015422106	-0.208911871	0.547197575	0.285483362	0.706454209
143	W903_RS00705	W903_0144	99552	99926	NA	putative lipoprotein BUG3	-0.306423997	0.170933729	0.190243173	0.276098899	0.45045028	0.070520975
144	W903_RS00710	W903_0145	100062	100262	NA	hypothetical protein	-0.192534574	0.692379451	0.35765566	0.303911069	0.789494193	0.186234711
145	W903_RS00715	W903_0146	101052	101648	NA	hypothetical protein	-0.611454993	0.004894732	-0.956318649	6.14803E-09	1.138004054	0.00000731
146	W903_RS00720	W903_0147	101822	102241	NA	hypothetical protein	-0.249531231	0.239586923	-0.330760324	0.00311575	-0.163788919	0.351562424
147	W903_RS00725	W903_0148	102441	102920	NA	putative aTP-dependent endopeptidase clp proteolytic subunit clpP	-0.240270593	0.778085155	0.465729155	0.330438231	0.034920859	0.980116892
148	W903_RS00730	W903_0149	103042	103734	NA	histidine phosphatase super family protein	0.430515804	0.014941801	-0.436939476	0.000117494	0.617546106	0.0000132
149	W903_RS00735	W903_0150	103731	104483	NA	D-alanyl-D-alanine carboxypeptidase family protein	0.098536461	0.58566575	-0.497061064	1.26045E-07	0.635107972	0.00000318
150	W903_RS00740	W903_0151	104480	105055	NA	mannosyl-glycoendo-beta-N-acetylglucosaminidase family protein	0.142240183	0.480806187	-0.793577616	3.54917E-10	0.166906726	0.349219874
151	W903_RS00745	W903_0152	105198	106232	hrcA	heat-inducible transcription repressor HrcA	0.610437264	5.39739E-05	2.744016781	0.000228381	1.751491871	3.3E-48
152	W903_RS00750	W903_0153	106274	106807	NA	grpE family protein	0.708370123	4.7969E-05	1.834919808	0.004215923	1.406509861	4.94E-27
153	W903_RS00755	W903_0154	106988	108817	dnaK	chaperone protein DnaK	1.186451135	1.94284E-13	2.121288703	0.000343446	1.145977543	4.8E-24
154	W903_RS00760	W903_0155	108933	109283	NA	hypothetical protein	1.329109786	7.73448E-09	1.546623024	7.25289E-07	0.721474946	0.00000918
155	W903_RS00765	W903_0156	109536	110675	dnaJ	chaperone protein DnaJ	1.627959919	5.37888E-27	1.858489175	0.001140041	0.90748645	9.82E-12
156	W903_RS00770	W903_0157	110793	112040	NA	bacterial regulatory s, gntR family protein	0.694135957	0.000342888	0.844556014	3.37411E-06	0.136872203	0.442093868
157	W903_RS00775	W903_0158	112111	112887	truA	tRNA pseudouridine(38-40) synthase	-0.990108749	7.50567E-10	2.524474721	7.8801E-133	0.083592168	0.658540428
158	W903_RS00780	W903_0159	112850	113608	NA	pfkB carbohydrate kinase family protein	-0.663372951	0.000170063	2.299746162	1.4723E-125	0.072076725	0.687530699
159	W903_RS00785	W903_0160	113618	114082	NA	ECF-type riboflavin transporter, S component family protein	0.144681768	0.525444689	2.86943696	2.094E-155	0.265682203	0.097248797
160	W903_RS00790	W903_0161	114079	114648	NA	hypothetical protein	0.316970182	0.192577816	2.64700434	2.57282E-90	0.218683003	0.219302092
161	W903_RS00795	W903_0162	114685	115527	NA	mechanosensitive ion channel family protein	0.29817359	0.157715534	1.462170674	1.96258E-38	0.096415255	0.64442102
162	W903_RS00800	W903_0163	115684	116967	tig	trigger factor	-0.983512147	1.87488E-12	-2.299121289	1.2846E-126	0.965027241	8.66E-14
163	W903_RS00805	W903_0164	117156	117740	NA	putative DNA-directed RNA polymerase subunit delta	-1.656692642	1.08554E-31	-0.638816605	1.45923E-10	0.139969715	0.501190182
164	W903_RS00810	W903_0165	118013	119617	pyrG	CTP synthase	-0.259781785	0.272693386	-0.639427401	4.55012E-08	0.266891656	0.053951545
165	W903_RS00815	W903_0166	119726	120652	NA	dienelactone hydrolase family protein	-1.084975582	3.87159E-14	-1.489384362	2.17073E-48	1.013943776	0.0000016
166	W903_RS00820	W903_0167	120699	120784	rRNA	NA	-0.304933837	NA	1.628568963	0.009749001	2.872340379	0.040488827
167	W903_RS00825	W903_0168	120847	121293	dut	dUTP diphosphatase family protein	-1.389300007	3.61483E-12	0.001549282	0.991802713	0.814752999	0.002923477
168	W903_RS00830	W903_0169	121455	122819	radA	DNA repair protein RadA	0.460483052	0.003654195	0.646245135	7.83398E-11	0.178096657	0.264437719
169	W903_RS00835	W903_0170	122955	123452	NA	carbonic anhydrase family protein	-0.1639169	0.811226915	0.072471769	0.600400495	1.073214717	0.0000033
170	W903_RS00840	W903_0171	123606	124925	NA	NAD-dependent glycerol-3-phosphate dehydrogenase family protein	0.311065237	0.057318233	-0.007432704	0.955989916	1.038386572	1.42E-13
171	W903_RS00845	W903_0172	125134	126588	gltX	glutamate--tRNA ligase	-0.620768072	0.000159371	-1.252706396	2.06816E-44	0.555689255	0.0000304
172	W903_RS00850	W903_0173	126628	127596	NA	sugar-binding protein	-0.1979775	0.57805903	2.387797925	3.90485E-57	1.365694591	9.08E-23
173	W903_RS00855	W903_0174	127649	128590	NA	branched-chain amino acid transport system / permease component family protein	-0.359284778	0.292125033	2.126842351	1.22412E-34	1.045647506	2.11E-12
174	W903_RS00860	W903_0175	128592	130070	NA	heme ABC exporter, ATP-binding protein CcmA	0.320790446	0.242243803	2.260404928	6.37762E-53	0.569553227	0.000102504
175	W903_RS00865	W903_0176	130086	130484	rbsD	D-ribose pyranase	-0.056591757	0.92897826	1.778521871	2.95828E-07	0.580417005	0.000165798
176	W903_RS00870	W903_0177	130459	131370	rbsK	ribokinase	-0.506539817	0.057168931	2.139521154	6.47202E-25	0.316414447	0.028154838
177	W903_RS00875	W903_0178	131363	132349	NA	helix-turn-helix family protein	-1.17174469	1.77068E-11	-0.995155433	2.01717E-17	0.221805531	0.173611356
178	W903_RS00880	W903_0179	132531	133619	NA	ftsX-like permease family protein	-0.297971715	0.546474694	1.19343533	2.32608E-06	-0.500689247	0.07942606
179	W903_RS00885	W903_0180	133619	134305	NA	ABC transporter family protein	-0.181079661	0.798433935	0.953281139	0.013366731	-0.957859886	0.014058124
180	W903_RS00890	W903_0181	134336	135007	NA	putative transcriptional activator CadC	-0.176376642	0.486062835	0.822166548	6.72612E-09	-0.763785374	0.00000156
181	W903_RS00895	W903_0182	135051	136070	NA	HAMP domain protein	-0.573136291	0.00089004	0.116669597	0.448188674	-0.595884026	0.0000173
182	W903_RS00900	W903_0183	136224	137414	argG	argininosuccinate synthase	1.479789064	8.63451E-13	1.461969137	7.03975E-24	0.118789008	0.605878823
183	W903_RS00905	W903_0184	137433	138821	argH	argininosuccinate lyase	1.086375733	2.5953E-08	0.848519173	1.06867E-07	0.497145632	0.004598795
184	W903_RS00910	W903_0185	138962	139843	fba	fructose-1,6-bisphosphate aldolase, class II	0.250190176	0.105440968	-2.652061832	1.6435E-122	1.933620797	3.47E-28
185	W903_RS00915	W903_0186	139928	140845	NA	L-2-hydroxyisocaproate dehydrogenase	1.29081304	3.24583E-12	1.185991003	5.79017E-26	0.891066623	0.00000004
186	W903_RS00920	W903_0187	141084	141272	rpmB	ribosomal protein L28	-1.110968591	6.02874E-05	-0.239431846	0.239895374	1.602056355	0.000253851
187	W903_RS00925	W903_0188	141404	141769	NA	asp23 family protein	-0.082449853	0.633686901	0.245317778	0.042955077	0.1283882	0.387825871
188	W903_RS00930	W903_0189	141769	143433	NA	DAK2 domain fusion YloV family protein	0.259307454	0.103410495	0.071291925	0.530226734	-0.410959677	0.000685888
189	W903_RS00935	W903_0190	143581	144465	NA	SPFH domain / Band 7 family protein	-0.77372457	5.96994E-05	-1.556519776	5.12678E-22	0.858217753	0.000159441
190	W903_RS00940	W903_0191	144557	144673	NA	hypothetical protein	-0.499152314	0.125617798	-2.059444407	1.53021E-13	0.484570551	0.630345493
191	W903_RS00945	W903_0192	145040	145270	NA	hypothetical protein	-0.514539603	0.003758254	-1.591369973	1.92723E-15	-0.656756695	0.000579971
192	W903_RS00950	W903_0193	145612	146352	NA	ABC transporter family protein	-0.117464701	0.504826288	-0.633859036	8.95176E-11	-0.203861019	0.214442953
193	W903_RS00955	W903_0194	146362	147912	NA	amino ABC transporter, permease , 3-TM region, His/Glu/Gln/Arg/opine family domain protein	0.035171064	0.845209685	-0.410401195	3.75344E-05	-0.346440511	0.017979031
194	W903_RS00960	W903_0195	148048	149931	NA	hypothetical protein	-0.283142362	0.080270011	0.513276297	4.54675E-06	0.233243193	0.071222138
195	W903_RS00965	W903_0196	149977	150816	NA	bacitracin resistance BacA family protein	-0.450247365	0.0059542	0.092119821	0.35307081	-0.294254407	0.024920127
196	W903_RS00970	W903_0197	150936	151691	NA	negative regulator of genetic competence family protein	-0.374895131	0.029560975	-0.357431264	0.001924934	1.60737069	7.29E-15
197	W903_RS00975	W903_0198	151693	152853	NA	glycosyl transferase 4 family protein	-0.157747199	0.423077872	0.260146488	0.007463719	1.285447905	2.43E-17
198	W903_RS00980	W903_0199	153018	153788	sufC	feS assembly ATPase SufC	0.275976945	0.152314871	0.231361442	0.02746427	0.573740719	0.00000988
199	W903_RS00985	W903_0200	153825	155087	sufD	feS assembly protein SufD	0.47735251	0.006001977	0.698706783	5.12379E-13	0.480385915	0.00011139
200	W903_RS00990	W903_0201	155089	156321	sufS	cysteine desulfurase, SufS family protein	0.569721531	0.001433861	0.98947963	4.94263E-21	0.044379662	0.767293079
201	W903_RS00995	W903_0202	156308	156751	NA	SUF system FeS assembly protein, NifU family	0.440489086	0.016783976	0.833719336	4.56529E-15	0.160313213	0.256395409
202	W903_RS01000	W903_0203	156851	158269	sufB	feS assembly protein SufB	0.332876799	0.058745063	0.542180445	2.63008E-08	0.054533794	0.706907824
203	W903_RS01005	W903_0204	158341	159528	NA	D-alanyl-D-alanine carboxypeptidase family protein	-0.022003079	0.914504482	-0.822324411	1.21046E-14	-0.752932063	5.46E-09
204	W903_RS01010	W903_0205	159681	160868	NA	D-alanyl-D-alanine carboxypeptidase family protein	-0.3314335	0.053941558	-0.764371251	8.84332E-12	-0.53887981	0.001294258
205	W903_RS01015	W903_0206	161158	162813	NA	bacterial extracellular solute-binding s, 5 Middle family protein	0.561450697	0.001675972	-1.625201144	3.64534E-36	-0.03311376	0.856773766
206	W903_RS01020	W903_0207	162932	163846	NA	binding--dependent transport system inner membrane component family protein	0.818582033	2.61384E-06	-1.141002593	1.74469E-16	-0.209438748	0.178921458
207	W903_RS01025	W903_0208	163856	164887	NA	N-terminal TM domain of oligopeptide transport permease C family protein	0.337953786	0.048604072	-1.576489193	1.24623E-26	-0.177893082	0.223626058
208	W903_RS01030	W903_0209	164900	165949	NA	oligopeptide/dipeptide ABC transporter, ATP-binding , C-terminal domain protein	0.32445646	0.043980081	-1.753746219	3.5735E-48	-0.163002339	0.264437719
209	W903_RS01035	W903_0210	165946	166878	NA	ABC transporter family protein	0.056200694	0.78773361	-1.960145966	9.57266E-51	-0.230348908	0.117441741
210	W903_RS01040	W903_0211	167284	168820	rRNA	NA	2.402607004	0.379068928	-4.328438143	0.027268189	-0.679536753	0.713104755
211	W903_RS01045	W903_0212	168880	168952	rRNA	NA	-0.330808731	NA	0.509805822	0.636247259	2.641401175	0.160759087
212	W903_RS01050	W903_0213	169109	172009	rRNA	NA	2.786630679	0.238231645	-4.468779074	0.024971458	0.152447588	0.923364541
213	W903_RS01055	W903_0214	172087	172200	rRNA	NA	0.119583736	NA	0.746123922	0.185782641	-0.260137639	0.424060077
214	W903_RS01060	W903_0215	172205	172278	tRNA	NA	-1.931519103	NA	0.097279542	0.973133722	1.876910281	0.77240887
215	W903_RS01065	W903_0216	172418	173269	ispE	4-(cytidine 5'-diphospho)-2-C-methyl-D-erythritol kinase	0.353970911	0.040437657	0.322052667	0.016738406	1.046760539	6.19E-11
216	W903_RS01070	W903_0217	173355	173798	adcR	transcriptional repressor AdcR	0.600272269	0.000328494	0.318364463	0.016838326	0.473086044	0.00472161
217	W903_RS01075	W903_0218	173801	174511	NA	ABC transporter family protein	0.413094773	0.006604414	-0.009292323	0.946331025	0.078458319	0.659915696
218	W903_RS01080	W903_0219	174501	175313	NA	fecCD transport family protein	0.106949561	0.589504886	-0.200399077	0.153425172	0.020021057	0.934181443
219	W903_RS01085	W903_0220	175523	176554	NA	DNA/RNA non-specific endonuclease family protein	0.454469628	0.077965777	0.518164246	0.014278265	-0.459890842	0.152405312
220	W903_RS01090	W903_0221	177134	178393	tyrS	tyrosine--tRNA ligase	0.186056661	0.278351491	-0.480891213	2.08747E-06	0.555685503	0.000349096
221	W903_RS01095	W903_0222	178504	180801	NA	penicillin binding transpeptidase domain protein	0.428521377	0.003758254	0.498169456	3.41243E-08	-0.476046603	0.000165481
222	W903_RS01100	W903_0223	181325	184900	rpoB	DNA-directed RNA polymerase, beta subunit	0.79661013	1.80906E-08	0.114502112	0.377485448	-0.205160602	0.176970847
223	W903_RS01105	W903_0224	185044	188667	rpoC	DNA-directed RNA polymerase, beta' subunit	1.030931108	1.63205E-13	-0.118723068	0.352563214	-0.22868095	0.123028341
224	NA	W903_0225	188624	188758	NA	glycerophosphoryl diester phosphodiesterase family protein	NA	NA	3.619698662	0.263576189	-0.829372112	0.906985371
225	W903_RS01110	W903_0226	188781	189146	NA	hypothetical protein	-0.652632471	0.033233312	2.585289496	6.17735E-55	0.511437188	0.011418332
226	W903_RS01115	W903_0227	189318	190289	NA	ATPase associated with various cellular activities family protein	0.81488714	0.032654149	2.937594061	1.3017E-39	-0.902538316	0.00018953
227	W903_RS01120	W903_0228	190354	191226	NA	type II secretion system (T2SS), F family protein	0.231570434	0.680577703	2.226869381	1.09893E-19	-0.756955327	0.002046848
228	W903_RS01125	W903_0229	191223	191552	NA	prepilin-type N-terminal cleavage/methylation domain protein	-0.308132563	NA	1.160387342	0.029295295	-0.14327596	0.799319842
229	W903_RS01130	W903_0230	191602	191940	NA	putative competence protein CglD	-0.492708973	NA	1.301855517	0.006234049	-0.710356071	0.063868599
230	W903_RS01135	W903_0231	191966	192211	NA	type II secretory pathway pseudopilin family protein	0.190271476	0.839238676	1.660457576	3.16204E-06	-0.824408896	0.004027062
231	NA	W903_0232	192294	192626	NA	putative comG operon protein 6	0.29605043	0.703622167	1.016138098	0.00461068	-0.924175591	0.001863264
232	W903_RS01145	W903_0233	192604	192975	NA	putative competence protein ComGG	0.65974835	NA	1.447621049	0.001566895	-0.45959792	0.379998225
233	W903_RS01150	W903_0234	193090	194064	NA	N-6 DNA Methylase family protein	-0.877323583	2.67929E-07	-1.498201647	3.86487E-24	-0.228732763	0.137536846
234	W903_RS01155	W903_0235	194096	195289	ackA	acetate kinase	-0.305233385	0.04932856	-0.925492021	3.75505E-17	0.317891144	0.020632542
235	NA	W903_0236	195478	195609	NA	helix-turn-helix family protein	1.718989894	NA	0.056992458	0.960968817	0.057320004	0.970615401
236	W903_RS10230	W903_0237	195603	195854	NA	putative membrane protein	-0.158879769	NA	-0.567912319	0.384566457	0.542503123	0.687649186
237	W903_RS01165	W903_0238	195923	196588	NA	CAAX protease self-immunity family protein	-0.572439722	0.003106036	-1.024210919	7.4477E-14	-0.299629692	0.290813087
238	W903_RS01170	W903_0239	196609	197379	proC	pyrroline-5-carboxylate reductase	0.089357211	0.642158087	-0.505822347	3.28517E-05	-0.579434143	0.00000339
239	W903_RS01175	W903_0240	197449	198516	pepA	glutamyl aminopeptidase	0.613031912	6.88899E-05	0.073720362	0.574644801	-0.900264165	7.58E-11
240	W903_RS01180	W903_0241	198696	198935	NA	putative membrane protein	0.724351387	0.003128903	1.021596287	5.15946E-06	-0.623501057	0.055202321
241	W903_RS01185	W903_0242	199096	199380	NA	hypothetical protein	-0.027680645	0.922793656	0.12481861	0.475150497	-0.211256412	0.42457806
242	W903_RS01190	W903_0243	199377	199700	NA	thioredoxin family protein	-0.348519559	0.069028379	-0.060601633	0.665501691	-0.457136185	0.004783359
243	W903_RS01195	W903_0244	199733	200359	NA	putative tRNA binding domain protein	-0.331198056	0.040469854	-0.352786663	0.001024914	-0.315120236	0.024532597
244	W903_RS01200	W903_0245	200599	201129	NA	methyltransferase domain protein	-0.206351698	0.381549257	-0.245022367	0.049088311	-0.381098312	0.043312547
245	W903_RS01205	W903_0246	201210	201605	NA	single-stranded DNA-binding family protein	0.975709004	0.020388883	2.227993298	5.37911E-18	-1.158893543	0.00000954
246	W903_RS01210	W903_0247	201729	202373	NA	HAD hydrolase, IA, variant 1 family protein	0.71390607	2.66938E-05	0.939163048	7.2982E-13	0.124654797	0.422033296
247	W903_RS01215	W903_0248	202400	204145	NA	histidine kinase-, DNA gyrase B-, and HSP90-like ATPase family protein	0.839116777	3.43143E-08	1.221989025	1.22365E-39	-0.02589105	0.868645392
248	W903_RS01220	W903_0249	204126	204866	NA	response regulator	0.955813834	5.88535E-05	1.27724907	2.34099E-16	0.024769782	0.900482041
249	W903_RS01225	W903_0250	205036	205491	NA	lrgA family protein	-0.09569392	0.816605926	-0.014207576	0.953178734	1.708962087	1.12E-23
250	W903_RS01230	W903_0251	205493	206221	lrgB	antiholin-like protein LrgB	0.795971316	0.006282405	0.462319344	0.006673725	1.393276551	3.38E-20
251	W903_RS01235	W903_0252	206464	208092	NA	bacterial extracellular solute-binding s, 5 Middle family protein	-0.507806435	0.03445741	-0.410641105	0.024604229	-0.670799325	0.013853724
252	W903_RS01240	W903_0253	208277	209182	NA	binding--dependent transport system inner membrane component family protein	1.082592066	0.005898782	0.440077318	0.134906061	-0.98576432	0.002639786
253	W903_RS01245	W903_0254	209179	210000	NA	binding--dependent transport system inner membrane component family protein	1.359694419	0.012886664	0.945287513	0.017440295	-1.066460246	0.009255188
254	W903_RS01250	W903_0255	210012	210815	NA	ABC transporter family protein	1.018620695	0.023654651	0.688056389	0.077274661	-0.635115173	0.113814896
255	W903_RS01255	W903_0256	210799	211425	NA	ABC transporter family protein	0.913746801	0.215830317	0.254967378	0.673886216	-1.465446703	0.016821368
256	W903_RS01260	W903_0257	211708	213738	treP	PTS system, trehalose-specific IIBC component	0.015231679	0.949637452	2.326142501	7.01496E-67	1.277729303	2.35E-15
257	W903_RS01265	W903_0258	213960	215585	treC	alpha,alpha-phosphotrehalase	-0.025029543	0.922986202	1.325073147	2.75731E-19	1.617356986	6.44E-27
258	W903_RS01270	W903_0259	215801	217837	NA	PRD domain protein	-0.407677428	0.11627191	0.939075186	4.91519E-07	-0.023478775	0.921574008
259	W903_RS01275	W903_0260	217840	218124	NA	PTS system, Lactose/Cellobiose specific IIB subunit	1.107348556	0.073197008	2.478812639	1.78033E-11	-0.393535813	0.333141529
260	W903_RS01280	W903_0261	218149	219492	NA	PTS system sugar-specific permease component family protein	1.120984227	2.58341E-05	1.471869654	6.50299E-18	-0.003311788	0.988518541
261	W903_RS01285	W903_0262	219495	220352	NA	thiamine pyrophosphate enzyme, C-terminal TPP binding domain protein	0.085860168	0.772213125	0.686496139	5.313E-05	0.102199713	0.628549531
262	W903_RS01290	W903_0263	220349	221278	NA	transketolase, pyrimidine binding domain protein	0.656454767	0.020946997	1.036467887	1.98101E-09	-0.103298283	0.654522375
263	W903_RS01295	W903_0264	221387	222646	NA	oxidoreductase NAD-binding domain protein	0.723420729	1.48561E-05	-1.560490385	2.41928E-21	1.565135711	4.8E-40
264	W903_RS01300	W903_0265	222734	223003	rpsO	ribosomal protein S15	-0.8909876	3.60753E-10	-0.70510929	9.26627E-10	0.508208491	0.00092145
265	W903_RS01305	W903_0266	223384	225513	pnp	polyribonucleotide nucleotidyltransferase	1.20212224	1.14315E-15	-0.236285793	0.093951661	-0.481919272	0.000200527
266	W903_RS01310	W903_0267	225515	226267	NA	hypothetical protein	1.26711762	2.46291E-15	0.175580549	0.145036933	-0.494103028	0.000738844
267	W903_RS01315	W903_0268	226276	226860	cysE	serine O-acetyltransferase	0.947813619	3.90391E-08	0.057430285	0.666706745	-0.655492893	0.00000103

268	W903_RS01320	W903_0269	226870	227052	NA	putative membrane protein	0.842105672	0.00133644	-0.024228665	0.922291966	-0.766505772	0.005197441
269	W903_RS01325	W903_0270	227049	228392	cysS	cysteine--tRNA ligase	0.755354903	9.18723E-07	0.087500605	0.531444645	-0.689841327	0.000000488
270	W903_RS01330	W903_0271	228385	228771	NA	ribonuclease III domain protein	0.506242307	0.010203245	-0.016257946	0.934830964	-0.787983479	0.000000774
271	W903_RS01335	W903_0272	228880	229629	NA	RNA 2'-O ribose methyltransferase substrate binding family protein	0.513056188	0.0010479	-0.423303985	0.000570048	0.375604383	0.022049432
272	W903_RS01340	W903_0273	229626	230144	NA	yacP-like NYN domain protein	0.47532442	0.002888992	-0.390328268	0.000640865	0.422818643	0.005208865
273	W903_RS01345	W903_0274	230237	231097	NA	EDD, DegV family domain protein	-0.270627932	0.136367761	-0.363093136	0.017465327	0.115719394	0.455140944
274	NA	W903_0275	231200	231328	NA	hypothetical protein	1.282105346	6.82786E-05	1.217263506	0.000278672	0.010138002	0.988149569
275	NA	W903_0276	231443	231562	NA	putative membrane protein	0.775827451	0.015633271	0.855713705	0.004913527	-1.003141822	0.012985194
276	W903_RS01350	W903_0277	231635	231757	NA	helix-turn-helix family protein	0.883158209	0.006679815	1.06330298	7.02663E-05	-0.651099304	0.081645464
277	W903_RS01355	W903_0278	231979	232425	rplM	ribosomal protein L13	-0.418273259	0.006084625	-0.275558357	0.038024879	-1.185022619	3.85E-17
278	W903_RS01360	W903_0279	232446	232838	rpsI	30S ribosomal protein S9	0.374677449	0.024139031	-0.361241177	0.009574104	-1.373856847	8.23E-19
279	W903_RS01365	W903_0280	233296	233586	NA	virulence factor esxA	-0.771416427	2.56634E-05	-0.238058517	0.102017803	-0.742679188	0.0000569
280	W903_RS01370	W903_0281	233760	234095	NA	hypothetical protein	-0.940520496	2.5318E-08	-1.320281434	3.00473E-34	0.165244801	0.353626163
281	NA	W903_0282	234095	235309	NA	hypothetical protein	0.126663777	0.47998015	-0.641089265	1.25534E-12	0.276741076	0.0368163
282	W903_RS01385	W903_0283	235294	235680	NA	hypothetical protein	-0.524607154	0.005148331	-1.204766872	2.07247E-24	0.222702025	0.112216176
283	W903_RS01390	W903_0284	235714	235995	NA	hypothetical protein	0.115656851	0.536398445	-0.698150881	1.90349E-07	0.005045136	0.978471403
284	NA	W903_0285	236582	236704	NA	hypothetical protein	-1.304738976	NA	-0.211533933	0.73319654	0.577621742	0.517492112
285	W903_RS01405	W903_0286	236826	237686	NA	transcriptional activator, Rgg/GadR/MutR family, C-terminal domain protein	-0.358972245	0.087957605	-0.253635736	0.060963871	0.010119007	0.976107129
286	W903_RS01410	W903_0287	237758	238939	NA	major Facilitator Superfamily protein	-0.038795594	0.892105609	0.470319227	0.004651838	0.101230088	0.717911865
287	W903_RS01415	W903_0288	239001	239651	NA	binding--dependent transport system inner membrane component family protein	0.982975101	6.68086E-09	0.444936032	0.000140452	0.154370036	0.359939522
288	W903_RS01420	W903_0289	239652	240578	opuCC	glycine betaine/carnitine/choline-binding protein	0.896766786	2.20645E-09	0.219459113	0.024733716	0.330471308	0.021608614
289	W903_RS01425	W903_0290	240581	241216	NA	binding--dependent transport system inner membrane component family protein	1.135099216	7.66679E-14	0.418125012	6.105E-05	0.146402416	0.427394265
290	W903_RS01430	W903_0291	241216	242361	NA	glycine betaine/L-proline transport ATP binding subunit	0.566330495	0.00047665	-0.108930869	0.256870419	0.15121359	0.379998225
291	W903_RS01435	W903_0292	242789	244325	rRNA	NA	2.4052954	0.380602152	-4.32388054	0.026897209	-0.684170235	0.712050623
292	W903_RS01440	W903_0293	244385	244457	rRNA	NA	-0.844621431	NA	0.387279407	0.765272841	1.037190642	0.68089625
293	W903_RS01445	W903_0294	244614	247515	rRNA	NA	2.838236902	0.217098082	-4.028684939	0.034422254	0.042953888	0.980285285
294	W903_RS01450	W903_0295	247593	247706	rRNA	NA	-1.4469859	0.0426321	0.500192879	0.377625001	0.007916478	0.983910921
295	W903_RS01455	W903_0296	247709	247781	tRNA	NA	-2.96875926	NA	2.024916062	0.699719099	1.876910281	0.77240887
296	W903_RS01460	W903_0297	247787	247857	tRNA	NA	-2.615095903	NA	0.07070075	0.980059476	4.699327825	0.014426963
297	W903_RS01465	W903_0298	247894	247970	rRNA	NA	-0.531150352	0.501170254	1.378076463	0.121735888	5.60029714	4.95E-08
298	W903_RS01470	W903_0299	247978	248049	rRNA	NA	-0.419920405	NA	0.297353191	0.899203501	6.762700952	0.000227944
299	W903_RS01475	W903_0300	248063	248152	rRNA	NA	-1.563337395	0.038467454	0.188459699	0.838900429	5.228776447	8.98E-09
300	W903_RS01480	W903_0301	248163	248236	NA	NA	NA	NA	NA	NA	NA	NA
301	W903_RS01485	W903_0302	248239	248311	rRNA	NA	-0.595913977	NA	0.456861165	0.605105891	6.166648908	0.00187005
302	W903_RS01490	W903_0303	248323	248403	rRNA	NA	-0.162396429	0.839238676	0.245726274	0.774863441	5.409945595	0.0000186
303	W903_RS01495	W903_0304	248410	248480	rRNA	NA	-0.408814678	0.604754542	-0.883741427	0.173947427	5.171918484	0.000410901
304	W903_RS01500	W903_0306	248495	248567	rRNA	NA	-0.277184968	NA	0.226621826	0.780618505	5.199112938	0.00412204
305	W903_RS01505	W903_0307	248574	248645	rRNA	NA	0.405466154	NA	-0.364998529	0.946331025	1.876910281	0.77240887
306	W903_RS01510	W903_0308	248656	248739	NA	NA	NA	NA	NA	NA	-1.163088165	0.867091528
307	W903_RS01515	W903_0309	248944	249504	NA	acetyltransferase domain protein	-0.228113903	0.330951294	0.465308775	0.000141032	0.714851112	0.00000516
308	W903_RS01520	W903_0310	249805	250215	NA	acetyltransferase family protein	-0.157278352	0.528875534	0.993699646	2.98824E-11	1.020509913	4.04E-08
309	W903_RS01525	W903_0311	250205	250912	NA	acetyltransferase domain protein	-0.404864329	0.039475339	0.321094882	0.004215923	0.56881691	0.000125456
310	W903_RS01530	W903_0312	251178	252125	NA	sigma factor regulator C-terminal family protein	-0.889542035	2.0136E-06	-0.469362931	0.000122448	0.205854803	0.230273963
311	W903_RS01535	W903_0313	252122	252613	NA	RNA polymerase sigma factor, sigma-70 family protein	-0.787681971	0.000285909	-0.762737002	1.86551E-06	0.239397808	0.198557097
312	NA	W903_0314	252773	252895	NA	glycerophosphoryl diester phosphodiesterase family protein	-0.76736975	0.138665132	0.979857005	0.000213975	0.377972639	0.381320189
313	W903_RS01540	W903_0315	252897	253058	NA	putative lipoprotein	0.103255552	0.78773361	1.326162089	1.48752E-09	0.126916293	0.684593239
314	W903_RS01545	W903_0316	253193	253801	NA	bacterial regulatory s, tetR family protein	-0.247820553	0.464171074	0.161011305	0.383763582	0.088128899	0.68127306
315	W903_RS01550	W903_0317	253825	254922	NA	ABC-2 type transporter family protein	-0.120005994	0.647719608	0.631332999	8.22248E-05	0.241990433	0.14801528
316	W903_RS01555	W903_0318	254925	255641	NA	ABC transporter family protein	-0.317604623	0.30968107	0.55055317	0.003187135	0.15961666	0.427394265
317	W903_RS01560	W903_0319	255904	256587	NA	hypothetical protein	-0.452376318	0.100497184	1.702090204	1.61481E-36	0.109203735	0.543100155
318	W903_RS01565	W903_0320	256607	256918	NA	transcriptional regulator PadR-like family protein	-0.710068697	0.009136126	2.497453364	1.54498E-73	0.795807374	0.00000523
319	W903_RS01570	W903_0321	257110	257817	NA	hypothetical protein	-1.296567946	3.57153E-19	-1.509323728	2.07548E-59	0.320893956	0.028591308
320	W903_RS01575	W903_0322	258095	259243	nagA	N-acetylglucosamine-6-phosphate deacetylase	0.399373858	0.041146409	0.646032219	1.89313E-11	-0.531328748	0.0000148
321	W903_RS01580	W903_0323	259362	259904	NA	isoprenylcysteine carboxyl methyltransferase family protein	-1.996014176	1.62365E-46	-2.978437285	3.1349E-138	-0.310864713	0.043326436
322	W903_RS01585	W903_0324	260217	261131	glyQ	glycine--tRNA ligase, alpha subunit	0.359440226	0.020363643	-1.849539232	4.70434E-37	1.241607303	1.87E-12
323	W903_RS01590	W903_0325	261131	261772	NA	NADPH-dependent FMN reductase family protein	0.193593043	0.243397527	-2.070903011	6.0079E-66	1.055981232	2.93E-08
324	W903_RS01595	W903_0326	261776	263815	glyS	glycine--tRNA ligase, beta subunit	0.458355481	0.001743195	-1.963547055	2.64094E-38	1.365474134	7.05E-17
325	W903_RS01600	W903_0327	263827	264084	NA	hypothetical protein	1.2923427	5.98477E-14	-1.371007189	6.24028E-23	1.196674962	4.19E-12
326	W903_RS01605	W903_0328	264173	264436	NA	hypothetical protein	1.13930424	4.21228E-07	-0.910634714	5.58589E-06	1.105777619	0.0000134
327	W903_RS01610	W903_0329	264551	266059	glpK	glycerol kinase	1.099480945	3.29177E-10	0.042944223	0.801929886	0.17418533	0.219302092
328	W903_RS01615	W903_0330	266072	267901	NA	FAD dependent oxidoreductase family protein	1.172120885	5.99895E-07	0.632104989	0.000176952	0.253339503	0.075555608
329	W903_RS01620	W903_0331	267913	268611	NA	MIP channel s family protein	1.348498922	1.29539E-05	0.934496481	2.17623E-05	0.313206532	0.102640994
330	W903_RS01625	W903_0332	268699	270036	NA	pyridine nucleotide-disulfide oxidoreductase family protein	0.17141604	0.407712338	1.108007094	1.93094E-17	-0.170837881	0.403808668
331	W903_RS01630	W903_0333	270027	271457	NA	mga helix-turn-helix domain protein	0.242961527	0.279637146	0.5772429	0.000154982	0.019726642	0.935917721
332	W903_RS01635	W903_0334	271582	273567	tkt	transketolase	0.469851069	0.001199773	-0.730457877	8.27676E-09	0.38981943	0.003719429
333	W903_RS01640	W903_0335	273779	274084	NA	enterocin A Immunity family protein	0.094574186	0.699641117	-1.001254106	6.30747E-17	0.605016547	0.0000201
334	W903_RS01645	W903_0336	274104	274838	NA	ABC transporter family protein	0.348810801	0.046727717	-1.005571055	5.535E-14	0.745312457	0.000000396
335	W903_RS01650	W903_0337	274842	276446	NA	putative membrane protein	0.448291208	0.003976592	-0.740291607	2.52419E-09	0.95223701	7.36E-11
336	W903_RS01655	W903_0338	276654	278039	NA	PTS system, glucose-like IIB component domain protein	0.27574653	0.247510373	2.384438811	1.86314E-81	-0.107512623	0.567728126
337	W903_RS01660	W903_0339	278330	278983	proB	glutamate 5-kinase	0.758614067	1.39166E-05	0.558030481	1.86692E-05	-0.348860939	0.02979613
338	W903_RS01665	W903_0340	278993	280246	proA	glutamate-5-semialdehyde dehydrogenase	0.526459783	0.000479238	0.45174082	2.32431E-05	0.258570959	0.088750346
339	W903_RS01670	W903_0341	280380	281276	rsmH	16S rRNA (cytosine(1402)-N(4))-methyltransferase	-0.037872496	0.832460447	0.746066445	5.37115E-15	-0.687644833	0.000014
340	W903_RS01675	W903_0342	281291	281617	ftsL	cell division protein FtsL	-0.283763348	0.118232223	-0.129836626	0.404141085	-0.891178032	0.000000333
341	W903_RS01680	W903_0343	281726	283879	NA	penicillin binding transpeptidase domain protein	0.038452741	0.833343489	-0.395324063	0.000135626	-0.612300262	0.000000774
342	W903_RS01685	W903_0344	283881	284891	mraY	phospho-N-acetylmuramoyl-pentapeptide-transferase	-0.028754419	0.890009272	-0.430214837	8.33598E-05	-0.368220703	0.021159253
343	W903_RS01690	W903_0345	284989	286332	NA	helicase domain protein	0.047110785	0.798030544	-1.084909764	8.76801E-34	0.132288734	0.345453053
344	W903_RS01695	W903_0346	286470	287282	NA	bacterial extracellular solute-binding s, 3 family protein	0.01403295	0.947109325	-1.338337849	2.96155E-25	0.821471434	0.00000136
345	W903_RS01700	W903_0347	287377	288180	NA	amino ABC transporter, permease , 3-TM region, His/Glu/Gln/Arg/opine family domain protein	0.415562446	0.005076862	1.046083299	3.78123E-19	0.209386154	0.217512204
346	W903_RS01705	W903_0348	288180	288923	NA	ABC transporter family protein	-0.048490509	0.784485464	0.456539149	0.000287317	0.298146999	0.028154838
347	W903_RS01710	W903_0349	289045	289269	NA	hypothetical protein	-0.510381572	0.047875045	0.650477096	0.000505388	0.009215609	0.983910921
348	W903_RS01715	W903_0350	289338	290252	trxB	thioredoxin-disulfide reductase	-0.286110878	0.112947803	-0.00438308	0.968072643	1.108879036	1.87E-16
349	W903_RS01720	W903_0351	290408	291868	NA	nicotinate phosphoribosyltransferase family protein	-0.011737523	0.948583974	-0.715726746	3.54169E-15	0.012701532	0.944283724
350	W903_RS01725	W903_0352	291865	292686	nadE	NAD+ synthetase	0.427111518	0.007606915	-0.340792231	0.001252578	-0.10247588	0.500364203
351	W903_RS01730	W903_0353	292799	294133	NA	papain cysteine protease family protein	0.330117536	0.168307538	-1.013841659	7.52719E-15	1.242987801	4.87E-18
352	W903_RS01735	W903_0354	294179	296425	NA	penicillin-binding , 1A family protein	0.437315329	0.002854352	0.243452192	0.031571299	-0.447489112	0.000227944
353	W903_RS01740	W903_0355	296412	297011	recU	recombination protein U	0.174665752	0.349244784	1.209367689	1.23222E-28	-0.120042326	0.51199985
354	W903_RS01745	W903_0356	297086	297604	NA	hypothetical protein	-0.451225073	0.085270842	0.422762609	0.016197645	-0.179462249	0.650219593
355	W903_RS01750	W903_0357	297725	298057	NA	divIVA domain protein	-0.79622695	1.46789E-07	-0.499435194	5.81829E-05	-0.045527066	0.790139283
356	W903_RS01755	W903_0358	298527	299681	NA	hypothetical protein	0.38258162	0.030932373	0.157632171	0.126115349	-0.164700383	0.357329223
357	W903_RS01760	W903_0359	299694	301157	NA	putative holliday junction-specific endonuclease	-0.098642756	0.608738677	-0.423401795	1.18962E-06	0.515915303	0.000159772
358	W903_RS01765	W903_0360	301382	301864	NA	S-Ribosylhomocysteinase family protein	0.044980738	0.816305791	0.381338958	0.003368482	-0.708228384	1.72E-08
359	W903_RS01770	W903_0361	302053	303660	rny	ribonuclease Y	-0.341737463	0.032371599	-1.227916518	2.11969E-29	-0.48393468	0.000292912
360	W903_RS01775	W903_0362	303789	304685	NA	ABC transporter family protein	0.241882234	0.355685142	0.721385851	1.84582E-06	-0.599679705	0.000536747
361	W903_RS01780	W903_0363	304672	305412	NA	ABC-2 type transporter family protein	0.488485738	0.071010168	0.497128298	0.004547655	-0.133727345	0.692408918
362	NA	W903_0364	305799	306494	NA	histidine kinase-, DNA gyrase B-, and HSP90-like ATPase family protein	-0.031273856	0.892719202	0.920218994	3.92E-12	-0.017004871	0.951589951
363	W903_RS01790	W903_0365	306496	307086	NA	bacterial regulatory s, luxR family protein	-0.523620894	0.003758254	1.050824366	6.97348E-25	0.115691078	0.51439141
364	W903_RS01795	W903_0366	307136	307840	NA	hypothetical protein	-0.028397393	0.901111124	1.409838875	1.90964E-37	-0.314097453	0.064407995
365	W903_RS01800	W903_0367	308007	308636	gmk	guanylate kinase	-0.661165011	8.45352E-05	-0.58386471	1.0408E-05	-0.330877831	0.038142
366	W903_RS01805	W903_0368	308659	308973	rpoZ	DNA-directed RNA polymerase, omega subunit	-0.665653731	6.36821E-05	-1.066960662	1.43033E-12	-0.872672594	0.00000954
367	W903_RS01810	W903_0369	309047	311437	priA	primosomal protein N'	0.16926115	0.362916975	0.27972571	0.066115276	-0.735375747	0.00000011
368	W903_RS01815	W903_0370	311484	312419	fmt	methionyl-tRNA formyltransferase	0.657758627	1.82019E-05	0.169882582	0.154532248	-0.258375172	0.065079176
369	W903_RS01820	W903_0371	312409	313731	sun	ribosomal RNA small subunit methyltransferase B	0.319337467	0.048361682	0.11338541	0.289883319	-0.43470884	0.000616242
370	W903_RS01825	W903_0372	313769	314506	NA	phosphatase 2C family protein	0.369798652	0.014692636	0.150175902	0.150892148	-0.694950253	3.98E-08
371	W903_RS01830	W903_0373	314506	316461	NA	phosphotransferase enzyme family protein	-0.078719312	0.647719608	-0.263668025	0.003799502	-0.827575386	3.75E-12
372	W903_RS01835	W903_0374	316622	317317	NA	hypothetical protein	-1.074332559	9.71241E-07	-0.388607182	0.009291667	-0.514134105	0.017166002
373	W903_RS01840	W903_0375	317314	318333	NA	histidine kinase-, DNA gyrase B-, and HSP90-like ATPase family protein	-0.63100365	0.000299908	-0.379780732	0.00132099	-0.413508779	0.010517931
374	W903_RS01845	W903_0376	318326	318967	NA	bacterial regulatory s, luxR family protein	-0.701895788	1.19037E-05	-0.304518777	0.002068081	-0.24061133	0.10148024
375	W903_RS01850	W903_0377	319009	320409	NA	putative bifunctional phosphatase/peptidyl-prolyl cis-trans isomerase	-0.199467122	0.218113744	-0.509569362	8.22223E-08	-0.16047114	0.247694719
376	W903_RS01855	W903_0378	320411	320767	NA	S1 RNA binding domain protein	0.395845804	0.095916955	-0.182640712	0.263082877	-0.296798624	0.153353703
377	W903_RS01860	W903_0379	320814	321590	NA	glycyl-radical enzyme activating family protein	0.566254177	0.021525304	1.104747217	4.5674E-13	-0.772590157	0.00000183
378	W903_RS01865	W903_0380	321711	322466	NA	deoR C terminal sensor domain protein	0.109346536	0.589504886	0.605526205	4.09962E-06	-0.510171398	0.00091455
379	W903_RS01870	W903_0381	322585	323568	NA	putative sugar-binding domain protein	0.07129117	0.71314676	0.417781032	0.000227348	-0.76734624	4.82E-09
380	W903_RS01875	W903_0382	323772	324095	NA	PTS system, Lactose/Cellobiose specific IIA subunit	1.05629019	0.076815293	0.705348223	0.038328853	-0.806653704	0.031848646
381	W903_RS01880	W903_0383	324112	324432	NA	PTS system, Lactose/Cellobiose specific IIB subunit	0.124062982	0.858438886	0.676481862	0.134649327	-1.096492903	0.004636009
382	W903_RS01885	W903_0384	324434	325735	NA	PTS system, lactose/cellobiose IIC component family protein	0.480933226	0.11315208	1.071144774	2.65308E-09	-1.212207181	2.8E-12
383	W903_RS01890	W903_0385	325916	328372	NA	glycyl radical enzyme, PFL2/glycerol dehydratase family protein	0.79925547	6.09827E-05	0.652835071	6.4858E-06	-0.512018729	0.0000668
384	W903_RS01895	W903_0386	328382	329050	NA	transaldolase family protein	0.644067667	0.009246904	0.881059801	7.88273E-10	-0.612284601	0.000028
385	W903_RS01900	W903_0387	329118	330206	gldA	glycerol dehydrogenase	0.304046398	0.364527265	0.551169194	0.003271423	-0.873015681	7.99E-08
386	W903_RS01905	W903_0388	330358	331284	cysK	cysteine synthase A	-0.08615722	0.64460116	-0.790308582	2.557E-10	0.179006614	0.198451833
387	W903_RS01910	W903_0389	331375	332019	NA	hypothetical protein	0.332796326	0.084095498	0.048134912	0.775345315	-0.098839756	0.748036011
388	W903_RS01915	W903_0390	332075	333364	NA	helicase domain protein	0.046254976	0.92288861	1.406160426	2.80568E-05	-0.325099945	0.510360749
389	W903_RS01920	W903_0391	333493	334029	NA	phosphoribosyl transferase domain protein	-0.034428751	NA	1.560866927	0.006415346	-0.664244581	0.476727024
390	W903_RS01925	W903_0392	334106	334660	raiA	ribosomal subunit interface protein	-0.356616931	0.045895244	-0.198271455	0.053199793	1.527443578	2.55E-30
391	W903_RS01930	W903_0393	334991	336527	rRNA	NA	2.668577167	0.226196936	-3.898324137	0.035553265	-0.752578182	0.674976013
392	W903_RS01935	W903_0394	336587	336659	rRNA	NA	-1.15094223	NA	1.679406325	0.228362631	2.245735273	0.219925518
393	W903_RS01940	W903_0395	336816	339716	rRNA	NA	2.78714616	0.238523766	-4.467329348	0.024974968	0.141506046	0.927962103
394	W903_RS01945	W903_0396	339794	339907	rRNA	NA	0.57966878	0.483069328	0.829343796	0.114633161	-0.111469228	0.750481516
395	W903_RS01950	W903_0397	339912	339985	rRNA	NA	-0.968406418	NA	1.048062688	0.86167603	-1.163088165	0.867091528
396	W903_RS01955	W903_0398	340201	341553	NA	aspartate kinase domain protein	-1.224013428	4.60599E-17	-1.781623265	1.62488E-55	0.346660017	0.071776352
397	W903_RS01960	W903_0399	341677	342297	NA	beta-phosphoglucomutase, putative	-1.092813528	1.3838E-12	-0.271294026	0.024654561	1.355394805	0.000000142
398	W903_RS01965	W903_0400	342435	343226	NA	enoyl-CoA hydratase/isomerase family protein	0.03865415	0.830008384	-1.540816338	2.38979E-14	0.487203274	0.002815495
399	W903_RS01970	W903_0401	343322	343756	NA	iron dependent repressor, N-terminal DNA binding domain protein	-0.182719651	0.277470661	-0.838229342	1.0477E-06	1.249422378	2.4E-16
400	W903_RS01975	W903_0402	343756	344727	fabH	3-oxoacyl-[acyl-carrier-] synthase III family protein	0.126578717	0.476095248	-0.828434146	5.03009E-06	0.91262979	3.37E-12
401	W903_RS01980	W903_0403	344785	345009	NA	phosphopantetheine attachment site family protein	-0.333661013	0.02634239	-1.338078728	1.95733E-13	0.966265216	2.42E-10

402	W903_RS01985	W903_0404	345163	346122	NA	nitronate monooxygenase family protein	0.932293426	2.06104E-11	-0.866663843	8.14412E-06	0.741512905	0.000000343
403	W903_RS01990	W903_0405	346142	347068	fabD	malonyl CoA-acyl carrier protein transacylase	0.843323813	6.02844E-09	-1.044815067	0.010498995	1.052101862	9.45E-17
404	W903_RS01995	W903_0406	347077	347811	fabG	3-oxoacyl-[acyl-carrier-protein] reductase	0.909315838	3.58569E-11	-1.253170818	0.001026254	1.215473168	8.27E-23
405	W903_RS02000	W903_0407	347827	349059	fabF	beta-ketoacyl-acyl-carrier-protein synthase II	0.593860516	7.32293E-05	-1.589277026	5.63478E-16	1.17414216	8.5E-23
406	W903_RS02005	W903_0408	349061	349561	accB	acetyl-CoA carboxylase, biotin carboxyl carrier protein	1.181927085	4.0096E-16	-1.620560291	1.99984E-20	0.897056606	2.04E-13
407	W903_RS02010	W903_0409	349558	349980	fabZ	beta-hydroxyacyl-(acyl-carrier-protein) dehydratase FabZ	0.801637199	2.25463E-06	-1.829847744	2.14854E-30	1.170840297	9.25E-17
408	W903_RS02015	W903_0410	350018	351388	accC	acetyl-CoA carboxylase, biotin carboxylase subunit	1.001484901	2.40058E-11	-1.925057187	4.83193E-34	0.868102152	7.52E-12
409	W903_RS02020	W903_0411	351397	352272	accD	acetyl-CoA carboxylase, carboxyl transferase, beta subunit	0.808604069	4.54971E-08	-2.370628542	1.50877E-67	0.794306156	5.62E-10
410	W903_RS02025	W903_0412	352277	353038	accA	acetyl-CoA carboxylase, carboxyl transferase, alpha subunit	1.13638034	4.87263E-14	-2.29644155	1.02391E-86	0.57050629	0.0000317
411	W903_RS02030	W903_0413	353506	354138	NA	glucose-6-phosphate 1-dehydrogenase family protein	0.432836348	0.043870218	-1.335223846	9.59178E-29	0.719093146	0.000370731
412	W903_RS02035	W903_0414	354184	355461	serS	serine--tRNA ligase	0.765627174	2.53023E-07	-1.972815653	2.51855E-66	1.764091626	7.68E-30
413	W903_RS02040	W903_0415	355827	356819	NA	acyltransferase family protein	-0.250846089	0.252500145	1.829073512	1.24499E-73	0.806478529	4.04E-08
414	W903_RS02045	W903_0416	356857	357219	NA	hypothetical protein	-0.199184362	0.240481793	-0.830694884	7.49911E-17	-0.605593556	0.0000983
415	W903_RS02050	W903_0417	357338	358249	NA	PTS system, mannose/fructose/sorbose , IID component family protein	0.805959492	3.78829E-08	-1.263889754	3.47832E-33	-0.384017537	0.005640612
416	W903_RS02055	W903_0418	358264	359076	NA	PTS system sorbose-specific iic component family protein	0.907354986	2.19433E-10	-1.335043314	1.77576E-26	-0.354736858	0.041747832
417	W903_RS02060	W903_0419	359109	360119	manX	PTS system mannose-specific EIIAB component	0.117396937	0.52257258	-1.93776494	2.0302E-85	-0.242187742	0.096504688
418	W903_RS02065	W903_0420	360422	361234	NA	HAD hydrolase, IIB family protein	-0.685885182	7.44243E-06	-0.361976717	0.00068489	-0.006358892	0.980285285
419	W903_RS02070	W903_0421	361323	361907	NA	putative membrane protein	-0.889570946	1.46769E-08	-0.187319893	0.05050587	0.233210512	0.147555743
420	W903_RS02075	W903_0422	361997	362608	NA	hypothetical protein	-0.429869219	0.004487954	-0.205901956	0.032057896	0.40406933	0.014884288
421	W903_RS02080	W903_0423	362711	364132	NA	permease family protein	0.323194554	0.140752343	-0.794075633	5.89751E-15	-0.615075376	0.0000312
422	W903_RS02085	W903_0424	364281	364724	NA	tRNA threonylcarbamoyl adenosine modification protein YjeE	-0.460681074	0.011313412	0.364289808	0.002558756	-0.37776872	0.120686609
423	W903_RS02090	W903_0425	364717	365238	NA	acetyltransferase family protein	-0.094529286	0.624569854	0.107521264	0.339547959	-0.394928854	0.026766182
424	W903_RS02095	W903_0426	365247	366554	brpA	biofilm regulatory protein A	-0.134404325	0.427430056	0.134937268	0.143554214	-0.547174199	0.0000449
425	W903_RS02100	W903_0427	366618	366914	NA	hypothetical protein	-0.636554006	0.004850929	-0.151549388	0.367209097	0.45822089	0.000792875
426	W903_RS02105	W903_0428	366911	367330	NA	HIT domain protein	0.502117402	0.002136569	1.569375276	1.83152E-29	-0.205649023	0.165733102
427	W903_RS02110	W903_0429	367666	368169	NA	hypothetical protein	-2.167807085	1.47349E-27	-3.629377703	0	0.339840902	0.014108968
428	W903_RS02115	W903_0430	368479	369222	NA	ABC transporter family protein	-0.366247486	0.024133038	-1.194208553	4.98014E-31	1.347400041	1.42E-17
429	W903_RS02120	W903_0431	369206	370240	NA	bacterial ABC transporter EcsB family protein	-0.01914786	0.914074262	-0.610640558	4.51752E-08	1.326614469	1.47E-20
430	W903_RS02125	W903_0432	370298	371098	NA	phosphotransferase enzyme family protein	-0.213341577	0.196263472	-0.808912416	1.2019E-16	1.235191766	5.57E-18
431	W903_RS02130	W903_0433	371088	371723	trmB	tRNA (guanine-N(7)-)-methyltransferase	-0.837659661	2.75984E-08	-0.604082165	2.1851E-08	1.395353168	2.05E-18
432	W903_RS02135	W903_0434	371767	371853	tRNA	NA	3.09206873	NA	0.473960349	0.776325669	3.450657021	0.011471626
433	NA	W903_0435	372176	372439	NA	hypothetical protein	0.127386303	NA	0.899345443	0.046369028	-0.332807274	0.319853189
434	NA	W903_0436	372929	373042	NA	glycerophosphoryl diester phosphodiesterase family protein	0.753565871	0.367966213	0.755314117	0.036274513	-0.519460076	0.094230027
435	NA	W903_0437	373292	373468	NA	glycerophosphoryl diester phosphodiesterase family protein	0.295420677	NA	0.619664968	0.169864073	-0.346782223	0.357329223
436	NA	W903_0438	373575	373739	NA	hypothetical protein	-0.283138384	NA	1.191920984	0.040036379	-0.496248781	0.284358956
437	W903_RS02150	W903_0439	373741	374775	NA	replication initiator A family protein	-0.443583354	0.296007939	0.591057016	0.020087488	-0.366623505	0.088353495
438	W903_RS02155	W903_0440	374769	375458	NA	glycerophosphoryl diester phosphodiesterase family protein	-0.343054637	0.519384764	0.774419109	0.005312801	-0.358647683	0.125065529
439	NA	W903_0441	375455	375595	NA	glycerophosphoryl diester phosphodiesterase family protein	-0.378845666	NA	0.900232408	0.460011568	0.261859367	0.849034787
440	W903_RS02160	W903_0442	375592	375789	NA	helix-turn-helix family protein	-1.065965087	0.060639429	0.246498892	0.470001629	-0.00875693	0.982093165
441	W903_RS02165	W903_0443	376172	376471	NA	hypothetical protein	-0.362287433	0.468176574	0.489982656	0.04244246	-0.233537577	0.418816098
442	W903_RS02170	W903_0444	376488	378752	NA	LPXTG cell wall anchor domain protein	-0.247319078	0.315859247	0.302059246	0.021252642	-0.206497347	0.204976505
443	W903_RS02175	W903_0445	378772	379494	NA	LPXTG cell wall anchor domain protein	0.224135161	0.525444689	0.479422146	0.030672411	-0.333540222	0.07829302
444	W903_RS02180	W903_0446	379547	382369	NA	putative cross-wall-targeting lipoprotein signal	-0.379643142	0.090008853	-0.086177761	0.504130818	-0.058960886	0.750481516
445	W903_RS02185	W903_0447	382664	382891	NA	putative membrane protein	-0.804988678	0.005621574	-0.357434787	0.066227141	0.268527713	0.451249358
446	W903_RS02190	W903_0448	382996	383577	NA	hypothetical protein	-0.7551924	1.37849E-05	0.733869599	5.07661E-13	-0.183958081	0.219925518
447	W903_RS02195	W903_0449	383628	384380	NA	putative membrane protein	-0.556628858	0.143974617	-0.216800211	0.45028242	-0.310141632	0.302332889
448	W903_RS02200	W903_0450	384417	384713	NA	glycerophosphoryl diester phosphodiesterase family protein	-1.29423176	0.083125569	-0.631028613	0.201124848	-0.812024043	0.287744144
449	W903_RS02205	W903_0451	384706	385164	NA	hypothetical protein	-0.214694886	0.711554523	0.577115431	0.078705731	-1.064816826	0.001657274
450	W903_RS02210	W903_0452	385226	385585	NA	hypothetical protein	-1.028297839	0.03641127	-1.126034514	0.005168433	-1.0751709	0.02115469
451	W903_RS02215	W903_0453	385566	387926	NA	AAA-like domain protein	0.4514854	0.124473964	-0.209685268	0.250206519	-0.798938986	0.0000533
452	W903_RS02220	W903_0454	387919	388512	NA	hypothetical protein	0.316301403	0.460404359	0.176518068	0.532192284	-1.052864008	0.000042
453	W903_RS02225	W903_0455	388536	388862	NA	glycerophosphoryl diester phosphodiesterase family protein	0.395030205	0.427465018	0.565863559	0.0535035	-1.090420904	0.000128899
454	W903_RS02230	W903_0456	388897	391587	NA	peptidase M23 family protein	0.475338649	0.057826884	0.443946164	0.005852891	-0.901888653	0.0000061
455	W903_RS02235	W903_0457	391608	392213	NA	putative prgL	0.234821203	0.71409499	-0.75693336	0.087222286	-1.13499573	0.009785052
456	W903_RS02240	W903_0458	392233	392505	NA	hypothetical protein	0.044184452	0.95607518	-0.271425984	0.653083749	-0.504184262	0.466417799
457	W903_RS02245	W903_0459	392505	393290	NA	hypothetical protein	-0.093284633	0.844341147	-0.702844855	0.013091519	-0.942368164	0.000770452
458	W903_RS02250	W903_0460	393312	393803	NA	hypothetical protein	0.275313281	0.50440675	-0.430023707	0.114442897	-1.023126463	0.000176965
459	W903_RS02255	W903_0461	393803	395848	NA	type IV secretory system Conjugative DNA transfer family protein	0.857236638	0.001726505	0.075259658	0.773514954	-1.224981639	1.11E-10
460	W903_RS02260	W903_0462	395890	396255	NA	glycerophosphoryl diester phosphodiesterase family protein	1.022684016	0.079081585	0.121000325	0.80144881	-1.125894042	0.000233496
461	W903_RS02265	W903_0463	396257	399496	NA	hypothetical protein	1.044061535	5.03682E-05	-0.335192262	0.117378053	-1.074450631	0.000000591
462	W903_RS02270	W903_0464	399548	399847	NA	glycerophosphoryl diester phosphodiesterase family protein	0.532599824	0.466957553	-2.618873431	1.10919E-06	0.779729659	0.439553611
463	W903_RS02275	W903_0465	400190	400750	NA	glycerophosphoryl diester phosphodiesterase family protein	0.377413387	0.58319645	1.500314548	8.06384E-06	-1.050497054	0.00029936
464	W903_RS02280	W903_0466	400753	401127	NA	bacterial mobilization family protein	-0.173907123	NA	1.520443483	0.041037569	-1.183016212	0.067702649
465	W903_RS02285	W903_0467	401099	402739	NA	relaxase/mobilization nuclease domain protein	0.409543699	0.402857741	0.910297555	0.005190617	-1.270204381	0.000878313
466	NA	W903_0468	402768	402917	NA	glycerophosphoryl diester phosphodiesterase family protein	0.474200736	NA	0.134087957	0.885242075	-0.289490917	0.956762095
467	W903_RS02290	W903_0469	402876	403283	NA	hypothetical protein	-0.149682661	0.604754542	0.364706579	0.036828065	0.352603902	0.242128369
468	W903_RS02295	W903_0470	403314	404597	NA	hypothetical protein	0.112436776	0.648392027	0.366905006	0.006241848	-0.303783783	0.13652889
469	W903_RS02300	W903_0471	405192	405575	rimP	ribosome maturation factor rimP	0.222693388	0.251844765	-0.21015826	0.312600545	1.498066714	1.77E-15
470	W903_RS02305	W903_0472	405611	406762	nusA	transcription termination factor NusA	0.152571017	0.381549257	-0.565599676	0.010522808	1.414220516	1.04E-29
471	W903_RS02310	W903_0473	406784	407080	NA	hypothetical protein	0.23473817	0.229316616	-0.656103548	0.001894406	1.290577782	5.05E-15
472	W903_RS02315	W903_0474	407073	407375	NA	ribosomal L7Ae/L30e/S12e/Gadd45 family protein	0.011896621	0.950641272	-0.739021243	0.00095757	1.318858926	1.9E-15
473	W903_RS02320	W903_0475	407395	410178	infB	translation initiation factor IF-2	0.600759179	7.43109E-05	-0.299446878	0.090909952	0.921069422	7.95E-14
474	W903_RS02325	W903_0476	410287	410637	rbfA	ribosome-binding factor A	0.373124246	0.059041648	-0.234482131	0.225683105	0.659938451	0.000955697
475	W903_RS02330	W903_0477	410721	411725	NA	alpha/beta hydrolase fold family protein	0.084288631	0.733854288	-0.901248242	1.56825E-18	-1.958812496	6.74E-24
476	W903_RS02335	W903_0478	411888	412304	NA	copper transport repressor, CopY/TcrY family	0.239101143	0.513275836	0.921726731	0.000661892	0.491652914	0.018398621
477	W903_RS02340	W903_0479	412317	414551	NA	copper-translocating P-type ATPase	0.059864062	0.769509545	1.462840341	4.84711E-29	-0.35292583	0.007696753
478	W903_RS02345	W903_0480	414592	414798	NA	heavy-metal-associated domain protein	0.265179109	0.483314144	1.391987013	1.11234E-11	-0.19546116	0.403367237
479	W903_RS02350	W903_0481	414908	415522	NA	hypothetical protein	0.555106435	0.00033977	0.624647911	1.27873E-07	0.210911258	0.161206091
480	W903_RS02355	W903_0482	415537	416349	NA	HAD hydrolase, IIB family protein	-0.128099425	0.466665594	-0.46295342	5.36623E-07	0.567658926	0.0000722
481	W903_RS02360	W903_0483	416462	419104	polA	DNA polymerase I	0.133449737	0.423657793	-0.44027238	7.85377E-07	0.425182778	0.00102214
482	W903_RS02365	W903_0484	419134	419574	NA	coA binding domain protein	-0.142599267	0.407970269	-0.591106209	1.59006E-07	0.586520802	0.0000153
483	W903_RS02370	W903_0485	419656	420135	NA	ferric uptake regulator family protein	-0.333235905	0.048427359	0.25434516	0.017968425	-0.086493496	0.581394606
484	W903_RS02375	W903_0486	420287	421852	NA	LPXTG cell wall anchor domain protein	-0.2402247	0.344004389	0.163698322	0.366292005	0.621887182	0.0000583
485	W903_RS02380	W903_0487	421965	422651	NA	putative transcriptional activator CadC	-0.627096062	0.000183797	0.362823079	0.000531942	-0.076766831	0.653517297
486	W903_RS02385	W903_0488	422653	423690	NA	HAMP domain protein	-0.849678806	1.97167E-07	-0.350158177	0.00092861	-0.455617771	0.001497599
487	W903_RS02390	W903_0489	423704	424444	NA	hypothetical protein	-0.252360905	0.150816281	0.387112883	0.008468648	-0.989877256	1.2E-09
488	W903_RS02395	W903_0490	424631	425773	tgt	queuine tRNA-ribosyltransferase	-0.255188264	0.116396909	-1.575725751	4.99882E-36	-0.407153255	0.02577451
489	NA	W903_0491	425748	425870	NA	glycerophosphoryl diester phosphodiesterase family protein	-0.089388986	NA	-3.415447656	0.377750863	NA	NA
490	W903_RS02400	W903_0492	425882	426190	NA	CHY zinc finger family protein	0.373207213	0.38120064	1.155995892	2.28654E-06	-0.259464147	0.445438442
491	W903_RS02405	W903_0493	426197	426736	NA	biotin transporter BioY	0.496687836	0.179320305	0.6792325	0.006051031	-0.40564807	0.161814946
492	W903_RS02410	W903_0494	426875	427651	NA	metallo-beta-lactamase superfamily protein	-0.029587273	0.876832988	-0.802543921	1.49462E-15	2.398038808	2.4E-49
493	W903_RS02415	W903_0495	427651	428157	NA	cytidine and deoxycytidylate deaminase zinc-binding region family protein	0.627920361	0.000291063	-0.378217806	0.000229417	1.562136889	2.93E-27
494	NA	W903_0496	428259	428393	NA	glycerophosphoryl diester phosphodiesterase family protein	0.166995372	0.593646859	1.297614852	3.22786E-15	0.30375489	0.124011293
495	W903_RS02425	W903_0497	428429	429778	NA	phosphoglucose isomerase family protein	-0.128783892	0.446374739	-0.785248983	4.69363E-18	0.530168658	0.000237946
496	W903_RS02430	W903_0498	430100	430627	NA	5-formyltetrahydrofolate cyclo-ligase	-0.536093312	0.012850849	-0.023346334	0.883933745	0.440976137	0.026693801
497	W903_RS02435	W903_0499	430618	431295	NA	rhomboid family protein	-0.43246043	0.007514823	-0.02247665	0.882747555	0.265855512	0.058483763
498	W903_RS02440	W903_0500	431427	432470	NA	basic membrane family protein	-0.738416658	2.82742E-06	-0.253551795	0.030525336	0.415464166	0.00130638
499	W903_RS02445	W903_0501	432561	433460	galU	UTP-glucose-1-phosphate uridylyltransferase	0.304109152	0.052360454	-0.897709312	1.53658E-20	0.880088167	5.79E-10
500	W903_RS02450	W903_0502	433497	434513	gpsA	glycerol-3-phosphate dehydrogenase	0.869688455	2.56544E-09	-0.699928471	3.8949E-13	0.59576373	0.000169142
501	W903_RS02455	W903_0503	434683	435012	rnpA	ribonuclease P protein component	-0.953135914	1.22964E-09	-0.441581856	4.93859E-05	0.739848314	0.000190396
502	W903_RS02460	W903_0504	435025	435840	NA	membrane insertase, YidC/Oxa1 family domain protein	-0.764158037	1.25872E-06	-0.873923697	2.83318E-17	0.584463989	0.000021
503	W903_RS02465	W903_0505	435848	436669	NA	KH domain protein	-0.765082337	7.75869E-08	-1.222309788	6.44862E-37	0.610857116	0.0000204
504	W903_RS02470	W903_0506	437018	438554	rRNA	NA	2.402121674	0.377928802	-4.309363724	0.025957193	-0.686798563	0.710491277
505	W903_RS02475	W903_0507	438614	438686	rRNA	NA	-0.604774779	NA	-0.082256581	0.960423095	3.04731421	0.151503588
506	W903_RS02480	W903_0508	438843	441741	rRNA	NA	2.826876075	0.219716273	-4.025499514	0.035237629	-0.009633346	0.996076319
507	W903_RS02485	W903_0509	441819	441932	rRNA	NA	-1.591778373	0.012387597	0.329722515	0.559046211	0.007515009	0.984008521
508	W903_RS02490	W903_0510	441935	442007	rRNA	NA	0.989144927	NA	1.994972911	0.726361185	0.011647432	0.997398417
509	W903_RS02495	W903_0511	442013	442083	rRNA	NA	0.694939672	NA	-0.395563864	0.90981923	4.280064253	0.040445234
510	W903_RS02500	W903_0512	442120	442196	rRNA	NA	-0.058656773	NA	1.231302304	0.18320809	7.319392502	0.0000266
511	W903_RS02505	W903_0513	442204	442275	NA	NA	NA	NA	NA	NA	NA	NA
512	W903_RS02510	W903_0514	442390	442923	NA	hypothetical protein	0.117071979	0.565005127	1.199422848	1.6836E-16	1.083227081	0.0000112
513	W903_RS02515	W903_0515	443007	443783	NA	recX family protein	-1.049954893	1.3009E-09	-0.72290606	5.90109E-08	0.742860756	0.007570098
514	W903_RS02520	W903_0516	443882	445237	NA	23S rRNA (uracil-5-)-methyltransferase RumA	-0.54796748	0.000852337	-0.649655645	7.95523E-11	0.523471859	0.001076653
515	W903_RS02525	W903_0517	445506	446270	NA	phosphorylase superfamily protein	-0.487311042	0.005514122	-1.176989884	5.75311E-30	0.901270712	0.0000422
516	W903_RS02530	W903_0518	446304	446747	NA	acetyltransferase family protein	-0.205035597	0.338526977	-0.763383207	1.46525E-16	0.836467335	0.000000269
517	W903_RS02535	W903_0519	446920	450621	scpA	C5a peptidase	-0.307168352	0.064728237	-1.695548389	1.7761E-46	-0.807078644	0.000000114
518	W903_RS02540	W903_0520	450831	451739	NA	glycosyl transferase 2 family protein	-0.724139799	3.39477E-05	-1.271256814	3.17579E-38	-0.673042178	0.00000718
519	W903_RS02545	W903_0521	452293	453303	nrdF	ribonucleoside-diphosphate reductase subunit beta	-0.136475949	0.755212804	-0.757714727	5.29993E-05	-0.382534059	0.135732891
520	W903_RS02550	W903_0522	453304	453717	NA	nrdI protein	0.011574989	0.981959839	-0.748176992	0.001681175	-0.492024951	0.047949217
521	W903_RS02555	W903_0523	453719	455887	NA	ribonucleoside-diphosphate reductase, alpha subunit	0.091253966	0.705759832	-0.383904543	0.005397271	0.01128558	0.951049564
522	W903_RS02560	W903_0524	455950	459117	NA	leucine rich repeats family protein	-0.154553388	0.466856237	-0.601985775	2.90751E-07	0.107948217	0.484211778
523	W903_RS02565	W903_0525	459130	459519	NA	pyridoxamine 5'-phosphate oxidase family protein	0.221491709	0.570214659	-0.377847274	0.048593988	0.0388461	0.896942103
524	W903_RS02570	W903_0526	459563	460054	NA	gyrI-like small molecule binding domain protein	0.890577709	1.31818E-05	1.449419683	5.15521E-30	-0.843151379	2.53E-11
525	W903_RS02575	W903_0527	460293	460577	NA	hypothetical protein	0.266917881	0.31244217	0.882273543	2.07842E-11	-0.525425554	0.0000301
526	W903_RS02580	W903_0528	460664	460981	NA	carboxymuconolactone decarboxylase family protein	0.706308023	0.002383671	1.190849909	7.90155E-24	-0.649268284	0.0000025
527	W903_RS02585	W903_0529	461005	461400	NA	cupin domain protein	0.90600684	1.02679E-06	1.280332261	1.77838E-28	-0.535240827	0.0000177
528	W903_RS02590	W903_0530	461410	461799	adhR	HTH-type transcriptional regulator AdhR	0.767232058	5.80076E-05	1.259549294	1.52867E-25	-0.504400428	0.0000273
529	W903_RS02595	W903_0531	461815	462852	NA	zinc-binding dehydrogenase family protein	0.704704787	2.50467E-05	1.030476791	6.85182E-29	-0.289020779	0.028614106
530	W903_RS02600	W903_0532	462962	463816	NA	aldo/keto reductase family protein	0.835020761	0.000354474	1.906719998	3.52541E-50	-0.707002351	0.00000397
531	W903_RS02605	W903_0533	463901	464764	NA	cation diffusion facilitator transporter family protein	-0.100757303	0.780622309	2.385500714	4.27771E-36	-2.561340853	1.41E-65
532	W903_RS02610	W903_0534	464896	465420	NA	bacterial regulatory s, tetR family protein	-0.270931013	0.231815288	-1.137863751	1.36027E-11	0.179529816	0.654525814
533	NA	W903_0535	465599	465739	NA	glycerophosphoryl diester phosphodiesterase family protein	-0.275545928	0.589912333	-1.003941795	0.024283597	-0.223418017	0.868645392
534	W903_RS02615	W903_0536	465826	466032	NA	putative membrane protein	0.112624999	0.768072746	-0.193179507	0.575919546	0.4284667	0.437834739
535	W903_RS02620	W903_0537	466129	466350	NA	hypothetical protein	3.271121411	NA	1.152665145	0.459351623	-0.566704482	0.804217297

536	W903_RS02625	W903_0538	466451	466521	tRNA	NA	1.666508696	0.000381532	-0.4203245	0.252485891	-0.36447215	0.383009534
537	W903_RS02630	W903_0539	466592	466897	NA	cupin domain protein	0.373715286	0.086558854	-0.880353627	3.62996E-08	-0.309064863	0.058112236
538	W903_RS02635	W903_0540	466899	467237	NA	cupin domain protein	0.542105589	0.003976592	-0.786623744	6.62041E-14	-0.079276927	0.663330816
539	W903_RS02640	W903_0541	467258	468013	NA	methyltransferase domain protein	-0.384635286	0.028734377	-1.365369977	4.75548E-38	0.28469579	0.06594996
540	W903_RS02645	W903_0542	468213	470273	NA	deoR-like helix-turn-helix domain protein	0.223844595	0.519650659	1.882505833	6.77331E-33	-0.455845327	0.009420368
541	W903_RS02650	W903_0543	470382	470837	NA	phosphoenolpyruvate-dependent sugar phosphotransferase system, EIIA 2 family protein	-0.180488308	0.736276249	-0.032043447	0.94134607	-1.455092662	0.00000455
542	W903_RS02655	W903_0544	470856	471134	NA	PTS system, Lactose/Cellobiose specific IIB subunit	0.672355351	NA	2.135025658	2.43506E-05	-1.32864203	0.00000954
543	W903_RS02660	W903_0545	471146	472483	NA	PTS system sugar-specific permease component family protein	-0.095603897	0.835816596	2.075917384	2.17729E-14	-1.176421559	1.47E-09
544	W903_RS02665	W903_0546	472506	473336	NA	class II Aldolase and Adducin N-terminal domain protein	0.132861975	0.786454518	1.906729802	1.93094E-17	-1.287393515	8.61E-14
545	W903_RS02670	W903_0547	473471	473935	NA	phosphoenolpyruvate-dependent sugar phosphotransferase system, EIIA 2 family protein	-0.092796959	0.844341147	0.241956862	0.435207178	-0.895422024	0.002010215
546	W903_RS02675	W903_0548	473948	475279	NA	PTS system sugar-specific permease component family protein	0.685832206	0.066496319	1.649931848	5.87236E-12	-0.93516754	0.00000396
547	W903_RS02680	W903_0549	475281	475565	NA	PTS system, Lactose/Cellobiose specific IIB subunit	0.526814043	0.302225232	1.630959674	1.88849E-09	-0.992169902	0.000000986
548	W903_RS02685	W903_0550	475850	476680	NA	bacterial regulatory helix-turn-helix s, AraC family protein	0.768484931	0.000104525	0.102927792	0.51939211	0.093034329	0.647544752
549	W903_RS02695	W903_0553	478280	479170	NA	binding--dependent transport system inner membrane component family protein	0.549729489	0.458894815	2.351053604	7.49184E-12	-0.855390756	0.003126577
550	W903_RS02700	W903_0554	479184	480017	NA	binding--dependent transport system inner membrane component family protein	0.934600083	0.1233448	1.993391003	1.85437E-13	-1.18353019	0.000000971
551	W903_RS02705	W903_0555	480027	482228	NA	glycosyl hydrolases 31 family protein	0.797449124	0.012080109	1.651874628	2.29455E-22	-0.711781857	0.0000814
552	W903_RS02710	W903_0556	482318	483490	galK	galactokinase	1.007620157	0.006936004	1.643528593	3.30874E-10	-0.409385977	0.056836982
553	W903_RS02715	W903_0557	483500	484984	galT	galactose-1-phosphate uridylyltransferase	0.812628166	0.039699193	1.432144731	6.76386E-14	-0.581841783	0.004958473
554	W903_RS02720	W903_0558	484986	485981	NA	UDP-glucose 4-epimerase GalE	0.855810721	0.04827578	1.697373124	2.62894E-14	-0.670894711	0.002368218
555	W903_RS02725	W903_0559	486107	487108	NA	aldose 1-epimerase family protein	0.380614682	0.41083729	1.117675245	1.63971E-07	-0.521705935	0.022712374
556	W903_RS02730	W903_0560	487299	487553	NA	hypothetical protein	-1.172838854	1.95216E-09	-1.267690946	5.96489E-17	0.002554517	0.996076319
557	W903_RS02735	W903_0561	487662	487973	NA	hypothetical protein	-1.251948042	1.64751E-14	-1.267267462	5.98618E-24	-0.604495196	0.008066194
558	W903_RS02740	W903_0562	488241	488810	NA	acetyltransferase family protein	1.093299541	8.3033E-13	-0.526424401	1.14548E-05	-0.25714707	0.112865322
559	W903_RS02745	W903_0563	488908	489492	NA	acetyltransferase family protein	1.017395107	3.09976E-10	-0.474316949	0.000185505	-0.028840446	0.904281083
560	W903_RS02750	W903_0564	489489	490055	NA	AAA domain protein	0.93441373	1.1975E-08	-1.135877994	2.56734E-16	1.121881705	2.19E-08
561	W903_RS02755	W903_0565	490055	492706	valS	valine--tRNA ligase	1.15701368	1.17327E-16	-1.389050413	4.69686E-33	1.404665496	7.18E-19
562	W903_RS02760	W903_0566	492787	493767	NA	glycerophosphoryl diester phosphodiesterase family protein	-1.020121347	5.65552E-08	0.011081539	0.946381898	0.022065869	0.937434812
563	W903_RS02765	W903_0567	493775	494299	NA	putative membrane protein	-0.646640294	0.018608585	0.395395181	0.024340504	-0.046358937	0.900482041
564	NA	W903_0568	494667	494822	NA	hypothetical protein	-0.270237401	0.327552535	0.453368625	0.006871657	0.889540342	0.000490656
565	NA	W903_0569	494842	495009	NA	glycerophosphoryl diester phosphodiesterase family protein	-0.976238392	0.037178662	0.471924617	0.157366473	0.823507662	0.151233379
566	W903_RS02770	W903_0570	495107	496066	NA	oxidoreductase , NAD-binding Rossmann fold family protein	-0.169308088	0.331233356	0.822914889	2.93254E-13	-0.16927311	0.213871802
567	W903_RS02775	W903_0571	496227	497129	NA	corA-like Mg2+ transporter family protein	-0.170474092	0.445570203	1.771937609	2.42256E-50	1.057880059	1.91E-14
568	W903_RS02780	W903_0572	497290	498354	NA	hypothetical protein	0.35505758	0.12356904	0.94547938	1.7359E-11	-0.872561482	0.000000259
569	W903_RS02785	W903_0573	498470	499462	asnA	aspartate--ammonia ligase	0.371984985	0.05130082	-0.253945946	0.011524372	-0.319248131	0.064245797
570	W903_RS02790	W903_0574	499507	499875	NA	hypothetical protein	-0.032936966	0.890009272	0.226214476	0.049102066	-0.177896783	0.445783091
571	W903_RS02795	W903_0575	500087	500626	NA	RNA methyltransferase, RsmD family	-0.095768887	0.590511184	-0.048886688	0.643701007	-0.07142588	0.654522375
572	W903_RS02800	W903_0576	500638	500928	NA	putative membrane protein	-0.717602126	3.38407E-05	-0.352883632	0.002157577	-0.067098043	0.742757559
573	W903_RS02805	W903_0577	500925	501410	coaD	pantetheine-phosphate adenylyltransferase	-0.186956506	0.274774767	-0.084159407	0.42755962	-0.036084076	0.848659707
574	W903_RS02810	W903_0578	501400	502473	NA	lon protease (S16) C-terminal proteolytic domain protein	-0.406445943	0.00814957	-0.696240483	6.2004E-13	-0.015832904	0.932578174
575	W903_RS02815	W903_0579	502671	503882	NA	5'-nucleotidase, C-terminal domain protein	0.266121231	0.136213354	0.798077074	2.82181E-15	-0.0216838	0.905991133
576	W903_RS02820	W903_0580	503952	504530	NA	hypothetical protein	0.460165733	0.003153271	0.139541258	0.182229127	-0.024157619	0.904814014
577	W903_RS02825	W903_0581	504581	505687	rlmN	23S rRNA methyltransferase	0.603107424	8.61363E-05	-0.233012815	0.059361285	-0.261880433	0.070551588
578	W903_RS02830	W903_0582	505687	506202	NA	vanZ like family protein	0.890224022	4.60839E-05	0.062721694	0.77640213	-0.360575301	0.12734839
579	W903_RS02835	W903_0583	506387	508132	NA	ABC transporter family protein	0.362504597	0.138091152	-0.115440514	0.32523653	-0.713217227	0.000000159
580	W903_RS02840	W903_0584	508122	509861	NA	ABC transporter family protein	0.208409961	0.43371711	-0.374629159	0.000425709	-0.501155631	0.000328367
581	W903_RS02845	W903_0585	509989	510555	NA	glutamine amidotransferase of anthranilate synthase/aminodeoxychorismate synthase family protein	-0.014650905	0.961982117	-0.091121581	0.647122672	-0.577048009	0.015426358
582	W903_RS02850	W903_0586	510622	511161	NA	biotin transporter BioY	0.583956098	0.126093455	-0.930597554	0.003772281	-1.999079255	0.00000353
583	W903_RS02855	W903_0587	511162	512154	bioB	biotin synthase	0.463228248	0.14610702	0.045394922	0.879707266	-1.602383891	0.0000844
584	W903_RS02860	W903_0588	512279	512794	NA	hypothetical protein	0.794435808	0.027719201	0.605230961	0.037889125	-1.133532515	0.024061031
585	W903_RS02865	W903_0589	512784	513899	NA	acetyl-CoA C-acetyltransferase family protein	1.253269125	7.27072E-07	0.372784065	0.065808305	-0.849397642	0.014347086
586	W903_RS02870	W903_0590	513892	515121	NA	AMP-binding enzyme family protein	0.561851287	0.069270068	0.654527002	0.000499994	-1.164734129	0.000172544
587	W903_RS02875	W903_0591	515234	515866	nth	endonuclease III	0.27836378	0.164668168	1.035464129	7.39198E-13	0.423521996	0.059504715
588	W903_RS02880	W903_0592	515867	516256	NA	type IV leader peptidase family protein	-0.803093599	NA	1.255963196	0.111511931	0.502921254	0.654522375
589	W903_RS02885	W903_0593	516417	516626	NA	hypothetical protein	-0.586508533	0.014369568	-0.805524581	1.29422E-06	-0.037329061	0.917475792
590	W903_RS02890	W903_0594	516623	517591	NA	ROK family protein	0.271833835	0.088864179	-0.268765431	0.005211136	-0.004103079	0.982093165
591	W903_RS02895	W903_0595	517603	517983	NA	rhodanese-like domain protein	0.60286342	0.000332729	-0.116891469	0.326112248	-0.120567266	0.4499439
592	W903_RS02900	W903_0596	518215	520056	typA	GTP-binding protein TypA/BipA	-0.239096819	0.139982028	-0.905361446	2.72312E-17	0.545684466	0.0000124
593	W903_RS02905	W903_0597	520101	520346	NA	hypothetical protein	0.361487829	0.108402596	-0.651641469	2.02961E-06	0.725858947	0.001437564
594	W903_RS02910	W903_0598	520476	521831	murD	UDP-N-acetylmuramoylalanine--D-glutamate ligase	0.193201041	0.257219764	-0.443021813	1.72572E-06	0.178140161	0.202722127
595	W903_RS02915	W903_0599	521834	522910	NA	glycosyltransferase family 28 C-terminal domain protein	0.168141993	0.328559663	-0.341098612	0.00101776	0.141592696	0.326816096
596	W903_RS02920	W903_0600	522914	524053	NA	cell division FtsQ family protein	-0.165581722	0.355685142	-0.530961386	1.22832E-06	-0.043098958	0.799795516
597	W903_RS02925	W903_0601	524325	525698	ftsA	cell division protein FtsA	0.188148684	0.252500145	-0.540146346	2.96226E-07	1.078891218	1.7E-18
598	W903_RS02930	W903_0602	525720	527000	ftsZ	cell division protein FtsZ	0.545311575	0.000130106	-0.487042783	1.3357E-07	0.987310744	5.04E-16
599	W903_RS02935	W903_0603	527009	527680	NA	alanine racemase, N-terminal domain protein	0.269350435	0.087925789	-0.45128445	7.20869E-05	1.072707971	7.64E-20
600	W903_RS02940	W903_0604	527692	528297	NA	hypothetical protein	0.439670869	0.005401446	-0.438389057	2.94036E-06	0.586190016	0.00000349
601	W903_RS02950	W903_0605	528556	529344	NA	S4 domain protein	-0.023685241	0.892365276	-0.267090748	0.00320721	0.414451018	0.0015135
602	W903_RS02955	W903_0606	529354	530124	NA	divIVA domain protein	-0.391331683	0.009367493	-0.890284641	2.19947E-20	0.389323979	0.007195244
603	W903_RS02960	W903_0607	530409	533201	ileS	isoleucine--tRNA ligase	0.48474049	0.000853006	-1.672939204	1.63828E-47	1.169925725	3.27E-12
604	W903_RS02965	W903_0608	533318	533620	NA	hypothetical protein	-0.526899286	0.009569687	-0.652128622	2.7883E-11	0.299865074	0.071296035
605	W903_RS02970	W903_0609	533684	534139	NA	NUDIX domain protein	-0.907680946	1.17678E-06	-1.264927942	5.50806E-28	0.20670489	0.271299635
606	W903_RS02975	W903_0610	534325	536586	NA	istB-like ATP binding family protein	0.580895212	0.000275248	1.427800195	1.14165E-18	0.835709976	1.02E-12
607	W903_RS02980	W903_0611	536884	537114	NA	hypothetical protein	-0.837192979	0.004795017	1.135739379	0.00030638	0.482622248	0.48384832
608	W903_RS02985	W903_0612	537255	537947	NA	amino ABC transporter, permease , 3-TM region, His/Glu/Gln/Arg/opine family domain protein	0.255519279	0.206759631	0.327589497	0.0242945	0.362132561	0.168793592
609	W903_RS02990	W903_0613	537940	538674	NA	ABC transporter family protein	-0.233857557	0.26326899	-0.164672704	0.249518326	0.429077329	0.09224362
610	W903_RS02995	W903_0614	538960	540654	NA	phosphoglucomutase/phosphomannomutase, C-terminal domain protein	-0.163036657	0.418459993	-0.920938312	4.21822E-10	0.014369248	0.934878411
611	W903_RS03000	W903_0615	540793	541647	NA	tetrahydrofolate dehydrogenase/cyclohydrolase, catalytic domain protein	-0.184724219	0.293839545	-0.977076865	3.11773E-19	0.676864015	0.0000134
612	W903_RS03005	W903_0616	541644	542480	NA	hypothetical protein	-0.008694322	0.964761157	-0.524874523	5.29168E-06	0.655073569	0.000170902
613	W903_RS03010	W903_0617	542606	543946	xseA	exodeoxyribonuclease VII, large subunit	0.126846395	0.495327691	0.101235359	0.343131155	0.177242484	0.20803063
614	W903_RS03015	W903_0618	543924	544139	xseB	exodeoxyribonuclease VII, small subunit	0.293348488	0.331233356	-0.210451783	0.300594877	0.452433409	0.075555608
615	W903_RS03020	W903_0619	544139	545011	NA	polyprenyl synthetase family protein	-0.051879243	0.780671567	0.028969135	0.814047485	0.176401127	0.219867757
616	W903_RS03025	W903_0620	545004	545831	NA	hemolysin TlyA family protein	0.078487792	0.705759832	-0.109273596	0.309264296	-0.004768683	0.980285285
617	W903_RS03030	W903_0621	545818	546291	NA	arginine repressor, DNA binding domain protein	-0.360026667	0.037886466	-0.52674463	1.57609E-06	0.265336675	0.074036078
618	W903_RS03035	W903_0622	546303	547961	recN	DNA repair protein RecN	-0.195736737	0.278351491	-0.409264179	2.56312E-05	0.041593488	0.788746689
619	W903_RS03040	W903_0623	548074	548910	NA	degV family protein	-0.115380378	0.549536178	-0.182410587	0.084994463	0.175513766	0.227680448
620	W903_RS03045	W903_0624	548978	549742	NA	GDSL-like Lipase/Acylhydrolase family protein	-0.194363146	0.300532643	-0.38929555	0.001044907	0.015335611	0.934157167
621	W903_RS03050	W903_0625	549717	550319	NA	hypothetical protein	-0.155903409	0.549536178	-0.18277579	0.168966153	0.177248025	0.402183341
622	W903_RS03055	W903_0626	550422	550697	hup	DNA-binding protein HU	-0.821413266	1.21209E-08	-0.875741976	1.8222E-21	1.174461065	2.1E-14
623	W903_RS03060	W903_0627	550785	551927	NA	phage integrase family protein	-0.218464876	0.286334644	-0.364387356	0.026161579	0.56413016	0.0000317
624	W903_RS03065	W903_0628	552042	552593	NA	hypothetical protein	0.551148763	0.004296654	-1.74104652	1.89006E-45	-0.603979954	0.0000266
625	W903_RS03070	W903_0629	552611	552997	NA	hypothetical protein	0.245290989	0.357011977	-0.909613962	3.54277E-10	-0.258003264	0.158675232
626	W903_RS03075	W903_0630	553001	553348	NA	helix-turn-helix family protein	-0.200750119	0.354573027	-1.000545436	3.08663E-09	-0.023437178	0.911458122
627	W903_RS03080	W903_0631	553645	553890	NA	glycerophosphoryl diester phosphodiesterase family protein	-1.046626844	1.32701E-06	-1.186649552	3.71793E-06	-1.30871474	3.15E-09
628	W903_RS03085	W903_0632	553841	554629	NA	putative phage membrane protein	-1.396524593	4.60599E-17	-1.225567305	2.8338E-25	-1.311083653	2.75E-20
629	W903_RS03090	W903_0633	554680	554871	NA	cro/C1-type HTH DNA-binding domain protein	-1.854164516	6.18138E-22	-1.746252835	1.45369E-22	-1.778199179	3.35E-24
630	W903_RS03095	W903_0634	554951	555262	NA	putative excisionase	-1.57173312	4.22581E-19	-1.141913693	7.97507E-15	-1.529045376	5.14E-22
631	NA	W903_0635	555267	555416	NA	hypothetical protein	-0.802800732	0.105367014	-1.088092283	0.028584335	-1.453763142	0.000250532
632	W903_RS03100	W903_0636	555410	555637	NA	hypothetical protein	-1.247982546	1.07998E-06	-0.842070856	4.60579E-05	-1.153732672	7.18E-09
633	W903_RS03105	W903_0637	555630	555860	NA	hypothetical protein	-1.466355516	4.73922E-08	-1.443726808	4.14108E-09	-1.501375792	8.03E-12
634	W903_RS03110	W903_0638	555844	557163	NA	AAA ATPase domain protein	-1.256559904	1.55448E-16	-1.41076039	3.8201E-43	-1.807624753	6.99E-46
635	W903_RS03115	W903_0639	557178	558272	NA	AAA domain protein	-0.857003873	1.17178E-08	-0.985533914	5.52074E-21	-1.664203141	3.54E-39
636	W903_RS03120	W903_0640	558312	558734	NA	putative phage protein	-1.20651038	1.17255E-14	-1.203507774	4.49658E-23	-1.593802091	4.93E-32
637	W903_RS03125	W903_0641	558736	559470	NA	putative phage protein	-1.028808397	2.00645E-10	-1.009525973	1.62849E-17	-1.833149483	5.8E-46
638	W903_RS03130	W903_0642	559491	560096	NA	hypothetical protein	-0.875157613	2.47168E-07	-0.930058018	7.92515E-14	-1.918086681	1.06E-45
639	W903_RS03135	W903_0643	560096	560704	NA	hypothetical protein	-1.292145307	5.00108E-17	-1.142106116	2.59777E-18	-1.642943714	2.65E-28
640	W903_RS03140	W903_0644	560749	562293	NA	DEAD/DEAH box helicase family protein	-0.969479725	9.44787E-10	-1.116865638	3.30637E-26	-1.921253832	1.4E-49
641	W903_RS03145	W903_0645	562302	562499	NA	glycerophosphoryl diester phosphodiesterase family protein	-2.187713904	1.44649E-07	-1.87222481	0.000329916	-1.969973325	0.0000327
642	W903_RS03150	W903_0646	562492	562743	NA	glycerophosphoryl diester phosphodiesterase family protein	-0.958971195	6.19034E-07	-1.260151765	1.64502E-18	0.667447036	0.000355623
643	W903_RS03155	W903_0647	562814	565093	NA	AAA domain protein	-1.466397549	4.79176E-24	-1.278392067	8.60786E-36	-2.151710593	6.35E-63
644	W903_RS03160	W903_0648	565455	565673	NA	putative rNA polymerase sigma factor	-1.645399761	1.85767E-11	-1.358994844	1.79581E-07	-1.764334619	2.9E-15
645	W903_RS03165	W903_0649	565666	566061	NA	endodeoxyribonuclease RusA family protein	-1.134688623	1.38088E-09	-1.210379343	3.40507E-16	-1.53712435	7.76E-21
646	W903_RS03170	W903_0650	566058	566273	NA	hypothetical protein	-1.066045369	0.04836609	-0.365942849	0.353519471	-1.760179802	0.0000101
647	W903_RS03175	W903_0651	566270	566506	NA	hypothetical protein	-1.667159556	1.56493E-20	-1.554179685	1.6555E-24	-1.577888882	8.86E-24
648	W903_RS03185	W903_0652	566813	567226	NA	phage transcriptional regulator, RinA family protein	-1.255035874	5.62443E-07	-1.226396242	2.26918E-07	-1.499318144	1.48E-12
649	W903_RS03190	W903_0653	567401	568153	NA	hypothetical protein	-2.291504639	1.37401E-49	-2.659271226	1.2795E-117	0.372328233	0.018983684
650	W903_RS03195	W903_0654	568207	568638	NA	terminase, small subunit	-2.55348602	2.31427E-25	-2.16844919	5.08707E-30	-0.160916215	0.593610168
651	W903_RS03200	W903_0655	568628	569908	NA	phage terminase, large subunit, PBSX family	-2.447463919	9.60013E-43	-2.113661063	6.28822E-44	-0.361418573	0.050577488
652	W903_RS03205	W903_0656	569986	571452	NA	phage portal protein, SPP1 family	-2.65852612	1.1325E-57	-2.391251708	9.72425E-92	-0.458989519	0.002237384
653	W903_RS03210	W903_0657	571418	572860	NA	phage head morphogenesis , SPP1 gp7 family domain protein	-2.339141739	1.18227E-50	-2.660782904	1.93606E-91	-0.539646573	0.000105909
654	NA	W903_0658	572960	573076	NA	putative membrane protein	-0.389123998	NA	0.777477434	0.199593713	-1.36330535	0.000188081
655	W903_RS03220	W903_0659	573081	573284	NA	hypothetical protein	-2.710837645	3.37838E-39	-2.940864446	4.57082E-49	-0.282135067	0.207310359
656	W903_RS03225	W903_0660	573427	573996	NA	hypothetical protein	-2.362052782	9.98927E-32	-3.020387156	6.0592E-83	-0.546478885	0.000149987
657	W903_RS10740	W903_0661	574015	574911	NA	putative head protein	-2.403742096	3.53172E-50	-3.135128863	2.4077E-124	-0.351936872	0.01334524
658	W903_RS03235	W903_0662	574917	575273	NA	phage gp6-like head-tail connector family protein	-2.625619921	5.92035E-31	-3.254316262	4.2104E-103	-0.261351181	0.10042923
659	W903_RS03240	W903_0663	575284	575562	NA	hypothetical protein	-2.322938881	1.39141E-25	-3.021561729	1.38981E-52	-0.401064317	0.021955879
660	W903_RS03245	W903_0664	575577	575903	NA	hypothetical protein	-2.573295639	4.7313E-32	-3.038477218	2.32772E-55	-0.615862719	0.005665076
661	W903_RS03250	W903_0665	575911	576270	NA	putative phage protein	-2.709893324	6.63388E-47	-3.170578213	4.1075E-112	-0.432304173	0.013283757
662	W903_RS03255	W903_0666	576282	576914	NA	phage major tail protein, TP901-1 family	-2.636292389	8.52271E-49	-2.982285768	2.5008E-100	-0.5399511	0.0000843
663	W903_RS03260	W903_0667	576965	577420	NA	phage family protein	-2.384059335	6.95819E-32	-2.573189108	1.71489E-73	-0.72514683	0.000000442
664	W903_RS03265	W903_0668	577495	577725	NA	hypothetical protein	-2.662628593	5.28866E-46	-2.87263206	3.3373E-128	-0.557552269	0.000181065
665	W903_RS03270	W903_0669	577754	581980	NA	tape measure domain protein	-2.315774545	2.02315E-53	-2.411438443	5.0479E-121	-1.081932905	3.69E-20
666	W903_RS03275	W903_0670	581993	582835	NA	phage tail family protein	-2.965951053	1.9711E-74	-2.785050193	8.9799E-140	-0.96281227	3.53E-12
667	NA	W903_0671	582848	584356	NA	prophage endopeptidase tail family protein	-2.850566794	7.64589E-75	-2.500190956	3.57751E-92	-0.955687898	2.87E-13
668	NA	W903_0672	584404	586674	NA	gp58-like family protein	-2.648777944	8.25563E-60	-2.591293641	3.2112E-119	-1.032320418	1.29E-15
669	NA	W903_0673	586683	586835	NA	glycerophosphoryl diester phosphodiesterase family protein	-1.756736365	2.69457E-11	-2.079138378	1.56751E-20	-1.175484153	8.86E-09

670	W903_RS03290	W903_0674	586846	587262	NA	hypothetical protein	-2.40319147	5.99265E-36	-2.531051734	2.96133E-51	-1.293142069	3.22E-15
671	NA	W903_0675	587325	587486	NA	hypothetical protein	-2.907780115	6.52619E-27	-2.83464243	2.04959E-36	-1.291402633	4.23E-08
672	W903_RS03295	W903_0676	587496	587786	NA	putative phage protein	-2.744560834	3.82561E-35	-2.783670594	1.79593E-93	-1.162379666	8.18E-14
673	W903_RS03300	W903_0677	587788	588039	NA	holin, phage phi LC3 family	-2.549500832	1.54757E-51	-2.4951233	5.72478E-39	-1.130718595	7.79E-17
674	W903_RS03310	W903_0678	588159	589352	NA	mannosyl-glycoendo-beta-N-acetylglucosaminidase family protein	-2.07737032	2.47868E-30	-2.492407057	2.7431E-104	-1.355489923	1.06E-22
675	W903_RS03315	W903_0679	589760	590233	NA	hypothetical protein	-1.205455497	1.98798E-13	0.510095681	1.89487E-06	-0.441568297	0.001352334
676	W903_RS03320	W903_0680	590360	590560	NA	csbD-like family protein	-0.851895606	0.064799054	0.476874604	0.335698455	0.795768294	0.072167233
677	NA	W903_0681	590601	590771	NA	putative nicotinate phosphoribosyltransferase	-1.17777237	0.067096287	-0.277684316	0.531434549	0.247179729	0.474469468
678	W903_RS03325	W903_0682	591191	591370	NA	hypothetical protein	-0.938974491	6.49819E-06	0.61196396	0.000821709	-0.903749157	0.0000545
679	W903_RS03330	W903_0683	591776	591973	NA	hypothetical protein	-0.949032577	0.206759631	0.145852535	0.739545253	-0.086178317	0.884601812
680	W903_RS03335	W903_0684	592017	592949	NA	putative dihydroorotate dehydrogenase	-0.370962377	0.616449631	-2.48248906	1.6064E-116	0.271292326	0.085739107
681	W903_RS03340	W903_0685	593135	594370	NA	femAB family protein	0.436817732	0.01342991	0.519486059	1.27694E-07	-0.511772629	0.000115259
682	W903_RS03345	W903_0686	594389	595600	NA	femAB family protein	0.384880364	0.015250379	0.586288557	4.81631E-11	-0.603630366	0.00000236
683	W903_RS03350	W903_0687	595613	596833	NA	femAB family protein	0.233231906	0.160806264	0.71094476	4.55494E-13	-0.589549424	0.0000011
684	W903_RS03355	W903_0688	596833	597645	NA	putative hydrolase	0.465567121	0.00221873	0.849182492	3.99446E-19	-0.56400853	0.00000321
685	W903_RS03360	W903_0689	597716	599032	NA	HD domain protein	0.403815851	0.014621274	0.514388812	1.99366E-05	-0.332992696	0.007124201
686	W903_RS03365	W903_0690	599096	599494	NA	hypothetical protein	-0.99121986	3.50053E-08	0.426572238	0.001065317	-0.809210807	0.000000251
687	W903_RS03370	W903_0691	599838	602522	NA	calcium-translocating P-type ATPase, PMCA-type	0.025161919	0.885855408	-0.609864531	7.94272E-06	-0.51701863	0.000156756
688	W903_RS03375	W903_0692	602567	603427	NA	phosphoesterase family protein	-0.519636619	0.006633666	1.057764706	1.00056E-19	-1.192683117	1.82E-16
689	W903_RS03380	W903_0693	603579	605510	fbp	fructose-1,6-bisphosphatase class 3	0.130230055	0.578632939	1.777376146	3.3983E-52	-0.017676645	0.921457827
690	W903_RS03385	W903_0694	605600	606724	queG	epoxyqueuosine reductase	-0.453716947	0.026176266	1.656159125	8.43291E-62	0.186660835	0.178184739
691	W903_RS03390	W903_0695	606911	607891	prfB	peptide chain release factor 2	0.23980682	0.126321509	-0.549603919	8.8808E-10	0.40658427	0.002613789
692	W903_RS03395	W903_0696	607910	608602	ftsE	cell division ATP-binding protein FtsE	0.248088129	0.126747878	-0.379972928	6.3735E-05	0.329693998	0.012154581
693	W903_RS03400	W903_0697	608595	609515	NA	ftsX-like permease family protein	-0.020073371	0.909836741	-0.628579047	3.87196E-09	0.319109914	0.011532601
694	W903_RS03405	W903_0698	609568	610278	NA	alpha/beta hydrolase family protein	0.314815798	0.080799139	1.032660606	2.16697E-29	-0.41795001	0.000668806
695	W903_RS03410	W903_0699	610275	610910	NA	hypothetical protein	0.34623521	0.065377276	0.823642438	1.71515E-16	-0.409514959	0.001200318
696	W903_RS03415	W903_0700	611141	611905	NA	acetoin reductases family protein	-0.137064665	0.475889989	0.48908623	1.7155E-06	0.885570133	3.9E-12
697	W903_RS03420	W903_0701	612055	614520	NA	exonuclease, DNA polymerase III, epsilon subunit family domain protein	0.080224691	0.661507684	-0.15870886	0.125809507	-0.350524457	0.013297101
698	W903_RS03425	W903_0702	614615	615799	NA	degT/DnrJ/EryC1/StrS aminotransferase family protein	0.300395347	0.061982832	-0.101386248	0.308001055	-0.674080682	0.00000118
699	W903_RS03430	W903_0703	615820	617166	asnS	asparagine--tRNA ligase	-0.078298208	0.654620604	-0.803643522	1.00693E-18	-0.575710572	0.00000575
700	W903_RS03435	W903_0704	617206	617763	NA	hypothetical protein	-0.41165345	0.027939254	-0.728900043	1.72935E-06	-1.544436543	5.37E-21
701	W903_RS03440	W903_0705	617760	618743	NA	inosine-uridine preferring nucleoside hydrolase family protein	-0.537363165	0.000250615	-0.743460108	3.65351E-12	-1.501488218	5.06E-36
702	W903_RS03445	W903_0706	618988	619401	NA	osmC-like family protein	-0.72413227	0.000185571	-0.42317244	0.002341797	0.500624409	0.035715209
703	W903_RS03450	W903_0707	619555	620445	NA	P-loop ATPase family protein	0.578920819	0.000877752	-0.499784853	1.85188E-05	0.470858229	0.001076653
704	W903_RS03455	W903_0708	620442	621416	NA	hypothetical protein	0.844308418	2.52525E-07	0.075491512	0.547051688	0.024563104	0.886664735
705	W903_RS03460	W903_0709	621413	622324	NA	sporulation Regulator WhiA C terminal domain protein	0.308708459	0.057293238	0.003866388	0.973334254	-0.011022693	0.948463645
706	W903_RS03465	W903_0710	622339	623736	pepD	dipeptidase	0.323045155	0.0426321	-0.068121845	0.502091611	-0.213488048	0.103316645
707	W903_RS03470	W903_0711	623878	625398	NA	yodA lipocalin-like domain protein	-0.782828123	1.99237E-05	-1.801895815	2.17638E-17	-0.229575257	0.460545618
708	W903_RS03475	W903_0712	625510	625770	rpmE	ribosomal protein L31	-1.05618314	1.48836E-14	-0.852615391	1.01048E-16	0.152213343	0.358331931
709	W903_RS03480	W903_0713	625879	626814	NA	DHHA1 domain protein	0.206899004	0.287022861	0.495590809	1.36574E-05	-0.197946961	0.219302092
710	W903_RS03485	W903_0714	627080	628102	add	adenosine deaminase	0.075218343	0.806170452	-1.559596434	7.52714E-30	-0.126353858	0.593789934
711	W903_RS03490	W903_0715	628161	628604	NA	flavodoxin	-0.956739803	9.10932E-07	-0.437960314	2.40012E-05	-0.454858725	0.015977082
712	W903_RS03495	W903_0716	628681	628956	NA	chorismate mutase	-0.863879864	7.49766E-06	0.098116545	0.62029633	-0.532222787	0.009331767
713	W903_RS03500	W903_0717	628949	630145	NA	voltage gated chloride channel family protein	-0.294227	0.203693857	0.460251463	0.000154982	-0.759333353	0.000000186
714	W903_RS03505	W903_0718	630729	631076	rplS	ribosomal protein L19	-1.18838736	3.17084E-16	-0.766216129	1.05248E-10	0.880914127	0.00000087
715	W903_RS03510	W903_0719	631187	631258	tRNA	NA	1.747111785	NA	0.612434274	0.758020569	0.3640478	0.868645392
716	W903_RS03520	W903_0721	632093	632497	NA	transposase family protein	-0.919783469	1.00413E-06	1.328713583	1.26181E-27	0.049421068	0.837242922
717	W903_RS03525	W903_0722	632659	632859	NA	putative integrase	-1.116348073	3.45948E-09	1.29653463	2.5435E-24	0.05501932	0.801756766
718	W903_RS03530	W903_0723	632884	633141	tnpSth1	ISSth1, transposase (Orf2), IS3 family	-1.261396398	7.41255E-10	1.592862038	1.82199E-17	-0.128742949	0.507165042
719	W903_RS10310	W903_0724	633649	633810	NA	TM2 domain protein	0.476858352	0.29098286	3.011610275	2.55488E-41	0.116119538	0.815896551
720	W903_RS03535	W903_0725	634553	635830	NA	ftsX-like permease family protein	-1.299598172	7.32005E-14	0.37658312	0.001878773	0.825306028	0.000385867
721	W903_RS03540	W903_0726	635840	636496	NA	ABC transporter family protein	-0.726759213	0.00113149	0.610813801	3.69044E-05	-0.35661111	0.135732891
722	W903_RS03545	W903_0727	636496	637872	NA	ftsX-like permease family protein	-0.269134833	0.149383746	0.636741836	3.02247E-08	-0.310392533	0.088054415
723	W903_RS03550	W903_0728	637969	638622	NA	putative transcriptional activator CadC	-0.86417661	0.000118972	-0.348450892	0.0270274	-0.185199945	0.554081589
724	W903_RS03555	W903_0729	638619	639938	NA	HAMP domain protein	-1.174777275	5.00705E-10	-0.754345972	1.37799E-07	0.361562295	0.149688996
725	W903_RS03560	W903_0730	639990	640601	NA	integrase core domain protein	-0.408680777	0.075582676	0.351721995	0.018010755	-0.436414073	0.029982601
726	W903_RS03565	W903_0731	640815	641015	NA	csbD-like family protein	0.111134485	0.758765308	0.59730218	0.008090943	0.316942647	0.265425947
727	W903_RS10315	W903_0732	641057	641245	NA	hypothetical protein	-0.526558651	0.17335524	0.338511499	0.077240652	0.699670788	0.088624368
728	W903_RS03570	W903_0733	641670	642875	NA	cell cycle family protein	-0.521779406	0.000700963	0.267438828	0.006754369	-0.321525772	0.023771104
729	W903_RS03575	W903_0734	642994	643554	NA	HAD hydrolase, IA, variant 1 family protein	-0.718176083	2.42897E-06	1.403788222	5.20103E-40	0.558524377	0.000182981
730	W903_RS03580	W903_0735	643555	645507	gyrB	DNA gyrase, B subunit	-0.246809219	0.116374595	0.059878105	0.558635249	0.163099123	0.245120896
731	W903_RS03585	W903_0736	645601	647325	NA	septation ring formation regulator, EzrA family protein	-0.632784439	1.36352E-05	-0.814740774	1.02301E-16	0.135563075	0.391010415
732	W903_RS03590	W903_0737	647419	648060	serB	phosphoserine phosphatase SerB	0.543730505	0.00230222	-0.441045877	0.000505627	-0.471246204	0.018499587
733	W903_RS03595	W903_0738	648081	648566	NA	NUDIX domain protein	0.237070731	0.412459017	-0.179518295	0.383844701	-0.670657239	0.000299858
734	W903_RS03600	W903_0739	648579	649034	NA	hypothetical protein	-0.084880001	0.705759832	-0.267419265	0.04397493	-0.368484333	0.026433358
735	W903_RS03605	W903_0740	649232	650539	eno	phosphopyruvate hydratase	-0.077209541	0.681248828	-1.252777837	4.2941E-48	0.828580731	0.000000633
736	W903_RS03610	W903_0741	650647	651711	NA	DNA/RNA non-specific endonuclease family protein	-0.199489691	0.337668674	1.716504665	3.60922E-56	-0.845883199	8.46E-09
737	W903_RS03615	W903_0742	651940	653223	aroA	3-phosphoshikimate 1-carboxyvinyltransferase	0.438207322	0.008380055	0.539938503	3.70185E-09	-0.019621913	0.906214492
738	W903_RS03620	W903_0743	653216	653728	NA	shikimate kinase family protein	-0.226968284	0.311453557	0.219858143	0.060223356	-0.053984667	0.76114099
739	W903_RS03625	W903_0744	653785	655158	NA	cell envelope-related function transcriptional attenuator common domain protein	0.029468317	0.873551447	0.179867072	0.093460728	-0.159314942	0.258606505
740	W903_RS03630	W903_0745	655259	656620	NA	23S rRNA (uracil-5-)-methyltransferase RumA	-0.725564929	1.92147E-05	-1.144193926	8.89396E-28	-0.266780617	0.181726727
741	NA	W903_0746	657718	657894	NA	hypothetical protein	-0.65310255	0.015353759	1.847643387	2.6128E-56	-0.976537893	7.69E-11
742	NA	W903_0747	659098	659292	NA	putative succinyl-diaminopimelate desuccinylase	-0.194062528	NA	1.997429693	0.010902711	0.172696589	0.907463508
743	W903_RS03645	W903_0748	659278	659508	NA	hypothetical protein	0.621183999	NA	2.619475396	0.036458903	-1.955533393	0.408429528
744	W903_RS03650	W903_0749	659569	660720	NA	beta-lactamase family protein	-0.346382773	0.083411153	0.498092791	2.02719E-07	-1.722541787	1.11E-36
745	W903_RS03655	W903_0750	660920	661912	NA	ABC transporter family protein	-0.350703235	0.065391433	0.657520336	2.53782E-09	0.171531936	0.301190237
746	W903_RS03665	W903_0753	662736	663521	NA	ABC-2 transporter family protein	0.215967823	0.365440383	0.934837602	1.07135E-13	-0.313909565	0.042536298
747	NA	W903_0754	664317	664451	NA	glycerophosphoryl diester phosphodiesterase family protein	-0.142506179	0.769579939	-0.535063113	0.005184447	-1.029117743	0.000000423
748	W903_RS03675	W903_0755	664451	665299	NA	glycerophosphoryl diester phosphodiesterase family protein	-0.398512326	0.051557722	-1.011052642	2.62201E-17	-0.787423884	5.36E-09
749	W903_RS03680	W903_0756	665296	666018	NA	short chain dehydrogenase family protein	0.09689937	0.717464977	-0.907942051	1.56032E-19	-0.494489643	0.0000295
750	W903_RS03685	W903_0757	666011	666316	NA	phosphopantetheine attachment site family protein	-0.620284562	0.010113378	-1.623338358	6.08856E-57	-0.576713048	0.00000784
751	W903_RS03690	W903_0758	666333	666776	NA	fabA-like domain protein	0.529081378	0.010546432	-0.638473044	4.4074E-12	-0.878296951	2.47E-13
752	W903_RS03695	W903_0759	666766	667695	NA	ABC transporter family protein	0.158517662	0.521627441	-0.928820508	5.48077E-24	-0.826148661	1.54E-12
753	W903_RS03700	W903_0760	667688	668566	NA	ABC-2 type transporter family protein	0.102962645	0.646585966	-0.922683409	7.41462E-25	-0.701551512	1.54E-08
754	W903_RS03705	W903_0761	668563	670566	NA	putative cylE	-0.09934888	0.631284966	-0.754945133	1.51003E-19	-0.628630715	0.000000131
755	W903_RS03710	W903_0762	670563	671516	NA	aminomethyltransferase folate-binding domain protein	-0.20004506	0.277322139	-0.825166151	1.88214E-21	-0.515438698	0.0000313
756	W903_RS03715	W903_0763	671552	673708	NA	beta-ketoacyl synthase, C-terminal domain protein	0.143655038	0.398263862	-0.424951816	1.40947E-05	-0.825534894	1.15E-12
757	W903_RS03720	W903_0764	673713	674924	NA	UDP-glucoronosyl and UDP-glucosyl transferase family protein	-0.578400574	0.000120828	-0.622065394	1.48752E-09	-0.489589636	0.0000643
758	W903_RS03725	W903_0765	674893	675468	NA	hypothetical protein	-0.71587674	4.57745E-06	-0.786115754	1.63938E-15	-1.065960138	2.45E-20
759	W903_RS03730	W903_0766	675611	676264	NA	ftsX-like permease family protein	0.272057889	0.534175267	1.265309329	8.00323E-11	-0.424276339	0.038807247
760	W903_RS03735	W903_0767	676274	677296	NA	macB-like periplasmic core domain protein	-0.081040838	0.818227551	0.988266924	7.74687E-08	-1.120413436	1.53E-11
761	W903_RS03745	W903_0770	677711	678382	NA	ABC transporter family protein	0.091237018	0.802265072	0.989184372	4.54647E-07	-1.224755511	1.16E-13
762	W903_RS03755	W903_0773	680145	680930	NA	DNA/RNA non-specific endonuclease family protein	0.134921772	0.632735521	1.116396665	2.4878E-17	-0.392676937	0.019335517
763	W903_RS03760	W903_0774	681008	681646	NA	hypothetical protein	1.225023928	6.23008E-07	1.839533273	8.68815E-26	0.882989374	0.0000777
764	W903_RS03765	W903_0775	681863	682519	NA	ABC transporter family protein	-0.496792185	0.000831153	-1.047670152	1.8382E-29	2.379489713	4.66E-54
765	W903_RS03770	W903_0776	682516	683289	NA	hypothetical protein	-0.22221319	0.256969376	-0.781867091	4.14362E-12	1.950982374	1.69E-25
766	W903_RS03775	W903_0777	683453	684271	NA	hypothetical protein	-0.061244281	0.870685211	-0.026330781	0.903573839	-0.002064004	0.996252226
767	NA	W903_0778	684308	684877	NA	lysR substrate binding domain protein	0.188966262	0.430272975	1.088266462	9.32371E-18	0.47847207	0.002847807
768	NA	W903_0779	685111	685365	NA	hypothetical protein	0.129739315	0.755212804	1.281356784	3.67861E-10	0.262735465	0.433134212
769	W903_RS03785	W903_0780	685385	686443	NA	phosphotransferase system, EIIC family protein	0.906720695	6.88E-05	0.875848502	4.56819E-11	-0.177424384	0.377320454
770	W903_RS03790	W903_0781	686457	687449	ldhD	D-lactate dehydrogenase	1.001049557	0.000314325	0.630648064	0.000240243	0.008233791	0.974193168
771	W903_RS03795	W903_0782	687657	689207	NA	MFS/sugar transport family protein	-0.661796068	0.077950145	-0.356370516	0.258066643	-0.747852112	0.008472562
772	W903_RS03800	W903_0783	689274	690299	NA	pfkB carbohydrate kinase family protein	0.699436377	0.280296301	1.836607117	2.32049E-06	-0.596014303	0.107586976
773	W903_RS03805	W903_0784	690316	692115	uidA	beta-glucuronidase	0.823984996	0.000141854	0.466734916	0.002212106	-0.047284386	0.837242922
774	W903_RS03810	W903_0785	692144	692815	NA	bacterial regulatory s, gntR family protein	0.442216972	0.016899212	-0.808357048	2.73264E-12	1.000874601	3.95E-08
775	W903_RS03815	W903_0786	692932	693549	NA	2-dehydro-3-deoxyphosphogluconate aldolase/4-hydroxy-2-oxoglutarate aldolase family protein	0.158778246	0.648392027	1.823687513	6.01275E-33	-0.626566239	0.000058
776	W903_RS03820	W903_0787	693566	694966	NA	glucuronate isomerase family protein	0.50389426	0.039791776	2.192369376	1.55827E-60	-0.805332073	0.000000019
777	W903_RS03825	W903_0788	694984	696030	uxuA	mannonate dehydratase	0.855734079	0.000331854	1.805694864	3.72262E-37	-0.742633365	0.000000392
778	W903_RS03830	W903_0789	696153	696992	NA	hypothetical protein	0.783279261	0.028426092	2.175954834	1.6303E-33	-0.978828903	7.18E-08
779	W903_RS03835	W903_0790	697145	697957	NA	HAD hydrolase, IA, variant 1 family protein	1.023801537	0.000104671	1.899649723	1.83187E-38	-0.802775388	0.0000941
780	W903_RS03840	W903_0791	697973	699763	NA	glycosyl hydrolase family 3 N terminal domain protein	0.731880501	0.000623182	1.657708486	4.1961E-37	-0.571767208	0.00076298
781	W903_RS03845	W903_0792	699820	700905	NA	metallopeptidase M24 family protein	-0.038148081	0.828736007	0.114912312	0.20883586	0.121751957	0.412636601
782	W903_RS03850	W903_0793	701115	702119	ccpA	catabolite control protein A	0.099689236	0.593049584	-0.088178173	0.549881366	0.680304024	0.000000124
783	W903_RS03855	W903_0794	702251	703717	NA	alpha amylase, catalytic domain protein	0.595592313	0.000168817	0.416432977	3.1953E-05	0.427318932	0.000860862
784	W903_RS03860	W903_0795	703762	704760	NA	glycosyl transferases group 1 family protein	0.086883264	0.643341349	0.86225215	2.35438E-22	-0.029274056	0.858480935
785	W903_RS03865	W903_0796	704762	706096	NA	glycosyl transferases group 1 family protein	-0.173499561	0.307346492	0.60055029	7.76645E-11	-0.169939372	0.204784941
786	W903_RS03870	W903_0797	706553	708496	thrS	threonine--tRNA ligase	0.337369096	0.023316963	-1.725054709	4.35731E-30	2.040651074	1.29E-33
787	W903_RS03875	W903_0798	708805	709422	NA	putative transcriptional activator CadC	-1.236078467	2.05901E-11	0.352987754	0.001844484	0.818600419	0.00000116
788	W903_RS03880	W903_0799	709520	710539	NA	hypothetical protein	-0.210704637	0.313877608	0.502167488	5.77906E-06	0.07889467	0.654568205
789	W903_RS03885	W903_0800	710706	711272	NA	peptidase propeptide and YPEB domain protein	-1.310636317	2.69047E-07	0.707224295	1.16413E-05	-0.08327548	0.724791933
790	W903_RS03890	W903_0801	711321	711971	NA	amino ABC transporter, permease , 3-TM region, His/Glu/Gln/Arg/opine family domain protein	-1.792446893	1.10415E-29	0.312619128	0.213891993	0.156166571	0.351708164
791	W903_RS03895	W903_0802	711983	712678	NA	amino ABC transporter, permease , 3-TM region, His/Glu/Gln/Arg/opine family domain protein	-1.144944262	1.17178E-08	0.8308668	5.83061E-05	-0.278452608	0.065092776
792	W903_RS03900	W903_0803	712691	713491	NA	bacterial extracellular solute-binding s, 3 family protein	-1.36812966	2.61125E-11	0.71670879	0.001391575	-0.37091745	0.010345785
793	W903_RS03905	W903_0804	713503	714258	NA	ABC transporter family protein	-1.253140059	2.02543E-08	1.089658465	1.8694E-06	-0.438157573	0.007997953
794	W903_RS03910	W903_0805	714598	715308	rr02	response regulator	0.408533606	0.018399558	-0.099116111	0.354588167	0.350914758	0.014238729
795	W903_RS03915	W903_0806	715301	716650	NA	sensory box protein	0.572655208	0.000136573	0.191056779	0.046904711	-0.0835436	0.573112535
796	W903_RS03920	W903_0807	716654	717463	NA	metallo-beta-lactamase superfamily protein	0.509386239	0.000947601	0.351538085	0.000469585	-0.359833659	0.004965513
797	W903_RS03925	W903_0808	717466	717834	NA	hypothetical protein	1.323994332	8.02534E-12	0.905979416	7.59477E-10	-0.843508256	1.25E-08
798	W903_RS03930	W903_0809	718010	718696	rnc	ribonuclease III	-0.108267318	0.565005127	-0.358375548	0.075744133	0.975881809	2.11E-09
799	W903_RS03935	W903_0810	718704	722243	smc	chromosome segregation protein SMC	-0.239358599	0.137687003	-0.410840488	0.005518727	0.490340623	0.000165399
800	W903_RS03940	W903_0811	722334	723131	NA	HAD hydrolase, IIB family protein	-0.202563556	0.266437751	0.776613796	1.12844E-06	-0.409142898	0.004072973
801	W903_RS03945	W903_0812	723131	723955	NA	HAD hydrolase, IIB family protein	0.259591847	0.129946656	1.094944617	4.12414E-27	-0.33160828	0.011179727
802	W903_RS03950	W903_0813	723955	725565	ftsY	signal recognition particle-docking protein FtsY	0.96214283	3.45372E-11	0.631630672	5.35523E-07	-0.36769384	0.006937188
803	W903_RS03955	W903_0814	725602	726414	NA	hypothetical protein	0.097296184	0.639428324	-0.266772848	0.008848499	-0.182690774	0.288890891

804 W903_RS03960	W903_0815	726414	727316	NA	putative permease family protein	-0.395039009	0.017118302	0.031566669	0.771729787	-0.258764868	0.106790922
805 W903_RS03965	W903_0816	727322	727450	NA	glycerophosphoryl diester phosphodiesterase family protein	-0.447015159	0.073270666	0.326614276	0.075192509	0.642192937	0.18283691
806 W903_RS03970	W903_0817	727583	728626	NA	luciferase-like monooxygenase family protein	0.123671072	0.832394958	3.030838756	2.48687E-37	2.888158907	3.8E-46
807 W903_RS03975	W903_0818	728732	730894	NA	S1 RNA binding domain protein	0.14705895	0.376532116	0.206120073	0.072190635	-0.108207721	0.440796011
808 W903_RS03980	W903_0819	730908	731315	NA	sprT-like family protein	0.197313965	0.348200027	0.224604867	0.124203578	-0.15274179	0.359667344
809 W903_RS03985	W903_0820	731396	731659	NA	pspC domain protein	0.690817635	0.000391694	1.368091358	7.6536E-20	0.465085823	0.016369095
810 NA	W903_0821	731637	731771	NA	hypothetical protein	0.98260001	4.81653E-05	1.283422732	5.14655E-07	2.091720961	8.6E-13
811 W903_RS03990	W903_0822	731887	732822	hprK	HPr(Ser) kinase/phosphatase	1.224389838	3.94157E-18	0.923077187	4.23028E-19	0.177032301	0.178899424
812 W903_RS03995	W903_0823	732815	733588	lgt	prolipoprotein diacylglyceryl transferase	1.332115349	2.45412E-21	0.898397952	4.77775E-18	-0.088479785	0.51199985
813 W903_RS04000	W903_0824	733603	734001	NA	hypothetical protein	0.279509218	0.086558854	0.066733835	0.606356145	0.141011764	0.29270162
814 W903_RS04005	W903_0825	733998	734429	NA	hypothetical protein	0.909333008	2.71044E-07	0.086497512	0.655322751	0.117535982	0.446338903
815 W903_RS04010	W903_0826	734470	734745	NA	hypothetical protein	-0.590017163	0.000536344	0.660525091	3.24451E-09	-1.090682349	4.43E-12
816 W903_RS04015	W903_0827	735087	736013	NA	peptidase U32 family protein	-0.026125035	0.914504482	-1.972667569	1.11389E-86	-0.42668725	0.007384743
817 W903_RS04020	W903_0828	736143	737429	NA	peptidase U32 family protein	0.169275337	0.46809963	-1.877735728	5.31282E-89	-0.338618004	0.021159253
818 W903_RS04025	W903_0829	737556	737768	NA	hypothetical protein	0.624600762	0.052097292	-1.236642408	1.98962E-05	-0.567461985	0.150976563
819 W903_RS04030	W903_0830	737892	738689	NA	putative membrane protein	-0.058732406	0.814729644	0.522805268	7.14483E-06	-0.218517012	0.138896779
820 W903_RS04035	W903_0831	738790	740130	NA	metal ion transporter, metal ion family protein	-0.062874206	0.734062253	0.30911799	0.00165815	-1.029700537	7.43E-14
821 W903_RS04040	W903_0832	740859	741968	ribD	riboflavin biosynthesis protein RibD	0.316076608	0.252500145	4.685798309	7.9708E-21	0.270032608	0.157870999
822 W903_RS04045	W903_0833	741949	742599	ribE	riboflavin synthase, alpha subunit	0.128379263	0.647719608	4.575524935	7.82067E-15	0.224206679	0.151497658
823 W903_RS04050	W903_0834	742617	743810	ribA	GTP cyclohydrolase II	0.020971443	0.933659036	4.243515478	3.2909E-19	0.34364935	0.01562914
824 W903_RS04055	W903_0835	743825	744295	ribH	6,7-dimethyl-8-ribityllumazine synthase	-0.254003612	0.277470661	4.042901573	3.31815E-24	0.155759741	0.36175722
825 W903_RS04060	W903_0836	744370	745860	lysS	lysine--tRNA ligase	0.138108546	0.423657793	-0.969292885	2.12494E-20	-0.199594112	0.25268107
826 W903_RS04065	W903_0837	746035	746937	NA	HAD hydrolase, IA, variant 1 family protein	-1.873198941	2.09743E-29	-1.484173793	1.42361E-22	0.086394934	0.64931477
827 W903_RS04070	W903_0838	746973	747614	NA	histidine phosphatase super family protein	0.360503955	0.047678813	0.140206962	0.248191356	0.185103954	0.215154311
828 W903_RS04075	W903_0839	747636	748109	NA	bacteriocin-type signal sequence domain protein	-0.069897911	0.740170779	-0.106350664	0.482226303	0.396158458	0.007413907
829 W903_RS04080	W903_0840	748404	749021	NA	NAD dependent epimerase/dehydratase family protein	-0.138080706	0.636323631	2.125776195	1.62578E-55	-0.432445778	0.066427252
830 W903_RS04085	W903_0841	749054	749902	NA	glycosyl hydrolases 25 family protein	-1.078881742	4.47528E-11	0.454129364	5.22116E-05	-0.108729686	0.564321397
831 W903_RS04090	W903_0842	750059	750583	NA	ECF-type riboflavin transporter, S component family protein	-0.229341516	0.278195052	0.577325002	5.17211E-05	-0.559206582	0.0210782
832 W903_RS04095	W903_0843	750567	750956	NA	putative lipoprotein	-0.540334155	0.003170788	-0.17262167	0.324269643	-0.517161967	0.022979299
833 W903_RS04100	W903_0844	751015	752814	NA	oligoendopeptidase F	0.539597295	0.003392532	-0.467447884	3.23259E-07	2.362727795	6.78E-59
834 W903_RS04105	W903_0845	753023	755818	NA	phosphoenolpyruvate carboxylase family protein	-0.371528687	0.037371044	0.086128497	0.39265141	0.392819982	0.002566017
835 W903_RS04110	W903_0846	756161	757192	NA	cell cycle family protein	0.121497935	0.549536178	0.180604537	0.106335863	0.394537146	0.030474805
836 W903_RS04115	W903_0847	757544	758740	tuf	translation elongation factor Tu	0.18734885	0.237745726	-1.079988749	2.48687E-37	1.02849347	6.71E-14
837 W903_RS04120	W903_0848	758921	759679	tpiA	triose-phosphate isomerase	0.12687315	0.437073844	-1.594378153	3.35753E-67	1.194098981	5.52E-13
838 W903_RS04125	W903_0849	759856	760548	NA	phosphoglycerate mutase 1 family protein	0.172219346	0.344073825	-1.717253266	6.46049E-68	1.268362467	1.48E-15
839 W903_RS04130	W903_0850	760681	760869	NA	phage integrase family protein	0.766540307	NA	1.5448946	0.057256852	-0.703882452	0.701811594
840 W903_RS04135	W903_0851	760984	761376	NA	phage integrase family protein	-2.059390653	NA	1.163522903	0.150585639	-0.435578164	0.714652882
841 NA	W903_0852	761549	762157	NA	glycerophosphoryl diester phosphodiesterase family protein	0.859567947	NA	2.395897755	0.002963539	0.418278534	0.749204283
842 NA	W903_0853	762413	762571	NA	glycerophosphoryl diester phosphodiesterase family protein	-0.802842705	NA	1.948360858	0.010470242	-0.354887036	0.789199622
843 W903_RS04145	W903_0854	763171	764094	NA	hypothetical protein	-0.794467367	6.80338E-06	-0.679120272	7.86496E-12	0.874306121	1.11E-08
844 W903_RS04150	W903_0855	764108	764659	NA	hypothetical protein	-0.697128706	0.004579459	-0.761243495	4.50839E-05	0.404560571	0.110521529
845 W903_RS04155	W903_0856	764974	767016	NA	penicillin binding transpeptidase domain protein	-0.022294801	0.893990343	0.018895362	0.885454913	-0.11303267	0.554081589
846 W903_RS04160	W903_0857	767031	767627	recR	recombination protein RecR	-0.011062449	0.955386747	-0.043740952	0.733215929	-0.533733856	0.014926233
847 W903_RS04165	W903_0858	767768	768814	ddl	D-alanine--D-alanine ligase	-0.385742313	0.015980682	-0.320986301	0.000626387	0.346481163	0.012662998
848 W903_RS04170	W903_0859	768961	770328	murF	UDP-N-acetylmuramoyl-tripeptide--D-alanyl-D-alanine ligase family protein	-0.584195997	8.55503E-05	0.291818003	0.002996617	-0.626618475	0.0000844
849 W903_RS04175	W903_0860	770516	771736	NA	major Facilitator Superfamily protein	0.267403593	0.274774767	0.35654911	0.009403192	-0.66225354	0.001289603
850 W903_RS04180	W903_0861	771865	772551	NA	integral membrane family protein	-1.197555698	3.00819E-14	0.079084883	0.556972826	0.246588401	0.132614013
851 W903_RS04190	W903_0864	774445	775989	prfC	peptide chain release factor 3	0.22198632	0.17915471	0.484481731	5.59505E-08	-0.240113158	0.070027772
852 W903_RS04195	W903_0865	776075	776455	NA	hypothetical protein	-0.468004993	0.013735518	0.013579824	0.931269524	-0.25650132	0.351562424
853 W903_RS04200	W903_0866	776649	777383	NA	ABC transporter family protein	-0.897465825	7.3538E-05	2.801229369	4.7841E-155	2.158624609	3.6E-67
854 W903_RS04205	W903_0867	777376	778038	NA	binding--dependent transport system inner membrane component family protein	-0.164777807	0.488081032	3.051246079	6.4904E-122	1.383616725	4.43E-27
855 W903_RS04210	W903_0868	778054	778884	NA	NLPA lipofamily protein	-0.627326089	0.009954694	2.225583009	4.98769E-59	1.066463391	7.15E-15
856 W903_RS04215	W903_0869	779119	780705	NA	helicase domain protein	-0.35626763	0.03638661	-2.221657152	3.01433E-76	2.24608607	8.81E-31
857 W903_RS04220	W903_0870	780890	781156	NA	GIY-YIG catalytic domain protein	0.673216395	0.014979291	0.044917228	0.86230103	-0.187836651	0.49024491
858 W903_RS04225	W903_0871	781149	781913	NA	methyltransferase domain protein	0.164390927	0.385202647	-0.612567971	3.01981E-07	-0.195331595	0.253124001
859 W903_RS04230	W903_0872	782048	782788	plsC	1-acyl-sn-glycerol-3-phosphate acyltransferase	-0.343692444	0.067561666	-0.227085308	0.051181591	-0.470480602	0.017539558
860 W903_RS04235	W903_0873	782887	783540	NA	competence ComEA helix-hairpin-helix repeat region domain protein	-0.151434491	NA	2.746797549	3.03472E-09	-0.485143862	0.442093868
861 W903_RS04240	W903_0874	783524	785761	NA	DNA internalization-related competence protein ComEC/Rec2	-0.259676788	0.285467335	1.088057002	1.92717E-17	-0.618722537	0.012058251
862 W903_RS04245	W903_0875	785887	786696	NA	HAD hydrolase, IIB family protein	-0.174658391	0.535808713	0.869934357	1.18905E-08	-0.817241841	0.000118038
863 W903_RS04250	W903_0876	786707	787651	NA	bacterial regulatory s, lacI family protein	0.467369266	0.043896081	1.303932212	3.9727E-17	-0.990864582	1.09E-09
864 W903_RS04255	W903_0877	787710	788702	NA	alpha/beta hydrolase fold family protein	0.952980523	1.35023E-05	2.10511535	3.84806E-87	-0.381181879	0.144736645
865 W903_RS04260	W903_0878	788877	789605	NA	methyltransferase domain protein	-0.213280693	0.446374739	0.702367382	7.91966E-07	-0.043562362	0.853078504
866 W903_RS04265	W903_0879	789659	790696	holA	DNA polymerase III, delta subunit	-0.807326836	8.1301E-07	1.02563183	5.41004E-25	-0.215809274	0.188805854
867 W903_RS04270	W903_0880	790776	791384	sodA	superoxide dismutase	-0.503032389	0.013077952	0.579715028	3.07149E-08	1.24115864	2.31E-16
868 W903_RS04275	W903_0881	791723	792574	licT	transcription antiterminator LicT	-0.023905541	0.914504482	2.116245743	1.61949E-70	-0.760589255	1.54E-09
869 W903_RS04280	W903_0882	792567	794435	NA	PTS system, beta-glucoside-specific IIABC component family protein	0.676901086	0.002573908	1.43235082	2.34928E-22	-0.552937713	0.005379462
870 W903_RS04285	W903_0883	794452	795879	NA	cellulase family protein	0.613255951	0.002303312	0.539552857	5.56628E-05	-0.730132248	0.000000186
871 W903_RS04290	W903_0884	795947	797041	NA	sugar diacid recognition family protein	-0.350068918	0.074564757	0.819806135	2.71834E-12	-0.143908921	0.492238969
872 W903_RS04295	W903_0885	797208	798350	NA	glycerate kinase family protein	0.307475349	0.346377236	0.915259299	0.000128038	-0.517221269	0.028488879
873 W903_RS04300	W903_0886	798375	799631	NA	citrate transporter family protein	0.237934893	0.497265329	0.950785469	9.00721E-05	-0.293257222	0.215869302
874 NA	W903_0887	800209	800796	NA	D-alanyl-D-alanine carboxypeptidase family protein	-0.644198715	0.000234013	0.137682786	0.389517383	-0.347131492	0.03078664
875 W903_RS10360	W903_0888	800851	801288	NA	marR family protein	0.163499842	0.40043987	-0.39131278	6.34074E-05	0.172698167	0.264437719
876 W903_RS04315	W903_0889	801375	802403	queA	tRNA ribosyltransferase-isomerase	0.609026171	8.03639E-05	-0.459513946	7.85122E-05	0.193249172	0.34804929
877 W903_RS04320	W903_0890	802556	803236	NA	VIT family protein	-0.424702931	0.086116923	-1.285226233	0.00219478	0.473147539	0.00136864
878 W903_RS04325	W903_0891	803387	804088	nagB	glucosamine-6-phosphate deaminase	-0.797368298	1.02679E-06	0.120494924	0.58148367	0.327467437	0.016790091
879 W903_RS04330	W903_0892	804160	805116	NA	glutathione S-transferase, C-terminal domain protein	-0.157013052	0.549536178	0.559716885	0.000460747	1.176726079	1.6E-15
880 W903_RS04335	W903_0893	805212	805931	NA	pseudouridine synthase family protein	-0.007294941	0.977276951	1.173978351	7.50621E-16	0.00174719	0.996076319
881 W903_RS04340	W903_0894	806004	807155	NA	major Facilitator Superfamily protein	-0.436044761	0.040928658	0.288907958	0.006393623	0.377161256	0.051737283
882 W903_RS04345	W903_0895	807207	808154	NA	competence CoiA-like family protein	0.559269905	0.267008777	1.86488977	7.83398E-11	0.594281932	0.196969847
883 W903_RS04350	W903_0896	808170	809975	NA	oligoendopeptidase F	0.679920891	6.37209E-06	-0.911381734	4.85788E-21	2.10318959	2.27E-32
884 W903_RS04355	W903_0897	810169	810795	NA	HAD hydrolase, IA, variant 1 family protein	0.206041909	0.323786712	-1.181255899	2.13537E-15	1.232838899	1.47E-08
885 W903_RS04360	W903_0898	810869	811576	NA	methyltransferase domain protein	-0.85099831	2.01201E-08	-1.06019632	5.91598E-25	1.070259496	0.000000123
886 W903_RS04365	W903_0899	811637	812566	NA	PPIC-type PPIASE domain protein	-0.291976576	0.069196501	-2.456231671	4.4712E-136	1.366924326	3.1E-25
887 W903_RS04370	W903_0900	812808	813293	NA	tubby C 2 family protein	0.319605157	0.05641049	-1.537315698	3.6479E-33	1.701437787	1E-21
888 W903_RS04375	W903_0901	813309	815927	alaS	alanine--tRNA ligase	0.608483543	1.80157E-05	-1.453017853	1.1199E-40	0.982864347	9.15E-12
889 W903_RS04380	W903_0902	816009	816725	NA	hypothetical protein	0.543414317	0.001717206	-0.64635599	3.56919E-06	0.300632825	0.133111163
890 W903_RS04385	W903_0903	816813	817631	NA	glycosyl transferase 8 family protein	-0.385640804	0.104469996	-0.573526169	7.16814E-05	0.337571974	0.181118891
891 W903_RS04390	W903_0904	817832	818020	NA	glycerophosphoryl diester phosphodiesterase family protein	0.808596764	0.165421465	2.598467587	2.17267E-12	-0.709346847	0.065259071
892 W903_RS04395	W903_0905	818055	818339	NA	hypothetical protein	0.085936628	0.860408926	2.516327272	1.88898E-17	-1.248636227	0.000249562
893 W903_RS04400	W903_0906	818384	818599	NA	helix-turn-helix family protein	0.440382766	0.574080165	1.539731819	0.003091065	-0.593648993	0.347525228
894 W903_RS04405	W903_0907	818602	819360	NA	hypothetical protein	0.258670512	0.648586851	2.47084304	1.80481E-11	-0.350407825	0.463353067
895 W903_RS04410	W903_0908	819445	820008	thiT	thiamine transporter ThiT	0.182907042	0.519354825	2.105923512	2.65045E-32	-1.20859769	1.35E-12
896 W903_RS04415	W903_0909	820249	821208	NA	ribonucleotide reductase, small chain family protein	-0.143734595	0.406936176	0.236586595	0.033451815	0.571414545	0.0000259
897 W903_RS04420	W903_0910	821411	823570	NA	ribonucleoside-diphosphate reductase, alpha subunit	0.101452533	0.549536178	0.62361703	6.27125E-08	0.534319502	0.00000865
898 W903_RS04425	W903_0911	823648	823872	nrdH	glutaredoxin-like protein nrdH	-0.389103678	0.058233186	1.185255559	6.77703E-16	1.100486922	0.00000264
899 W903_RS04430	W903_0912	824254	824517	ptsH	phosphocarrier protein HPr	-0.736971121	3.33987E-07	-0.731073704	1.12893E-12	0.304015581	0.02324368
900 W903_RS04435	W903_0913	824522	826255	ptsP	phosphoenolpyruvate-protein phosphotransferase	-0.311034953	0.040469854	-0.955647936	4.51502E-28	0.024393801	0.880701494
901 W903_RS04440	W903_0914	826405	827832	gapN	NADP-dependent glyceraldehyde-3-phosphate dehydrogenase	0.743243547	1.84679E-06	-0.701534439	5.92303E-15	0.855793089	6.27E-12
902 W903_RS04445	W903_0915	827972	829225	NA	polysaccharide deacetylase family protein	0.339478092	0.060262783	-0.480837761	0.000327832	-0.159501611	0.347525228
903 W903_RS04450	W903_0916	829256	830338	NA	DEAD/DEAH box helicase family protein	-0.103857016	0.559276733	-0.632135298	5.86111E-10	-0.634897827	0.000000894
904 W903_RS04455	W903_0917	830483	831112	udk	uridine kinase	-1.636782399	8.5766E-26	0.148009732	0.137643547	-0.280592628	0.087785952
905 W903_RS04460	W903_0918	831200	831697	NA	GAF domain protein	-0.299762495	0.079081585	0.179501265	0.141328327	0.389946762	0.008999529
906 W903_RS04465	W903_0919	831697	833361	dnaX	DNA polymerase III, subunit gamma and tau	-0.417022037	0.007096258	0.149145634	0.110455436	0.020456905	0.905991133
907 W903_RS04470	W903_0920	833450	833644	NA	hypothetical protein	1.655190916	7.02613E-05	0.987798056	0.013552105	-0.099732185	0.819894382
908 W903_RS04475	W903_0921	833625	834560	NA	biotin--[acetyl-CoA-carboxylase] ligase	0.176816451	0.377928802	0.129187624	0.296728612	-0.258484215	0.123916224
909 W903_RS04480	W903_0922	834745	835941	metK	methionine adenosyltransferase	0.07262008	0.687671982	-1.009813404	1.45567E-29	1.243633307	1.87E-13
910 W903_RS04490	W903_0923	836462	838453	NA	coagulase family protein	-1.27773695	1.15101E-09	-1.69534454	1.13382E-21	-0.409414367	0.246459592
911 W903_RS04495	W903_0924	838520	839065	NA	hypothetical protein	-1.296128561	7.22377E-07	-1.572137612	1.09427E-09	-0.377254286	0.510360749
912 W903_RS04500	W903_0925	839461	840027	NA	thiamine-transporter family protein	-0.187253712	0.747730994	2.54022457	9.09392E-15	-1.00504205	0.001191221
913 W903_RS04505	W903_0926	840024	840578	NA	putative HMP/thiamine permease protein ykoE	0.214380894	0.731761702	1.47222816	4.83686E-08	-1.206133687	0.0000423
914 W903_RS04510	W903_0927	840582	841868	NA	heme ABC exporter, ATP-binding protein CcmA	0.551387343	0.191586598	2.152761673	3.36552E-18	-0.891832288	0.0000988
915 W903_RS04515	W903_0928	841856	842524	NA	putative membrane protein	0.216671925	0.768072746	1.915527421	2.76566E-09	-1.347788547	0.0000165
916 W903_RS04520	W903_0929	842608	843288	tenA	thiaminase II	0.660164437	0.165913277	1.281699335	3.67004E-06	-1.033398949	0.00023759
917 W903_RS04525	W903_0930	843290	844087	thiD	hydroxymethylpyrimidine/phosphomethylpyrimidine kinase	0.865869684	0.013905768	1.562102293	8.88578E-13	-0.984297583	0.0000204
918 W903_RS04530	W903_0931	844089	844859	thiM	hydroxyethylthiazole kinase	0.649564754	0.054921009	1.355437684	9.60419E-11	-1.253550262	1.45E-09
919 W903_RS04535	W903_0932	844873	845544	thiE	thiamine-phosphate pyrophosphorylase	0.111559806	0.827375315	1.702970419	1.49322E-12	-0.956892571	0.000237764
920 W903_RS04540	W903_0933	845671	846930	NA	UDP-N-acetylglucosamine 1-carboxyvinyltransferase	0.126808875	0.502942487	0.048554572	0.726361185	-0.271394326	0.08915084
921 W903_RS04545	W903_0934	847032	847586	NA	acetyltransferase family protein	-0.006844134	0.973222982	1.089903919	2.2781E-21	-0.075660977	0.718594428
922 W903_RS04550	W903_0935	847579	848862	NA	CBS domain protein	0.1979352	0.252500145	0.422247474	1.36959E-05	-0.023089139	0.900482041
923 W903_RS04555	W903_0936	848885	849745	map	methionine aminopeptidase, type I	0.700862764	1.463E-05	0.163100331	0.129617586	0.10139023	0.503115078
924 W903_RS04560	W903_0937	849747	850667	NA	virulence factor BrkB family protein	0.635292113	4.78083E-05	0.335821379	0.001432285	0.159343109	0.264579289
925 W903_RS04565	W903_0938	850684	851139	NA	gtrA-like family protein	-0.969942647	0.005076862	0.412538158	0.204414793	-0.847757518	0.003990971
926 W903_RS04570	W903_0939	851321	851830	queT	queuosine transporter QueT	-0.820233541	4.1732E-08	-1.555717816	3.48248E-33	-0.517798347	0.013320471
927 W903_RS04575	W903_0940	851982	853928	ligA	DNA ligase, NAD-dependent	0.499522963	0.001105734	0.094260677	0.341223627	-0.28074088	0.023115201
928 W903_RS04580	W903_0941	853940	854959	NA	lipid kinase, YegS/Rv2252/BmrU family protein	0.538295361	0.000460978	0.039197986	0.733689214	-0.271860751	0.033977143
929 W903_RS04585	W903_0942	854963	857263	pulA	pullulanase, type I	0.180721998	0.275766915	-0.199103221	0.03565578	-0.058458458	0.690797222
930 W903_RS04590	W903_0943	857469	859337	glgB	1,4-alpha-glucan branching enzyme	1.147536936	7.20686E-10	0.689094887	3.16448E-12	0.869438834	1E-13
931 W903_RS04595	W903_0944	859379	860518	glgC	glucose-1-phosphate adenylyltransferase	1.738736481	7.30603E-21	0.480783946	5.57154E-05	1.078385559	4.1E-20
932 W903_RS04600	W903_0945	860508	861641	glgD	glucose-1-phosphate adenylyltransferase, GlgD subunit	1.625405778	1.32511E-17	0.581260459	4.38414E-05	0.852787102	1.83E-12
933 W903_RS04605	W903_0946	861638	863068	glgA	glycogen/starch synthase, ADP-glucose type family protein	1.254972527	8.69757E-15	0.425758519	0.002453192	1.143227678	5.66E-22
934 W903_RS04610	W903_0947	863400	863600	NA	ATP synthase subunit C family protein	0.113499867	0.565005127	-0.648632548	5.17678E-08	-0.185482674	0.275519269
935 W903_RS04615	W903_0948	863632	864348	atpB	ATP synthase F0, A subunit	0.096971111	0.566833212	-0.704060931	1.66235E-12	-0.09911596	0.537635804
936 W903_RS04620	W903_0949	864366	864863	atpF	ATP synthase F0, B subunit	0.594541876	6.49823E-05	-0.516535419	5.11704E-06	-0.24936739	0.073786513
937 W903_RS04625	W903_0950	864863	865399	atpH	ATP synthase F1, delta subunit	0.706212105	2.61462E-06	-0.879868289	1.12479E-17	-0.439591979	0.000651342

938	W903_RS04630	W903_0951	865415	866920	atpA	ATP synthase F1, alpha subunit	0.849687546	6.5073E-09	-0.62307236	1.37651E-10	-0.177574429	0.260887517
939	W903_RS04635	W903_0952	866936	867817	atpG	ATP synthase F1, gamma subunit	0.692146799	3.55732E-06	-0.693101048	2.01354E-11	-0.097398484	0.553178386
940	W903_RS04640	W903_0953	867891	869297	atpD	ATP synthase F1, beta subunit	0.783040975	3.23016E-08	-0.738559477	3.22541E-15	-0.365329298	0.01192786
941	W903_RS04645	W903_0954	869310	869723	atpC	ATP synthase F1, epsilon subunit	1.329937158	1.08436E-16	-0.395196372	0.001681175	-0.475580778	0.000460326
942	W903_RS04650	W903_0955	869788	870018	NA	hypothetical protein	0.734576826	0.001272911	-1.03416977	1.80514E-07	0.669663876	0.134592934
943	W903_RS04655	W903_0956	870081	871352	NA	UDP-N-acetylglucosamine 1-carboxyvinyltransferase	0.749580013	8.9586E-07	0.4368301	1.70934E-05	0.16630393	0.256512364
944	W903_RS04660	W903_0957	871354	871545	NA	DNA-directed RNA polymerase subunit beta family protein	0.825946141	0.001376657	0.193972897	0.363249424	0.521153367	0.103284154
945	W903_RS04665	W903_0958	871620	872477	NA	DNA/RNA non-specific endonuclease family protein	-0.188041779	0.28022116	-0.193781955	0.087342605	0.654073175	0.000117514
946	W903_RS04670	W903_0959	872768	873808	pheS	phenylalanine--tRNA ligase, alpha subunit	0.227233409	0.167861172	-1.570721215	8.34367E-27	0.592734524	0.000847837
947	W903_RS04675	W903_0960	873891	874412	NA	acetyltransferase family protein	0.125444731	0.490201152	-1.789312478	5.1102E-45	0.702489454	0.002549382
948	W903_RS04680	W903_0961	874466	876871	pheT	phenylalanine--tRNA ligase, beta subunit	0.240197075	0.137041773	-1.85193665	2.7745E-44	1.370813448	3.38E-20
949	W903_RS04685	W903_0962	876939	877841	NA	neutral zinc metallopeptidase family protein	0.317367129	0.110401251	2.05864763	1.84115E-93	-0.45358949	0.001300951
950	W903_RS04690	W903_0963	878019	881252	rexB	ATP-dependent nuclease subunit B	0.391482996	0.017653818	0.502130919	2.10902E-07	0.273253389	0.042053577
951	W903_RS04695	W903_0964	881242	884865	addA	helicase-exonuclease AddAB, AddA subunit	0.211443288	0.208680463	0.017994262	0.8647731	0.744806607	4.77E-08
952	W903_RS04700	W903_0965	884878	885795	NA	corA-like Mg2+ transporter family protein	-0.189294265	0.334106354	-0.451918576	5.52356E-05	0.764826877	0.0000441
953	W903_RS04705	W903_0966	885827	887203	trmE	tRNA modification GTPase TrmE	-0.195553936	0.290890617	0.000537306	0.997018054	0.142670059	0.393053232
954	W903_RS04710	W903_0967	887508	887858	NA	hypothetical protein	-1.490340285	2.55238E-11	-4.128840511	8.0402E-199	-1.636503602	0.00000117
955	W903_RS04715	W903_0968	888203	890113	NA	ABC transporter family protein	-0.15239356	0.38790006	-0.591778495	6.46995E-08	0.519140093	0.001955464
956	W903_RS04720	W903_0969	890262	891230	NA	thiamine pyrophosphate enzyme, C-terminal TPP binding domain protein	0.248347419	0.192577816	1.396252508	5.47321E-47	0.91125326	2.56E-14
957	W903_RS04725	W903_0970	891305	892303	NA	transketolase, pyrimidine binding domain protein	0.465439034	0.006084625	1.431849224	7.46068E-39	0.585498636	0.00000125
958	W903_RS04730	W903_0971	892430	893818	NA	e3 binding domain protein	0.445085532	0.005100077	1.266415484	1.8157E-40	0.484708266	0.0000588
959	W903_RS04735	W903_0972	893878	895635	lpdA	dihydrolipoyl dehydrogenase	0.668969552	5.5686E-06	1.029621219	1.62365E-21	0.094326713	0.489444277
960	W903_RS04740	W903_0973	895733	896722	NA	lipoyltransferase and lipoate-ligase family protein	0.168595424	0.426882086	-0.090503022	0.497184259	0.251715236	0.079824453
961	W903_RS04745	W903_0974	896830	897615	NA	cobB/CobQ-like glutamine amidotransferase domain protein	0.2068702	0.232196112	-0.177741973	0.081294498	-0.113315738	0.491748971
962	W903_RS04750	W903_0975	897615	898958	NA	mur ligase middle domain protein	0.327748104	0.043817578	0.265326678	0.004760232	-0.286271821	0.043485243
963	W903_RS04755	W903_0976	899098	899949	NA	disA bacterial checkpoint controller nucleotide-binding family protein	-0.137479698	0.514758507	0.589146484	3.73204E-09	-0.077794786	0.614802555
964	W903_RS04760	W903_0977	899952	900911	NA	hypothetical protein	-0.387866288	0.012850878	-0.18708536	0.045923079	0.128437266	0.389110381
965	W903_RS04765	W903_0978	900965	902317	glmM	phosphoglucosamine mutase	-0.453411839	0.001988807	-0.246906462	0.0053025	-0.045723108	0.77240887
966	W903_RS04770	W903_0979	902440	902811	NA	hypothetical protein	0.015317681	0.944361683	0.373736065	0.008723108	0.762331439	0.00000735
967	W903_RS04775	W903_0980	902836	903216	NA	pyrimidine dimer DNA glycosylase family protein	0.530030905	0.120099062	0.772471959	0.003091065	0.532042435	0.195189197
968	W903_RS04780	W903_0981	903310	904440	NA	radical SAM superfamily protein	-0.245245007	0.149383746	1.148482687	1.79779E-32	0.332320669	0.00978164
969	W903_RS04785	W903_0982	904444	905181	NA	acyl-ACP thioesterase family protein	-0.64196342	4.81156E-05	0.829806056	2.30494E-15	0.401419964	0.004729178
970	W903_RS04790	W903_0983	905182	905952	NA	hydrolase, haloacid dehalogenase-like family	-0.385232253	0.044189912	0.722493515	1.27746E-10	-0.030142349	0.862157506
971	W903_RS04795	W903_0984	905927	906598	NA	hypothetical protein	-0.708924041	0.000189977	0.598523583	8.89813E-07	-0.054555085	0.750481516
972	W903_RS04800	W903_0985	906987	911111	NA	HNH endonuclease family protein	-0.679028455	2.2204E-06	0.27234835	0.005217265	0.226568882	0.075559845
973	W903_RS04805	W903_0986	911113	911982	cas1	CRISPR-associated endonuclease Cas1	-0.665322705	0.000251548	0.285077782	0.015513928	0.169589285	0.251853039
974	W903_RS04810	W903_0987	911979	912320	cas2	CRISPR-associated endoribonuclease Cas2	-1.335496895	1.38538E-08	-0.04188144	0.829234282	0.334160525	0.132141007
975	W903_RS04815	W903_0988	912307	912972	NA	CRISPR-associated family protein	-0.927890899	5.35334E-09	0.48828333	1.80761E-05	0.330500258	0.014842412
976	NA	W903_0989	913084	913464	NA	hypothetical protein	-1.127185294	1.45102E-07	0.63823914	1.24958E-07	0.110840015	0.4499439
977	NA	W903_0990	913480	913593	NA	glycerophosphoryl diester phosphodiesterase family protein	-0.360436845	0.426630721	0.757413363	0.000246443	0.153813527	0.42539727
978	NA	W903_0991	914013	914150	NA	hypothetical protein	-0.715979221	NA	1.341763201	0.007392586	-0.636350106	0.271292986
979	NA	W903_0992	914244	914381	NA	putative membrane protein	-0.299057454	0.701826668	1.446888733	0.000214025	-0.250972128	0.62530696
980	W903_RS04825	W903_0993	914522	914938	awd	nucleoside diphosphate kinase	0.743428884	2.02559E-05	-0.097028393	0.358860404	1.302155518	6.67E-17
981	W903_RS04830	W903_0994	915074	916906	lepA	GTP-binding protein LepA	-0.326564932	0.042811516	0.016074657	0.879954279	-0.188542416	0.219907298
982	NA	W903_0995	916957	917100	NA	hypothetical protein	-0.394517434	NA	4.191601493	0.127064085	1.029887815	0.789199622
983	W903_RS04835	W903_0996	917152	919785	NA	histidine triad domain protein	-0.827218848	8.1812E-06	-1.36470238	1.22179E-24	0.027347148	0.930859283
984	W903_RS04840	W903_0997	919891	920544	NA	HD domain protein	-0.290680628	0.159911711	0.757717577	3.091E-14	0.740514095	0.0000214
985	W903_RS04845	W903_0998	920553	921017	NA	acetyltransferase family protein	-0.349486284	0.077791473	0.45472412	1.18142E-05	0.79434996	0.00000366
986	W903_RS04850	W903_0999	921017	921451	msrB	peptide methionine sulfoxide reductase MsrB	0.374668368	0.117276802	0.590541669	0.000151643	0.478244483	0.028693196
987	W903_RS04855	W903_1000	921603	924395	NA	HAD ATPase, P-type, IC family protein	0.234978826	0.183944453	1.130264495	1.24617E-33	0.169954573	0.202542728
988	W903_RS04860	W903_1001	924395	925498	NA	hypothetical protein	0.019183562	0.929646957	1.069954729	1.55446E-22	0.333647443	0.010625781
989	W903_RS04865	W903_1002	925617	926186	NA	bacterial transferase hexapeptide family protein	-0.087880786	NA	3.842935328	2.308E-09	0.778859409	0.301091774
990	W903_RS04870	W903_1003	926872	927483	NA	hypothetical protein	-0.525775316	0.002136569	0.614254055	9.43318E-07	-0.262962148	0.153013236
991	W903_RS04875	W903_1004	927520	928302	NA	putative membrane protein	0.72198109	7.37723E-07	-0.99318671	5.1281E-27	0.474772928	0.004469671
992	W903_RS04880	W903_1005	928314	929012	NA	ABC transporter family protein	-0.035082814	0.842938478	-1.610800849	2.09409E-63	0.150466664	0.354332934
993	W903_RS04885	W903_1006	929013	929381	NA	bacterial regulatory s, gntR family protein	-0.211226338	0.192257021	-1.394950694	1.37574E-41	0.574012771	0.000196508
994	W903_RS04890	W903_1007	929526	932630	dnaE	DNA polymerase III, alpha subunit	-0.036996004	0.842938478	-0.002845509	0.978444394	0.553516633	0.000106787
995	W903_RS04895	W903_1008	932711	933733	pfkA	6-phosphofructokinase	0.394831271	0.009634177	-1.144514647	2.07751E-30	0.818374877	2.62E-09
996	W903_RS04900	W903_1009	933782	935284	pyk	pyruvate kinase	0.131263983	0.423805945	-1.353117956	1.9412E-44	0.913660331	8.57E-10
997	W903_RS04905	W903_1010	935455	936012	NA	signal peptidase I	-0.876288753	7.48811E-07	-0.020089791	0.879954279	0.85267087	8.27E-10
998	NA	W903_1011	936030	936161	NA	hypothetical protein	-1.499887449	0.011610497	-0.018514995	0.971479693	1.586140156	0.168850428
999	W903_RS04910	W903_1012	936270	938084	glmS	glutamine-fructose-6-phosphate transaminase	-2.117060745	2.33753E-19	-1.257799978	1.04005E-15	1.287458649	1.85E-19
1000	W903_RS04915	W903_1013	938247	938576	NA	phnA family protein	-0.922438391	7.53337E-05	-0.200771601	0.52506759	0.495032219	0.517209215
1001	W903_RS04920	W903_1014	938711	939352	NA	amino ABC transporter, permease , 3-TM region, His/Glu/Gln/Arg/opine family domain protein	-1.040921967	2.14802E-09	-0.446121438	2.48943E-05	-0.59048486	0.00892297
1002	W903_RS04925	W903_1015	939362	939991	NA	ABC transporter family protein	-0.912858801	5.27257E-07	-0.561417008	5.65561E-08	-0.603962256	0.006932039
1003	W903_RS04930	W903_1016	940053	940838	NA	polar amino acid ABC uptake transporter substrate binding protein	-1.206954827	1.15038E-12	-0.802246588	6.77636E-12	-0.784173694	0.000556266
1004	W903_RS04935	W903_1017	940923	941156	rpsT	ribosomal protein S20	-1.105516521	1.95216E-09	-0.580864885	0.005341415	0.406517488	0.433134212
1005	W903_RS04940	W903_1018	941225	942145	coaA	pantothenate kinase	-0.60535395	0.000884833	-0.413705912	0.003076482	0.723659096	0.000959302
1006	W903_RS04945	W903_1019	942251	942841	NA	mycolic acid cyclopropane synthetase family protein	-0.885244065	4.80153E-06	-0.105947149	0.521261113	0.076108736	0.718594428
1007	W903_RS04950	W903_1020	943167	943556	cdd	cytidine deaminase	-0.037159333	0.858438886	-0.757667579	1.94808E-07	0.04184961	0.824418193
1008	W903_RS04955	W903_1021	943621	944670	NA	basic membrane family protein	-0.01553147	0.935760556	-1.4448642	1.60762E-24	0.132604368	0.354847058
1009	W903_RS04960	W903_1022	944815	946350	NA	heme ABC exporter, ATP-binding protein CcmA	0.19054838	0.299971351	-1.361568311	1.30159E-36	0.021962163	0.904836992
1010	W903_RS04965	W903_1023	946343	947404	NA	branched-chain amino acid transport system / permease component family protein	0.122605214	0.529108033	-1.383217537	2.34324E-33	0.162816835	0.226095447
1011	W903_RS04970	W903_1024	947406	948362	NA	branched-chain amino acid transport system / permease component family protein	0.286336675	0.131385468	-1.38822	1.03265E-21	0.150641096	0.280881365
1012	W903_RS04975	W903_1025	948568	949938	NA	putative NADH oxidase	0.359578089	0.031813237	-0.800446947	5.11772E-12	0.623765115	0.0000114
1013	W903_RS04980	W903_1026	950059	951048	NA	L-lactate dehydrogenase	0.459327378	0.001743195	-0.275358255	0.001939386	0.575304754	0.000129325
1014	W903_RS04985	W903_1027	951287	953746	gyrA	DNA gyrase, A subunit	0.056016647	0.74558947	0.202381064	0.029736522	0.451640415	0.000403812
1015	W903_RS04990	W903_1028	953753	954496	NA	sortase family protein	-0.437252663	0.004077726	0.025149018	0.830532698	0.474264552	0.000232388
1016	W903_RS04995	W903_1029	954512	954925	NA	hypothetical protein	-0.572663473	0.000533008	-0.265478625	0.010396499	0.571576995	0.0000637
1017	W903_RS05000	W903_1030	955031	955993	NA	hypothetical protein	-0.213803056	0.242243803	-0.800979699	3.01264E-20	0.666950594	0.00000201
1018	W903_RS05005	W903_1031	956056	957183	NA	sodium/hydrogen exchanger family protein	0.107254602	0.603411444	1.130283088	2.40344E-14	2.300442819	1.53E-35
1019	W903_RS05010	W903_1032	957574	958164	NA	helix-turn-helix family protein	-0.743654325	7.96905E-05	-1.421425854	2.0911E-17	-0.05306322	0.852893118
1020	W903_RS05015	W903_1033	958413	958904	NA	hypothetical protein	0.149684612	NA	1.032758133	0.213587937	-0.070355244	0.934878411
1021	W903_RS05020	W903_1034	959221	959643	NA	glycerophosphoryl diester phosphodiesterase family protein	-0.449507019	NA	0.313947655	0.673406985	-0.092010544	0.905991133
1022	W903_RS05025	W903_1035	959636	960694	NA	replication initiation factor family protein	0.02558737	0.950325218	1.919446089	2.4225E-21	0.328817656	0.252499383
1023	W903_RS05035	W903_1036	960918	961136	NA	hypothetical protein	0.600027814	0.204883473	0.243000764	0.507515057	-0.559444403	0.427394265
1024	W903_RS05040	W903_1037	961129	962310	NA	phage integrase family protein	0.308672824	0.346377236	-0.032925265	0.885454913	-0.488106036	0.087368756
1025	NA	W903_1038	962437	962550	NA	putative membrane protein	-0.277468129	0.125079485	-0.571072423	6.41649E-08	1.335736727	9.8E-19
1026	W903_RS05045	W903_1039	962786	964348	guaA	GMP synthase domain protein	0.465461831	0.001566678	-0.588688537	2.38376E-09	-0.23957446	0.086991754
1027	W903_RS05050	W903_1040	964561	965259	NA	bacterial regulatory s, gntR family protein	0.175666594	0.334549017	-0.458367517	2.42966E-05	0.448591687	0.012794856
1028	W903_RS05055	W903_1041	965373	966707	trmFO	m(5)U-54 methyltransferase	0.044728945	0.798496828	-0.048921778	0.622519677	0.712930418	4.29E-08
1029	W903_RS05060	W903_1042	966794	967537	NA	acetyltransferase family protein	-0.13057707	0.566833212	0.139623828	0.266694644	0.448367184	0.028725045
1030	W903_RS05065	W903_1043	967670	968518	NA	NLPA lipofamily protein	-0.23197186	0.295683724	0.238692558	0.041743461	0.25554633	0.159879898
1031	W903_RS05070	W903_1044	968550	969038	NA	hypothetical protein	0.88477219	2.55269E-06	0.598801215	1.6234E-10	1.265124008	2.2E-16
1032	W903_RS05075	W903_1045	969204	970166	NA	peptidase S41 family protein	0.698236375	0.013978764	1.721640789	7.17993E-25	0.045604495	0.848581618
1033	W903_RS05080	W903_1046	970192	970944	NA	ABC transporter family protein	0.21510272	0.330951294	0.236678106	0.074331436	0.463546191	0.00715078
1034	W903_RS05085	W903_1047	970946	972901	NA	ftsX-like permease family protein	-0.160311424	0.437073844	0.24514496	0.030623076	0.482736466	0.000784106
1035	W903_RS05090	W903_1048	973011	973679	NA	response regulator	0.203244592	0.330951294	0.52850549	4.65191E-05	0.377949662	0.027686432
1036	W903_RS05095	W903_1049	973703	974614	NA	histidine kinase-, DNA gyrase B-, and HSP90-like ATPase family protein	0.109266094	0.56709391	0.079332922	0.487119712	0.590984551	0.000115304
1037	W903_RS05100	W903_1050	974652	975722	NA	phage integrase family protein	0.05348529	0.800421156	0.673451243	1.19823E-05	0.396277975	0.008022297
1038	W903_RS05105	W903_1051	976181	977842	NA	bacterial extracellular solute-binding s, 5 Middle family protein	-0.131777185	0.576503921	-1.213494805	2.48408E-08	0.639853095	0.02324368
1039	W903_RS05110	W903_1052	977875	978648	NA	hypothetical protein	0.244176225	0.355685142	-0.439413437	0.027758752	0.265896306	0.222823609
1040	W903_RS05115	W903_1053	978645	979325	NA	putative dNA-binding protein	-0.16283813	0.549536178	-0.444370331	0.064405998	0.227103982	0.381320189
1041	W903_RS05120	W903_1054	979392	980957	ffh	signal recognition particle protein	-0.087806129	0.62592635	-0.547678564	8.4829E-09	0.175374224	0.217512204
1042	W903_RS05125	W903_1055	980975	981307	NA	sigma-70, region 4 family protein	-0.497038222	0.004636477	0.151544867	0.241301424	0.343978697	0.076802695
1043	W903_RS05130	W903_1056	981396	982709	NA	his Kinase A domain protein	0.133534307	0.495003156	-0.236881108	0.025859052	0.241834731	0.067613003
1044	W903_RS05135	W903_1057	982693	983373	NA	response regulator	0.046018626	0.834002409	-0.182602882	0.134991815	0.753333946	0.0000443
1045	W903_RS05140	W903_1058	983535	986084	NA	peptidase M1 family protein	0.394244282	0.014605413	-0.005649973	0.960968817	0.111540281	0.41812632
1046	W903_RS05145	W903_1059	986230	986883	NA	phosphate transport system regulatory protein PhoU	-0.035622952	0.851542185	-1.097246084	2.19634E-23	0.321922177	0.025716029
1047	W903_RS05150	W903_1060	986917	987675	NA	phosphate ABC transporter, ATP-binding protein	0.05462653	0.778085155	-0.787718413	1.121E-13	0.284288703	0.039745065
1048	W903_RS05155	W903_1061	987687	988490	NA	phosphate ABC transporter, ATP-binding protein	0.257207452	0.145574329	-0.516320058	3.85918E-07	0.361962233	0.015518884
1049	W903_RS05160	W903_1062	988502	989389	NA	phosphate ABC transporter, permease protein PstA	0.522045471	0.000639817	-0.251763097	0.041256441	0.056163795	0.719313212
1050	W903_RS05165	W903_1063	989379	990296	NA	phosphate ABC transporter, permease protein PstC	0.608769445	0.000154533	-0.237010445	0.046369028	-0.143142291	0.354562741
1051	W903_RS05170	W903_1064	990342	991202	pstS	phosphate binding family protein	0.281682283	0.092175742	-0.743157971	2.48408E-08	-0.090525453	0.602101327
1052	W903_RS05175	W903_1065	991387	992697	NA	ftsJ-like methyltransferase family protein	0.163588202	0.390412221	-0.047537206	0.709747935	-0.042173506	0.817003097
1053	W903_RS05180	W903_1066	992765	993529	NA	inositol monophosphatase family protein	-0.083323932	0.669580883	0.268329235	0.021806423	-0.396617959	0.001925909
1054	W903_RS05185	W903_1067	993519	993800	NA	hypothetical protein	0.016802247	0.938553751	0.135859	0.403553308	-0.308858596	0.145578588
1055	W903_RS05190	W903_1068	993793	994206	spxA	regulatory protein spx	-0.554021654	0.000452079	0.020634245	0.853816698	0.362446633	0.00641007
1056	W903_RS05195	W903_1069	994249	995181	ribF	riboflavin biosynthesis protein RibF	0.491763362	0.003331312	1.130789841	7.76635E-30	0.671713838	0.00000988
1057	W903_RS05200	W903_1070	995194	996078	truB	tRNA pseudouridine(55) synthase	-0.033011312	0.861869569	0.628038424	1.1865E-07	0.776395909	0.0000011
1058	W903_RS05205	W903_1071	996170	996601	NA	acetyltransferase family protein	-0.290254613	0.17335524	0.320186287	0.012890278	-0.560912806	0.000144043
1059	W903_RS05210	W903_1072	996614	997885	NA	hypothetical protein	-0.425374872	0.006895447	0.244982897	0.022675249	-0.325060632	0.015755494
1060	W903_RS05215	W903_1073	997919	998509	NA	type I restriction modification DNA specificity domain protein	-0.373322776	0.032840104	0.547784026	2.6099E-08	-0.117627204	0.429886933
1061	W903_RS05220	W903_1074	998616	999494	NA	CAAX protease self-immunity family protein	-0.835354967	7.66601E-07	1.422368186	2.53374E-21	1.436555398	7.71E-24
1062	W903_RS05225	W903_1075	999600	1002230	NA	ftsX-like permease family protein	0.038770624	0.851821574	3.624720564	3.09334E-87	0.788749293	4.56E-08
1063	W903_RS05230	W903_1076	1002242	1002943	NA	ABC transporter family protein	-0.024755527	0.92288861	2.895117123	5.35815E-54	0.55720417	0.000161273
1064	W903_RS05235	W903_1077	1003080	1005200	topA	DNA topoisomerase I	0.118578784	0.521627441	0.698862247	2.98749E-14	0.025683595	0.917248446
1065	W903_RS05240	W903_1078	1005295	1006137	dprA	DNA protecting protein DprA	-0.050319992	0.927127413	0.741231601	0.024319473	-1.513017474	0.018558027
1066	W903_RS05245	W903_1079	1006276	1007304	NA	hypothetical protein	-0.512384535	0.001986404	0.116491089	0.294963144	-1.495154291	4.52E-19
1067	W903_RS05250	W903_1080	1007366	1008127	NA	ABC transporter family protein	-0.354709529	0.051345143	0.472744412	1.26168E-06	-1.448795021	7.14E-19
1068	W903_RS05255	W903_1081	1008124	1009098	NA	fecCD transport family protein	-0.293154303	0.095916955	0.513511973	3.10491E-08	-1.646962382	3.24E-23
1069	W903_RS05260	W903_1082	1009095	1010057	NA	fecCD transport family protein	0.103943913	0.566833212	0.760960972	1.86107E-10	-1.886312679	5.15E-33
1070	W903_RS05265	W903_1083	1010297	1010845	NA	bacterial transferase hexapeptide family protein	-0.347465529	0.057826884	-0.349344681	0.002709689	-0.399543522	0.006541848
1071	W903_RS05270	W903_1084	1010866	1011627	NA	ribonuclease HII family protein	-0.077299993	0.730573433	-0.109696718	0.4214648	-0.309141035	0.087826581

1072	W903_RS05275	W903_1085	1011614	1012465	ylqF	ribosome biogenesis GTP-binding protein YlqF	0.074936839	0.690197928	-0.088890143	0.527329565	0.073739422	0.674976013
1073	W903_RS05290	W903_1089	1014521	1014742	NA	helix-turn-helix family protein	0.329823509	0.216420552	-0.483948614	0.003697406	0.138148595	0.703114345
1074	W903_RS05295	W903_1090	1014848	1015444	NA	bacterial regulatory s, luxR family protein	-1.259681053	3.45274E-14	-1.811246594	5.63076E-42	0.131916649	0.537655259
1075	W903_RS05300	W903_1091	1015425	1016999	NA	putative membrane protein	-1.373358235	5.70773E-16	-1.307870428	1.49247E-33	0.029438937	0.906214492
1076	W903_RS05305	W903_1092	1017176	1019194	NA	ftsX-like permease family protein	-0.94614881	9.0664E-05	1.126136888	8.0194E-18	-0.556089709	0.002595772
1077	W903_RS05310	W903_1093	1019206	1019940	NA	ABC transporter family protein	-0.996542015	0.013431719	0.81326389	0.000994203	-0.263064982	0.360420549
1078	W903_RS05315	W903_1094	1020496	1020672	NA	hypothetical protein	-1.123540428	2.95302E-13	0.713385014	1.35147E-13	0.620433949	0.0000145
1079	W903_RS05320	W903_1095	1020760	1020942	NA	hypothetical protein	-0.539872638	0.126627854	-0.037295534	0.889974398	0.124282585	0.637118222
1080	W903_RS05325	W903_1096	1021026	1021208	NA	hypothetical protein	-1.183168427	0.000480529	-0.417738301	0.038603155	0.314836601	0.246459592
1081	W903_RS05330	W903_1097	1022147	1024270	NA	ABC transporter family protein	-0.536941065	0.017858701	1.455318183	3.5163E-15	0.188924034	0.312003355
1082	W903_RS05335	W903_1098	1024283	1027234	lanM	type 2 lantibiotic biosynthesis LanM family protein	0.019228186	0.927127413	2.520135474	9.7698E-160	-0.221034257	0.142045893
1083	W903_RS05340	W903_1099	1027412	1027894	NA	lantibiotic streptin immunity family protein	-0.608195888	0.083786987	2.430676904	8.30669E-47	-0.483126445	0.027328437
1084	W903_RS05345	W903_1100	1027966	1028607	NA	relaxase/mobilization nuclease domain protein	0.003681667	0.994427047	2.123147543	1.17366E-28	-0.348603265	0.10148024
1085	W903_RS05350	W903_1101	1028975	1029547	NA	L,D-transpeptidase catalytic domain protein	-0.84974796	1.67301E-07	0.183810998	0.267299509	0.704518218	0.000000329
1086	W903_RS05355	W903_1102	1029748	1031232	NA	carbon starvation CstA family protein	2.707598809	4.95695E-55	-0.04341803	0.764577305	-0.684088432	0.0000249
1087	W903_RS05360	W903_1103	1031388	1032122	NA	response regulator	0.172190109	0.47748382	1.590185046	4.18683E-39	-0.401170668	0.009420368
1088	W903_RS05365	W903_1104	1032134	1033873	NA	histidine kinase-, DNA gyrase B-, and HSP90-like ATPase family protein	-0.142592219	0.496067777	1.331373512	1.01551E-37	-0.332961423	0.020531944
1089	NA	W903_1105	1034411	1034533	NA	putative lipoprotein	0.075430553	0.798433935	1.10012684	9.21107E-15	0.122753725	0.64606451
1090	NA	W903_1106	1034760	1034882	NA	hypothetical protein	-1.006396532	0.005759166	0.612537894	0.019444779	0.321428359	0.429886933
1091	W903_RS05375	W903_1107	1034895	1035092	NA	N-acetylmuramoyl-L-alanine amidase domain protein	0.397312623	0.246905013	1.170772539	2.66387E-08	-0.192342538	0.52792383
1092	W903_RS05380	W903_1108	1035609	1036025	NA	hypothetical protein	-0.467800692	0.083234472	0.250996769	0.176041325	0.678799986	0.002289085
1093	W903_RS05385	W903_1109	1036196	1036441	NA	hypothetical protein	-0.428866621	0.366086675	0.1286682	0.689485296	0.213841911	0.632747072
1094	W903_RS05390	W903_1110	1036495	1037145	NA	glycerophosphoryl diester phosphodiesterase family protein	0.508004462	0.12356904	0.731959295	0.002698068	0.575338245	0.043756958
1095	W903_RS05395	W903_1111	1037158	1037934	NA	hypothetical protein	0.633337996	0.000587297	0.578185948	1.44392E-05	0.130419748	0.440830357
1096	W903_RS05400	W903_1112	1038229	1038579	NA	hypothetical protein	0.339247553	0.125003968	0.153445255	0.315205531	0.479554517	0.078940691
1097	W903_RS05405	W903_1113	1038734	1039039	NA	hypothetical protein	0.071064649	0.733854288	-0.220246804	0.087125947	0.719386973	0.00187083
1098	W903_RS05410	W903_1114	1039041	1039694	NA	hypothetical protein	0.305822406	0.096549476	0.000423475	0.997018054	0.953469464	7.91E-08
1099	W903_RS05425	W903_1117	1040973	1041278	NA	hypothetical protein	0.675373737	0.000221839	0.216358045	0.132408127	1.261170637	7.09E-09
1100	W903_RS05435	W903_1118	1041470	1041970	NA	glycerophosphoryl diester phosphodiesterase family protein	0.272508901	0.214111123	-0.163938988	0.321132808	1.721593548	3.96E-13
1101	W903_RS05440	W903_1119	1041983	1043419	NA	LXG domain of WXG superfamily protein	1.222771331	1.72555E-16	0.508549227	7.27455E-06	1.012305122	2.36E-15
1102	W903_RS05445	W903_1120	1043400	1043801	NA	glycerophosphoryl diester phosphodiesterase family protein	1.065459578	9.24753E-07	0.472664432	0.01549857	1.165921428	0.000000333
1103	W903_RS05450	W903_1121	1043807	1044187	NA	type VII secretion effector, TIGR04197 family protein	1.29441355	2.1653E-10	0.697657825	2.39622E-05	0.611466948	0.002854789
1104	W903_RS05455	W903_1122	1044201	1048610	essC	type VII secretion protein EssC	1.650125219	1.62442E-25	0.916936862	3.72887E-15	0.535764361	0.0000209
1105	W903_RS05460	W903_1123	1048617	1049894	essB	type VII secretion protein EssB	1.551773359	5.69163E-14	1.077341693	2.27303E-12	0.194332516	0.230227539
1106	W903_RS05465	W903_1124	1049894	1050106	NA	yukD family protein	1.707846687	2.92519E-08	1.42227992	1.55977E-12	0.237449149	0.424971378
1107	W903_RS05470	W903_1125	1050120	1050548	essA	type VII secretion protein EssA	0.758825789	0.003754263	0.576742385	0.000662629	0.44512403	0.0210782
1108	W903_RS05475	W903_1126	1050602	1053613	esaA	type VII secretion protein EsaA	1.513785472	3.57802E-10	1.055397543	3.70801E-19	0.018328755	0.923848652
1109	NA	W903_1127	1053764	1053985	NA	virulence factor esxA	0.958831873	0.001370887	2.300780357	1.29522E-46	0.010298564	0.970287626
1110	W903_RS05485	W903_1128	1054152	1054442	NA	virulence factor esxA	0.33311804	0.171350406	-0.862474361	5.3001E-10	2.725841074	2.09E-58
1111	W903_RS05490	W903_1129	1054559	1055341	NA	lipase family protein	1.627813081	0.367966213	-0.272089017	0.079675359	4.339746606	1.12E-152
1112	W903_RS05495	W903_1130	1055338	1055661	NA	hypothetical protein	1.349117117	0.508587412	-0.567816165	0.000110681	4.733559817	1.85E-107
1113	W903_RS05500	W903_1131	1055787	1058969	carB	carbamoyl-phosphate synthase, large subunit	2.423695939	0.302225232	-0.49571727	0.000509529	4.90019079	4.51E-210
1114	W903_RS05505	W903_1132	1059000	1060076	NA	carbamoyl-phosphate synthase, small subunit	3.241283673	0.18396017	-0.658533218	6.9593E-06	5.149937284	3.62E-281
1115	W903_RS05510	W903_1133	1060087	1061013	pyrB	aspartate carbamoyltransferase	3.330470062	0.185033565	-0.81648181	1.08118E-09	4.885361458	2.68E-258
1116	W903_RS05515	W903_1134	1061182	1062474	pyrC	dihydroorotase, multifunctional complex type domain protein	2.926347114	0.171028626	-0.531821574	3.51623E-07	4.460290941	6.26E-213
1117	W903_RS05520	W903_1135	1062486	1063115	pyrE	orotate phosphoribosyltransferase	2.968996739	0.170250592	-0.897893956	1.40427E-21	4.129889703	1.79E-167
1118	W903_RS05525	W903_1136	1063128	1063829	pyrF	orotidine 5'-phosphate decarboxylase	3.273827889	0.140850224	-0.933074958	9.43323E-15	3.629275579	1.43E-122
1119	W903_RS05530	W903_1137	1064042	1065274	NA	hypothetical protein	0.650334646	2.41743E-05	1.157503483	7.29086E-34	-0.514335675	0.00023953
1120	W903_RS05535	W903_1138	1065314	1066855	NA	heme ABC exporter, ATP-binding protein CcmA	0.594362592	0.00021008	1.008559552	5.31242E-26	-0.199774783	0.161438417
1121	W903_RS05540	W903_1139	1066986	1067324	NA	ATP cone domain protein	-0.219607479	0.301270944	0.690430484	3.07363E-10	0.461684527	0.002862191
1122	W903_RS05545	W903_1140	1067494	1068570	asd	aspartate-semialdehyde dehydrogenase	0.150487429	0.423756017	-0.647972123	2.96697E-09	0.022480807	0.904836992
1123	W903_RS05550	W903_1141	1068779	1069867	NA	LPXTG cell wall anchor domain protein	1.145750511	2.32386E-14	-0.903220082	1.61028E-16	-1.586491143	0.00014422
1124	W903_RS05555	W903_1142	1070557	1072152	cls	cardiolipin synthase	0.127070903	0.495327691	0.05865886	0.568288627	-0.292989734	0.032006706
1125	W903_RS05560	W903_1143	1072271	1073941	NA	formate--tetrahydrofolate ligase family protein	0.844441711	1.463E-05	-0.284625866	0.009051042	0.079473756	0.614802555
1126	W903_RS05565	W903_1144	1074030	1075049	NA	lipoyltransferase and lipoate-ligase family protein	0.430575315	0.018037897	1.147878918	5.25972E-34	-0.084837769	0.594495109
1127	W903_RS05570	W903_1145	1075076	1075954	NA	hypothetical protein	0.696186533	0.000522225	1.400309026	4.12082E-36	-0.559157467	0.000106685
1128	W903_RS05575	W903_1146	1075923	1076741	NA	macro domain protein	0.374497796	0.070238826	1.044396607	1.45015E-24	-0.490571681	0.000143477
1129	W903_RS05580	W903_1147	1076734	1077066	NA	glycine cleavage H-family protein	-0.166035262	0.484301603	0.674557387	0.000128659	-0.500493492	0.005172184
1130	W903_RS05585	W903_1148	1077095	1078081	NA	luciferase oxidoreductase, group 1 family protein	-0.149717797	0.430969743	1.031021223	1.2815E-27	-0.51266046	0.0000563
1131	W903_RS05590	W903_1149	1078078	1079277	NA	flavin oxidoreductase / NADH oxidase family protein	0.309929852	0.070856166	1.391738507	1.15079E-30	-0.438175462	0.001058535
1132	W903_RS05595	W903_1150	1079270	1080106	NA	biotin/lipoate A/B ligase family protein	0.125281788	0.549536178	1.148741662	4.91333E-20	-0.483508085	0.001007897
1133	W903_RS05600	W903_1151	1080260	1080946	coaB	phosphopantothenate--cysteine ligase	0.270932893	0.252500145	0.198772191	0.203463071	-1.52262962	4.61E-14
1134	W903_RS05605	W903_1152	1080939	1081481	coaC	phosphopantothenoylcysteine decarboxylase	0.918076042	6.49053E-06	0.379602767	0.012429057	-1.445843576	1.88E-21
1135	W903_RS05610	W903_1153	1081527	1082099	panT	pantothenic acid transporter PanT	-0.139438521	0.547728992	0.268508956	0.064799571	-1.401597102	5.37E-16
1136	W903_RS05615	W903_1154	1082209	1083927	NA	phosphoglucomutase/phosphomannomutase, C-terminal domain protein	0.239411047	0.133952618	-0.258334275	0.020187344	0.654757555	0.00000117
1137	W903_RS05625	W903_1155	1084201	1085934	NA	ABC transporter family protein	-0.213973402	0.259242344	0.496848661	2.57132E-05	-0.548160837	0.0000319
1138	W903_RS05630	W903_1156	1085935	1087656	NA	ABC transporter family protein	0.185023834	0.355552361	0.503704357	1.62826E-06	-0.592172368	0.0000187
1139	W903_RS05635	W903_1157	1087668	1088270	NA	lysozyme-like family protein	0.241550808	0.144486237	0.424820018	5.12463E-05	-0.345301204	0.010183546
1140	W903_RS05640	W903_1158	1088272	1089246	NA	37-kD nucleoid-associated bacterial family protein	0.170454638	0.330951294	0.133263844	0.263576189	-0.200251477	0.142699054
1141	W903_RS05645	W903_1159	1089254	1090510	NA	serine hydroxymethyltransferase family protein	0.559992261	0.000142889	0.27810538	0.004686857	-0.240706458	0.063451632
1142	W903_RS05650	W903_1160	1090602	1091198	NA	tRNA threonylcarbamoyl adenosine modification protein, Sua5/YciO/YrdC/YwlC family	0.625360642	7.96905E-05	0.564086128	7.9152E-08	-0.58154345	0.000130118
1143	W903_RS05655	W903_1161	1091191	1092021	prmC	protein-(glutamine-N5) methyltransferase, release factor-specific	0.398330508	0.013077952	0.458487662	3.91675E-05	-0.32938136	0.031937696
1144	W903_RS05660	W903_1162	1092021	1093100	prfA	peptide chain release factor 1	0.287284446	0.103410495	0.357905848	0.001202895	-0.486409846	0.00110111
1145	W903_RS05665	W903_1163	1093135	1093704	NA	thymidine kinase family protein	-0.004491633	0.98220294	0.690716371	3.28328E-10	-0.347496855	0.068681578
1146	W903_RS05670	W903_1164	1093842	1094024	NA	tautomerase enzyme family protein	0.616317107	0.17915471	-1.217106807	0.000235652	-0.758571555	0.400596927
1147	W903_RS05675	W903_1165	1094200	1095111	NA	apbE family protein	-0.111380911	0.648243664	-1.322793257	1.31129E-48	-1.072933593	8.98E-16
1148	W903_RS05680	W903_1166	1095129	1095731	NA	NADPH-dependent FMN reductase family protein	0.582951876	0.00609631	-1.195898511	7.74918E-34	-0.819937613	7.3E-11
1149	W903_RS05685	W903_1167	1095755	1096990	NA	NADPH-dependent FMN reductase family protein	0.487477817	0.011787837	-0.911037261	7.04866E-20	-0.808011736	7.04E-10
1150	W903_RS05690	W903_1168	1097089	1097877	NA	formate/nitrite transporter family protein	-0.497316611	0.002888992	-0.69288253	3.87193E-12	-1.043346992	1.19E-18
1151	W903_RS05695	W903_1169	1097976	1099250	pbuX	xanthine permease family protein	-0.525898388	0.503169838	-1.167897525	8.68515E-18	-0.310415937	0.073603915
1152	W903_RS05700	W903_1170	1099250	1099831	xpt	xanthine phosphoribosyltransferase	-0.91877624	0.126321509	-1.351270535	1.19076E-17	0.144967813	0.492501788
1153	W903_RS05705	W903_1171	1100086	1101069	guaC	guanosine monophosphate reductase	0.258014347	0.801246181	-1.38532491	5.82959E-30	3.45310447	2.87E-78
1154	W903_RS05710	W903_1172	1101272	1102612	NA	major Facilitator Superfamily protein	-1.605247484	6.36731E-17	0.035851383	0.828867428	0.17338801	0.550748674
1155	W903_RS05715	W903_1173	1102904	1103575	NA	hypothetical protein	-0.564104835	0.012778518	2.529667467	2.4636E-126	0.285991376	0.171266648
1156	W903_RS05720	W903_1174	1103647	1105647	NA	potassium uptake protein	-0.236874465	0.165421465	0.36216689	0.001689706	-1.233897312	4.12E-19
1157	W903_RS05725	W903_1175	1105782	1106543	NA	short chain dehydrogenase family protein	-0.330988688	0.119042498	1.328049876	3.6958E-19	0.12798586	0.501190182
1158	W903_RS05730	W903_1176	1106605	1107597	pta	phosphate acetyltransferase	-0.428051591	0.009686322	0.687897419	1.39181E-14	0.486905097	0.00076298
1159	W903_RS05735	W903_1177	1107623	1108507	NA	pseudouridine synthase, RluA family protein	0.427402882	0.011872739	1.398731405	2.27186E-35	-0.157990862	0.352137223
1160	W903_RS05740	W903_1178	1108504	1109340	NA	ATP-NAD kinase family protein	0.174910545	0.337197526	1.141608995	1.97082E-17	-0.202461012	0.25268107
1161	W903_RS05745	W903_1179	1109315	1109986	NA	RelA / SpoT s family protein	-0.075755542	0.701826668	1.301743108	6.63042E-29	-0.218743762	0.160759087
1162	W903_RS05750	W903_1180	1110096	1110668	NA	CYTH domain protein	-0.652537108	0.001794779	-0.888093863	1.41542E-15	0.688220052	0.000630647
1163	W903_RS05755	W903_1181	1110845	1111819	prs2	ribose-phosphate pyrophosphokinase 2	0.429331647	0.004709769	0.055689572	0.605838703	-0.324914076	0.012015664
1164	W903_RS05760	W903_1182	1111823	1112938	NA	aminotransferase class-V family protein	0.337548613	0.045426067	0.270928538	0.003671115	-0.183713534	0.151916333
1165	W903_RS05765	W903_1183	1112940	1113287	NA	amino acid metabolism family protein	0.144460416	0.421324797	-0.034561809	0.77640213	-0.017682517	0.930859283
1166	W903_RS05770	W903_1184	1113442	1114080	NA	coA binding domain protein	-0.54220383	0.00473903	0.780417862	3.90463E-14	1.942272914	8.42E-48
1167	W903_RS05775	W903_1185	1114093	1114773	NA	DNA repair RadC family protein	-0.1454613	0.705759832	0.959755583	2.9645E-07	1.190476944	4.77E-08
1168	W903_RS05780	W903_1186	1114869	1116002	NA	hypothetical protein	-0.402381061	0.012299144	-0.12658035	0.246865747	-0.146336314	0.322798369
1169	W903_RS05785	W903_1187	1116093	1117532	NA	glycosyl hydrolase 1 family protein	0.361735279	0.067561666	1.456424106	1.84123E-43	0.554108829	0.000444459
1170	W903_RS05790	W903_1188	1117692	1118306	NA	GDSL-like Lipase/Acylhydrolase family protein	-0.252185205	0.322923737	1.23876817	1.38931E-22	0.783293503	0.000211504
1171	W903_RS05795	W903_1189	1118407	1119228	NA	HAD hydrolase, IIB family protein	-0.15706692	0.446374739	0.628907367	9.63239E-09	0.715877279	0.000103404
1172	W903_RS05800	W903_1190	1119853	1120782	NA	cupin domain protein	-0.430325599	0.064894799	1.208060492	4.62239E-19	0.107203492	0.625089267
1173	W903_RS05805	W903_1191	1120839	1122371	NA	voltage gated chloride channel family protein	0.440239374	0.007362892	-0.21031068	0.049176934	-0.074935677	0.659915696
1174	W903_RS05810	W903_1192	1122480	1123553	NA	bacterial extracellular solute-binding family protein	0.296117743	0.087957605	-0.125902827	0.271291798	-0.322237099	0.014447091
1175	W903_RS05815	W903_1193	1123546	1124322	NA	binding--dependent transport system inner membrane component family protein	-0.043865788	0.83442661	-0.167537451	0.212139333	-0.069882164	0.713104755
1176	W903_RS05820	W903_1194	1124319	1125113	NA	binding--dependent transport system inner membrane component family protein	0.355579134	0.042927774	-0.026565417	0.840175823	-0.388987506	0.01273087
1177	W903_RS05825	W903_1195	1125097	1126251	NA	polyamine ABC transporter, ATP-binding family protein	0.246198791	0.144808373	-0.304811282	0.00100782	-0.276330541	0.046641415
1178	W903_RS05830	W903_1196	1126300	1127202	murB	UDP-N-acetylenolpyruvoylglucosamine reductase	-0.043559086	0.820911404	-0.618220932	5.14632E-09	-0.280011344	0.06047458
1179	W903_RS05835	W903_1197	1127346	1127834	folK	2-amino-4-hydroxy-6-hydroxymethyldihydropteridine diphosphokinase	0.942179839	3.08386E-07	0.789384685	1.1223E-05	0.103978068	0.650219593
1180	W903_RS05840	W903_1198	1127831	1128193	folB	dihydroneopterin aldolase	0.468780425	0.003312238	0.514965258	3.60574E-07	0.399926306	0.007905887
1181	W903_RS05845	W903_1199	1128195	1128998	folP	dihydropteroate synthase	0.612429765	7.72947E-05	0.580318447	5.71887E-08	0.539354799	0.0000473
1182	W903_RS05850	W903_1200	1129002	1129565	folE	GTP cyclohydrolase I	0.666816908	2.3133E-05	0.320061633	0.012172152	0.374457208	0.009255188
1183	W903_RS05855	W903_1201	1129584	1130846	folC	bifunctional FolC family protein	0.618889598	4.64408E-05	0.369783781	0.000122622	0.447156993	0.001289603
1184	W903_RS05860	W903_1202	1130848	1131735	NA	eamA-like transporter family protein	0.799495799	1.80896E-06	0.522289464	5.53317E-06	0.567695396	0.000276035
1185	W903_RS05865	W903_1203	1131722	1132588	thrB	homoserine kinase	1.052446577	7.13862E-11	0.617470643	3.26812E-07	0.749761545	0.00000339
1186	W903_RS05870	W903_1204	1132590	1133873	NA	homoserine dehydrogenase family protein	0.616431214	0.000575006	0.211564123	0.029145032	0.732188552	6.47E-08
1187	W903_RS05875	W903_1205	1134050	1134937	NA	polysaccharide deacetylase family protein	0.950537346	2.44262E-09	-0.463548235	0.000127039	-0.150319784	0.441254078
1188	W903_RS05880	W903_1206	1134960	1136507	NA	transporter, betaine/carnitine/choline transporter family protein	0.972959617	1.55178E-10	1.287231249	3.49508E-40	0.552940385	0.0000312
1189	W903_RS05885	W903_1207	1136650	1138026	ssdh	NAD-dependent aldehyde dehydrogenase	0.344435673	0.062591184	1.65375241	2.4803E-46	1.367418946	1.16E-32
1190	W903_RS05890	W903_1208	1138100	1139107	NA	conserved hypothetical 698 family protein	0.701790639	5.03372E-06	0.001963468	0.98621151	0.351400696	0.009856131
1191	W903_RS05895	W903_1209	1139215	1139901	NA	hypothetical protein	-0.160451877	0.576355478	0.287730718	0.016836589	1.213871539	1.07E-09
1192	W903_RS05900	W903_1210	1140380	1141660	NA	putative streptokinase-like protein	0.90550979	1.17861E-08	1.217714795	2.26244E-15	2.286237827	2.63E-82
1193	W903_RS05910	W903_1211	1142154	1142687	NA	hypothetical protein	0.086327497	0.78773361	2.3099964	2.31596E-50	0.689539597	0.000122609
1194	W903_RS05915	W903_1212	1142763	1143257	NA	putative thiol peroxidase	-0.812735287	0.000370662	1.831980755	1.34119E-66	0.584258066	0.0000237
1195	W903_RS05920	W903_1213	1143330	1143989	NA	DNA alkylation repair enzyme family protein	-0.693155265	7.02613E-05	0.637553272	2.12756E-09	0.463152737	0.00038808
1196	W903_RS05925	W903_1214	1143989	1144753	NA	HAD hydrolase, IIB family protein	-0.47220092	0.003389623	1.112707791	4.42738E-24	1.052606613	4.23E-19
1197	W903_RS05930	W903_1215	1144758	1145399	NA	deoR-like helix-turn-helix domain protein	-0.661303643	2.27955E-05	1.113454187	6.35989E-32	0.729849015	9.74E-10
1198	W903_RS05935	W903_1216	1145563	1146114	NA	asp23 family protein	-0.659066947	2.54872E-05	1.99526403	2.27956E-69	0.109193897	0.492501788
1199	W903_RS05940	W903_1217	1146136	1146333	NA	csbD-like family protein	-0.397407744	0.032607546	2.125890036	5.145E-58	0.224621453	0.105036054
1200	W903_RS05945	W903_1218	1146380	1146922	NA	asp23 family protein	-0.516934381	0.001989825	2.025334204	1.93516E-92	0.301806275	0.054046365
1201	W903_RS05950	W903_1219	1146978	1147172	NA	hypothetical protein	-0.345507573	0.069792582	2.03096036	3.72483E-92	0.165480288	0.309206592
1202	W903_RS05955	W903_1220	1147182	1147763	NA	hypothetical protein	-0.26571018	0.15126138	1.968075446	2.38217E-61	0.17749667	0.178175996
1203	W903_RS05960	W903_1221	1147778	1148026	NA	transglycosylase associated family protein	0.105280137	0.606893258	2.129049005	4.55176E-75	0.803851466	1.89E-10
1204	W903_RS05965	W903_1222	1148438	1150717	pcrA	ATP-dependent DNA helicase PcrA	0.13338851	0.503712789	-0.486116286	2.20027E-06	-0.051372522	0.763222804
1205	W903_RS05970	W903_1223	1150823	1151209	NA	hypothetical protein	0.314464252	0.855132285	-1.359597336	3.41174E-22	0.142809779	0.613217398

1206	W903_RS05975	W903_1224	1151342	1152667	NA	uracil-xanthine permease family protein	0.263974689	0.892719202	-2.425016088	8.49354E-91	0.974978207	6.7E-10
1207	W903_RS05980	W903_1225	1153264	1154610	agcS	amino acid carrier family protein	-0.377288236	0.034076374	-1.340368812	2.56998E-36	-1.157097816	5.44E-17
1208	W903_RS05985	W903_1226	1154674	1155909	NA	cation diffusion facilitator transporter family protein	-0.166794557	0.377928802	-0.265264007	0.034954341	-0.91669579	1.06E-13
1209	W903_RS05990	W903_1227	1156068	1156460	NA	lrgA family protein	-0.045159707	0.849655992	0.292383519	0.016014306	-0.436333394	0.002725634
1210	W903_RS05995	W903_1228	1156441	1157136	NA	lrgB-like family protein	-0.308798074	0.093988955	-0.242360806	0.056328032	-0.536522798	0.0000342
1211	W903_RS06000	W903_1229	1157205	1157828	NA	hypothetical protein	0.419703502	0.010676812	0.034734518	0.786620424	-0.291378006	0.034407545
1212	W903_RS06005	W903_1230	1158097	1158168	rRNA	NA	0.436011686	0.57647511	0.141413279	0.744297935	-0.146708778	0.819785648
1213	W903_RS06010	W903_1231	1158241	1159443	NA	S1 RNA binding domain protein	-0.637177534	5.25301E-06	-1.7814513	7.62959E-53	0.753766961	7.99E-08
1214	W903_RS06015	W903_1232	1159566	1159640	rRNA	NA	-0.038063934	NA	2.749820137	0.005102876	2.521830861	0.054468621
1215	W903_RS06020	W903_1233	1159646	1159726	rRNA	NA	0.285116626	NA	0.298445605	0.816430743	2.148633708	0.362694468
1216	W903_RS06025	W903_1234	1159787	1160017	NA	hypothetical protein	-0.960295824	0.000992352	1.767058055	1.30186E-14	1.578364734	0.001830863
1217	W903_RS06030	W903_1235	1160101	1161123	ilvE	branched-chain amino acid aminotransferase	-0.103235648	0.612207657	-1.020942623	1.23107E-24	0.087778612	0.570252167
1218	W903_RS06035	W903_1236	1161237	1163696	parC	DNA topoisomerase IV, A subunit	0.041222279	0.817491868	0.172917936	0.074709372	-0.057956812	0.695969553
1219	W903_RS06040	W903_1237	1163830	1165779	parE	DNA topoisomerase IV, B subunit	0.176187341	0.306496536	0.125394577	0.358647227	-0.485993895	0.001348063
1220	W903_RS06045	W903_1238	1165908	1166531	plsY	acyl-phosphate glycerol 3-phosphate acyltransferase	-0.780577206	8.00499E-07	-0.308781143	0.039225265	-0.701541783	0.00000304
1221	W903_RS06050	W903_1239	1166597	1167250	ung	uracil-DNA glycosylase	0.68094111	0.000233553	1.215807235	2.14412E-21	-0.457382381	0.006874489
1222	W903_RS06055	W903_1240	1167349	1167834	NA	hypothetical protein	-0.062071294	0.76251576	0.676496582	3.53153E-11	-0.002391821	0.989781024
1223	W903_RS06060	W903_1241	1167948	1169189	NA	GDSL-like Lipase/Acylhydrolase family protein	-0.412813556	0.010498911	-2.423410121	3.35475E-67	0.368477238	0.052075098
1224	W903_RS06065	W903_1242	1169200	1169829	NA	sugar O-acyltransferase, sialic acid O-acetyltransferase NeuD family protein	-0.339664177	0.045859063	-1.818653716	2.71737E-38	0.146129991	0.484391391
1225	W903_RS06070	W903_1243	1169826	1170980	neuC	UDP-N-acetyl-D-glucosamine 2-epimerase, UDP-hydrolysing	0.131262536	0.483957086	-1.519232702	6.42632E-28	-0.012194296	0.955648631
1226	W903_RS06075	W903_1244	1171057	1172082	neuB	N-acetylneuraminate synthase	0.222264648	0.219518125	-1.200500069	4.43591E-16	-0.055655148	0.765405013
1227	W903_RS06080	W903_1245	1172082	1173482	NA	polysaccharide biosynthesis family protein	-0.242510614	0.200727304	-0.940259488	8.6663E-15	-0.213330981	0.299180096
1228	W903_RS06085	W903_1246	1173479	1174435	NA	glycosyltransferase 52 family protein	-0.012583334	0.94796136	-0.807641134	1.90287E-13	-0.040073003	0.856773766
1229	W903_RS06090	W903_1247	1174432	1175397	NA	glycosyl transferase 2 family protein	-0.408612253	0.019814003	-0.882085157	6.30826E-15	-0.035072761	0.907463508
1230	W903_RS06095	W903_1248	1175390	1176373	NA	glycosyl transferase 2 family protein	-0.045032633	0.816605926	-0.710346408	7.83276E-12	0.170442353	0.398921296
1231	W903_RS06100	W903_1249	1176377	1177264	NA	glycosyl transferase 2 family protein	-0.883121765	2.20188E-07	-1.203866886	5.59528E-32	0.435180851	0.045586912
1232	W903_RS06105	W903_1250	1177290	1178015	NA	tcdA/TcdB catalytic glycosyltransferase domain protein	-0.514335692	0.001518599	-0.752536872	3.65107E-12	0.750195519	0.00000794
1233	W903_RS06110	W903_1251	1178054	1179148	NA	putative membrane protein	-1.03021102	1.52546E-08	-0.868209449	4.21881E-09	0.425109029	0.157120573
1234	W903_RS06115	W903_1252	1179135	1179626	NA	glycosyltransferase family 28 C-terminal domain protein	-0.545837285	0.002556422	-0.783399408	9.32472E-10	0.262139125	0.379321135
1235	W903_RS06120	W903_1253	1179626	1180075	NA	oligosaccharide biosynthesis Alg14 like family protein	-0.988405645	6.80475E-08	-0.966728685	1.99526E-15	0.597335557	0.042970258
1236	W903_RS06125	W903_1254	1180099	1181448	NA	exopolysaccharide biosynthesis polyprenyl glycosylphosphotransferase family protein	-0.736463214	3.41419E-06	-0.619695835	4.59342E-11	0.661275423	0.0000599
1237	W903_RS06130	W903_1255	1181500	1182198	cpsD	tyrosine-protein kinase CpsD	-0.799099788	2.33193E-07	-0.501045827	7.70307E-08	1.116332629	2.35E-12
1238	W903_RS06135	W903_1256	1182209	1182901	NA	chain length determinant family protein	-0.983432878	7.80592E-12	-0.832232818	1.01166E-18	0.844955039	6.79E-08
1239	W903_RS06140	W903_1257	1182910	1183641	cpsB	tyrosine-protein phosphatase CpsB	-0.881125201	1.04858E-07	-0.383651223	0.010569112	0.783123251	0.0000615
1240	W903_RS06145	W903_1258	1183647	1184990	NA	cell envelope-related function transcriptional attenuator common domain protein	-0.688567059	2.82859E-06	0.233807532	0.013589572	0.97714469	9.33E-11
1241	W903_RS06150	W903_1259	1185293	1186216	NA	bacterial regulatory helix-turn-helix , lysR family protein	-0.817446687	1.35979E-06	-0.235388983	0.041620173	-0.473699078	0.004253831
1242	W903_RS06155	W903_1260	1186241	1187008	NA	CRISPR-associated family protein	-0.014869539	0.935760556	-0.121538364	0.281119065	-0.203301183	0.112636231
1243	W903_RS06160	W903_1261	1187017	1187727	deoD	purine nucleoside phosphorylase	0.44233566	0.006967	0.099957538	0.388409219	-0.366051906	0.004511438
1244	W903_RS06165	W903_1262	1187711	1188883	NA	voltage gated chloride channel family protein	1.1157725	1.48836E-14	0.959724014	2.94891E-25	-0.261050001	0.057627416
1245	W903_RS06170	W903_1263	1188969	1189778	NA	purine nucleoside phosphorylase I, inosine and guanosine-specific	1.319582178	2.12645E-20	0.709501786	4.3196E-12	-0.110552229	0.483527276
1246	W903_RS06175	W903_1264	1189817	1190224	arsC	arsenate reductase	0.78640106	3.69583E-07	0.496833407	4.61343E-06	-0.04959679	0.761947617
1247	W903_RS06180	W903_1265	1190275	1191486	NA	phosphopentomutase	1.046136699	6.41305E-13	0.774658234	5.28046E-18	-0.161769421	0.245560557
1248	W903_RS06185	W903_1266	1191543	1192214	rpiA	ribose 5-phosphate isomerase A	0.730565372	1.25088E-06	1.028777953	9.37056E-21	-0.350771928	0.013384682
1249	W903_RS06190	W903_1267	1192452	1193144	NA	peptidase M54 family protein	-0.030601951	0.874114533	0.142224479	0.132778843	0.6067019	0.00000551
1250	W903_RS06195	W903_1268	1193252	1194040	NA	esterase family protein	0.237961195	0.14722531	0.361615016	0.001786465	0.961791059	7.8E-12
1251	W903_RS06200	W903_1269	1194097	1195758	NA	metallo-beta-lactamase superfamily protein	0.129167674	0.446374739	0.361893839	0.006250497	0.244301709	0.06594996
1252	W903_RS06205	W903_1270	1196054	1196812	NA	ABC transporter family protein	-0.163198849	0.483314144	0.266884185	0.042722465	0.454163255	0.014148783
1253	W903_RS06210	W903_1271	1196815	1197678	NA	branched-chain amino acid transport system / permease component family protein	-0.054433612	0.823792061	0.435841824	0.000293885	0.821848189	0.00000309
1254	W903_RS06215	W903_1272	1197691	1198695	NA	hypothetical protein	-0.282510574	0.170805376	-0.133662015	0.246936264	2.082001292	1.22E-40
1255	W903_RS06220	W903_1273	1199054	1200709	NA	fibronectin-binding A family protein	1.062881388	1.80086E-09	-0.182390586	0.179148679	-0.411361731	0.008959062
1256	W903_RS06225	W903_1274	1200763	1201482	budA	alpha-acetolactate decarboxylase	0.627754496	0.001080054	0.873599719	4.92057E-22	-0.112811576	0.427394265
1257	W903_RS06230	W903_1275	1201496	1203178	alsS	acetolactate synthase, catabolic	0.191794177	0.36122005	1.065991421	1.24994E-36	0.065415913	0.655835799
1258	W903_RS06235	W903_1276	1203288	1204514	NA	TPR repeat family protein	0.829610696	5.93578E-09	-0.243697025	0.008243248	0.293657297	0.037600888
1259	W903_RS06240	W903_1277	1204504	1205694	NA	hypothetical protein	0.775212866	6.4908E-08	-0.237509944	0.008945849	0.096385965	0.526336216
1260	W903_RS06245	W903_1278	1205786	1206247	NA	NUDIX domain protein	1.059250345	1.2197E-08	0.451716124	0.001516639	0.148031017	0.401864338
1261	W903_RS06250	W903_1279	1206237	1206719	mutX	mutator mutT protein	0.914259811	1.67172E-06	0.317639926	0.026068244	0.279583077	0.154012458
1262	W903_RS06260	W903_1282	1210208	1211254	rfbB	dTDP-glucose 4,6-dehydratase	0.707387072	8.20836E-07	-0.219618241	0.03300524	0.040615017	0.810530928
1263	W903_RS06265	W903_1283	1211461	1212054	NA	putative dTDP-4-dehydrorhamnose 3,5-epimerase	0.665520691	1.13108E-05	-0.468147519	1.81042E-06	0.097067244	0.551386059
1264	W903_RS06270	W903_1284	1212054	1212923	rfbA	glucose-1-phosphate thymidylyltransferase	0.73068025	5.55494E-07	-0.414202125	0.000636484	0.155264304	0.2906234
1265	W903_RS06275	W903_1285	1212982	1214085	NA	pyridine nucleotide-disulfide oxidoreductase family protein	0.687975377	2.68445E-05	0.015148529	0.900332109	-0.409156239	0.002000429
1266	W903_RS06280	W903_1286	1214094	1214882	NA	hypothetical protein	0.304078638	0.068552366	-0.42634577	3.45276E-05	-0.343051546	0.011752656
1267	W903_RS06285	W903_1287	1214872	1215555	NA	hypothetical protein	-0.176894511	0.367966213	-0.464266995	0.000117539	-0.361842469	0.027508943
1268	W903_RS06290	W903_1288	1215659	1216339	NA	hypothetical protein	-0.305717431	0.082114182	0.146274415	0.404141085	1.223486521	5.93E-11
1269	W903_RS06295	W903_1289	1216457	1216975	apt	adenine phosphoribosyltransferase	-0.884541823	8.18391E-07	-0.214420962	0.046764803	1.148698052	7.73E-13
1270	W903_RS06300	W903_1290	1217098	1219662	NA	kxYKxGKxW signal peptide domain protein	0.468013821	0.007850889	-1.302257542	1.61566E-46	-0.031782065	0.847599862
1271	W903_RS06305	W903_1291	1219814	1222012	recJ	single-stranded-DNA-specific exonuclease RecJ	0.008608861	0.958972063	0.004391513	0.973133722	-0.086139337	0.552317029
1272	W903_RS06310	W903_1292	1222009	1222770	NA	short chain dehydrogenase family protein	-0.00569148	0.977276951	0.108544845	0.361422285	-0.033023251	0.858303202
1273	W903_RS06315	W903_1293	1222772	1223701	rnz	ribonuclease Z	0.272906195	0.090008853	0.253915637	0.007874373	0.128809291	0.387464831
1274	W903_RS06320	W903_1294	1223743	1224390	NA	putative tRNA delta(2)-isopentenylpyrophosphate transferase	0.026938684	0.885855408	-0.137910466	0.240816638	0.207585646	0.219788403
1275	W903_RS06325	W903_1295	1224383	1225621	hflX	GTP-binding protein HflX	-0.105243094	0.549948356	-0.03857239	0.720005868	0.398742628	0.004823295
1276	W903_RS06330	W903_1296	1225712	1226602	miaA	tRNA dimethylallyltransferase	-0.884786133	1.72768E-07	-0.80305294	1.28616E-10	0.411115713	0.010240474
1277	W903_RS06335	W903_1297	1226713	1226889	NA	hypothetical protein	0.497129996	0.040946436	-0.288325125	0.038074983	0.721602973	0.000392816
1278	W903_RS06340	W903_1298	1226931	1227848	NA	C4-dicarboxylate transporter/malic acid transport family protein	-0.083699749	0.666552071	0.914056899	4.82761E-12	0.62559914	0.0000215
1279	W903_RS06345	W903_1299	1227983	1231741	NA	pullulanase, extracellular	0.000298067	0.999136702	1.466147175	1.22525E-09	-0.168904372	0.41470361
1280	W903_RS06355	W903_1302	1232658	1233242	NA	hypothetical protein	1.104859171	1.28257E-11	0.533576269	3.3551E-07	-0.453686562	0.000646535
1281	W903_RS06360	W903_1303	1233339	1234745	pepV	dipeptidase PepV	1.147838782	1.82309E-16	0.25474762	0.010374128	-0.10823002	0.423506874
1282	W903_RS06365	W903_1304	1234792	1235394	NA	putative NAD(P)H nitroreductase	0.448813982	0.00423563	-0.129828803	0.216159401	0.157843004	0.287866351
1283	W903_RS06375	W903_1307	1237387	1239168	uvrC	excinuclease ABC subunit C	0.249237072	0.143974617	0.681578818	2.1055E-12	-0.322214306	0.0210782
1284	W903_RS06380	W903_1308	1239327	1240094	NA	hypothetical protein	-0.445374222	0.016377276	-0.631055952	7.31889E-08	-0.498697196	0.009282018
1285	W903_RS06385	W903_1309	1240153	1241493	NA	MATE efflux family protein	0.332351958	0.05092725	0.911357584	8.22077E-21	0.031553196	0.853105716
1286	W903_RS06390	W903_1310	1241593	1242003	NA	3-demethylubiquinone-9 3-methyltransferase family protein	-0.066813326	0.733554053	0.696658616	3.23855E-11	0.500120033	0.000537688
1287	W903_RS06395	W903_1311	1242013	1242510	NA	hypothetical protein	-0.552976785	0.00040011	0.861183525	1.63237E-19	1.244479524	1.32E-16
1288	W903_RS06400	W903_1312	1242732	1243328	NA	host cell surface-exposed lipofamily protein	-0.623776182	0.001751002	-0.481820623	0.001560769	-1.301825743	5.44E-18
1289	NA	W903_1313	1243517	1243807	NA	transposase family protein	-0.098115519	0.828736007	1.389982092	8.67796E-11	-1.161433453	0.000000703
1290	W903_RS06410	W903_1314	1243987	1244622	NA	integrase core domain protein	-0.221859151	0.545409579	1.370941158	1.34053E-13	-0.883419005	0.00000783
1291	W903_RS06415	W903_1315	1244830	1245999	NA	hypothetical protein	-0.794913031	0.000222738	0.615909109	4.1605E-05	-0.123310818	0.593648001
1292	W903_RS06420	W903_1316	1246134	1246499	rplL	ribosomal protein L7/L12	-0.814184588	8.7789E-08	-2.697783052	7.9602E-114	-0.255369634	0.077620084
1293	W903_RS06425	W903_1317	1246563	1247063	NA	ribosomal L10 family protein	-1.262493267	4.70311E-19	-2.905014541	1.1519E-197	-0.282702204	0.06034205
1294	W903_RS06430	W903_1318	1247496	1249604	NA	putative ATP-dependent Clp protease ATP-binding subunit	-0.08991759	0.733854288	4.868058095	1.01264E-17	1.021309828	5.36E-12
1295	W903_RS06435	W903_1319	1249697	1250641	mmuM	homocysteine S-methyltransferase	0.939282781	0.000160331	0.365299822	0.10411911	-1.363863603	5.69E-17
1296	W903_RS06440	W903_1320	1250646	1252022	NA	amino acid permease family protein	1.340926526	2.30524E-09	0.668714182	0.000124543	-1.175554694	8.56E-10
1297	W903_RS06445	W903_1321	1252152	1252802	NA	hypothetical protein	1.068569436	0.021849764	0.694678934	0.013097361	-0.984073187	0.0000425
1298	NA	W903_1322	1252960	1253283	NA	hypothetical protein	0.674374824	0.251726351	0.488780419	0.088632077	-0.363651901	0.258953364
1299	W903_RS06455	W903_1323	1253555	1254103	NA	bacterial regulatory s, tetR family protein	0.444943371	0.129641826	1.098087639	9.384E-13	-1.01885577	0.0000109
1300	W903_RS06460	W903_1324	1254165	1254761	ysxC	ribosome biogenesis GTP-binding protein YsxC	0.317737574	0.098023272	0.736055939	1.44175E-10	-0.381010239	0.00477215
1301	W903_RS06465	W903_1325	1254772	1255998	clpX	ATP-dependent Clp protease, ATP-binding subunit ClpX	0.404603763	0.012061269	0.913589361	2.48631E-16	-0.43359269	0.000493918
1302	NA	W903_1326	1256028	1256198	NA	putative membrane protein	0.300086558	0.141342389	1.127863919	7.88471E-21	-0.252408222	0.133855499
1303	W903_RS06475	W903_1327	1256216	1256710	dhfR	dihydrofolate reductase	0.078244302	0.701826668	1.045913486	8.46275E-13	-0.407763681	0.005023624
1304	W903_RS06480	W903_1328	1256790	1257629	NA	thymidylate synthase family protein	0.244788563	0.156956193	1.047811315	5.00144E-21	-0.174114923	0.225831203
1305	W903_RS06485	W903_1329	1257783	1258955	NA	hydroxymethylglutaryl-CoA synthase	0.012839345	0.942141234	-0.216461793	0.058167976	0.270025048	0.064242791
1306	W903_RS06490	W903_1330	1258957	1260240	NA	hydroxymethylglutaryl-CoA reductase, degradative	-0.135720091	0.43289178	-0.17416308	0.166981255	-0.14240942	0.403380004
1307	W903_RS06495	W903_1331	1260332	1260781	NA	hypothetical protein	1.326113554	4.94156E-14	-1.007639004	1.3367E-14	1.39996703	7.06E-23
1308	W903_RS06500	W903_1332	1260774	1261430	NA	channel , hemolysin III family protein	2.35489015	4.47616E-31	-0.251797588	0.100943123	0.0212733	0.918801321
1309	W903_RS06505	W903_1333	1261517	1262431	NA	lipid kinase, YegS/Rv2252/BmrU family protein	1.923535338	4.52609E-26	-0.073782144	0.626202626	-0.514527138	0.000220392
1310	W903_RS06510	W903_1334	1262532	1263386	NA	glutathione S-transferase, C-terminal domain protein	-1.007463458	9.79895E-07	1.436449017	8.12215E-29	1.326473379	5.5E-20
1311	W903_RS06515	W903_1335	1263432	1263650	NA	hypothetical protein	0.695097777	0.075787491	0.631027735	0.043044182	0.000646427	0.998712491
1312	W903_RS06520	W903_1336	1263718	1264713	fni	isopentenyl-diphosphate delta-isomerase, type 2	0.243106555	0.133078652	0.325805303	0.007811223	-0.016186489	0.930859283
1313	W903_RS06525	W903_1337	1264710	1265702	NA	phosphomevalonate kinase	0.1108823	0.560572697	0.34613809	0.003465317	-0.130022005	0.450682737
1314	W903_RS06530	W903_1338	1265695	1266639	mvaD	diphosphomevalonate decarboxylase	0.228058283	0.192634382	0.457307743	2.16126E-05	-0.072077324	0.727854742
1315	W903_RS06535	W903_1339	1266621	1267499	mvk	mevalonate kinase	-0.121620569	0.525444689	0.68663371	1.92994E-06	-0.01002733	0.966937318
1316	W903_RS06540	W903_1340	1267615	1268844	NA	his Kinase A domain protein	-0.274286715	0.102080595	0.39782384	2.32854E-05	-0.50186828	0.000123663
1317	W903_RS06545	W903_1341	1268844	1269530	NA	putative transcriptional activator CadC	0.220094913	0.243397527	0.599164467	1.48202E-09	-0.460482708	0.000857285
1318	W903_RS06550	W903_1342	1269532	1270158	NA	RelA / SpoT s family protein	-0.007615803	0.966792967	0.458017716	3.59172E-06	-0.336736166	0.014148783
1319	W903_RS06555	W903_1343	1270307	1270513	NA	hypothetical protein	0.070046984	0.881155871	1.005955167	1.19022E-05	-0.361228378	0.154012458
1320	W903_RS06560	W903_1344	1271165	1273573	NA	LPXTG cell wall anchor domain protein	0.148861692	0.355685142	0.295323837	0.00202924	0.191779999	0.135113246
1321	W903_RS06565	W903_1345	1274332	1274772	NA	marR family protein	-0.939103981	2.25955E-07	1.534992088	1.95984E-43	0.831443708	2.93E-08
1322	W903_RS06570	W903_1346	1275048	1277120	NA	calcineurin-like phosphoesterase family protein	-0.579925293	0.001251359	-1.077817143	2.31256E-08	-0.527616554	0.00027441
1323	W903_RS06575	W903_1347	1277157	1277567	NA	polypeptide deformylase family protein	0.532350286	0.007514823	0.586773874	2.91923E-05	0.41201494	0.007496613
1324	W903_RS06580	W903_1348	1277637	1278986	NA	glutamate/Leucine/Phenylalanine/Valine dehydrogenase family protein	0.664612425	0.00041814	0.159291638	0.132778843	0.326372792	0.014842412
1325	W903_RS06585	W903_1349	1279154	1279663	NA	hypothetical protein	0.745122779	6.92818E-06	0.395586347	0.000154703	-0.046148175	0.787346245
1326	W903_RS06590	W903_1350	1279704	1281473	NA	ABC transporter family protein	0.208181092	0.270803414	0.524254028	6.38585E-07	0.120515539	0.427394265
1327	W903_RS06595	W903_1351	1281478	1283217	NA	ABC transporter family protein	0.304015198	0.092519667	0.481762227	2.79395E-06	-0.061001891	0.690797222
1328	W903_RS06605	W903_1354	1283754	1285622	NA	ABC transporter family protein	0.396521493	0.027448288	0.201311307	0.043937525	-0.349922755	0.006093578
1329	W903_RS06610	W903_1355	1285633	1286826	NA	poly A polymerase head domain protein	0.14455382	0.423756017	-0.031591939	0.805210061	-0.025235709	0.889083826
1330	W903_RS06615	W903_1356	1287026	1287874	NA	degV family protein	0.252083238	0.11442041	-0.096705524	0.354936197	0.18107454	0.194664147
1331	W903_RS06620	W903_1357	1287874	1288254	NA	hypothetical protein	-0.464013483	0.003622254	-0.594975114	5.34182E-10	0.483136279	0.001076653
1332	W903_RS06625	W903_1358	1288326	1288859	NA	hypothetical protein	0.415950972	0.03611521	0.351100069	0.008290542	0.098052012	0.654522375
1333	W903_RS06630	W903_1359	1288910	1289404	NA	hypothetical protein	-0.171826751	0.364890673	0.26303013	0.029727196	1.358145185	3.17E-15
1334	W903_RS06635	W903_1360	1289593	1291557	fruA	PTS system, fructose subfamily, IIA component domain protein	0.722412793	4.56038E-07	-0.058154159	0.575881752	-0.356981404	0.007955004
1335	W903_RS06640	W903_1361	1291554	1292465	pfkB	1-phosphofructokinase	-0.105356133	0.561356379	-0.637423848	3.61998E-12	-0.050956	0.721797567
1336	W903_RS06645	W903_1362	1292462	1293205	NA	deoR C terminal sensor domain protein	-0.30880776	0.045859063	-0.822034936	1.0368E-14	0.79704967	0.000000235
1337	W903_RS06650	W903_1363	1293336	1294571	NA	femAB family protein	-0.166889229	0.330951294	0.985739964	0.078839324	1.485043652	4.26E-25
1338	W903_RS06655	W903_1364	1294715	1296349	NA	gram-positive signal peptide, YSIRK family domain protein	-0.465186349	0.002147831	0.514410915	0.282483334	2.652412134	2.94E-87
1339	W903_RS06660	W903_1365	1296561	1297484	NA	2-dehydropantoate 2-reductase family protein	-0.047266514	0.828736007	-1.862496808	9.32617E-75	0.468319983	0.00184105

1340	W903_RS06665	W903_1366	1297497	1298567	NA	phosphotransferase system, EIIC family protein	0.335635238	0.087948687	-1.348876987	4.62285E-39	-0.064308488	0.702263859
1341	W903_RS06670	W903_1367	1298704	1299696	NA	pyridine nucleotide-disulfide oxidoreductase family protein	-0.351319009	0.098516323	-0.572307069	2.0121E-05	0.27698418	0.208511256
1342	W903_RS06675	W903_1368	1299677	1300429	trmD	tRNA (guanine(37)-N(1))-methyltransferase	-0.199812013	0.368429976	-0.462777011	0.000105813	-0.016788219	0.948562652
1343	W903_RS06680	W903_1369	1300419	1300937	rimM	16S rRNA processing protein RimM	-0.840052156	0.00040299	-0.500432977	0.003776266	0.874708192	0.004958473
1344	W903_RS06685	W903_1370	1301000	1301071	rRNA	NA	-1.361794617	NA	1.749024832	0.394304118	1.913103038	0.576427024
1345	W903_RS06690	W903_1371	1301080	1301151	rRNA	NA	-1.693603038	NA	1.414811274	0.464350799	4.579295302	0.094856967
1346	W903_RS06695	W903_1372	1301259	1302770	NA	M trans-acting positive regulator (MGA) PRD domain protein	-0.509898554	0.001559194	0.614415611	2.44493E-11	1.196765082	1.11E-14
1347	W903_RS06700	W903_1373	1302902	1303144	NA	KH domain protein	-1.795302431	3.83034E-16	-3.028437101	2.57868E-15	2.438591495	3.96E-32
1348	W903_RS06705	W903_1374	1303154	1303426	rpsP	ribosomal protein S16	-2.02658652	2.13764E-26	-3.291055952	8.00682E-13	2.267338545	3.91E-18
1349	W903_RS06710	W903_1375	1303555	1304802	NA	ftsX-like permease family protein	0.246565079	0.154337021	-2.538618827	2.3512E-154	0.244121289	0.182456141
1350	W903_RS06715	W903_1376	1304817	1305527	NA	ABC transporter family protein	0.59265889	0.000489113	-2.132492155	1.11724E-89	0.301049106	0.056418248
1351	W903_RS06720	W903_1377	1305530	1306774	NA	efflux transporter, RND family, MFP subunit	0.173158621	0.370894861	-2.403392787	5.5274E-111	0.711705161	0.0000273
1352	W903_RS06725	W903_1378	1306976	1308574	NA	carbamoyl-phosphate synthase L chain, ATP binding domain protein	-0.120773013	0.655104421	-0.220658026	0.059189362	0.494517391	0.000498201
1353	W903_RS06730	W903_1379	1308630	1309700	NA	carbamoyl-phosphate synthase, small subunit	0.180794479	0.535808713	-0.378271061	7.24933E-05	0.326645518	0.020897783
1354	W903_RS06735	W903_1380	1309749	1310270	pyrR	bifunctional protein pyrR	0.079428098	0.795809871	-0.511303123	6.40686E-08	0.350719678	0.016042162
1355	W903_RS06740	W903_1381	1310446	1311336	NA	pseudouridine synthase, RluA family protein	0.054909352	0.78773361	-0.254498121	0.016235582	-0.27951368	0.053074535
1356	W903_RS06745	W903_1382	1311320	1311784	lspA	signal peptidase II	-0.628270137	0.001863435	-0.153410799	0.293289048	0.174125238	0.427394265
1357	W903_RS06750	W903_1383	1311793	1312698	NA	bacterial regulatory helix-turn-helix , lysR family protein	-0.405665497	0.016838871	0.002313954	0.986368808	-0.189707611	0.313413822
1358	W903_RS06755	W903_1384	1312914	1313207	rpmA	ribosomal protein L27	-0.577157549	0.000252231	-0.880677398	2.2785E-07	-0.992983919	3.62E-10
1359	W903_RS06760	W903_1385	1313229	1313567	NA	hypothetical protein	-1.235234514	3.01443E-16	-1.081589142	5.37872E-25	-0.12597115	0.393859163
1360	W903_RS06765	W903_1386	1313574	1313888	rplU	ribosomal protein L21	-0.75634837	4.73097E-05	-0.496488432	0.000374705	-0.593832911	0.0000532
1361	W903_RS06770	W903_1387	1314078	1315256	NA	bacterial capsule synthesis PGA_cap family protein	-0.17030046	0.368868788	0.117404307	0.464958121	0.034785011	0.847939358
1362	W903_RS06775	W903_1388	1315358	1316572	thiI	tRNA sulfurtransferase ThiI	0.150625232	0.423657793	-0.210993406	0.033853887	0.139003219	0.484211778
1363	W903_RS06780	W903_1389	1316574	1317719	NA	aminotransferase class-V family protein	-0.232708256	0.176735425	0.014357076	0.899203501	0.496064952	0.000505478
1364	W903_RS06785	W903_1390	1317966	1318418	NA	hypothetical protein	-0.129900141	0.560572697	0.939992577	1.30111E-13	-1.048253401	1.44E-12
1365	W903_RS06790	W903_1391	1318461	1319810	gor	glutathione-disulfide reductase	0.628021329	0.00014327	1.074522511	1.27263E-28	0.610253523	0.00000388
1366	W903_RS06795	W903_1392	1319985	1320320	NA	hypothetical protein	-0.749726588	1.36475E-05	-0.261003704	0.013223196	0.366074021	0.011782922
1367	W903_RS06800	W903_1393	1320404	1321570	aroC	chorismate synthase	0.010186278	0.955813252	0.386138695	0.000668051	-0.140469748	0.395862986
1368	W903_RS06805	W903_1394	1321571	1322638	aroB	3-dehydroquinate synthase	0.38752971	0.030488964	0.935249611	1.51222E-12	-0.076933154	0.713615523
1369	W903_RS06810	W903_1395	1322732	1323409	aroD	3-dehydroquinate dehydratase	-0.900433087	3.80062E-10	-0.328489882	0.000392025	0.119928585	0.489038842
1370	W903_RS06815	W903_1396	1323409	1324566	NA	methyltransferase domain protein	-0.623238881	3.5705E-05	0.293997236	0.001558514	0.699090341	0.000000917
1371	W903_RS06820	W903_1397	1324701	1326845	NA	sulfatase family protein	-0.074388727	0.682977733	-0.567651659	3.98817E-08	-0.020453144	0.913965151
1372	W903_RS06825	W903_1398	1327086	1327445	rplT	ribosomal protein L20	-0.504123731	0.018433975	-1.306325271	6.86701E-11	-0.694421398	0.00000694
1373	W903_RS06830	W903_1399	1327503	1327703	rpmI	ribosomal protein L35	-1.112150779	4.02772E-10	-1.475473442	5.87667E-20	-0.508169356	0.000106787
1374	W903_RS06835	W903_1400	1327743	1328273	infC	translation initiation factor IF-3	-1.179292007	2.72453E-11	-0.910003297	1.92455E-10	-0.341577215	0.011117806
1375	W903_RS06840	W903_1401	1328434	1329117	cmk	cytidylate kinase	-0.124901878	0.525444689	-0.015115236	0.89958526	-0.17857388	0.227680448
1376	W903_RS06845	W903_1402	1329128	1329652	NA	hypothetical protein	-0.177075791	0.310469581	-0.216295672	0.025995864	-0.049270269	0.755876193
1377	W903_RS06850	W903_1403	1329698	1329895	NA	4Fe-4S binding domain protein	0.263452627	0.251844765	0.329730178	0.007661664	0.411954206	0.006129098
1378	W903_RS06855	W903_1404	1329882	1330373	NA	putative pore forming protein	0.253736828	0.165035668	-0.029858032	0.810879367	0.297252657	0.060379126
1379	W903_RS06860	W903_1405	1330402	1331622	pepT	peptidase T	0.13423112	0.435156558	-0.034771615	0.754408822	0.276780507	0.038791958
1380	W903_RS06865	W903_1406	1331758	1333392	NA	polysaccharide biosynthesis family protein	0.813544453	1.44649E-07	0.788578082	4.5371E-16	-1.161729928	2.04E-13
1381	W903_RS06870	W903_1407	1333516	1334970	murE	UDP-N-acetylmuramoyl-L-alanyl-D-glutamate--L-lysine ligase	0.181912597	0.274844361	0.133255122	0.189790019	0.445376156	0.000540928
1382	W903_RS06875	W903_1408	1335125	1335919	NA	ABC transporter family protein	-0.682991415	0.000491514	-0.715610064	1.75685E-08	-0.861089711	0.00073208
1383	W903_RS06880	W903_1409	1335943	1336875	NA	periplasmic binding family protein	-0.568058596	0.000852337	-1.256590812	2.68174E-33	-1.070168703	3.05E-08
1384	W903_RS06885	W903_1410	1336891	1337916	NA	fecCD transport family protein	0.16522813	0.479431807	-0.372344989	0.004450061	-2.251047154	5.38E-40
1385	W903_RS06890	W903_1411	1337913	1338914	NA	fecCD transport family protein	0.279664695	0.251844765	-0.124473906	0.373490113	-2.100047939	5.06E-36
1386	W903_RS06895	W903_1412	1338943	1339596	NA	hypothetical protein	-0.422681444	0.060494851	-0.509160218	0.000937089	0.446276227	0.112865322
1387	W903_RS06900	W903_1413	1339656	1340591	NA	putative manganese-dependent inorganic pyrophosphatase	-0.739396518	6.07421E-07	-1.076197571	4.97235E-34	0.837518018	5.02E-08
1388	W903_RS06905	W903_1414	1340708	1341496	pflA	pyruvate formate-lyase 1-activating enzyme	-0.620320638	0.000214091	0.462063876	2.46811E-05	0.962101064	3.37E-10
1389	W903_RS06910	W903_1415	1341564	1342898	NA	transporter associated domain protein	-0.271095964	0.082629392	0.116846777	0.283039273	-0.508785398	0.000200804
1390	W903_RS06915	W903_1416	1343078	1343644	NA	hypothetical protein	0.548003677	0.0007287	-0.474915844	2.2489E-06	-0.267746337	0.105462883
1391	W903_RS06920	W903_1417	1343637	1344572	NA	radical SAM superfamily protein	0.047599699	0.80486759	-0.533530966	6.88665E-07	0.090400813	0.637118222
1392	W903_RS06925	W903_1418	1344665	1345306	NA	PAP2 superfamily protein	-0.525482118	0.007276705	1.294917334	1.47522E-15	0.500329308	0.0000545
1393	W903_RS06930	W903_1419	1345287	1345871	NA	hypothetical protein	-0.656667285	0.002088991	1.475005754	4.42607E-14	0.787890318	6.53E-11
1394	W903_RS06935	W903_1420	1346172	1347098	NA	LPXTG cell wall anchor domain protein	-1.352033317	2.32386E-14	-2.300720382	4.02072E-68	-0.157048846	0.543036436
1395	W903_RS06940	W903_1421	1347095	1347979	NA	sortase C	-1.216812399	5.73715E-09	-1.87965627	1.47273E-36	-0.319355465	0.244312098
1396	W903_RS06945	W903_1422	1347979	1348857	NA	sortase C	-0.714891231	0.001320461	-1.379970314	1.11852E-20	-0.217588964	0.354562741
1397	W903_RS06950	W903_1423	1348969	1351017	NA	fimbrial isopeptide formation D2 domain protein	-1.053240393	3.45372E-11	-4.047861594	1.0303E-294	0.629713052	0.00000663
1398	W903_RS06955	W903_1424	1351136	1353826	NA	cell wall surface anchor family protein	-1.036081682	5.89644E-08	-3.343663823	5.3409E-229	0.369049068	0.035715209
1399	W903_RS06960	W903_1425	1354110	1355639	NA	M trans-acting positive regulator (MGA) PRD domain protein	-0.169330666	0.523824366	0.085609469	0.507515057	-0.750415946	0.000000151
1400	W903_RS06965	W903_1426	1355691	1356830	NA	glycosyl transferases group 1 family protein	0.055971101	0.774979832	-0.833792203	1.08805E-16	-0.344786226	0.007357139
1401	W903_RS06970	W903_1427	1356820	1357668	NA	glycosyl transferase 2 family protein	0.027227047	0.89099822	-0.86426016	1.57976E-18	-0.156744204	0.34238917
1402	W903_RS06975	W903_1428	1357665	1359089	NA	polysaccharide biosynthesis family protein	0.165919388	0.38790006	-0.616896109	4.88057E-12	-0.178460579	0.197485676
1403	W903_RS06980	W903_1429	1359089	1360453	NA	hypothetical protein	0.207135171	0.295806045	-0.620151506	1.29533E-10	-0.398277746	0.007321985
1404	W903_RS06985	W903_1430	1360455	1361402	NA	glycosyl transferase 2 family protein	0.153283832	0.453150756	-0.581467677	3.69181E-10	0.04421773	0.799319842
1405	W903_RS06990	W903_1431	1361411	1362346	NA	glycosyl transferase 2 family protein	0.165398188	0.377432566	-0.668287461	1.41021E-12	0.230481031	0.100048083
1406	W903_RS06995	W903_1432	1362348	1363403	NA	NAD dependent epimerase/dehydratase family protein	0.142935692	0.433694308	-0.648220384	2.03741E-11	0.403281541	0.003302692
1407	W903_RS07000	W903_1433	1363406	1364125	NA	hypothetical protein	0.25763547	0.16938444	-0.608830928	3.90547E-09	0.397942268	0.010265864
1408	W903_RS07005	W903_1434	1364128	1364952	NA	licD family protein	0.575884936	0.001471444	-0.301392283	0.00212951	0.200746522	0.194664147
1409	W903_RS07010	W903_1435	1364977	1366710	NA	putative lipoprotein	-0.163561998	0.352424613	-0.787202197	6.70563E-18	0.385533962	0.003101714
1410	W903_RS07015	W903_1436	1366703	1367056	NA	hypothetical protein	0.307613914	0.117859086	-0.194404759	0.109006868	0.256130522	0.208618794
1411	W903_RS07020	W903_1437	1367053	1367781	NA	glycosyl transferase 2 family protein	0.164044873	0.344004389	-0.256037243	0.00765911	0.29689863	0.039949875
1412	W903_RS07025	W903_1438	1367786	1368727	NA	glycosyl transferase 2 family protein	-0.158310096	0.362515556	-0.304738451	0.003474711	0.16414625	0.35861038
1413	W903_RS07030	W903_1439	1368717	1369871	NA	glycosyl transferases group 1 family protein	0.07504716	0.688067609	0.316188499	0.001211492	0.175616155	0.250214894
1414	W903_RS07035	W903_1440	1369988	1370842	rfbD	dTDP-4-dehydrorhamnose reductase	-0.30001968	0.130857241	-0.840559022	1.9238E-10	1.210275057	8.98E-16
1415	W903_RS07040	W903_1441	1370932	1371273	NA	hypothetical protein	-0.75874698	9.58718E-06	-0.865067277	1.269E-11	1.005548189	0.00000309
1416	W903_RS07045	W903_1442	1371381	1372490	rpoD	RNA polymerase sigma factor RpoD	-0.603242192	0.000101683	-0.497314123	6.17148E-07	1.020361472	8.35E-14
1417	W903_RS07050	W903_1443	1372498	1374279	dnaG	DNA primase	-0.667026278	6.09867E-06	0.09289736	0.418282977	1.202389441	1.51E-21
1418	W903_RS07055	W903_1444	1374496	1374873	mscL	large conductance mechanosensitive channel protein	0.070286937	0.755212804	0.830611224	2.59101E-09	0.003421427	0.986485815
1419	W903_RS07060	W903_1445	1374970	1375146	rpsU	ribosomal protein S21	-0.586440349	0.007276705	-0.71255138	6.105E-05	-0.719316815	0.007365859
1420	W903_RS07065	W903_1446	1375250	1375753	NA	flagellin N-methylase family protein	-0.567222514	0.012308375	0.641506157	1.20901E-05	-1.223793335	1.41E-08
1421	W903_RS07070	W903_1447	1375836	1376642	NA	bacterial extracellular solute-binding s, 3 family protein	-0.899991089	0.000388739	-0.319556368	0.103344382	0.862299152	0.052916802
1422	W903_RS07075	W903_1448	1376967	1377950	NA	ammonium Transporter family protein	0.546032732	0.376532116	1.24510401	0.000500841	-0.730884815	0.261965365
1423	W903_RS07080	W903_1449	1378090	1379217	NA	pyridine nucleotide-disulfide oxidoreductase family protein	-0.091790479	0.809360802	2.014188166	1.55815E-19	0.680237635	0.111792252
1424	W903_RS07085	W903_1450	1379254	1380240	NA	rhodanese-like domain protein	-0.637637511	0.002162424	1.576122893	6.65344E-36	0.939229698	2.62E-13
1425	W903_RS07090	W903_1451	1380403	1380708	NA	hypothetical protein	-0.343363949	0.324889793	1.307486312	1.19958E-08	-0.344715534	0.334063312
1426	W903_RS07095	W903_1452	1380863	1382236	NA	major Facilitator Superfamily protein	0.667043282	0.000406817	0.860000603	2.5766E-13	-0.3744598	0.016369095
1427	W903_RS07100	W903_1453	1382652	1384916	glgP	glycogen/starch/alpha-glucan phosphorylases family protein	-0.892630195	7.19092E-08	0.363904966	0.133732721	-0.323986512	0.01096057
1428	W903_RS07105	W903_1454	1384928	1386424	malQ	4-alpha-glucanotransferase	-0.994139175	2.08136E-08	0.758370367	0.169343986	-0.301403386	0.018760217
1429	W903_RS07110	W903_1455	1386552	1387574	NA	helix-turn-helix family protein	-0.328887615	0.129985556	-0.316702514	0.014763377	0.127916148	0.479475728
1430	W903_RS07115	W903_1456	1387830	1389077	NA	bacterial extracellular solute-binding family protein	-0.530068348	0.005626831	0.196852718	0.362680838	0.079254517	0.568333981
1431	W903_RS07120	W903_1457	1389205	1390545	NA	binding--dependent transport system inner membrane component family protein	0.056390702	0.814729644	0.384965212	0.121209805	0.027300296	0.886079028
1432	W903_RS07125	W903_1458	1390545	1391381	NA	binding--dependent transport system inner membrane component family protein	-0.267572112	0.280531005	0.35275358	0.069177011	0.224916966	0.137420841
1433	W903_RS07130	W903_1459	1391612	1393084	NA	H+ symporter family protein	0.310924923	0.066830443	0.428299116	0.000876209	-1.270917747	9.11E-19
1434	W903_RS07135	W903_1460	1393129	1393593	NA	NUDIX domain protein	-0.001577055	0.996232944	-0.078329941	0.666663035	0.043884027	0.868645392
1435	W903_RS07140	W903_1461	1393677	1393865	NA	glycerophosphoryl diester phosphodiesterase family protein	-1.236372335	4.39404E-11	-1.619847244	4.67218E-31	0.338551847	0.095578317
1436	W903_RS07145	W903_1462	1393876	1395201	gtfB	accessory Sec system glycosyltransferase GtfB	-0.275524004	0.086116923	-0.839623653	2.2121E-20	0.373495568	0.005834298
1437	W903_RS07150	W903_1463	1395194	1396702	gtfA	accessory Sec system glycosylation protein GtfA	0.285804382	0.106218391	-0.348221773	0.001024914	0.126544247	0.446561965
1438	W903_RS07155	W903_1464	1396716	1399103	secA2	accessory Sec system translocase SecA2	0.399508451	0.014818108	-0.09485244	0.353952072	0.355054668	0.006593344
1439	W903_RS07160	W903_1465	1399090	1400082	asp3	accessory Sec system protein Asp3	0.059443503	0.774979832	-0.227492004	0.05646919	0.275014916	0.060924474
1440	W903_RS07165	W903_1466	1400079	1401638	asp2	accessory Sec system protein Asp2	0.06353573	0.734621538	-0.206363889	0.034195137	0.125677609	0.387395324
1441	W903_RS07170	W903_1467	1401645	1403189	asp1	accessory Sec system protein Asp1	-0.109295595	0.570060513	-0.255690188	0.024498859	0.311372208	0.032578536
1442	W903_RS07175	W903_1468	1403189	1404418	secY2	accessory Sec system translocase SecY2	-0.174418535	0.309794012	-0.117185776	0.234354938	0.425869427	0.0015296
1443	W903_RS07180	W903_1469	1404551	1405738	NA	hypothetical protein	-0.208556877	0.240876774	-0.354757897	0.000137031	0.379925881	0.004357441
1444	W903_RS07185	W903_1470	1405799	1406674	NA	glycosyl transferase 2 family protein	-0.349429768	0.043830037	-0.525180147	2.51002E-07	0.175604559	0.22741219
1445	W903_RS07190	W903_1471	1406667	1407851	NA	glycosyl transferase 8 family protein	0.096903616	0.592240846	-0.190995527	0.091141142	0.064958477	0.688372764
1446	W903_RS07195	W903_1472	1407841	1409082	NA	glycosyl transferase 8 family protein	0.258581752	0.161486188	0.057363028	0.712424109	0.146750021	0.335322464
1447	W903_RS07200	W903_1473	1409079	1410284	NA	glycosyl transferase 8 family protein	0.180525119	0.3320891	-0.083164752	0.476467653	0.163051272	0.275543186
1448	W903_RS07205	W903_1474	1410293	1411300	nss	nucleotide sugar synthetase-like protein	0.274226516	0.142331643	-0.07250433	0.533125342	0.120701631	0.446561965
1449	NA	W903_1475	1411631	1414939	NA	serine-rich repeat adhesion glycoprotein	0.25046965	0.215887609	-1.677266764	8.33241E-57	0.02038562	0.934878411
1450	W903_RS07220	W903_1476	1415322	1416818	NA	M trans-acting positive regulator (MGA) PRD domain protein	-0.055183362	0.821237299	2.652482198	1.3927E-153	-0.183128109	0.156498703
1451	W903_RS07225	W903_1477	1416915	1418906	uvrB	excinuclease ABC subunit B	0.245150765	0.165387542	0.708644946	7.35714E-13	0.277152853	0.052122051
1452	W903_RS07230	W903_1478	1418967	1419887	NA	CAAX protease self-immunity family protein	-0.034021036	0.856711352	0.045475803	0.66458976	-0.308814403	0.070409157
1453	W903_RS07235	W903_1479	1420070	1422253	NA	lysine-arginine-ornithine-binding periplasmic family protein	-0.663510233	0.000490758	0.524757547	0.000550351	-0.788221503	5.96E-11
1454	W903_RS07240	W903_1480	1422253	1422993	NA	ABC transporter family protein	-0.83204429	4.99687E-08	0.288293587	0.024498859	-0.650756713	0.00000039
1455	W903_RS07245	W903_1481	1423187	1423492	NA	hypothetical protein	-0.483434725	0.280296301	0.703947631	0.001601873	1.076744074	0.017177056
1456	NA	W903_1482	1423584	1423742	NA	putative uDP-N-acetylglucosamine 1-carboxyvinyltransferase	2.811408311	3.94437E-06	0.473682686	0.370308421	0.992836685	0.437834739
1457	W903_RS07255	W903_1483	1423768	1425081	cgtA	obg family GTPase CgtA	0.36800494	0.033233312	-1.065583313	2.45937E-20	1.15667421	3.93E-13
1458	W903_RS07260	W903_1484	1425142	1425270	NA	hypothetical protein	-0.156817441	0.784408717	0.574823015	0.111949519	0.412750777	0.510360749
1459	W903_RS07265	W903_1485	1425448	1426689	NA	thermophilic metalloprotease family protein	0.785933113	5.31769E-06	-0.472619266	1.19763E-06	0.553852888	0.0000621
1460	W903_RS07270	W903_1486	1426723	1427301	NA	LPXTG cell wall anchor domain protein	0.191940988	0.344444325	1.016469948	2.83655E-20	0.713326266	0.000000339
1461	W903_RS07275	W903_1487	1427411	1429450	NA	LPXTG cell wall anchor domain protein	0.455599444	0.027363481	1.804565716	5.08301E-57	1.156555203	1.84E-14
1462	W903_RS07280	W903_1488	1429535	1430257	NA	pseudouridine synthase family protein	0.467247953	0.051755221	-1.059213913	2.61028E-08	0.037286037	0.886664735
1463	W903_RS07285	W903_1489	1430448	1431290	NA	aldo/keto reductase family protein	0.474792386	0.048619216	2.560398632	4.21293E-63	0.513004854	0.000666099
1464	W903_RS07290	W903_1490	1431557	1432231	NA	putative NAD(P)H nitroreductase yfkO	1.495668273	1.574E-19	0.471867818	7.08162E-05	1.265988188	3.13E-16
1465	W903_RS07295	W903_1491	1432319	1432711	gloA	lactoylglutathione lyase	0.833028769	0.000110647	1.46441753	4.75203E-29	1.027754855	0.000000005
1466	W903_RS07300	W903_1492	1432840	1433766	NA	glycosyl transferase 2 family protein	0.473002653	0.017858701	0.538883962	3.94801E-05	-0.004278646	0.983035442
1467	W903_RS07305	W903_1493	1433895	1435283	NA	amino acid permease family protein	0.342243802	0.082656145	-0.230022253	0.030623076	-0.153265092	0.288110703
1468	W903_RS07310	W903_1494	1435367	1435834	smpB	ssrA-binding protein	-0.220399263	0.274477724	0.027190957	0.827468074	0.047460249	0.809266568
1469	W903_RS07315	W903_1495	1435837	1438242	rnr	ribonuclease R	-0.04725524	0.798496828	0.482253274	9.54619E-06	0.366977589	0.008690088
1470	W903_RS07320	W903_1496	1438355	1438591	secG	preprotein translocase, SecG subunit	-0.524075059	0.019069696	0.476757164	0.011312258	-0.444328915	0.033895256
1471	W903_RS07325	W903_1497	1438781	1439950	NA	multidrug resistance family protein	-0.083711247	0.664595983	0.824200861	4.20431E-13	-0.078510968	0.6179303
1472	W903_RS07330	W903_1498	1440146	1441792	NA	putative membrane protein	-0.038686485	0.877849387	-0.868243765	1.66499E-08	-0.162914101	0.500416856
1473	W903_RS07335	W903_1499	1441782	1442483	NA	ABC transporter family protein	-0.090113124	0.746248871	-0.956284495	1.8187E-06	-0.390687798	0.214162445

1474	W903_RS07340	W903_1500	1442608	1443195	coaE	dephospho-CoA kinase	0.158385809	0.541626703	0.803492997	2.38004E-08	0.01178253	0.963079211
1475	W903_RS07345	W903_1501	1443192	1444013	mutM	formamidopyrimidine-DNA glycosylase	-0.05941393	0.78773361	0.939175689	8.33189E-14	-0.186212167	0.29270162
1476	W903_RS07350	W903_1502	1444162	1445010	NA	transcriptional activator, Rgg/GadR/MutR family, C-terminal domain protein	-0.091537116	0.78773361	1.428642202	1.09966E-17	-0.311568336	0.152381795
1477	W903_RS07355	W903_1503	1445227	1446819	NA	transglutaminase-like superfamily protein	0.093555423	0.66650765	-1.093687562	2.79629E-13	-0.009833296	0.965825388
1478	NA	W903_1504	1446855	1447031	NA	hypothetical protein	1.467525725	NA	-1.507976142	0.103344382	-0.22317324	0.880701494
1479	NA	W903_1505	1447082	1447207	NA	hypothetical protein	1.027862569	NA	0.805700789	0.263576189	-1.217414224	0.271608447
1480	NA	W903_1506	1447779	1447916	NA	glycerophosphoryl diester phosphodiesterase family protein	0.263367413	0.434399494	0.443797651	0.03654145	-0.280399171	0.42457806
1481	W903_RS07375	W903_1507	1448788	1449177	NA	putative membrane protein	-0.258878541	0.349244784	0.509953603	0.002792875	-0.322413351	0.175795214
1482	W903_RS07380	W903_1508	1449414	1450313	era	GTP-binding protein Era	-0.282537673	0.07935691	-0.578392565	1.57085E-09	0.518421638	0.00019673
1483	W903_RS07385	W903_1509	1450355	1450753	dgkA	undecaprenol kinase	-0.454990229	0.004474302	-0.716638094	2.33648E-11	0.712712026	0.0000201
1484	W903_RS07390	W903_1510	1450734	1451219	NA	putative rRNA maturation factor YbeY	-0.343687266	0.050167456	-0.449711681	9.44617E-05	0.521811835	0.006187319
1485	W903_RS07395	W903_1511	1451625	1452431	NA	leucine carboxyl methyltransferase family protein	-0.552979326	0.022827022	-0.698970464	4.24311E-07	0.975239512	0.00013024
1486	W903_RS07400	W903_1512	1452754	1453230	NA	NUDIX domain protein	-1.360624944	2.25122E-10	0.23380444	0.099323405	0.525469247	0.161438417
1487	W903_RS07405	W903_1513	1453422	1454225	NA	hypothetical protein	0.085820399	0.717288774	0.013154843	0.931286353	-0.974268777	0.00000166
1488	W903_RS07410	W903_1514	1454317	1455303	NA	istB-like ATP binding family protein	-0.235142725	0.229316616	-0.565083057	2.54757E-09	0.221886934	0.265635487
1489	W903_RS07415	W903_1515	1455425	1457197	NA	myosin-cross-reactive antigen family protein	0.882197798	6.53542E-08	1.6193005	7.4048E-52	0.311030601	0.011437351
1490	W903_RS07420	W903_1516	1457354	1457569	NA	hypothetical protein	1.078089016	0.001357887	0.148479234	0.600759461	-0.309123311	0.462168118
1491	W903_RS07425	W903_1517	1457566	1458075	msrA	peptide-methionine (S)-S-oxide reductase	0.050705182	0.789718528	-0.112786392	0.331073284	0.067689843	0.730505102
1492	W903_RS07430	W903_1518	1458213	1459067	NA	S1 domain protein	-0.086291112	0.628327964	-0.638733817	2.63292E-06	0.396991762	0.004659454
1493	W903_RS07435	W903_1519	1459185	1459742	frr	ribosome recycling factor	-0.508874171	0.000533842	-0.778378763	2.20851E-14	-0.146349576	0.322798369
1494	W903_RS07440	W903_1520	1459758	1460486	pyrH	UMP kinase	-0.744016864	2.34669E-06	-0.374957225	0.000106603	-0.413428858	0.001083991
1495	W903_RS07445	W903_1521	1460607	1461287	NA	ABC transporter family protein	-0.22013671	0.506939331	0.596402235	0.000132804	-2.057356977	2.8E-38
1496	W903_RS07450	W903_1522	1461274	1462062	NA	ABC transporter family protein	0.194873912	0.570214659	0.861179295	5.48434E-08	-1.912952254	1.01E-34
1497	W903_RS07455	W903_1523	1462050	1462856	NA	binding--dependent transport system inner membrane component family protein	0.125365886	0.808409811	0.295288423	0.173092651	-2.161171194	1.17E-45
1498	W903_RS07460	W903_1524	1462856	1463800	NA	binding--dependent transport system inner membrane component family protein	0.73160318	0.157715534	0.034952251	0.894008319	-1.955338747	4.86E-38
1499	W903_RS07465	W903_1525	1463787	1465403	nikA	nickel ABC transporter, nickel/metallophore periplasmic binding protein	-0.624090235	0.054953125	-0.698927734	7.05339E-05	-2.133275585	4.23E-45
1500	W903_RS07470	W903_1526	1465792	1466481	rplA	ribosomal protein L1	-0.939526627	1.88838E-07	-1.705022444	1.69726E-86	-0.251329585	0.081505483
1501	W903_RS07475	W903_1527	1466585	1467010	rplK	ribosomal protein L11	-1.150454003	3.19259E-09	-2.068415977	1.2916E-130	-0.213127115	0.121490111
1502	W903_RS07480	W903_1528	1467217	1468599	norB	quinolone resistance protein norB	-0.600772361	0.00034075	-0.858032102	9.87016E-13	-0.452410013	0.008180333
1503	W903_RS07485	W903_1529	1468607	1469806	NA	amidohydrolase family protein	-0.247225636	0.169884599	-0.637683533	9.4038E-06	-0.797255977	0.000000898
1504	W903_RS07490	W903_1530	1470101	1470985	NA	bacterial regulatory helix-turn-helix , lysR family protein	-0.594126369	0.000589242	2.263310676	6.06078E-36	-0.699802267	0.000548223
1505	NA	W903_1531	1471142	1471399	NA	hypothetical protein	-0.792659685	NA	2.05137614	0.173092651	0.153692129	0.938664356
1506	W903_RS07500	W903_1532	1471428	1473143	pabB	aminodeoxychorismate synthase, component I	-0.19522404	0.272689622	-0.043137956	0.705204536	-0.445936621	0.002386258
1507	W903_RS07505	W903_1533	1473225	1475666	NA	ftsK/SpoIIIE family protein	0.264529194	0.099359894	-0.814209374	2.17307E-14	-0.466034342	0.000422751
1508	W903_RS07510	W903_1534	1475841	1476644	NA	cyclophilin type peptidyl-prolyl cis-trans isomerase/CLD family protein	-0.791990317	6.03427E-06	-1.410193394	7.38353E-34	0.676474523	0.002277927
1509	W903_RS07515	W903_1535	1476696	1477529	NA	fecCD transport family protein	-0.760484963	5.03682E-05	-2.183815241	6.92982E-35	-1.217307169	2.31E-16
1510	W903_RS07520	W903_1536	1477531	1478247	NA	ABC transporter family protein	-0.767487394	3.14237E-05	-2.225432082	8.62241E-52	-0.954161154	6.93E-15
1511	W903_RS07525	W903_1537	1478418	1479344	fimA	manganese ABC transporter substrate-binding lipoprotein	-1.466350234	1.1629E-19	-2.960544957	1.9021E-146	-0.387084799	0.01096057
1512	W903_RS07530	W903_1538	1479511	1480158	scaR	metalloregulator ScaR	-0.686806594	8.68469E-05	-0.060465832	0.588788665	0.688212377	0.00000174
1513	W903_RS07535	W903_1539	1480198	1480887	mtnN	MTA/SAH nucleosidase	0.579582402	0.000350848	-0.232405192	0.020187344	0.394917382	0.00509521
1514	W903_RS07540	W903_1540	1480897	1481166	NA	putative foldase protein PrsA	0.543767068	0.008170216	-0.452048725	0.000833946	0.904750737	0.000000254
1515	W903_RS07545	W903_1541	1481166	1481720	NA	NUDIX domain protein	0.5546231	0.000383195	-0.513320325	9.4038E-06	1.005938264	2.6E-12
1516	W903_RS07550	W903_1542	1481741	1483120	glmU	UDP-N-acetylglucosamine diphosphorylase/glucosamine-1-phosphate N-acetyltransferase	0.231117109	0.17915471	-0.652526335	6.42432E-11	0.83455673	1.4E-10
1517	W903_RS07555	W903_1543	1483364	1483777	NA	3-demethylubiquinone-9 3-methyltransferase family protein	0.324274203	0.370786992	0.926448307	1.47288E-06	0.614879957	0.016511812
1518	NA	W903_1544	1483790	1483975	NA	putative lactoylglutathione lyase	-0.257601515	0.589651879	0.771380501	0.006479785	1.044752712	0.007530162
1519	W903_RS07565	W903_1545	1484256	1485212	NA	oxidoreductase , NAD-binding Rossmann fold family protein	-0.091371574	0.627955026	-0.085951182	0.57055785	0.329815381	0.032578536
1520	W903_RS07570	W903_1546	1485209	1485661	NA	ASCH domain protein	-0.313987162	0.095763598	-0.118442761	0.433635575	0.64204259	0.000626139
1521	W903_RS07575	W903_1547	1485658	1485903	NA	hypothetical protein	0.306151477	0.150272362	0.276635989	0.09618279	0.895811615	0.000253397
1522	W903_RS07580	W903_1548	1485900	1486598	NA	short chain dehydrogenase family protein	0.356176686	0.065019973	0.206541357	0.150324397	0.214654974	0.234198533
1523	W903_RS07585	W903_1549	1486591	1486827	NA	hypothetical protein	0.522635234	0.070688377	0.25261522	0.329723128	-0.091052856	0.742757559
1524	W903_RS07590	W903_1550	1486917	1487093	NA	putative ybaK/prolyl-tRNA synthetase associated domain-containing protein	-0.283133381	0.244713212	-0.401437743	0.008198443	-0.404547416	0.029982601
1525	W903_RS07595	W903_1551	1487329	1487862	NA	acetyltransferase family protein	-0.273918839	0.388160613	-0.267214213	0.280364998	-0.460159641	0.059368356
1526	W903_RS07600	W903_1552	1488107	1489519	NA	glycosyl transferase 2 family protein	-0.424766212	0.006621952	-1.251536564	7.30628E-30	-2.431573713	9.45E-100
1527	W903_RS07605	W903_1553	1489659	1491818	NA	hypothetical protein	-0.373340414	0.038740238	-1.214808822	2.70511E-33	-2.519455514	2.07E-99
1528	W903_RS07610	W903_1554	1491825	1493102	NA	cotH family protein	-0.578803539	0.001041071	-1.13155417	1.49653E-30	-2.382567969	4.19E-52
1529	W903_RS07615	W903_1555	1493263	1493940	NA	hypothetical protein	-0.890701892	1.03979E-07	-0.967957117	4.03151E-18	-2.549188686	1.27E-80
1530	W903_RS07620	W903_1556	1493991	1494602	NA	VTC domain protein	-1.362235318	5.1369E-16	-0.004024393	0.978444394	-2.429501749	8.73E-53
1531	W903_RS07625	W903_1557	1495052	1496389	NA	branched-chain amino acid transport system II carrier protein	-0.081376119	0.795848747	0.508468125	0.001960237	-1.197743909	0.000000616
1532	W903_RS07630	W903_1558	1496585	1498582	metG	methionine--tRNA ligase	-0.036185183	0.842711997	-1.169243024	2.9256E-32	0.722490513	0.000000812
1533	W903_RS07635	W903_1559	1498725	1499600	tehB	tellurite resistance protein TehB	0.328262077	0.071814014	0.201065019	0.121134866	-0.181384431	0.268370868
1534	W903_RS07640	W903_1560	1499758	1500453	NA	hypothetical protein	-1.13611211	7.02613E-05	0.272377572	0.173092651	-0.856174916	0.008959062
1535	W903_RS07645	W903_1561	1500789	1502006	NA	phosphotransferase system, EIIC family protein	0.487245433	0.007128116	0.803954377	2.35636E-18	-0.726297264	4.06E-08
1536	W903_RS07650	W903_1562	1501990	1502832	NA	hypothetical protein	1.117049844	5.29956E-10	1.373468754	2.70456E-44	-0.734882513	0.000000983
1537	W903_RS07655	W903_1563	1502909	1503736	NA	exodeoxyribonuclease III family protein	0.530013472	0.002541416	0.80738119	1.69708E-13	-0.850030867	7.5E-12
1538	W903_RS07660	W903_1564	1503778	1504134	NA	putative arsenate reductase	0.091725905	0.78773361	0.157464472	0.449901665	-0.550424376	0.03054793
1539	W903_RS07665	W903_1565	1504136	1504612	NA	methylated-DNA-[]-cysteine S-methyltransferase family protein	0.174550969	0.392991647	0.602078433	4.11763E-07	-0.394345579	0.013297101
1540	W903_RS07670	W903_1566	1504668	1505849	NA	ACT domain protein	0.136885903	0.572454169	-1.760161113	2.7229E-83	1.495754609	3.35E-15
1541	W903_RS07675	W903_1567	1505912	1506460	NA	acetyltransferase, GNAT family	-0.145461667	0.598058959	-2.153080516	3.6168E-120	1.773081541	6.64E-23
1542	W903_RS07680	W903_1568	1506528	1507619	serC	phosphoserine transaminase	0.25304901	0.332406371	-2.091791196	2.0695E-121	1.968273096	1.47E-23
1543	W903_RS07685	W903_1569	1507752	1508387	NA	cutC-like protein	-0.61335632	0.000486794	0.514196394	0.000145166	-0.135574344	0.457213502
1544	W903_RS07690	W903_1570	1508656	1509519	NA	tetrapyrrole (Corrin/Porphyrin) Methylases family protein	-0.605646639	5.88535E-05	-0.211210528	0.04397493	-0.066846483	0.68013203
1545	W903_RS07695	W903_1571	1509525	1509851	NA	hypothetical protein	-1.484304593	4.55491E-26	-0.891051679	1.45923E-10	0.451277455	0.006715033
1546	W903_RS07700	W903_1572	1509882	1510745	NA	DNA polymerase III, delta subunit	-0.726367572	5.28589E-07	-0.571926811	1.47859E-08	0.322386399	0.027508125
1547	W903_RS07705	W903_1573	1510765	1511400	tmk	thymidylate kinase	-0.669798815	5.38044E-06	0.03298252	0.805210061	0.444118761	0.006238549
1548	W903_RS07710	W903_1574	1511489	1512148	NA	CBS domain protein	0.596624575	0.324446752	0.109632238	0.789706167	-0.413879551	0.361245022
1549	W903_RS07715	W903_1575	1512167	1512877	NA	ABC transporter family protein	0.161571638	0.78773361	0.380384798	0.28297559	-0.309895156	0.501681147
1550	W903_RS07720	W903_1576	1512877	1513641	NA	ABC transporter family protein	0.521193052	0.409695168	1.092051177	0.009164051	-0.331678163	0.548868441
1551	W903_RS07725	W903_1577	1513642	1514595	NA	branched-chain amino acid transport system / permease component family protein	0.86243753	0.157715534	0.542924273	0.18028891	-0.424040547	0.389749667
1552	W903_RS07730	W903_1578	1514598	1515467	NA	branched-chain amino acid transport system / permease component family protein	0.539545641	0.482215313	0.865318311	0.05529493	-0.610810819	0.389110381
1553	W903_RS07735	W903_1579	1515575	1516741	NA	receptor ligand binding region family protein	-0.183996811	0.642192846	-0.627935632	0.038074983	-0.406734675	0.614799929
1554	W903_RS07740	W903_1580	1516897	1517142	NA	hypothetical protein	-1.956962722	1.10492E-11	-0.524028485	0.098484959	1.63259873	0.07357623
1555	W903_RS07745	W903_1581	1517242	1517832	clpP	ATP-dependent Clp protease proteolytic subunit	-0.0669727	0.734062253	-0.124502682	0.219762312	1.129335239	2.71E-16
1556	W903_RS07750	W903_1582	1517957	1518586	upp	uracil phosphoribosyltransferase	-0.912166887	2.68394E-09	-1.986976127	8.43205E-92	1.267949034	5.83E-14
1557	W903_RS07755	W903_1583	1518696	1519865	NA	putative beta-cystathionase	0.102053809	0.665634427	0.151514323	0.194418772	0.808183733	0.000086
1558	W903_RS07760	W903_1584	1520000	1520548	NA	putative tRNA (cytidine/uridine-2'-O-)-methyltransferase	-1.229731999	9.39152E-12	-0.426349547	0.000974329	-0.45219331	0.027455957
1559	W903_RS07765	W903_1585	1520721	1522073	NA	amino acid permease family protein	-0.768436618	3.31367E-06	-0.382048348	0.000575682	-0.49773351	0.001960075
1560	W903_RS07770	W903_1586	1522279	1523628	NA	NAD-dependent glycerol-3-phosphate dehydrogenase family protein	0.704267163	2.20021E-06	1.153178235	1.66335E-23	-1.171080007	1.35E-19
1561	W903_RS07775	W903_1587	1523645	1525072	NA	cation transport family protein	0.733631642	1.56293E-06	0.651419608	1.35758E-10	-1.332389749	2.24E-25
1562	W903_RS07780	W903_1588	1525096	1525347	NA	putative membrane protein insertion efficiency factor	0.605144524	0.251844765	-0.029066168	0.943692261	-0.290383943	0.507834653
1563	W903_RS07785	W903_1589	1525347	1526069	NA	pseudouridine synthase family protein	-0.050769837	0.795622196	-0.318217676	0.007747297	-0.134927833	0.384317388
1564	W903_RS07790	W903_1590	1526059	1526643	scpB	segregation and condensation protein B	-0.063099885	0.734062253	0.046632446	0.73319654	0.274131364	0.081188135
1565	W903_RS07795	W903_1591	1526640	1527347	NA	scpA/B family protein	-0.116139405	0.549948356	-0.061508903	0.629127772	0.277138064	0.079310658
1566	NA	W903_1592	1527347	1527793	NA	phage integrase family protein	0.090546769	0.658733956	0.155491743	0.162873965	0.355402431	0.010945575
1567	W903_RS07805	W903_1593	1528077	1528550	NA	CBS domain protein	0.257240403	0.137687003	0.045358237	0.715229253	0.347418562	0.015050738
1568	W903_RS07810	W903_1594	1528547	1529068	NA	phosphodiesterase, MJ0936 family protein	0.241043548	0.179534464	0.027066746	0.866466723	0.371017922	0.010472825
1569	W903_RS07815	W903_1595	1529050	1530024	rdgB	non-canonical purine NTP pyrophosphatase, RdgB/HAM1 family	0.258626909	0.12378284	0.077402623	0.533125342	0.302614306	0.021674525
1570	W903_RS07820	W903_1596	1530021	1530815	murI	glutamate racemase	0.258146556	0.129946656	0.334247173	0.002236428	0.094411103	0.559571908
1571	W903_RS07825	W903_1597	1530990	1531229	NA	hypothetical protein	-0.557995488	0.000751358	-0.029653654	0.832864156	0.123330611	0.507165042
1572	W903_RS07830	W903_1598	1531387	1531929	NA	putative membrane protein	-0.970406436	0.010713828	-0.747686854	0.009454176	0.529624243	0.329644874
1573	W903_RS07835	W903_1599	1531991	1532512	nadR	transcription repressor NadR	0.06162763	0.816605926	-0.251570638	0.075482189	-0.190652427	0.408633907
1574	W903_RS07840	W903_1600	1532580	1533269	NA	inhibitor of apoptosis-promoting Bax1 family protein	0.236403242	0.157666073	0.121572636	0.427621248	0.519329854	0.0000305
1575	W903_RS07845	W903_1601	1533292	1533795	NA	hypothetical protein	0.515120032	0.001094055	0.195563364	0.041109007	0.459383075	0.001352334
1576	W903_RS07850	W903_1602	1533833	1534576	NA	RNA 2'-O ribose methyltransferase substrate binding family protein	0.426162783	0.006387938	0.314831947	0.0067343	0.037881823	0.842810928
1577	W903_RS07855	W903_1603	1534608	1534886	NA	acylphosphatase family protein	-0.054900269	0.823792061	-0.732072257	7.10507E-07	1.898422397	1.73E-14
1578	W903_RS07860	W903_1604	1534971	1535903	NA	membrane insertase, YidC/Oxa1 family domain protein	-0.014905601	0.933659036	-0.782257429	8.14824E-15	0.623062529	0.0000178
1579	W903_RS07865	W903_1605	1536103	1536768	NA	amino ABC transporter, permease , 3-TM region, His/Glu/Gln/Arg/opine family domain protein	0.597966817	0.001470233	1.272259854	1.38725E-17	1.061288833	1.01E-09
1580	W903_RS07870	W903_1606	1536792	1537649	NA	putative ABC transporter extracellular-binding protein yckB	0.213241502	0.306567098	2.168994875	1.58908E-71	1.661862944	5.38E-34
1581	W903_RS07875	W903_1607	1537810	1539270	NA	amidase family protein	0.122852683	0.535771604	-0.32355636	0.039786013	0.067171996	0.717604959
1582	W903_RS07880	W903_1608	1539373	1539855	greA	transcription elongation factor GreA domain protein	-1.180109613	1.27264E-11	-1.641226254	1.22595E-65	0.115191171	0.520729165
1583	W903_RS07885	W903_1609	1539928	1541730	NA	yceG-like family protein	-0.610170692	2.30894E-05	-0.890979987	3.63526E-25	-0.075043603	0.614799929
1584	W903_RS07890	W903_1610	1541817	1542320	NA	acetyltransferase family protein	-0.129094875	0.506982826	-0.801390861	5.02282E-15	0.113552486	0.501190182
1585	W903_RS07895	W903_1611	1542406	1543737	murC	UDP-N-acetylmuramate--alanine ligase	-0.263224996	0.095916955	-0.854411244	1.03001E-19	-0.416704666	0.000770452
1586	W903_RS07900	W903_1612	1543747	1544364	NA	hypothetical protein	-0.431132746	0.010493967	0.06683623	0.549881366	-0.107622985	0.457213502
1587	W903_RS07905	W903_1613	1544529	1547627	NA	helicase domain protein	0.072367082	0.719967852	0.852476158	1.40209E-20	-0.510834228	0.000014
1588	W903_RS07910	W903_1614	1547782	1549092	engA	ribosome-associated GTPase EngA	0.510475203	0.000987298	-0.023774962	0.818696636	1.104277546	1.08E-17
1589	W903_RS07915	W903_1615	1549140	1550042	NA	istB-like ATP binding family protein	0.263313194	0.126321509	0.207123226	0.033305523	0.953630419	1.27E-14
1590	W903_RS07920	W903_1616	1550039	1551214	NA	replication initiation and membrane attachment family protein	-0.049349668	0.798455981	0.107160882	0.411968516	0.865600051	1.8E-10
1591	W903_RS07925	W903_1617	1551214	1551693	nrdR	transcriptional regulator NrdR	-0.307328483	0.127010881	0.249041499	0.046610215	0.847169975	0.0000427
1592	W903_RS07930	W903_1618	1551842	1553347	NA	HAMP domain protein	0.114572055	0.534615849	0.644651706	2.20999E-05	-0.012162924	0.944283724
1593	W903_RS07935	W903_1619	1553337	1554026	NA	putative transcriptional activator CadC	0.226400528	0.223189077	-0.764050538	1.08171E-16	-0.29090967	0.076635901
1594	W903_RS07940	W903_1620	1554262	1554795	NA	hypothetical protein	-0.048833789	0.789718528	-1.066079948	1.844E-22	-0.090484287	0.588651755
1595	W903_RS07945	W903_1621	1554900	1555790	NA	peptidase M48 family protein	0.090845442	0.61223294	-0.707341629	5.01091E-13	-0.244144255	0.117361893
1596	W903_RS07950	W903_1622	1555811	1556365	NA	lemA family protein	-0.093621661	0.64576643	-1.417755589	1.34829E-47	0.516192272	0.001344321
1597	W903_RS07955	W903_1623	1556493	1557206	gidB	16S rRNA (guanine(527)-N(7))-methyltransferase GidB	0.624041052	0.000275248	-0.055951301	0.667464794	-1.071858349	1.44E-09
1598	W903_RS07960	W903_1624	1557221	1558600	NA	cation transport family protein	0.874782077	1.22964E-09	-0.079019465	0.430105865	-0.924914874	2.32E-14
1599	W903_RS07965	W903_1625	1558613	1559284	NA	oxidoreductase , NAD-binding Rossmann fold family protein	1.082631017	9.48589E-12	-0.197069029	0.102643961	-0.857157101	4.79E-09
1600	W903_RS07970	W903_1626	1559319	1560149	NA	cobalt transport family protein	-0.458889039	0.038510502	-0.631935365	1.00107E-08	-0.860833552	1.64E-09
1601	W903_RS07975	W903_1627	1560142	1561818	NA	heme ABC exporter, ATP-binding protein CcmA	-0.322211683	0.122332628	-0.463487593	2.23876E-06	-0.957474427	8.35E-14
1602	W903_RS07980	W903_1628	1561835	1562383	NA	ECF-type riboflavin transporter, S component family protein	-0.792672219	1.4832E-05	-0.376253676	0.000437181	-0.440732694	0.017814214
1603	W903_RS07985	W903_1629	1562552	1563760	NA	dicarboxylate symporter family protein	-0.150639234	0.38120064	0.102741555	0.373490113	-0.291148585	0.053384788
1604	W903_RS07990	W903_1630	1563936	1565303	NA	branched-chain amino acid transport system II carrier protein	0.080810509	0.664768671	-1.563948653	3.50548E-13	-0.039611943	0.937434812
1605	W903_RS07995	W903_1631	1565417	1566472	NA	zinc-binding dehydrogenase family protein	-0.428968291	0.00897114	-0.064962311	0.686314149	0.587371443	0.0000218
1606	W903_RS08000	W903_1632	1566521	1567213	NA	binding--dependent transport system inner membrane component family protein	0.408360774	0.029988359	-0.803080121	3.4262E-09	-0.476824519	0.004547009
1607	W903_RS08005	W903_1633	1567210	1568280	NA	ABC transporter family protein	-0.071717021	0.717464977	-1.016524617	4.76227E-25	-0.511862841	0.000557398

1608	W903_RS08010	W903_1634	1568273	1569649	NA	peptidase M20/M25/M40 family protein	-0.506646448	0.001518599	-0.9503528	6.27566E-16	-0.189634971	0.308765422
1609	W903_RS08015	W903_1635	1569783	1570607	NA	NLPA lipofamily protein	-1.034842418	4.51584E-11	-2.153908117	1.16915E-92	-0.664125827	0.0000308
1610	W903_RS08020	W903_1636	1570748	1571581	NA	bacterial extracellular solute-binding s, 3 family protein	-0.72647848	2.38556E-06	-1.336312988	8.47636E-32	-0.097374036	0.548491797
1611	W903_RS08025	W903_1637	1571683	1572372	NA	glutamine amidotransferase class-I family protein	-1.090098113	5.84055E-11	-0.857684313	7.80248E-09	-0.55688085	0.000158435
1612	W903_RS08030	W903_1638	1572527	1573243	NA	hypothetical protein	-0.867354705	3.31822E-07	-0.619199588	2.50785E-08	-0.145555187	0.427394948
1613	W903_RS08035	W903_1639	1573361	1574347	NA	putative dihydroxyacetone kinase DhaK1b subunit	0.107649368	0.631284966	0.514950067	1.64178E-05	-0.246625619	0.17739869
1614	W903_RS08040	W903_1640	1574357	1574893	dhaS	HTH-type dhaKLM operon transcriptional activator dhaS	-0.793908664	0.030430284	0.08177946	0.803556471	0.602511738	0.13652889
1615	W903_RS08045	W903_1641	1575067	1576056	dhaK	dihydroxyacetone kinase, DhaK subunit	-0.191786197	0.366086675	-0.684561723	2.41817E-10	-0.318141386	0.016566691
1616	W903_RS08050	W903_1642	1576094	1576672	dhaL	dihydroxyacetone kinase, L subunit	0.922864853	8.46431E-08	1.921138118	2.70581E-39	-0.493899497	0.000061
1617	W903_RS08055	W903_1643	1576672	1577046	dhaM	PTS-dependent dihydroxyacetone kinase, phosphotransferase subunit dhaM	0.450765041	0.022280295	1.447891591	3.71227E-28	-0.652483282	0.00000017
1618	W903_RS08060	W903_1644	1577056	1577769	NA	MIP channel s family protein	0.999401842	1.06098E-06	2.039148341	2.71301E-56	-0.763767177	6.36E-09
1619	W903_RS08065	W903_1645	1578034	1578438	NA	cupin domain protein	0.140410552	0.47998015	0.267030548	0.015128732	0.232095744	0.073786513
1620	W903_RS08070	W903_1646	1578577	1579290	NA	merR regulatory family protein	-0.532194715	0.011222826	-0.614706025	4.79752E-05	0.41401352	0.038408961
1621	W903_RS08075	W903_1647	1579383	1580492	NA	HIGH Nucleotidyl Transferase family protein	-0.337337221	0.073842666	-0.516089681	0.001026254	0.045175675	0.841546003
1622	W903_RS08080	W903_1648	1580489	1580740	NA	glycerophosphoryl diester phosphodiesterase family protein	-0.428425783	0.089583515	-0.295502395	0.182676067	0.068832527	0.868645392
1623	W903_RS08085	W903_1649	1580763	1581497	NA	ubiE/COQ5 methyltransferase family protein	-0.354768778	0.075629973	-0.664402708	0.001543421	0.126306758	0.68013203
1624	W903_RS08090	W903_1650	1581563	1581919	NA	oligomerization domain protein	0.331844509	0.107167871	-0.216554995	0.112075635	0.620381346	0.024549784
1625	W903_RS08095	W903_1651	1581921	1582442	NA	isochorismatase family protein	-0.684973593	6.78746E-05	-0.82010594	6.16998E-17	1.06101004	5.32E-12
1626	W903_RS08100	W903_1652	1582505	1583092	NA	HD domain protein	0.086308202	0.705155762	-0.305310928	0.006665851	0.863456373	0.0000722
1627	W903_RS08105	W903_1653	1583089	1583721	nadD	nicotinate (nicotinamide) nucleotide adenylyltransferase	-0.148864183	0.441516503	-0.423804966	7.61299E-05	0.897056366	0.000000118
1628	W903_RS08110	W903_1654	1583851	1584168	NA	CRS1 / YhbY domain protein	-0.385048162	0.044216917	-0.484178516	0.000864787	0.867344561	0.000258653
1629	W903_RS08115	W903_1655	1584261	1585379	yqeH	ribosome biogenesis GTPase YqeH	-0.119190405	0.561356379	-0.037710223	0.7803261	0.569250415	0.005504873
1630	W903_RS08120	W903_1656	1585379	1585906	NA	HAD hydrolase, family IIIA domain protein	-0.519334558	0.01804916	0.49790082	0.005810612	0.66810824	0.112143914
1631	W903_RS08125	W903_1657	1586023	1586937	NA	eamA-like transporter family protein	0.431252506	0.020169789	-0.385728215	0.008164277	0.371645883	0.047247534
1632	W903_RS08130	W903_1658	1587067	1588509	gatB	aspartyl/glutamyl-tRNA(Asn/Gln) amidotransferase, B subunit	-0.058949871	0.74077786	-0.879460594	1.61089E-17	0.677786448	0.00000595
1633	W903_RS08135	W903_1659	1588509	1589975	gatA	aspartyl/glutamyl-tRNA(Asn/Gln) amidotransferase, A subunit	-0.043992114	0.802690043	-0.91415112	2.8338E-25	0.294296429	0.035385705
1634	W903_RS08140	W903_1660	1589975	1590277	gatC	aspartyl/glutamyl-tRNA(Asn/Gln) amidotransferase, C subunit	0.236940127	0.149701198	-0.445749137	0.000169103	0.616279746	0.000166519
1635	W903_RS08145	W903_1661	1590415	1593060	ppdK	pyruvate, phosphate dikinase	0.450989315	0.018088852	1.05559725	1.12079E-18	0.19913582	0.13652889
1636	W903_RS08150	W903_1662	1593073	1593903	NA	putative phosphotransferase yqfL	-0.233232928	0.354583094	0.377117989	0.007874373	0.468624614	0.00110111
1637	W903_RS08155	W903_1663	1593914	1594411	NA	CBS domain protein	0.043404535	0.863740633	0.512029911	0.001702434	0.371598451	0.016042162
1638	W903_RS08160	W903_1664	1594732	1595697	NA	NAD-dependent glycerol-3-phosphate dehydrogenase family protein	-0.361047051	0.086603111	2.114170025	2.5675E-61	0.355879918	0.006600533
1639	W903_RS08165	W903_1665	1595744	1596292	NA	isochorismatase family protein	0.533994689	0.002467368	-1.349400379	1.09941E-30	0.111580393	0.702863249
1640	W903_RS08170	W903_1666	1596359	1597144	codY	GTP-sensing transcriptional pleiotropic repressor CodY	-0.338329503	0.026138651	-2.048935546	1.6998E-103	0.990340053	3.29E-12
1641	W903_RS08175	W903_1667	1597270	1598481	NA	aminotransferase class-V family protein	0.606537621	0.000187386	-1.083511983	5.36243E-22	0.740075142	0.000000131
1642	W903_RS08180	W903_1668	1598748	1599200	NA	universal stress family protein	-0.553441596	0.002150964	0.823880827	2.7804E-12	0.117400979	0.484211778
1643	W903_RS08185	W903_1669	1599256	1600638	NA	cof-like hydrolase family protein	-0.407676918	0.031816433	-0.173232458	0.121735888	0.40908949	0.017216874
1644	W903_RS08190	W903_1670	1600707	1601669	NA	asparaginase family protein	0.746829686	3.70455E-05	-0.194018089	0.220452171	-0.355637438	0.070191841
1645	W903_RS08195	W903_1671	1601704	1602582	aroE	shikimate 5-dehydrogenase	-0.172686779	0.437073844	-1.274970377	1.01929E-24	0.952208888	0.000000475
1646	W903_RS08200	W903_1672	1602680	1603594	NA	aldo/keto reductase family protein	0.316576187	0.106581382	-0.99604639	9.41742E-14	1.0999173	1.6E-12
1647	W903_RS08205	W903_1673	1603885	1605900	recG	ATP-dependent DNA helicase RecG	0.291482664	0.083297881	0.340344434	0.001101518	0.275078785	0.039880851
1648	W903_RS08210	W903_1674	1605980	1607518	NA	mannosyl-glycoendo-beta-N-acetylglucosaminidase family protein	0.422520404	0.007484845	-0.527263253	2.79047E-09	0.314220023	0.012794856
1649	W903_RS08215	W903_1675	1607611	1608711	alr	alanine racemase	0.277316952	0.181018251	-0.010833093	0.941876554	-0.276589256	0.056214563
1650	W903_RS08220	W903_1676	1608708	1609067	acpS	holo-[acyl-carrier-protein] synthase	0.752043287	0.000156836	0.159927324	0.224934497	-0.053599233	0.803440141
1651	W903_RS08225	W903_1677	1609092	1610099	NA	3-deoxy-7-phosphoheptulonate synthase	0.177028982	0.409695168	-0.0375122	0.809786215	-0.167147871	0.249088783
1652	W903_RS08230	W903_1678	1610225	1612753	secA	preprotein translocase, SecA subunit	-0.225012032	0.146022994	-0.936604963	1.24855E-21	0.713203081	0.000000039
1653	W903_RS08235	W903_1679	1612862	1613809	manA	mannose-6-phosphate isomerase, class I	-0.321063012	0.107454755	-0.888280888	5.88431E-20	0.06175223	0.752654096
1654	W903_RS08240	W903_1680	1613927	1614808	NA	ROK family protein	-0.180403659	0.402234902	-0.617218321	0.000454839	-0.645590208	0.00000726
1655	W903_RS08245	W903_1681	1614876	1616795	NA	PTS system, sucrose-specific IIBC component	0.033567387	0.851542185	-1.799433745	2.25867E-44	-1.32027887	1.42E-29
1656	W903_RS08250	W903_1682	1616980	1618419	NA	sucrose-6-phosphate hydrolase family protein	0.456048091	0.003498671	-0.169914789	0.145407556	-1.913273811	6.62E-36
1657	W903_RS08255	W903_1683	1618421	1619383	NA	helix-turn-helix family protein	0.245743107	0.165272261	-0.353347171	0.001702434	-1.72668485	4.08E-37
1658	W903_RS08260	W903_1684	1619470	1619904	nusB	transcription antitermination factor NusB	-0.94956815	1.80137E-09	-1.736868894	8.48765E-66	0.026766001	0.905991133
1659	W903_RS08265	W903_1685	1619897	1620286	NA	hypothetical protein	-1.067868252	7.61316E-14	-1.844933477	2.96241E-66	-0.037989975	0.837242922
1660	W903_RS08270	W903_1686	1620375	1620935	efp	translation elongation factor P	-1.205057963	2.95699E-17	-1.886278289	2.54715E-87	-0.348018358	0.020149626
1661	NA	W903_1687	1620966	1621082	NA	glycerophosphoryl diester phosphodiesterase family protein	-0.040417287	0.914541872	1.471104076	1.92833E-14	-0.957763069	0.000000694
1662	NA	W903_1688	1621664	1621804	NA	hypothetical protein	0.237857486	0.299845493	1.568080412	3.60909E-41	-0.527468864	0.000425844
1663	W903_RS10500	W903_1689	1622455	1622820	NA	hypothetical protein	-1.392591364	5.76347E-07	0.309394394	0.199593713	0.070061301	0.812349466
1664	NA	W903_1690	1622833	1623003	NA	glycerophosphoryl diester phosphodiesterase family protein	0.07228399	0.834002409	0.786482153	0.000104928	0.125697987	0.60369534
1665	W903_RS08280	W903_1691	1623016	1623456	NA	hypothetical protein	-1.188841321	0.000252682	0.32902453	0.332527466	0.469109057	0.682251721
1666	W903_RS10510	W903_1692	1623593	1623715	NA	hypothetical protein	-0.335707993	0.180011398	0.47922254	0.001399302	0.561529681	0.005197441
1667	W903_RS08285	W903_1693	1624058	1624510	NA	cytidine and deoxycytidylate deaminase zinc-binding region family protein	0.228866585	0.277470661	0.382919959	0.000470333	-0.366606879	0.010264349
1668	W903_RS08290	W903_1694	1624522	1625589	pepP	aminopeptidase P	0.125454143	0.500410795	0.090120412	0.372042982	-0.014820101	0.935027856
1669	W903_RS08295	W903_1695	1625689	1626405	NA	hypothetical protein	0.187754774	0.494912393	2.356565611	1.0553E-101	-0.366774536	0.004729178
1670	W903_RS08300	W903_1696	1626407	1627906	NA	H+ antiporter-2 family protein	0.841831808	2.0698E-06	2.952354868	1.8631E-212	-0.424602623	0.001369375
1671	NA	W903_1697	1627994	1628110	NA	hypothetical protein	-0.064920286	0.864846675	3.342082774	4.139E-102	3.170494194	1.78E-55
1672	W903_RS08305	W903_1698	1628242	1631070	uvrA	excinuclease ABC subunit A	1.118089741	5.78596E-14	0.495367346	2.08816E-07	0.207361059	0.13971349
1673	W903_RS08310	W903_1699	1631202	1631873	NA	hypothetical protein	-0.018058289	0.934706308	-0.902314724	2.11074E-16	0.383128308	0.029880226
1674	W903_RS08315	W903_1700	1631898	1632842	NA	corA-like Mg2+ transporter family protein	-0.090695241	0.702717756	-0.637683611	8.56274E-08	0.566310225	0.000640683
1675	W903_RS08320	W903_1701	1633012	1633251	rpsR	ribosomal protein S18	-1.034511286	2.00645E-10	-3.355785491	1.213E-160	0.48017874	0.054279472
1676	W903_RS08325	W903_1702	1633296	1633787	NA	single-stranded DNA-binding family protein	-1.299281718	2.91549E-15	-3.274456858	1.9738E-229	0.694028038	0.00000339
1677	W903_RS08330	W903_1703	1633799	1634086	rpsF	ribosomal protein S6	-1.6598835	1.45243E-24	-3.395225719	1.1708E-193	0.946963475	0.00000021
1678	W903_RS08335	W903_1704	1635149	1636303	mutY	A/G-specific adenine glycosylase	0.496727159	0.003153271	0.901639141	6.42436E-16	-0.597516411	0.000374514
1679	W903_RS08340	W903_1705	1636480	1637073	NA	helix-turn-helix family protein	0.024261442	0.922793656	0.418416837	0.007236236	-0.429117147	0.031814969
1680	W903_RS08345	W903_1706	1637119	1637433	trxA	thioredoxin	-0.305588838	0.085081391	-0.521124961	1.40947E-05	0.292704854	0.150976563
1681	W903_RS08350	W903_1707	1637514	1638014	NA	PAP2 superfamily protein	0.062787814	0.746248871	-0.375542218	0.001038634	0.593626744	0.000144165
1682	W903_RS08355	W903_1708	1638018	1640357	NA	mutS2 family protein	0.298806033	0.055319871	-0.396183666	2.17646E-05	0.456283021	0.000491801
1683	W903_RS08360	W903_1709	1640442	1640984	NA	colicin V production family protein	-0.437535937	0.056189077	0.397960952	0.011548615	0.014482465	0.960946287
1684	W903_RS08365	W903_1710	1640987	1641298	NA	putative cell division protein ZapA	-0.914400992	1.12186E-06	0.619325284	6.31891E-07	0.517098893	0.032006706
1685	W903_RS08370	W903_1711	1641424	1642302	rnhC	ribonuclease HIII	-0.226740455	0.183867077	-0.165891009	0.132142401	-0.849425362	9.19E-08
1686	W903_RS08375	W903_1712	1642318	1642911	NA	signal peptidase I	-0.043703803	0.82365983	-0.672032012	2.10054E-08	-0.451502154	0.017183866
1687	W903_RS08380	W903_1713	1643040	1645460	NA	viral (Super1) RNA helicase family protein	0.148092762	0.529715837	-0.093886011	0.353519471	-0.615512348	0.0000201
1688	W903_RS08385	W903_1714	1645574	1646056	NA	putative membrane protein	-0.032209201	0.876832988	-0.984721966	2.38776E-11	-0.953924462	3.99E-16
1689	W903_RS08390	W903_1715	1646127	1647221	NA	impB/mucB/samB family protein	-0.187801941	0.346377236	0.642274233	5.54563E-09	0.197502821	0.210412292
1690	W903_RS08395	W903_1716	1647406	1649718	pflB	formate acetyltransferase	0.066156732	0.734621538	-0.368353614	0.000119118	0.213148724	0.107767397
1691	W903_RS08400	W903_1717	1649819	1650193	NA	FMN-binding protein	1.087214858	0.000598806	0.336647545	0.083914006	0.201446966	0.353626163
1692	W903_RS08405	W903_1718	1650228	1651157	NA	beta-lactamase family protein	-0.190636157	0.316474166	0.234324978	0.144823595	-1.448140719	2.82E-21
1693	W903_RS08410	W903_1719	1651154	1651909	cppA	C3-degrading proteinase	-0.39353164	0.034343288	0.525403061	1.26896E-05	-0.985146572	9.07E-10
1694	W903_RS08415	W903_1720	1652034	1652930	NA	sulfite exporter TauE/SafE family protein	-0.320964442	0.071230182	-0.370978854	0.002913308	-1.28005077	1.63E-12
1695	W903_RS08420	W903_1721	1653008	1653835	gla	glycerol facilitator-aquaporin gla	-0.398753891	0.021467784	-1.830409754	9.10951E-89	-0.937770526	8.05E-10
1696	W903_RS08425	W903_1722	1654025	1654477	NA	universal stress family protein	1.193577739	8.99585E-08	-0.951818699	1.82646E-22	0.18641665	0.152381795
1697	W903_RS08430	W903_1723	1654495	1655697	NA	sugar (and other) transporter family protein	-0.109093243	0.549536178	-2.038037572	7.56677E-85	0.374164243	0.002082089
1698	W903_RS08435	W903_1724	1655853	1656512	NA	crp-like helix-turn-helix domain protein	-0.03980127	0.842711997	-0.130250416	0.457950226	-1.342761014	2.2E-14
1699	W903_RS08440	W903_1725	1656536	1658821	pepX	xaa-Pro dipeptidyl-peptidase	0.175694371	0.302225232	-0.450326404	1.26448E-05	-1.370905442	3.04E-28
1700	W903_RS08445	W903_1726	1658825	1659184	NA	hypothetical protein	-0.862482773	6.7014E-07	-1.18346694	5.00761E-21	-0.963548612	1.03E-08
1701	W903_RS08450	W903_1727	1659230	1660210	NA	geranylgeranyl pyrophosphate synthase	0.217067731	0.205169742	1.181924403	3.03728E-24	-0.901044502	1.26E-11
1702	W903_RS08455	W903_1728	1660295	1662043	cydC	thiol reductant ABC exporter, CydC subunit	0.54093964	0.000536344	1.206275226	2.40652E-22	0.055153572	0.704262597
1703	W903_RS08460	W903_1729	1662036	1663754	cydD	thiol reductant ABC exporter, CydD subunit	0.585920948	0.000127171	1.519369362	2.86191E-33	0.267794487	0.033250474
1704	W903_RS08465	W903_1730	1663754	1664773	cydB	cytochrome d ubiquinol oxidase, subunit II	0.858998949	1.34288E-08	1.37367034	5.48649E-51	0.041445275	0.777923826
1705	W903_RS08470	W903_1731	1664774	1666201	NA	bacterial Cytochrome Ubiquinol Oxidase family protein	0.988831525	1.15305E-10	1.564354553	1.07232E-59	0.22233734	0.086991754
1706	W903_RS08475	W903_1732	1666304	1667512	NA	pyridine nucleotide-disulfide oxidoreductase family protein	0.362894971	0.025739642	1.352235775	2.38934E-51	0.417446993	0.000590374
1707	W903_RS08480	W903_1733	1667525	1668424	NA	ubiA prenyltransferase family protein	-0.037258108	0.842711997	1.874323182	3.99509E-80	0.453926221	0.000313149
1708	W903_RS08485	W903_1734	1668833	1669279	NA	hypothetical protein	0.905350411	0.00118909	0.822208684	5.81671E-08	0.154839277	0.441797847
1709	NA	W903_1735	1669893	1670039	NA	hypothetical protein	-0.364668948	0.448100444	0.92593623	0.002211472	-0.027495466	0.958321121
1710	W903_RS08490	W903_1736	1670162	1670461	NA	DNA-binding protein, YbaB/EbfC family	-1.47634131	1.18012E-17	-1.360159116	1.53222E-17	1.057590562	0.000172469
1711	W903_RS08495	W903_1737	1670622	1671812	NA	methyltransferase domain protein	0.443418836	0.002888992	0.118795852	0.315767408	0.882807764	2.63E-10
1712	W903_RS08505	W903_1740	1672732	1673319	NA	exonuclease family protein	-0.998160611	3.74698E-07	0.718071314	4.81515E-09	-0.645951354	0.0000201
1713	W903_RS08510	W903_1741	1673363	1673899	NA	hypothetical protein	-1.573353892	4.34334E-20	0.159403395	0.292032486	-1.116365879	1.82E-13
1714	W903_RS08520	W903_1744	1675250	1675906	NA	hypothetical protein	-0.000446989	0.998547303	0.428117957	0.003002164	-0.319404486	0.110521529
1715	W903_RS08525	W903_1745	1676191	1676460	rpsN2	alternate 30S ribosomal protein S14	-1.198762283	1.1711E-15	-3.643587372	1.1196E-181	0.614071148	0.011356328
1716	W903_RS08530	W903_1746	1676822	1677847	NA	aminotransferase class-V family protein	-0.694328079	4.19675E-05	-1.126790433	7.53933E-23	0.995783202	0.000553843
1717	W903_RS08535	W903_1747	1677968	1678978	NA	tRNA threonylcarbamoyl adenosine modification protein YgjD	0.064882628	0.740170779	1.214430442	2.05027E-27	0.834810191	0.000000159
1718	W903_RS08540	W903_1748	1679058	1679465	rimI	ribosomal-protein-alanine acetyltransferase	-0.069942951	0.736690134	0.986263408	2.19824E-21	0.63914385	0.0000726
1719	W903_RS08545	W903_1749	1679467	1680156	yeaZ	tRNA threonylcarbamoyl adenosine modification protein YeaZ	-0.381913915	0.032203904	0.851507035	2.18763E-17	0.17745758	0.268098257
1720	W903_RS08550	W903_1750	1680338	1680568	NA	hypothetical protein	-0.511963432	0.010713828	-0.832381072	1.25276E-07	0.136479961	0.681840652
1721	W903_RS08555	W903_1751	1680622	1682301	NA	putative permease	0.65980316	3.5941E-06	0.032772837	0.76682987	-0.473286322	0.000126326
1722	W903_RS08560	W903_1752	1682469	1682966	NA	CHAP domain protein	-1.593140471	1.53209E-12	0.904958633	5.69726E-07	0.025881302	0.904093136
1723	W903_RS08565	W903_1753	1683126	1684472	glnA	glutamine synthetase, type I	-0.866579555	7.00312E-05	-0.605384517	0.000174009	0.756923587	6.74E-08
1724	W903_RS08570	W903_1754	1684506	1684877	NA	merR regulatory family protein	-1.197445062	7.23226E-09	-0.405995975	0.006700449	0.701276815	0.00000127
1725	W903_RS08575	W903_1755	1684957	1685496	NA	hypothetical protein	-0.092161862	0.727209397	0.48606698	0.00520545	0.2302255	0.359773393
1726	W903_RS08580	W903_1756	1685759	1686955	NA	phosphoglycerate kinase family protein	0.711529679	7.31218E-07	-0.80401642	2.35508E-18	0.876239751	5.75E-09
1727	W903_RS08585	W903_1757	1687090	1687959	NA	5'-nucleotidase, lipoprotein e(P4) family	0.099655843	0.665634427	-0.682747364	1.91425E-05	0.532454904	0.000253292
1728	W903_RS08590	W903_1758	1688168	1689178	gap	glyceraldehyde-3-phosphate dehydrogenase, type I	-0.020352785	0.903260831	-1.242269849	6.55406E-36	1.457074113	6.5E-17
1729	W903_RS08595	W903_1759	1689382	1691460	fusA	translation elongation factor G	0.113783152	0.497060166	-1.285292764	1.07899E-42	0.862761942	7.57E-11
1730	W903_RS08600	W903_1760	1691615	1692085	rpsG	ribosomal protein S7	-0.222014367	0.206759631	-1.759692822	1.42595E-81	0.466409875	0.005203036
1731	W903_RS08605	W903_1761	1692107	1692520	rpsL	ribosomal protein S12	-0.333073829	0.051573608	-1.221159054	4.06291E-21	0.814231074	0.000000747
1732	W903_RS08610	W903_1762	1692739	1693551	purR	pur operon repressor PurR	-0.348252729	0.023937559	-0.530814664	2.64349E-08	0.645762489	0.000000639
1733	W903_RS08615	W903_1763	1693648	1694589	NA	OB-fold nucleic acid binding domain protein	0.275783425	0.089070255	-0.298941746	0.002763438	0.287682594	0.044509617
1734	W903_RS08620	W903_1764	1694579	1695853	NA	rmuC family protein	0.408528379	0.011872739	-0.150510537	0.15555551	0.217427052	0.131215849
1735	W903_RS08625	W903_1765	1695855	1696487	NA	thiamine pyrophosphokinase	0.534666245	0.001743195	0.049444164	0.713960404	-0.156045743	0.390953295
1736	W903_RS08630	W903_1766	1696480	1697142	rpe	ribulose-phosphate 3-epimerase	0.3641407	0.024183283	0.425369246	1.26617E-05	-0.460081161	0.001392418
1737	W903_RS08635	W903_1767	1697149	1698021	rsgA	ribosome small subunit-dependent GTPase A	0.238753238	0.162932846	0.330878401	0.004568194	-0.256598703	0.093404774
1738	W903_RS08640	W903_1768	1698177	1699028	NA	methyltransferase domain protein	-0.222019862	0.257219764	-0.55435461	5.46574E-07	0.406849002	0.067178069
1739	W903_RS08645	W903_1769	1699032	1699904	ksgA	dimethyladenosine transferase	0.093919973	0.650703911	-0.509390284	5.7351E-06	-0.140684504	0.465221378
1740	W903_RS08650	W903_1770	1700079	1700315	NA	glycerophosphoryl diester phosphodiesterase family protein	-0.090320123	0.667345462	-0.31845199	0.011544977	0.387575727	0.101280013
1741	W903_RS08655	W903_1771	1700429	1701046	rnmV	ribonuclease M5	0.055496821	0.786454518	-0.730406626	4.29952E-10	-0.129034001	0.55991878

1742	W903_RS08660	W903_1772	1701033	1701815	NA	hydrolase, TatD family protein	0.198948458	0.333429233	-0.408561603	0.000851918	-0.326257296	0.118890665
1743	NA	W903_1773	1701792	1701905	NA	putative membrane protein	0.495525403	NA	2.121063891	0.008681719	0.292490457	0.821812858
1744	W903_RS08665	W903_1774	1701997	1702278	NA	hypothetical protein	0.168159736	0.402234902	0.221253774	0.224953026	0.84477167	0.0000598
1745	W903_RS08670	W903_1775	1702288	1702680	NA	hypothetical protein	0.211331829	0.331350991	0.257760664	0.22132204	0.520792902	0.016616134
1746	W903_RS08675	W903_1776	1702671	1703963	NA	lipase family protein	0.212189602	0.216011431	-0.024812421	0.843220044	1.061437206	5.13E-11
1747	W903_RS08680	W903_1777	1703963	1704355	NA	hypothetical protein	0.231225716	0.256969376	-0.372487989	0.040120052	1.135931166	0.0000125
1748	W903_RS08685	W903_1778	1704530	1705792	dltD	D-alanyl-lipoteichoic acid biosynthesis protein DltD	-0.349038277	0.039098661	-0.874085647	3.64258E-24	0.04509295	0.768154102
1749	W903_RS08690	W903_1779	1705785	1706024	dltC	D-alanine--poly(phosphoribitol) ligase, subunit 2	0.315071846	0.093926896	-0.443784032	8.79723E-06	-0.09632433	0.576203871
1750	W903_RS08695	W903_1780	1706039	1707304	dltB	D-alanyl-lipoteichoic acid biosynthesis protein DltB	0.064092766	0.733854288	-0.616460379	1.12703E-11	0.002978895	0.986831473
1751	W903_RS08700	W903_1781	1707301	1708836	dltA	D-alanine--poly(phosphoribitol) ligase, subunit 1	0.438996846	0.004405075	-0.511722047	1.98838E-07	-0.051994265	0.749278743
1752	W903_RS08710	W903_1782	1708982	1710169	NA	his Kinase A domain protein	-0.564701267	0.00012166	-0.137704404	0.185762219	-0.504196771	0.000232388
1753	W903_RS08715	W903_1783	1710169	1710843	NA	response regulator	-0.716877253	1.89622E-06	-0.188339115	0.071196555	-0.554178381	0.0000599
1754	W903_RS08720	W903_1784	1711199	1711333	rpmH	ribosomal protein L34	-1.048192663	4.77255E-09	-1.103709876	8.74607E-08	0.123898838	0.806709028
1755	W903_RS08725	W903_1785	1711517	1712872	NA	nucleoside recognition family protein	-0.621208281	0.001094055	-0.794895514	7.55025E-13	-0.471415912	0.004077139
1756	W903_RS08730	W903_1786	1713118	1714845	busAB	betaine ABC transporter permease and substrate binding protein	-2.058855367	1.48427E-33	1.911953586	2.49282E-83	-1.416882489	4.39E-31
1757	W903_RS08735	W903_1787	1714864	1716087	NA	glycine betaine/L-proline transport ATP binding subunit	-1.929986391	2.9184E-29	1.92039778	1.16596E-59	-0.957707556	2.7E-15
1758	NA	W903_1788	1716230	1716349	NA	glycerophosphoryl diester phosphodiesterase family protein	1.98598427	NA	2.756212616	0.105253183	-2.318922459	0.227921022
1759	W903_RS08740	W903_1789	1716464	1718842	xpkA	xylulose-5-phosphate phosphoketolase	0.147474762	0.680975887	0.529123992	0.007816336	-0.753769957	0.000104836
1760	W903_RS08745	W903_1790	1718932	1720023	NA	beta-lactamase superfamily domain protein	-1.09382911	5.35526E-10	-2.37528529	4.21293E-63	0.52279839	0.00528238
1761	W903_RS08750	W903_1791	1720340	1722019	NA	PRD domain protein	-0.482457448	0.001988807	-1.085452177	4.95837E-24	0.984597662	5.51E-09
1762	W903_RS08755	W903_1792	1722085	1722846	NA	xylose isomerase-like TIM barrel family protein	0.212206103	NA	1.229455587	0.038691516	-0.789122041	0.242182735
1763	W903_RS08760	W903_1793	1722865	1724382	NA	badF/BadG/BcrA/BcrD ATPase family protein	1.084277082	0.050329076	2.268588304	3.04639E-15	-1.184230753	0.00000816
1764	W903_RS08765	W903_1794	1724448	1725437	NA	amidohydrolase family protein	0.68603113	0.318469963	2.06112027	7.47916E-10	-0.908835177	0.000630543
1765	W903_RS08770	W903_1795	1725459	1726910	NA	PTS system sugar-specific permease component family protein	0.392539291	0.454068409	2.508017002	1.92798E-20	-0.713648638	0.006599834
1766	W903_RS08775	W903_1796	1727008	1727964	gyaR	glyoxylate reductase	0.507157153	0.058233186	0.853653279	0.000244916	-0.168170887	0.519162999
1767	W903_RS08780	W903_1797	1727983	1729002	NA	hypothetical protein	0.445910152	0.240876774	0.906517818	0.005029472	-0.119184976	0.713104755
1768	W903_RS08785	W903_1798	1729237	1730220	NA	bacterial regulatory s, lacI family protein	-1.332663088	5.40762E-12	-1.411459797	4.5387E-29	1.196775446	0.0000013
1769	W903_RS08790	W903_1799	1730384	1731031	fsa	fructose-6-phosphate aldolase	0.079215501	NA	2.867450652	4.55025E-08	-0.264379794	0.473436008
1770	W903_RS08795	W903_1800	1731042	1731746	NA	class II Aldolase and Adducin N-terminal domain protein	0.17353828	0.785262915	2.724692757	3.41371E-25	-0.276662517	0.196824439
1771	W903_RS08800	W903_1801	1731760	1732623	NA	xylose isomerase-like TIM barrel family protein	-0.325083744	0.669954532	2.367096818	2.1709E-17	-0.52326195	0.020751806
1772	W903_RS08805	W903_1802	1732627	1733292	NA	orotidine 5'-phosphate decarboxylase / HUMPS family protein	-0.002367485	0.997456768	1.216345263	0.00699783	-0.238024822	0.588651755
1773	W903_RS08810	W903_1803	1733405	1733890	ulaC	ascorbate-specific phosphotransferase enzyme IIA component	-0.746012369	0.352448028	0.819750413	0.218024453	0.461866388	0.360270201
1774	W903_RS08815	W903_1804	1733957	1734235	ulaB	ascorbate-specific phosphotransferase enzyme IIB component	-0.782974168	0.153759352	-0.03142964	0.962400513	1.152728048	0.012128648
1775	W903_RS08820	W903_1805	1734263	1735702	NA	PTS system sugar-specific permease component family protein	-0.438392056	0.014074658	-3.318957402	6.9561E-118	1.851968342	1.53E-15
1776	W903_RS08825	W903_1806	1735870	1736487	NA	putative membrane protein	-0.505489708	0.0048532	0.386930235	0.000893779	-0.274161334	0.057161032
1777	W903_RS10750	W903_1807	1736826	1737119	NA	putative membrane protein	-0.247772735	0.62856232	1.795337772	7.24724E-07	-0.057949771	0.904545002
1778	W903_RS08835	W903_1808	1737142	1738434	purA	adenylosuccinate synthase	0.163200606	0.484301603	-0.245639965	0.0711446	1.181483911	1E-17
1779	W903_RS08840	W903_1809	1738805	1739827	NA	phosphotransferase system, EIIC family protein	-0.281960589	0.113933064	0.462592459	0.009282981	0.918766086	0.000000488
1780	W903_RS08845	W903_1810	1740189	1740863	NA	sensory box protein	-0.54214029	0.035084742	0.105664927	0.551873035	-0.761716663	0.000634803
1781	W903_RS08850	W903_1811	1741033	1743285	gshAB	glutamate--cysteine ligase/gamma-glutamylcysteine synthetase	1.122717602	7.39753E-14	0.273696217	0.009830684	-0.563761424	0.00000907
1782	W903_RS08855	W903_1812	1743409	1744227	NA	5-bromo-4-chloroindolyl phosphate hydrolysis family protein	0.421357043	0.011321873	-0.955673459	6.51055E-19	0.356661041	0.038791958
1783	W903_RS08860	W903_1813	1744224	1745480	NA	toxic anion resistance family protein	0.773978854	2.43314E-07	-0.470943043	4.86716E-05	0.389760493	0.010264349
1784	W903_RS08865	W903_1814	1745620	1746495	NA	hsp33 family protein	-0.124672424	0.484301603	0.193614549	0.059396249	-0.077168386	0.68013203
1785	W903_RS08870	W903_1815	1746479	1747456	NA	TIM-barrel , nifR3 family protein	0.168011556	0.340236882	-0.266988295	0.009454176	-0.29849562	0.067178069
1786	W903_RS08875	W903_1816	1747577	1748218	NA	deoxyguanosine kinase	-0.380226553	0.017385085	-1.339422784	2.72085E-18	-0.202829719	0.301508316
1787	W903_RS08880	W903_1817	1748232	1748723	NA	acetyltransferase family protein	0.157864591	0.446374739	-1.032154292	1.91541E-16	-0.631691427	0.004893651
1788	W903_RS08885	W903_1818	1748943	1751390	NA	istB-like ATP binding family protein	0.366568706	0.020156518	0.17101991	0.165234204	1.286389365	1.65E-24
1789	W903_RS08890	W903_1819	1751387	1751851	ctsR	transcriptional regulator CtsR	-0.257854134	0.202251087	0.53352448	0.000296096	1.420345572	1.2E-09
1790	W903_RS08895	W903_1820	1752024	1752485	NA	small multi-drug export family protein	0.564673968	0.000748712	0.975150827	7.52584E-11	-1.313662292	3.51E-10
1791	W903_RS08900	W903_1821	1752645	1753685	tsf	translation elongation factor Ts	-1.002817767	1.1873E-12	-2.479614173	3.4915E-150	0.15818435	0.278867989
1792	W903_RS08905	W903_1822	1753779	1754549	rpsB	ribosomal protein S2	-1.197429297	5.25843E-13	-0.718622256	4.32787E-09	-0.057497824	0.720982766
1793	W903_RS08910	W903_1823	1754826	1755386	ahpC	peroxiredoxin	0.106791668	0.566833212	0.44407329	0.001406202	0.34604235	0.025925184
1794	W903_RS08915	W903_1824	1755404	1756936	ahpF	alkyl hydroperoxide reductase subunit F	0.377287838	0.020388883	0.824500377	4.71265E-12	-0.084486817	0.560367964
1795	W903_RS08920	W903_1825	1757070	1757140	tRNA	NA	3.58862055	NA	0.640064274	0.889840444	-0.829372112	0.906985371
1796	W903_RS08925	W903_1826	1757212	1759281	NA	sodium/hydrogen exchanger family protein	0.024350129	0.904494477	0.389707137	3.19069E-05	-0.256001075	0.056891679
1797	W903_RS08930	W903_1827	1759283	1759519	NA	hypothetical protein	-0.564285431	0.010372194	0.069172668	0.705439019	0.52682079	0.050577488
1798	W903_RS08935	W903_1828	1759686	1760639	NA	LD-carboxypeptidase family protein	1.57847549	1.59909E-24	0.04239461	0.754725798	0.480156375	0.002276653
1799	W903_RS08940	W903_1829	1760689	1762584	NA	peptidase M13 family protein	0.100668347	0.560572697	-0.536717053	2.53782E-09	1.293161328	1.98E-16
1800	W903_RS08945	W903_1830	1762732	1763715	NA	oxidoreductase , NAD-binding Rossmann fold family protein	1.241598171	2.10787E-13	0.580533905	6.37142E-07	0.321355877	0.030248645
1801	W903_RS08950	W903_1831	1763761	1764912	NA	major Facilitator Superfamily protein	0.123107091	0.532862789	1.016154446	8.79972E-14	-0.849759853	0.000000835
1802	W903_RS08955	W903_1832	1765036	1765680	NA	cyclic nucleotide-binding domain protein	0.199651016	0.41083729	0.705935946	1.13949E-05	-0.102837808	0.635022781
1803	W903_RS08960	W903_1833	1765746	1766360	def	peptide deformylase	-0.212124699	0.295652635	-0.053418469	0.644760747	-0.245344392	0.129064888
1804	W903_RS08965	W903_1834	1766432	1767433	NA	periplasmic binding s and sugar binding domain of LacI family protein	-0.612630321	0.000520967	-0.117741847	0.30573053	0.420630815	0.004930049
1805	W903_RS08970	W903_1835	1767513	1769417	NA	heparinase II/III-like family protein	0.441419016	0.300281871	1.817279985	4.1687E-15	1.201458607	0.000000883
1806	W903_RS08975	W903_1836	1769497	1770312	NA	PTS system mannose/fructose/sorbose IID component family protein	0.652121671	0.335504362	2.192251117	2.78161E-07	-0.054921511	0.845336834
1807	W903_RS08980	W903_1837	1770299	1771165	NA	PTS system sorbose-specific iic component family protein	1.324229028	NA	1.818960378	9.91968E-05	-0.481512222	0.041073973
1808	W903_RS08985	W903_1838	1771201	1771695	NA	PTS system sorbose subIIB component family protein	0.248075814	NA	1.923720464	0.000140452	-0.526187206	0.017102098
1809	W903_RS08990	W903_1839	1771750	1772946	NA	unsaturated glucuronyl hydrolase	0.477534755	0.509464899	1.851647171	2.31732E-08	-0.532306571	0.003943415
1810	W903_RS08995	W903_1840	1772949	1773383	NA	PTS system fructose IIA component family protein	-0.696269897	NA	0.717170993	0.236949627	-0.134861329	0.721797567
1811	W903_RS09000	W903_1841	1773661	1774473	NA	short chain dehydrogenase family protein	2.302661039	0.000740448	2.482918186	1.05919E-05	0.085840882	0.822794125
1812	W903_RS09005	W903_1842	1774490	1775131	NA	ribose/galactose isomerase	1.329868427	NA	2.948395948	2.13528E-06	-0.182123827	0.53749266
1813	W903_RS09010	W903_1843	1775157	1776164	NA	pfkB carbohydrate kinase family protein	0.654423958	NA	3.366898733	4.51641E-07	0.128453869	0.631059232
1814	W903_RS09015	W903_1844	1776176	1776814	NA	2-dehydro-3-deoxyphosphogluconate aldolase/4-hydroxy-2-oxoglutarate aldolase family protein	1.023085894	NA	3.938262657	7.94108E-05	0.127768652	0.757137233
1815	W903_RS09020	W903_1845	1776861	1777475	NA	nitroreductase family protein	-0.75536597	1.83832E-06	0.023198656	0.915869759	1.150692314	4.93E-17
1816	W903_RS09025	W903_1846	1777608	1778033	NA	marR family protein	-0.256830541	0.240481793	-1.096387009	2.09373E-11	0.710209961	0.000668806
1817	W903_RS09030	W903_1847	1778160	1782566	polC	DNA polymerase III, alpha subunit, Gram-positive type	0.242466109	0.137418058	-0.426271925	0.003271423	0.327153839	0.015243328
1818	W903_RS09035	W903_1848	1782689	1783273	NA	mannosyl-glycoendo-beta-N-acetylglucosaminidase family protein	0.392750886	0.115557171	1.642789124	4.53427E-30	-0.144865273	0.619956429
1819	W903_RS09040	W903_1849	1783398	1785236	proS	proline--tRNA ligase	0.312636586	0.05092725	-0.449592564	8.15087E-07	0.035118848	0.837242922
1820	W903_RS09045	W903_1850	1785343	1786602	rseP	RIP metalloprotease RseP	0.797060122	1.43422E-07	0.000669335	0.995648905	-0.212379077	0.106813894
1821	W903_RS09050	W903_1851	1786633	1787427	NA	cytidylyltransferase family protein	1.182985936	7.03393E-14	0.411449217	7.44742E-05	-0.150750753	0.325168963
1822	W903_RS09055	W903_1852	1787442	1788194	uppS	di-trans,poly-cis-decaprenylcistransferase	0.976481453	2.74553E-10	0.943393411	7.19223E-16	0.19175718	0.219302092
1823	W903_RS09060	W903_1853	1788361	1788702	yajC	preprotein translocase, YajC subunit	-0.629888857	3.35718E-05	-1.490840994	1.06913E-29	0.399796911	0.086974904
1824	W903_RS09065	W903_1854	1788779	1789123	NA	thioredoxin family protein	-1.239386845	6.37043E-11	-1.183437968	1.62706E-15	0.646878366	0.024691714
1825	W903_RS09070	W903_1855	1789314	1790477	NA	malic enzyme, NAD binding domain protein	0.476020366	0.304435229	1.328945391	3.10285E-06	-0.399067657	0.239119004
1826	W903_RS09075	W903_1856	1790502	1791839	NA	citrate carrier , CCS family protein	0.228924556	0.64054486	1.814860165	2.93254E-13	-0.770227712	0.0083522
1827	W903_RS09080	W903_1857	1791993	1793519	NA	putative sensor kinase dpiB	-0.061941792	0.749756627	1.232187728	6.6466E-31	-0.916667177	1.28E-09
1828	W903_RS09085	W903_1858	1793521	1794201	NA	response regulator	-0.100731597	0.735007232	1.046856428	1.77402E-11	-0.983820353	2.59E-08
1829	W903_RS09090	W903_1859	1794222	1795217	NA	UDP-glucose 4-epimerase GalE	0.396767748	0.014621274	0.18680356	0.061233605	-0.536821077	0.0000312
1830	W903_RS09095	W903_1860	1795305	1796912	dexB	glucan 1,6-alpha-glucosidase	0.18329191	0.32052743	0.545615542	0.017465327	-0.366404711	0.005107774
1831	W903_RS09100	W903_1861	1797041	1798174	NA	ABC transporter family protein	-0.62686271	1.67942E-05	-0.674809314	3.41538E-12	-0.157841351	0.257676631
1832	W903_RS09105	W903_1862	1798275	1799126	NA	bacterial regulatory , Fis family protein	-0.28797207	0.156525661	-0.651147949	1.71571E-07	1.507280348	2.9E-15
1833	W903_RS09110	W903_1863	1799221	1800117	NA	aldose 1-epimerase family protein	0.469780747	0.549536178	2.340875741	9.20468E-05	0.921783477	0.0000107
1834	W903_RS09115	W903_1864	1800171	1801148	lacD	tagatose 1,6-diphosphate aldolase	0.902731941	0.047311061	1.933487849	3.55339E-05	0.755855817	0.000350822
1835	W903_RS09120	W903_1865	1801150	1802082	lacC	tagatose-6-phosphate kinase	0.750568329	0.014781856	1.915687076	2.56544E-10	0.195996714	0.295440105
1836	W903_RS09125	W903_1866	1802093	1802608	lacB	galactose-6-phosphate isomerase, LacB subunit	0.51252228	0.123170371	1.416979224	1.57061E-10	-0.048624546	0.841546003
1837	W903_RS09130	W903_1867	1802629	1803054	lacA	galactose-6-phosphate isomerase, LacA subunit	1.240007871	7.16966E-05	1.678658436	5.86751E-24	-0.049009782	0.854236911
1838	W903_RS09135	W903_1868	1803301	1805751	NA	BNR repeat-like domain protein	-0.713197212	0.000147486	-0.688516247	1.10426E-06	-0.677352106	0.0000011
1839	W903_RS09140	W903_1869	1805990	1807438	NA	PTS system sugar-specific permease component family protein	0.496412278	0.246836258	2.003000477	1.30212E-12	-0.708109463	0.0000426
1840	W903_RS09145	W903_1870	1807478	1807783	NA	PTS system, Lactose/Cellobiose specific IIB subunit	0.295555851	0.420365776	1.260872598	3.352E-06	-0.308342684	0.161652228
1841	W903_RS09150	W903_1871	1807785	1808249	NA	phosphoenolpyruvate-dependent sugar phosphotransferase system, EIIA 2 family protein	-0.161037089	0.678957096	1.445820488	3.84701E-05	0.162277259	0.431844632
1842	W903_RS09155	W903_1872	1808467	1809243	NA	deoR C terminal sensor domain protein	-0.357080092	0.05821367	-0.261324798	0.054015945	0.417912145	0.026267527
1843	W903_RS09160	W903_1873	1809525	1812107	NA	histidine triad domain protein	0.19746083	0.483314144	0.117504302	0.566340884	-0.763834232	0.00110111
1844	W903_RS09165	W903_1874	1812134	1813057	lmb	lipoprotein	-0.057915715	0.920058563	0.43844838	0.311105051	-1.272372665	0.013126219
1845	W903_RS09170	W903_1875	1813312	1813755	dtd	D-tyrosyl-tRNA(Tyr) deacylase	-0.071786932	0.725515949	-0.892879636	2.02294E-12	1.15358727	1.19E-09
1846	W903_RS09175	W903_1876	1813765	1815981	relA	bifunctional (p)ppGpp synthase/hydrolase RelA	0.262164354	0.110401251	-0.603326177	9.53769E-11	0.811249979	3.02E-08
1847	W903_RS09180	W903_1877	1816241	1818643	yfkN	trifunctional nucleotide phosphoesterase protein YfkN	-0.036379424	0.901111124	0.178128927	0.342788828	-0.758832275	0.000221357
1848	W903_RS09185	W903_1878	1818800	1819255	NA	nrdI protein	0.07942557	0.705759832	-0.885508111	6.45738E-15	0.3640308	0.035025758
1849	W903_RS09190	W903_1879	1819266	1820303	NA	M42 glutamyl aminopeptidase family protein	0.226076305	0.184861237	-0.520346327	1.0302E-07	0.248960837	0.109990222
1850	W903_RS09195	W903_1880	1820303	1820800	NA	AAA domain protein	-0.101602583	0.574080165	-0.438194915	2.35986E-05	0.256847732	0.09063097
1851	NA	W903_1881	1820857	1820976	NA	putative membrane protein	1.148016801	NA	1.091254364	0.184629227	-0.570504367	0.569830054
1852	W903_RS09205	W903_1882	1821022	1822059	NA	bacterial extracellular solute-binding family protein	-0.397462601	0.057187032	0.183833937	0.21848066	-0.487461205	0.002549382
1853	W903_RS09210	W903_1883	1822071	1822844	NA	response regulator	-0.509813018	0.0389296	0.163497498	0.301672743	-0.351045753	0.063868599
1854	W903_RS09215	W903_1884	1822848	1824497	NA	histidine kinase-, DNA gyrase B-, and HSP90-like ATPase family protein	-0.596704615	0.00628389	0.322604854	0.019176813	-0.735679468	0.0000147
1855	W903_RS09220	W903_1885	1824635	1825462	NA	PTS system mannose/fructose/sorbose IID component family protein	0.510558896	0.400428001	1.300089904	1.07978E-05	-1.429946633	0.0000178
1856	W903_RS09225	W903_1886	1825459	1826268	NA	PTS system sorbose-specific iic component family protein	0.629927415	0.280296301	1.811951471	1.40328E-09	-0.998324198	0.006001295
1857	W903_RS09230	W903_1887	1826284	1826775	NA	putative phosphotransferase enzyme IIB component	-0.548171823	0.433931975	0.128224475	0.785179748	-0.521733865	0.184238652
1858	W903_RS09235	W903_1888	1826793	1827218	NA	PTS system fructose IIA component family protein	-1.562085984	0.000841296	-0.785707558	0.010778327	-0.052163732	0.921351455
1859	W903_RS09240	W903_1889	1827465	1828517	NA	putative membrane protein	-0.154223101	0.647719608	-0.747991428	3.63882E-05	-0.891000271	0.00177433
1860	W903_RS10555	W903_1890	1828551	1828733	NA	hypothetical protein	-0.153776473	NA	-0.434852691	0.555258975	-0.51697554	0.61458654
1861	W903_RS09245	W903_1891	1828728	1829891	NA	ABC-2 transporter family protein	-0.005632795	0.976894053	-1.179049078	2.97635E-26	-0.827093899	2.89E-09
1862	W903_RS09250	W903_1892	1829897	1830745	NA	ABC transporter family protein	-0.214400407	0.304901614	-1.280684144	3.24504E-33	-0.596898807	0.0000521
1863	W903_RS09255	W903_1893	1830868	1831239	rgfC	sensor histidine kinase domain protein	-0.822727603	0.000525381	-1.193728301	3.68487E-12	-0.193747843	0.53260373
1864	W903_RS09260	W903_1894	1831236	1831988	NA	response regulator	-0.494915443	0.082441176	-0.455985941	0.014763377	-0.840498482	0.003858981
1865	W903_RS09265	W903_1895	1832265	1833080	NA	endonuclease/Exonuclease/phosphatase family protein	-0.28473305	0.626463743	1.960720837	1.94667E-10	0.023731269	0.911458122
1866	W903_RS09270	W903_1896	1833134	1835317	ptbA	PTS system, glucose subfamily, IIA component domain protein	-0.406382789	0.069411897	2.013888255	2.8476E-27	0.044725318	0.802180629
1867	W903_RS09275	W903_1897	1835475	1837130	NA	his Kinase A domain protein	-0.015379779	0.935760556	0.344681345	0.001740056	-0.5826399	0.0000225
1868	W903_RS09280	W903_1898	1837123	1837800	NA	putative transcriptional activator CadC	0.237654184	0.32718836	0.517649365	5.2319E-05	-0.55502017	0.001616204
1869	W903_RS09285	W903_1899	1837800	1838456	NA	phosphate transport system regulatory protein PhoU	0.699317112	0.094489493	0.906351817	0.000657655	-0.779467493	0.039801773
1870	W903_RS09290	W903_1900	1838453	1839202	NA	phosphate ABC transporter, ATP-binding protein	0.537006539	0.210346037	0.403704864	0.151963522	-0.89611095	0.013820767
1871	W903_RS09295	W903_1901	1839195	1840073	NA	phosphate ABC transporter, permease protein PstA	0.74599229	0.216011431	0.976929731	0.004053678	-1.198809199	0.010426202
1872	W903_RS09300	W903_1902	1840075	1840920	NA	phosphate ABC transporter, permease protein PstC	1.253744795	0.084095498	1.425081536	0.001379337	-1.45081076	0.001524322
1873	W903_RS09305	W903_1903	1840935	1841816	NA	PBP superfamily domain protein	0.739032471	0.106581382	0.914729006	0.001765546	-1.512579911	0.0000162
1874	W903_RS09315	W903_1906	1842502	1843242	NA	RNA methyltransferase, RsmE family protein	0.707876654	7.57372E-06	-0.766757664	2.28973E-08	0.053968059	0.750481516
1875	W903_RS09320	W903_1907	1843242	1844195	prmA	ribosomal protein L11 methyltransferase	1.223357141	5.86408E-15	-0.443152557	0.000430606	0.019099479	0.926016995

1876	W903_RS09325	W903_1908	1844212	1845855	NA	viral (Super1) RNA helicase family protein	0.553717304	0.000299026	-1.201745836	3.84366E-32	1.287581527	3.39E-13
1877	W903_RS09330	W903_1909	1845865	1846335	NA	hypothetical protein	0.269460236	0.3563629	-1.3483521	1.24473E-13	1.201084955	1.03E-09
1878	W903_RS09335	W903_1910	1846409	1846936	NA	hypothetical protein	-0.294050149	0.256969376	0.928556159	4.70087E-11	-0.048951949	0.770615589
1879	W903_RS09340	W903_1911	1846939	1847127	NA	helix-turn-helix family protein	-0.672259617	0.016993817	1.038912052	2.00925E-12	0.977377775	2.8E-12
1880	W903_RS09345	W903_1912	1847746	1848222	NA	putative acetyltransferase	1.314847223	7.04296E-15	1.099584727	3.83179E-20	-0.488175073	0.004241642
1881	W903_RS09350	W903_1913	1848215	1849483	NA	sigma-54 interaction domain protein	0.989227669	7.53022E-12	0.751420034	1.75825E-13	-0.475899903	0.001076653
1882	W903_RS09355	W903_1914	1849765	1849837	tRNA	NA	2.088336601	NA	3.822955837	0.000256494	2.457508635	0.003463875
1883	W903_RS09360	W903_1915	1850026	1850331	NA	hypothetical protein	-0.151312278	0.734062253	0.942616132	5.38624E-05	0.816743597	0.024549784
1884	W903_RS09365	W903_1916	1850315	1850716	NA	hypothetical protein	-0.516214972	0.035040964	0.881225027	7.82426E-06	0.45599679	0.116697017
1885	W903_RS09370	W903_1917	1850704	1851912	NA	lipase family protein	-0.031660205	0.875177314	0.626278092	2.58628E-10	0.275217823	0.063980273
1886	W903_RS09375	W903_1918	1851914	1852246	NA	hypothetical protein	0.044058184	0.858438886	0.77764404	7.0316E-08	0.493420348	0.009964592
1887	W903_RS09380	W903_1919	1852518	1852847	NA	hypothetical protein	-0.504225634	0.51480251	1.509272727	0.001470484	0.213443495	0.62852392
1888	NA	W903_1920	1853743	1853889	NA	hypothetical protein	-0.620765488	0.450275205	1.841980042	0.000155583	-0.040997845	0.965614462
1889	NA	W903_1921	1854327	1854455	NA	hypothetical protein	1.203542895	4.49436E-06	2.448434855	6.13206E-49	-1.298846454	5.97E-14
1890	NA	W903_1922	1855114	1855233	NA	hypothetical protein	-0.712296421	NA	0.700099716	0.17448358	-2.295781532	0.000668576
1891	W903_RS09395	W903_1923	1855334	1855717	NA	hypothetical protein	0.023852356	0.950641272	-0.17114939	0.462725173	-0.076156688	0.882622485
1892	NA	W903_1926	1856750	1856869	NA	putative membrane protein	-0.244198542	NA	-0.105872245	0.864322941	0.520972877	0.523454643
1893	W903_RS09410	W903_1927	1857020	1857214	NA	addiction module antitoxin, RelB/DinJ family protein	-0.393734696	NA	0.524329755	0.35048364	-1.481289582	0.046912801
1894	W903_RS09415	W903_1928	1857350	1857640	NA	hypothetical protein	-1.398064442	0.000992727	-0.134544776	0.642537481	-0.237928879	0.671055155
1895	W903_RS09420	W903_1929	1857925	1858323	NA	hypothetical protein	-0.970082763	4.35696E-06	-0.506379027	0.000373093	-0.21753486	0.347713426
1896	W903_RS09425	W903_1930	1858477	1859199	NA	ABC-2 transporter family protein	-1.184638829	1.0928E-09	-0.710245067	2.21015E-05	-0.268774221	0.18220066
1897	W903_RS09430	W903_1931	1859203	1859820	NA	ABC transporter family protein	-1.014064255	1.52055E-08	-0.336124397	0.001878773	0.246119581	0.275543186
1898	W903_RS09435	W903_1932	1860499	1861353	NA	streptomycin adenylyltransferase family protein	-1.034515223	1.33437E-08	-0.762530094	5.41636E-08	1.538306544	4.18E-17
1899	NA	W903_1933	1861645	1861764	NA	glycerophosphoryl diester phosphodiesterase family protein	-1.009828217	NA	0.34591432	0.729077664	0.541058686	0.770615589
1900	W903_RS09440	W903_1934	1861725	1862321	NA	phosphoribulokinase / Uridine kinase family protein	-0.557805575	0.000243819	0.308295339	0.00776568	0.892988065	1.74E-09
1901	W903_RS09445	W903_1935	1862356	1862913	NA	histidine phosphatase super family protein	-0.420653465	0.062042403	0.899922377	1.04926E-09	-0.141458741	0.403118592
1902	W903_RS09450	W903_1936	1862950	1863285	NA	transcriptional regulator PadR-like family protein	0.119445267	0.625465629	1.341083336	8.02463E-17	-0.478090824	0.00281387
1903	W903_RS09455	W903_1937	1863807	1864295	NA	acetyltransferase family protein	-0.858129344	0.012457606	-0.189166947	0.39066178	-0.264765	0.33236378
1904	W903_RS09460	W903_1938	1864464	1865225	NA	ABC-2 transporter family protein	-0.450201652	0.177720765	0.107965483	0.561814829	-0.252914101	0.242128369
1905	W903_RS09465	W903_1939	1865222	1866124	NA	ABC transporter family protein	-0.699404253	0.010307756	-0.168872503	0.309379118	-0.00599627	0.980820944
1906	W903_RS09470	W903_1940	1866121	1866327	NA	phospholipase D family protein	-1.115061898	0.038932854	-0.629626077	0.045148438	0.164370138	0.729736611
1907	W903_RS09475	W903_1941	1866329	1867405	NA	helix-turn-helix family protein	-0.780810233	0.000234013	-0.030046012	0.83454704	0.347514637	0.043203469
1908	NA	W903_1942	1867492	1867638	NA	glycerophosphoryl diester phosphodiesterase family protein	-1.697992011	NA	3.349244553	0.008678291	-1.457260387	0.251919775
1909	W903_RS09480	W903_1943	1867654	1868268	NA	PAP2 superfamily protein	-0.346415506	0.049102307	-1.584123579	3.8947E-40	-0.324774916	0.096423136
1910	W903_RS09485	W903_1944	1868482	1868799	NA	mazG nucleotide pyrophosphohydrolase domain protein	0.124152702	0.62856232	-1.219418321	2.79824E-12	0.338996793	0.122127324
1911	W903_RS09490	W903_1945	1868796	1869356	NA	putative lysine decarboxylase family protein	0.835505709	2.95629E-05	-0.463381338	0.001718665	0.16941243	0.457213502
1912	W903_RS09495	W903_1946	1869661	1870524	NA	CAAX protease self-immunity family protein	-0.036621259	0.857640534	-1.136283781	1.28132E-13	-0.985573169	5.36E-11
1913	W903_RS09500	W903_1947	1870681	1870983	NA	rhodanese-like domain protein	-0.193996927	0.525444689	1.878396785	6.82964E-47	-0.409712765	0.006243726
1914	W903_RS09505	W903_1948	1871156	1871923	NA	protein B	-0.974021122	1.12582E-05	1.101056964	4.27015E-19	-0.286912205	0.042728121
1915	W903_RS09510	W903_1949	1872709	1874112	NA	major Facilitator Superfamily protein	0.423861457	0.015340361	1.114911067	1.8344E-20	0.575754762	0.000765708
1916	W903_RS09515	W903_1950	1874348	1874974	NA	transcriptional regulator	-0.179786002	0.362457208	-0.518378403	0.000665604	-0.344055454	0.072756357
1917	W903_RS09520	W903_1951	1875046	1875585	NA	AAA domain protein	0.363316973	0.062591184	-0.334824656	0.006456007	-0.195409919	0.305814721
1918	W903_RS09525	W903_1952	1875786	1876871	NA	iron-containing alcohol dehydrogenase family protein	0.715805961	2.82007E-05	0.060876785	0.605746199	-0.395617106	0.00258055
1919	W903_RS09530	W903_1953	1877035	1877742	NA	hypothetical protein	-0.661999869	0.001504386	-1.387274331	8.69805E-32	-0.108146603	0.660371743
1920	W903_RS09535	W903_1954	1878089	1879933	NA	homocysteine S-methyltransferase family protein	0.131807857	0.699641117	0.743946525	5.64354E-05	-0.905146175	0.000038
1921	W903_RS09540	W903_1955	1879978	1882215	metE	5-methyltetrahydropteroyltriglutamate--homocysteine S-methyltransferase	0.199538816	0.551312517	0.745474307	1.65438E-05	-1.046869474	0.000000841
1922	W903_RS09545	W903_1956	1882585	1882908	NA	branched-chain amino acid transport family protein	-0.05401302	NA	-0.144547049	0.835875842	-0.948497741	0.492238969
1923	W903_RS09550	W903_1957	1882898	1883590	NA	azlC family protein	-0.204929624	0.449631468	0.119520289	0.558128426	-0.575919741	0.090456196
1924	W903_RS09555	W903_1958	1883913	1888406	NA	gram-positive signal peptide, YSIRK family domain protein	0.650212267	0.009224565	0.562242566	0.004433204	-0.852457963	0.000748084
1925	W903_RS09560	W903_1959	1888434	1888628	NA	LPXTG cell wall anchor domain protein	0.252585086	NA	0.777355776	0.301442646	-1.59892574	0.209197762
1926	W903_RS09565	W903_1960	1888861	1889547	NA	response regulator	-0.43601328	0.028260269	-0.354888585	0.004331399	-0.430957048	0.010264349
1927	W903_RS09570	W903_1961	1889544	1890932	NA	his Kinase A domain protein	-0.103554469	0.60183357	-0.35162728	0.0080325	-0.843081014	2.76E-09
1928	W903_RS09575	W903_1962	1891025	1891633	NA	putative exported protein	-0.457731914	0.04595567	-0.40433864	0.009660559	-0.683767621	0.000123193
1929	W903_RS09580	W903_1963	1891723	1894224	leuS	leucine--tRNA ligase	0.660723434	3.76096E-06	-0.81807287	1.0077E-20	-0.571922995	0.000065
1930	W903_RS09585	W903_1964	1894601	1895848	NA	sugar (and other) transporter family protein	3.039378551	0.091840069	-0.143967231	0.205053582	1.452936888	2.43E-17
1931	W903_RS09590	W903_1965	1895891	1896733	NA	patatin-like phospholipase family protein	-0.198623618	0.297989939	-0.494759867	1.8647E-05	1.302711459	3.46E-14
1932	W903_RS09595	W903_1966	1896874	1897413	nusG	transcription termination/antitermination factor NusG	-1.208839109	3.22774E-15	-1.378717901	1.16165E-34	0.194599205	0.398784512
1933	W903_RS09600	W903_1967	1897619	1899004	NA	LPXTG cell wall anchor domain protein	-1.304320289	5.1369E-16	-3.255514858	6.1038E-110	-1.03972656	1.13E-14
1934	W903_RS09605	W903_1968	1899317	1899490	secE	preprotein translocase, SecE subunit	-0.047633004	0.858438886	-0.839671211	0.000109533	0.01901988	0.960648167
1935	W903_RS09610	W903_1969	1899765	1902047	NA	penicillin-binding , 1A family protein	0.639938287	3.70455E-05	-0.483043991	3.0871E-07	0.768649611	0.00000339
1936	W903_RS09615	W903_1970	1902091	1902975	NA	pseudouridine synthase, RluA family protein	0.287662222	0.137561528	0.161506342	0.204744079	-0.955934675	0.000536643
1937	W903_RS09625	W903_1973	1904809	1906020	NA	phosphopentomutase	1.177924877	2.68857E-14	0.949149301	4.02823E-24	0.216216623	0.116142955
1938	W903_RS09630	W903_1974	1906087	1906758	deoC	deoxyribose-phosphate aldolase	1.221102238	5.4948E-16	0.98623458	1.37353E-24	0.551641603	0.0000036
1939	W903_RS09635	W903_1975	1906788	1907990	NA	na+ dependent nucleoside transporter family protein	1.111800215	1.41332E-14	1.183843475	1.24836E-39	0.711016097	2.44E-09
1940	W903_RS09640	W903_1976	1908011	1908790	udp	uridine phosphorylase	2.002679947	1.05838E-31	1.132675401	2.38995E-26	1.150024469	7.39E-22
1941	W903_RS09645	W903_1977	1908949	1909686	NA	bacterial regulatory s, gntR family protein	-4.228036374	4.50401E-69	-3.541994742	4.8467E-135	-4.580910737	5.49E-182
1942	W903_RS09655	W903_1978	1910096	1911718	groL	chaperonin GroL	0.536491172	0.000233887	-0.307006683	0.022800768	0.8874294	2.31E-13
1943	W903_RS09660	W903_1979	1911814	1912098	groS	10 kDa chaperonin	0.550375039	0.002144701	-1.079673131	6.46002E-19	1.120389526	5.54E-16
1944	W903_RS09665	W903_1980	1912273	1913076	NA	ABC transporter family protein	0.158973584	0.483314144	-2.377166985	4.72852E-42	1.089830311	2.48E-14
1945	NA	W903_1981	1913081	1913977	NA	branched-chain amino acid transport system / permease component family protein	0.248850788	0.306567098	-2.419194222	3.14119E-43	1.280550131	6.93E-16
1946	NA	W903_1982	1913993	1914955	NA	ABC transporter substrate binding family protein	0.591105321	0.007850889	-3.367332011	5.55691E-63	1.327171063	5.27E-18
1947	W903_RS09680	W903_1983	1915420	1916217	NA	HAD hydrolase, IIB family protein	-0.870796137	1.13824E-06	-0.250340302	0.038978604	0.53516569	0.000465306
1948	W903_RS09685	W903_1984	1916403	1917263	NA	glyoxalase/Bleomycin resistance /Dioxygenase superfamily protein	-0.531549124	0.384231534	1.74167635	1.63268E-13	1.612257104	1.73E-13
1949	W903_RS09690	W903_1985	1917306	1918037	NA	hypothetical protein	-0.741757511	8.09099E-05	-0.130991514	0.310910114	-0.015541135	0.949468353
1950	W903_RS09695	W903_1986	1918394	1919011	nrdG	anaerobic ribonucleoside-triphosphate reductase activating protein	0.171862561	0.546474694	1.310569932	1.21114E-28	-1.183758552	3.22E-15
1951	W903_RS09700	W903_1987	1919084	1919575	NA	acetyltransferase family protein	-0.228400935	0.265487886	1.158714352	3.15483E-25	-0.73207084	3.94E-09
1952	W903_RS09705	W903_1988	1919584	1920516	NA	oxidoreductase , NAD-binding Rossmann fold family protein	-0.177418865	0.338526977	0.820723238	7.02761E-16	-0.550646166	0.0000139
1953	NA	W903_1989	1920529	1920672	NA	putative 30S ribosomal protein S10	0.3653722	0.060886057	0.946500358	6.44316E-21	-0.342650671	0.01169286
1954	W903_RS09715	W903_1990	1920747	1922945	nrdD	anaerobic ribonucleoside-triphosphate reductase	-0.704233078	6.81189E-05	0.030387336	0.795751374	-0.200888456	0.114570995
1955	W903_RS09720	W903_1991	1923044	1924585	NA	putative membrane protein	-0.859302431	1.31967E-09	-0.134888823	0.204453777	-0.795358774	8.05E-12
1956	NA	W903_1992	1924631	1924753	NA	hypothetical protein	0.572714056	0.318469963	0.279113848	0.609439204	-0.558020335	0.434224528
1957	W903_RS09730	W903_1993	1924836	1925153	NA	hypothetical protein	-0.823923718	3.90391E-08	-0.087414761	0.471982532	0.361091278	0.013853254
1958	W903_RS09735	W903_1994	1925179	1925589	NA	hypothetical protein	-0.57802275	0.000308364	0.164554204	0.118151879	0.372445277	0.0083522
1959	W903_RS09740	W903_1995	1925598	1925864	NA	hypothetical protein	0.11731725	0.586984816	0.457282136	0.000157186	1.158368701	6.01E-17
1960	W903_RS09745	W903_1996	1926066	1926464	NA	transcriptional regulator, Spx/MgsR family protein	0.213323993	0.219716273	0.19519702	0.050133133	0.577363568	0.0000124
1961	W903_RS09750	W903_1997	1926680	1927819	recA	protein RecA	0.547046052	0.000260862	-0.196535925	0.126115349	0.300421811	0.070191841
1962	W903_RS09755	W903_1998	1927893	1929152	NA	competence/damage-inducible CinA C-terminal domain protein	0.914318589	1.4888E-09	0.078709915	0.457950226	0.219963551	0.200167635
1963	W903_RS09760	W903_1999	1929241	1929792	NA	methyladenine glycosylase family protein	0.325411333	0.082241734	0.842961329	2.15325E-15	-0.50103723	0.001273266
1964	W903_RS09765	W903_2000	1929815	1930405	ruvA	holliday junction DNA helicase RuvA	0.080339562	0.661340936	0.604183743	3.25462E-09	0.02521782	0.903813611
1965	W903_RS09770	W903_2001	1930407	1931639	NA	major Facilitator Superfamily protein	0.458496306	0.003515823	0.696207937	2.9024E-13	-0.317566931	0.016511812
1966	W903_RS09775	W903_2002	1931671	1933644	mutL	DNA mismatch repair MutL family protein	0.216989254	0.201350226	0.368064369	0.001124196	-0.116195752	0.48384832
1967	W903_RS09780	W903_2003	1933869	1934072	NA	'Cold-shock' DNA-binding domain protein	-1.793581594	0.177260421	-3.621337945	2.7883E-11	2.308661733	0.000202454
1968	W903_RS09785	W903_2004	1934129	1936705	mutS	DNA mismatch repair protein MutS	-0.890461185	7.91414E-09	-1.226116704	2.86833E-27	0.782760915	0.000000223
1969	W903_RS09790	W903_2005	1936762	1937199	NA	arginine repressor	-0.883231549	4.15632E-07	-1.305090775	1.17455E-32	0.829963935	0.0000545
1970	W903_RS09795	W903_2006	1937421	1939112	argS	arginine--tRNA ligase	0.226532149	0.155594447	-0.802961751	1.11911E-13	0.407342888	0.001755024
1971	W903_RS09800	W903_2007	1939200	1939508	uviB	bacteriocin	-0.054275787	0.85392705	-0.944285539	3.46814E-06	-0.937173836	0.001625519
1972	W903_RS09805	W903_2008	1939535	1940407	NA	hypothetical protein	-0.146271005	0.484301603	-0.609877644	5.39408E-08	0.796959067	0.0000198
1973	W903_RS09810	W903_2009	1940515	1941459	NA	hypothetical protein	-0.273178268	0.114842415	-1.210585143	2.6124E-41	0.877238854	0.00000147
1974	W903_RS09815	W903_2010	1941449	1943200	aspS	aspartate--tRNA ligase	-0.136035966	0.42574285	-1.591497581	3.57409E-67	1.124468807	1.33E-12
1975	W903_RS09820	W903_2011	1943293	1944573	hisS	histidine--tRNA ligase	-0.116490384	0.536398445	-1.130289601	5.44202E-31	1.58771796	5.29E-24
1976	W903_RS09825	W903_2012	1944793	1944975	rpmF	ribosomal protein L32	-1.033203143	6.73104E-09	-1.500454515	0.000864787	1.124037443	3.34E-13
1977	W903_RS09830	W903_2013	1944991	1945140	rpmG	ribosomal protein L33	0.336589039	0.102080595	-1.006318353	4.92906E-19	0.454837779	0.016041796
1978	W903_RS09835	W903_2014	1945433	1946047	NA	cadmium resistance transporter family protein	-0.885202701	9.23129E-06	-0.773935076	0.000207777	0.894545087	0.004903017
1979	W903_RS09840	W903_2015	1946059	1946397	NA	bacterial regulatory , arsR family protein	-1.205222859	1.45377E-11	-0.977230095	1.11715E-07	1.195952285	0.0000637
1980	W903_RS09845	W903_2016	1946441	1947856	NA	hypothetical protein	-0.96818615	1.33437E-08	-0.681502384	6.10496E-05	0.808886703	0.002127082
1981	W903_RS09850	W903_2017	1948178	1948627	NA	sigma-70, region 4 family protein	1.421948427	0.068653209	1.499204768	2.78646E-06	-0.816549896	0.004568686
1982	W903_RS09855	W903_2018	1948830	1949150	NA	hypothetical protein	1.767873677	NA	1.687066843	0.005623457	-0.491292722	0.477336856
1983	W903_RS09860	W903_2019	1949150	1950250	NA	ftsK/SpoIIIE family protein	1.799928498	0.000589242	1.08667606	3.03157E-05	-0.846667636	0.003652445
1984	W903_RS09865	W903_2020	1950247	1950852	NA	hypothetical protein	0.015053813	0.98246628	1.249592291	0.000313745	-0.611554607	0.076635901
1985	W903_RS09870	W903_2021	1951051	1951656	NA	plasmid replication family protein	0.155731761	0.844341147	0.516311159	0.237846132	-0.60443634	0.088775232
1986	W903_RS09875	W903_2022	1951649	1951897	NA	merR HTH regulatory family protein	-2.551407256	NA	-0.687838552	0.611805082	-1.307182639	0.301508316
1987	W903_RS09880	W903_2023	1951986	1953251	NA	phage integrase family protein	0.211125078	0.280824017	0.981926879	6.67953E-21	-1.092594032	6.31E-16
1988	W903_RS09885	W903_2024	1953609	1954280	NA	peptidase propeptide and YPEB domain protein	-0.121451704	0.842711997	-2.066406596	6.3639E-11	0.526274905	0.451268237
1989	W903_RS09890	W903_2025	1954365	1955036	NA	response regulator	-0.442736694	0.04309935	0.014100853	0.929147843	-0.046394151	0.85007182
1990	W903_RS09895	W903_2026	1955020	1956384	NA	HAMP domain protein	0.091850704	0.644585685	0.138360195	0.207857147	-0.454093921	0.000820693
1991	W903_RS09900	W903_2027	1956496	1958049	NA	citrate transporter family protein	0.874943039	0.19150187	1.623589449	0.000126246	-0.371127773	0.351708164
1992	W903_RS09905	W903_2028	1958143	1959069	NA	carbamate kinase	0.73399848	0.344511372	1.717749938	9.73771E-05	-0.27666199	0.487309421
1993	W903_RS09910	W903_2029	1959081	1960079	NA	ornithine carbamoyltransferase	0.717719715	0.299030732	1.325929875	0.000455035	-0.005707269	0.990559887
1994	W903_RS09915	W903_2030	1960172	1961467	NA	histidine kinase-, DNA gyrase B-, and HSP90-like ATPase family protein	-0.465776924	0.17915471	0.655771265	0.000638759	0.133759711	0.533404459
1995	W903_RS09920	W903_2031	1961464	1962297	NA	response regulator	-0.421696458	0.405459082	1.010405506	1.66551E-05	0.333554307	0.106790922
1996	W903_RS09925	W903_2032	1962519	1963241	NA	ABC transporter family protein	0.600320552	0.001577285	1.054586337	7.94189E-21	-1.080051052	7.46E-15
1997	W903_RS09930	W903_2033	1963244	1964758	NA	substrate binding domain of ABC-type glycine betaine transport system family protein	0.782197739	2.55269E-06	0.663345581	1.75685E-08	-1.028298192	4.92E-15
1998	W903_RS09935	W903_2034	1964836	1967376	NA	hypothetical protein	0.302759879	0.083297881	0.015460451	0.880187916	-0.199051688	0.131215849
1999	W903_RS09940	W903_2035	1967360	1968109	NA	alpha/beta hydrolase fold family protein	-0.15406601	0.374246483	0.602864026	4.74302E-07	0.204752494	0.13362333
2000	W903_RS09945	W903_2036	1968237	1968593	NA	hypothetical protein	0.008419905	0.99218052	2.255895415	2.06169E-11	-0.792670666	0.000380504
2001	W903_RS09950	W903_2037	1968660	1970978	NA	yhgE/Pip N-terminal domain protein	0.381958588	0.087187835	2.049196976	2.45153E-57	-0.701347184	0.00000159
2002	W903_RS09955	W903_2038	1971117	1971656	NA	bacterial regulatory s, tetR family protein	-1.002874769	0.000217519	0.670045713	0.0002586	0.28704853	0.237252666
2003	W903_RS09960	W903_2039	1971742	1972038	NA	hypothetical protein	0.003533634	0.995623902	1.503969485	5.93146E-06	0.520063178	0.439553611
2004	W903_RS09965	W903_2040	1972282	1972893	rpsD	ribosomal protein S4	-0.662014912	0.000240839	-2.226532275	3.7419E-135	0.442576941	0.001451644
2005	W903_RS09970	W903_2041	1973223	1973510	NA	hypothetical protein	1.046032862	9.94861E-08	1.305065241	1.37829E-10	0.34411823	0.206297331
2006	W903_RS09975	W903_2042	1973522	1974877	dnaB	replicative DNA helicase	0.201517916	0.232940747	0.341477472	9.14611E-05	0.235373183	0.082603039
2007	W903_RS09980	W903_2043	1974920	1975372	rplI	ribosomal protein L9	0.453591747	0.003977185	0.218659435	0.066115276	0.310210987	0.042875742
2008	W903_RS09985	W903_2044	1975378	1977360	NA	DHHA1 domain protein	-0.711684182	1.01233E-06	0.415037193	7.70726E-06	0.564405787	0.00000615
2009	W903_RS09990	W903_2045	1977427	1979328	gidA	tRNA uridine 5-carboxymethylaminomethyl modification enzyme GidA	0.387583186	0.014404326	-1.293714109	1.94062E-32	0.314832281	0.042024364

2010	W903_RS09995	W903_2046	1979498	1980109	NA	marC integral membrane family protein	1.140820888	4.35962E-09	0.536817731	0.007468917	1.033460729	1.41E-15
2011	W903_RS10000	W903_2047	1980146	1981267	trmU	tRNA (5-methylaminomethyl-2-thiouridylate)-methyltransferase	0.618945146	0.000799965	-0.138695512	0.491687749	1.034298666	6.99E-13
2012	W903_RS10005	W903_2048	1981513	1982181	sdaAB	L-serine dehydratase, iron-sulfur-dependent, beta subunit	0.028913711	0.876832988	0.05426951	0.723359137	0.163159672	0.343048092
2013	W903_RS10010	W903_2049	1982196	1983068	sdaAA	L-serine dehydratase, iron-sulfur-dependent, alpha subunit	0.421648709	0.008233986	0.018567459	0.912279779	0.054784823	0.750481516
2014	W903_RS10015	W903_2050	1983203	1983880	NA	transglycosylase SLT domain protein	-2.340634132	3.99635E-32	0.493749481	1.3357E-07	1.411566209	1.01E-14
2015	W903_RS10020	W903_2051	1984013	1984552	NA	lysM domain protein	-2.354301098	4.55491E-26	1.56229905	2.15718E-47	1.443532455	0.00000158
2016	W903_RS10025	W903_2052	1984768	1985562	ecfT	energy-coupling factor transporter transmembrane protein EcfT	0.430804839	0.006108359	-0.128899421	0.445223688	0.737100766	0.0000062
2017	W903_RS10030	W903_2053	1985555	1986397	NA	ABC transporter family protein	0.487784996	0.00234211	-0.233920954	0.197309173	0.907117623	4.04E-08
2018	W903_RS10035	W903_2054	1986373	1987212	NA	ABC transporter family protein	-0.227311389	0.210500581	-0.319410436	0.001702434	0.293788948	0.032778678
2019	W903_RS10040	W903_2055	1987212	1987751	pgsA	CDP-diacylglycerol--glycerol-3-phosphate 3-phosphatidyltransferase	-0.39260945	0.011457307	-0.502635092	1.21981E-07	0.040770813	0.809266568
2020	W903_RS10045	W903_2056	1987877	1989160	NA	peptidase M16 inactive domain protein	-0.271943731	0.155541445	-0.598801922	1.59311E-06	-0.012475083	0.95113423
2021	W903_RS10050	W903_2057	1989162	1990406	NA	peptidase M16 inactive domain protein	-0.930224427	4.54475E-08	-0.712188833	1.22854E-09	0.215377144	0.261314987
2022	W903_RS10055	W903_2058	1990645	1990998	NA	S4 domain protein	-1.124722471	9.23878E-12	0.196026433	0.132791205	0.48566369	0.037931516
2023	W903_RS10060	W903_2059	1991001	1992110	recF	DNA replication and repair RecF family protein	-1.141044264	2.04946E-13	-0.578584449	7.92181E-08	0.429867631	0.008764276
2024	W903_RS10065	W903_2060	1992122	1992988	NA	sugar transport family protein	-0.612517746	0.00012166	0.84356435	2.61559E-13	-0.634946587	0.00000504
2025	W903_RS10070	W903_2061	1993044	1993706	NA	helix-turn-helix family protein	-1.326476902	4.07285E-16	0.757373459	1.3733E-06	-0.147855804	0.523702761
2026	W903_RS10075	W903_2062	1993781	1995262	guaB	inosine-5'-monophosphate dehydrogenase	-0.030935472	0.869731936	-0.266077175	0.007712364	-1.079610896	3.13E-20
2027	W903_RS10080	W903_2063	1995419	1995904	NA	arginine regulator	0.399258056	0.045135298	-0.202432702	0.131126092	-0.500076265	0.004423635
2028	W903_RS10085	W903_2064	1995914	1996594	NA	cyclic nucleotide-binding domain protein	-1.22856987	9.01453E-12	-1.535580212	7.724E-41	-0.118458389	0.503115078
2029	W903_RS10090	W903_2065	1996802	1997506	NA	B3/4 domain protein	-0.677817743	0.013077952	0.514797222	0.014637218	-0.493494552	0.201962156
2030	W903_RS10095	W903_2066	1997773	1999005	arcA	arginine deiminase	-0.863548983	3.08386E-07	0.210407438	0.07780587	0.015848555	0.923096515
2031	NA	W903_2067	1999101	1999442	NA	acetyltransferase family protein	0.040903096	0.872590989	3.198095092	4.4486E-202	0.021610179	0.907463508
2032	W903_RS10105	W903_2068	1999458	2000471	NA	ornithine carbamoyltransferase	0.428846235	0.087948687	4.015566188	8.5186E-250	-0.220466299	0.178465726
2033	W903_RS10110	W903_2069	2000534	2001961	arcD	arginine-ornithine antiporter	0.15560012	0.549536178	4.683935603	2.0375E-297	0.652170747	0.00000744
2034	W903_RS10115	W903_2070	2001982	2002938	NA	carbamate kinase	-0.238916228	0.446374739	4.706848033	0	0.726832219	0.00000218
2035	W903_RS10120	W903_2071	2003048	2004073	trpS	tryptophan--tRNA ligase	-0.918860711	4.12945E-10	-0.120299826	0.277888046	-0.426995199	0.000575314
2036	W903_RS10125	W903_2072	2004181	2004873	NA	hypothetical protein	-1.386788767	2.35475E-12	-0.064309076	0.632450797	0.088715204	0.671907692
2037	W903_RS10130	W903_2073	2005117	2005989	NA	hypothetical protein	-0.93610709	1.44791E-06	-1.20897515	1.34149E-26	-0.884750386	0.000000109
2038	W903_RS10135	W903_2074	2006055	2007674	NA	ABC transporter family protein	0.010577469	0.9495054	-0.701971158	1.73807E-13	-0.054859188	0.761947617
2039	W903_RS10140	W903_2075	2007797	2010376	NA	bacterial membrane YfhO family protein	-0.141927832	0.484301603	0.174797879	0.232430401	-0.02999222	0.8778595
2040	W903_RS10145	W903_2076	2010444	2010517	NA	NA	NA	NA	NA	NA	NA	NA
2041	W903_RS10150	W903_2077	2010547	2010618	rRNA	NA	-1.319861842	NA	2.619633458	0.546966741	4.758259215	0.077620084
2042	W903_RS10155	W903_2078	2010810	2010883	tRNA	NA	5.983581516	NA	-3.965965277	0.334290595	2.011914018	0.44908985
2043	W903_RS10160	W903_2079	2010955	2011434	rlmH	rRNA large subunit m3Psi methyltransferase RlmH	-0.224996669	0.272179322	1.1328715	1.29697E-13	0.507223291	0.006093578
2044	W903_RS10165	W903_2080	2011635	2012864	NA	trypsin family protein	-0.471948839	0.001425449	-1.905234906	5.73704E-52	1.559673944	4.91E-29
2045	W903_RS10170	W903_2081	2012962	2013735	NA	parB/RepB/Spo0J family partition domain protein	-0.065278084	0.750272478	-0.326422211	0.015128732	-0.595535853	0.000662119
