# Supplementary material for: Genome-Wide fitness analysis of group B Streptococcus in human amniotic fluid reveals a transcription factor that controls multiple virulence traits
Source: PLoS Pathog. 2021 Mar 8;17(3):e1009116. doi: 10.1371/journal.ppat.1009116 (PMC7971860; doi:10.1371/journal.ppat.1009116)
Supplement: S1 Table — (PDF) [file ppat.1009116.s003.pdf]

**Supplemental Data 3:** Oligonucleotides Used in this Study

| **Name** | **Sequence (5' to 3')** | **Notes** |
|---|---|---|
| MembProt n87 PS F* | AAATACAAATAAACAACTAAGACAG | Membrane protein protospacer targeting nucleotide 87 |
| MembProt n150 PS F* | AAATAAAATTCATACTTATGTACTG | Membrane protein protospacer targeting nucleotide 150 |
| Hypo n57 PS F* | AAATTTTCAAAGTGAATGAACTGTG | Hypothetical protein protospacer targeting nucleotide 57 |
| Hypo n270 PS F* | AAATAATCTTCTAAACGTGAAATCG | Hypothetical protein protospacer targeting nucleotide 270 |
| *mrvR* n103 PS F* | AAATTCAGAAGGCAATTGCATTCCG | *mrvR* protospacer targeting nucleotide 103 |
| *mrvR* n128 PS F* | AAATATTAAGTTGCCCTGCTAATTG | *mrvR* protospacer targeting nucleotide 128 |
| *deoR* n316 PS F* | AAATAATTTCTCTAAGGTTGTTCCG | *deoR* protospacer targeting nucleotide 316 |
| *deoR* n326 PS F* | AAATTTGCGTAGCTAATTTCTCTAG | *deoR* protospacer targeting nucleotide 326 |
| ABC n103 PS F* | AAATCTTAATTTATCTATCCCTACG | ABC transporter protospacer targeting nucleotide 103 |
| ABC n158 PS F* | AAATAAATTGGGTAAATACTGTGTG | ABC transporter protospacer targeting nucleotide 158 |
| 3015b NT PS F* | AGACCGCGTACTACGCCTATTGGT | Non-targeting (sham) protospacer used in control knockdown strain |
| MembProt qRT-PCR F | TCATCGGAAGTGTCATCCTTT | Membrane protein qRT-PCR primer |
| MembProt qRT-PCR R | CCGGATGTTTCTAGCTTAGACT | Membrane protein qRT-PCR primer |
| Hypo qRT-PCR F | AAGACCTCATAAGCGTCGTAAG | Hypothetical protein qRT-PCR primer |
| Hypo qRT-PCR R | GAGCATTGGCATTTGACTGG | Hypothetical protein qRT-PCR primer |
| *mrvR* qRT-PCR F | GAAGTGGAATTAGAATTTCGTGTAGAG | *mrvR* qRT-PCR primer |
| *mrvR* qRT-PCR R | TGGTACCAACGATCAGCAATAA | *mrvR* qRT-PCR primer |
| *deoR* qRT-PCR F | GGTAGTAACCAATAGTCTTCCTGTT | *deoR* qRT-PCR primer |
| *deoR* qRT-PCR R | GCACCAGTAATTTCTCTGTACTCT | *deoR* qRT-PCR primer |
| ABC qRT-PCR F | TCACTCGTTGTTCTACAGTATCT | ABC protein qRT-PCR primer |
| ABC qRT-PCR R | AATTCCTCCAATTGCCACTAAA | ABC protein qRT-PCR primer |

| | | |
|---|---|---|
| *recA* qRT-PCR F | GTGGGATTGCTGCCTTTATTG | For qRT-PCR normalization |
| *recA* qRT-PCR R | CTGAGTCAGGTTGAGACAAGAG | For qRT-PCR normalization |
| CRISPRi qPCR R | CAGCTGAATACGGACTGGATAT | Reverse qPCR primer used for CRISPRi competition experiment |
| 3015b qPCR F | GCGCCTTATCCGGTAACTATC | qPCR primer binds the 3015b plasmid backbone independent of the protospacer; for qPCR normalization |
| 3015b qPCR R | CTACATACCTCGCTCTGCTAATC | qPCR primer binds the 3015b plasmid backbone independent of the protospacer; for qPCR normalization |
| *mrvR* comp GA F | ACAGCTATGACATGATTACGGATAAAGACCTCCTTAATGTC | For Gibson assembly cloning into pBSU101 double-digested with XbaI and EcoRI |
| *mrvR* comp GA R | TGCATGCCTGCAGGTCGACTCTTAATGAGAGGTAATGGAC | For Gibson assembly into pBSU101 double-digested with XbaI and EcoRI |
| *dmrvR* mut GA F | ATTGGGTACCGGGCCCCC | For Gibson assembly of the *mrvR* allelic exchange knockout cassette into pMBsacB digested with XhoI and NotI |
| *dmrvR* mut GA R | TGGAGCTCCACCGCGGTGGCAAAAAGTAGCAAAACAATGATTGCTATTACCTTAGATTGAGATCG | For Gibson assembly of the *mrvR* allelic exchange knockout cassette into pMBsacB digested with XhoI and NotI |
| 1084 Phage R1 F | ACACCGAAAGACCAAGCGAT | For PCR confirmation of prophage region 1 presence |
| 1084 Phage R1 R | TTACGACGGCGGTATGTACG | For PCR confirmation of prophage region 1 presence |
| 1084 Phage qPCR R1 F | AGGGCAAAGACTCGGTAATG | Prophage region 1 qRT-PCR primer |
| 1084 Phage qPCR R1 R | CATTCTTTATGGGTTCGGGAATTT | Prophage region 1 qRT-PCR primer |
| 1084 Phage R2 F | GGTGACTGTCGTCGTTGTCT | For PCR confirmation of prophage region 2 presence |
| 1084 Phage R2 R | GCCACACGTTGTTGTTCTCC | For PCR confirmation of prophage region 2 presence |
| 1084 Phage qPCR R2 F | CTAGATGCCTGTCCAACTTCTT | Prophage region 2 qRT-PCR primer |

| | | |
|---|---|---|
| 1084 Phage qPCR R2 R | ACTCTATGTCCATCATTCCCTTATC | Prophage region 2 qRT-PCR primer |
| 1084 Phage R3 F | GCTGGTGTTGCTGAAATGCA | For PCR confirmation of prophage region 3 presence |
| 1084 Phage R3 R | CATCATCAGGTACGCGACGA | For PCR confirmation of prophage region 3 presence |
| Erm pFfluc GA F | CACTCTTAAGGTATTTAAAGATACCCCAAGAAG | For Gibson assembly replacement of the kanamycin resistance gene in the luciferase expression plasmid pLZ12Km2-P23R:TA:ffluc with the erythromycin resistance gene (*erm*) |
| Erm pFfluc GA R | CTTTAACTACAAGCTTTTTAGAC | For Gibson assembly replacement of the kanamycin resistance gene in the luciferase expression plasmid pLZ12Km2-P23R:TA:ffluc with the erythromycin resistance gene (*erm*) |
| Luc1 pFfluc GA F | TAAAAAGCTTGTAGTTAAAGGCGACTCATAGAATTATTTCC | For Gibson assembly replacement of the kanamycin resistance gene in the luciferase expression plasmid pLZ12Km2-P23R:TA:ffluc with the erythromycin resistance gene (*erm*) |
| Luc1 pFfluc GA R | AGTAACGATTCATTGACCGCCAATACTCATGAACGGCTTG | For Gibson assembly replacement of the kanamycin resistance gene in the luciferase expression plasmid pLZ12Km2-P23R:TA:ffluc with the erythromycin resistance gene (*erm*) |
| Luc2 pFfluc GA F | GCGGTCAATGAATCGTTAC | For Gibson assembly replacement of the kanamycin resistance gene in the luciferase expression plasmid pLZ12Km2-P23R:TA:ffluc with the erythromycin resistance gene (*erm*) |
| Luc2 pFfluc GA R | CTTTAAATACCTTAAGAGTGTGTTGATAGTG | For Gibson assembly replacement of the kanamycin resistance gene in the luciferase expression plasmid pLZ12Km2-P23R:TA:ffluc with the erythromycin resistance gene (*erm*) |
| Tn-seq Amp F | CAAGCAGAAGACGGCATACGAGGTTGAACTGCTGATCTTCGG | For transposon-genome junction amplification in Tn-seq. See reference 41 |

| | | |
|---|---|---|
| Tn-seq Amp R | AATGATACGGCGACCACCGAGATCACACTCTTT CCCTACACGACGCTCTTCC | For transposon-genome junction amplification in Tn-seq. See reference 41 |

*Forward protospacer oligonucleotide; also used as forward qPCR primer in CRISPRi comptetion experiment
