# Supplementary figures and images for: Genome-Wide fitness analysis of group B Streptococcus in human amniotic fluid reveals a transcription factor that controls multiple virulence traits
Source: PLoS Pathog. 2021 Mar 8;17(3):e1009116. doi: 10.1371/journal.ppat.1009116 (PMC7971860; doi:10.1371/journal.ppat.1009116)

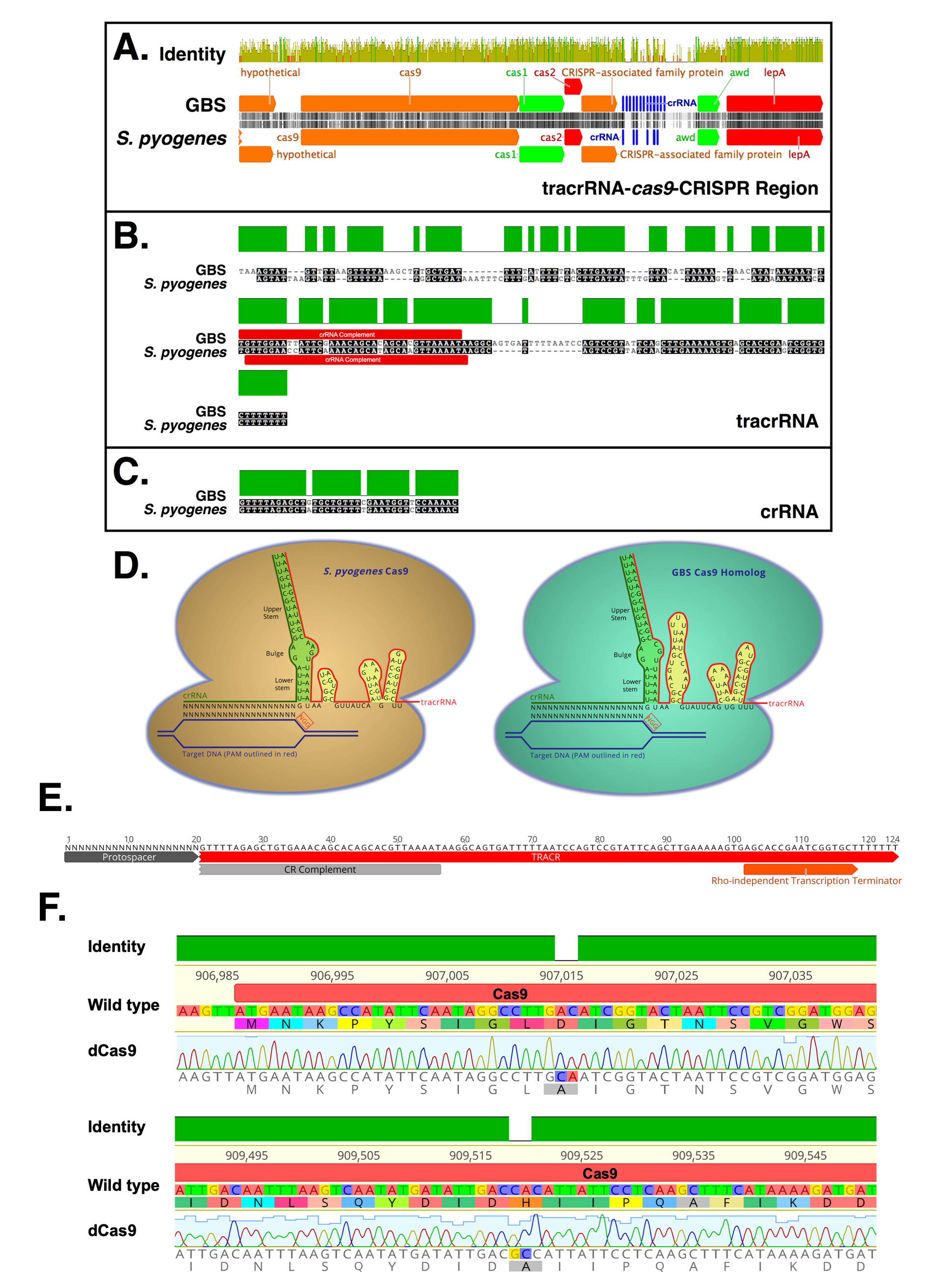

Supplement: S1 Fig — Alignments of GBS and S. pyogenes genomic DNA regions encoding CRISPR-Cas complex components (A-C). Predicted folding of gRNA complexes (D) generated using the mfold server [141]. Sequence of sgRNA complex used to target candidate genes using GBS CRISPRi system (E). Sanger sequence showing the two targeted mutations used to generate dCas9 on the 10/84 chromosome (F). (TIF) [file ppat.1009116.s004.tif]

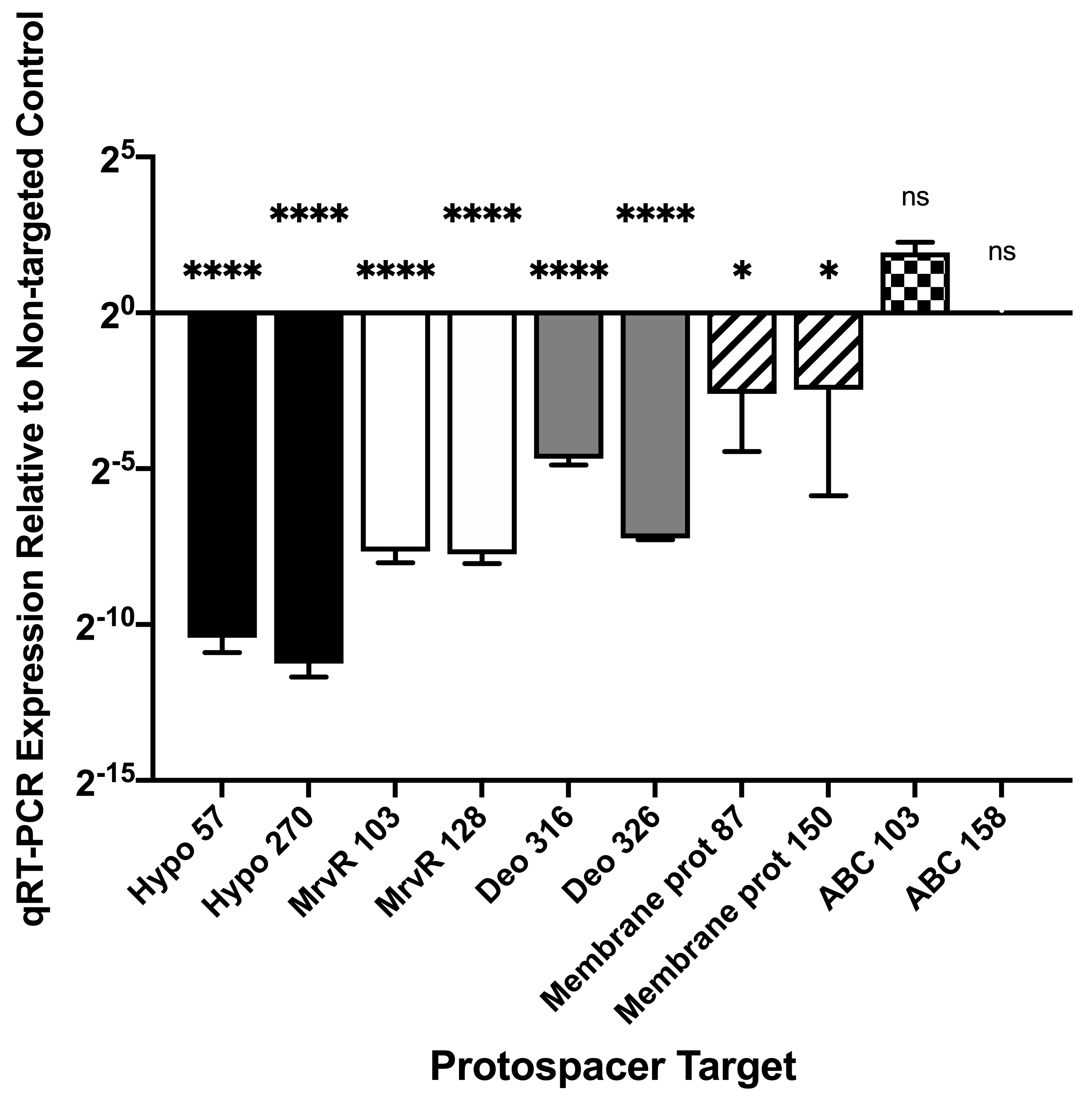

Supplement: S2 Fig — Each silenced strain was assessed for normalized expression of the targeted gene relative to a non-targeted control strain. This experiment was performed with triplicate samples across two independent replicates. (*p<0.05, **** p<0.001, ns = not significant; t test against a single value of 1, error bars show standard error surrounding the mean). (TIF) [file ppat.1009116.s005.tif]

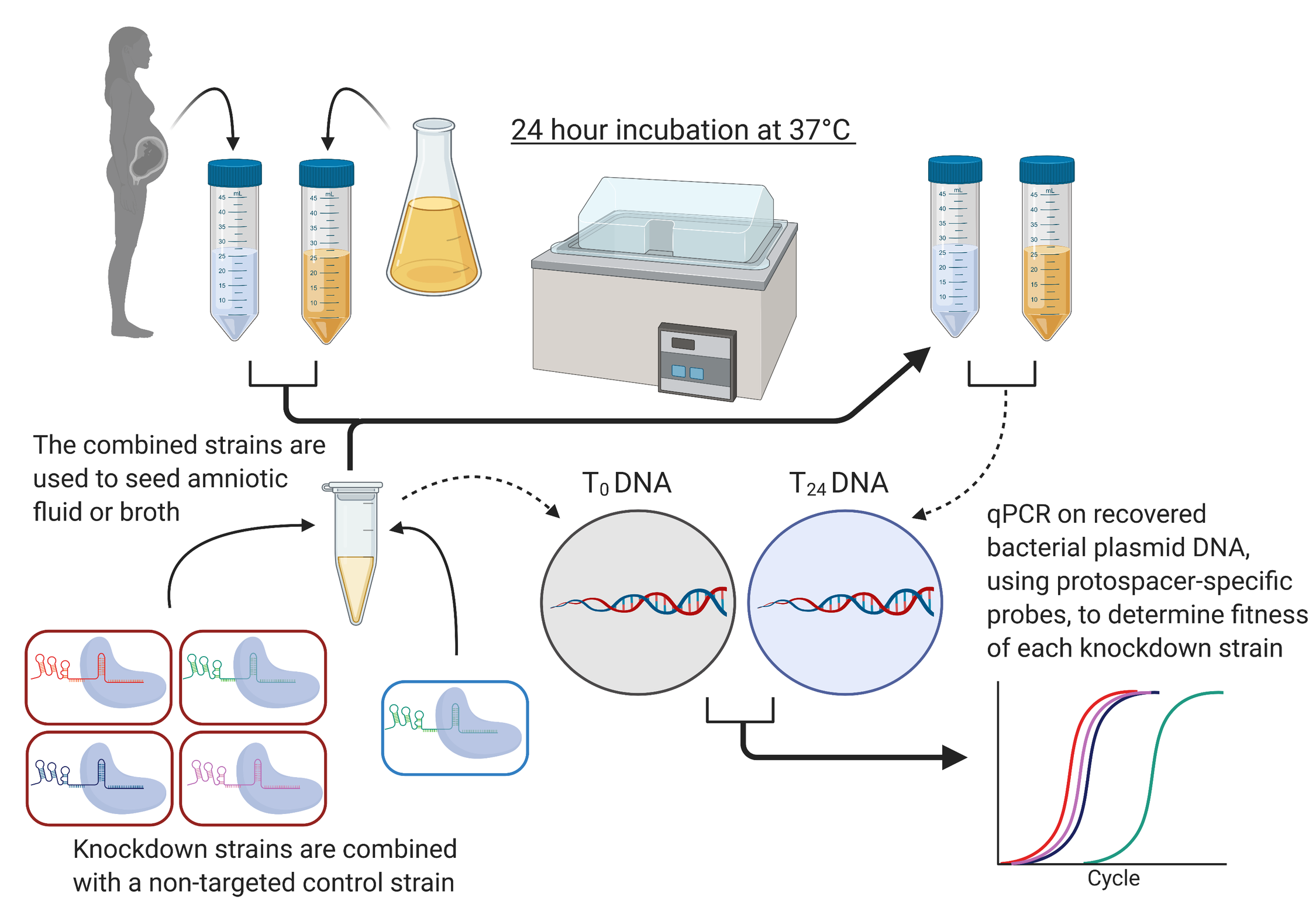

Supplement: S3 Fig — Image created with BioRender.com. (TIF) [file ppat.1009116.s006.tif]

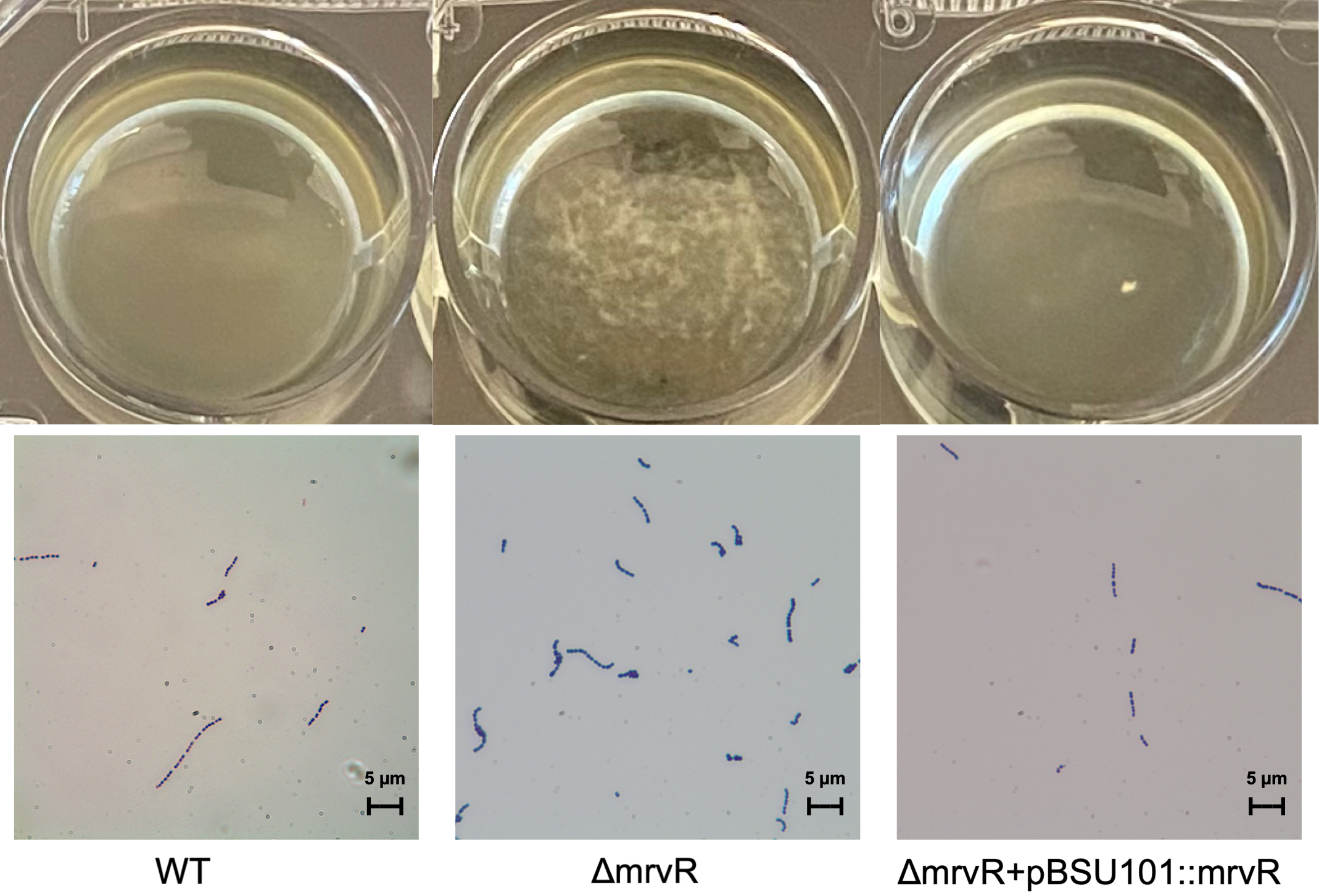

Supplement: S4 Fig — Strains were grown in TS broth (with spectinomycin selection for the complemented control strain) and photographed after overnight growth in a 24-well plate (top). Microscopic examination of Gram-stained preparations did not reveal obvious differences in cellular morphology (bottom). (TIF) [file ppat.1009116.s007.tif]

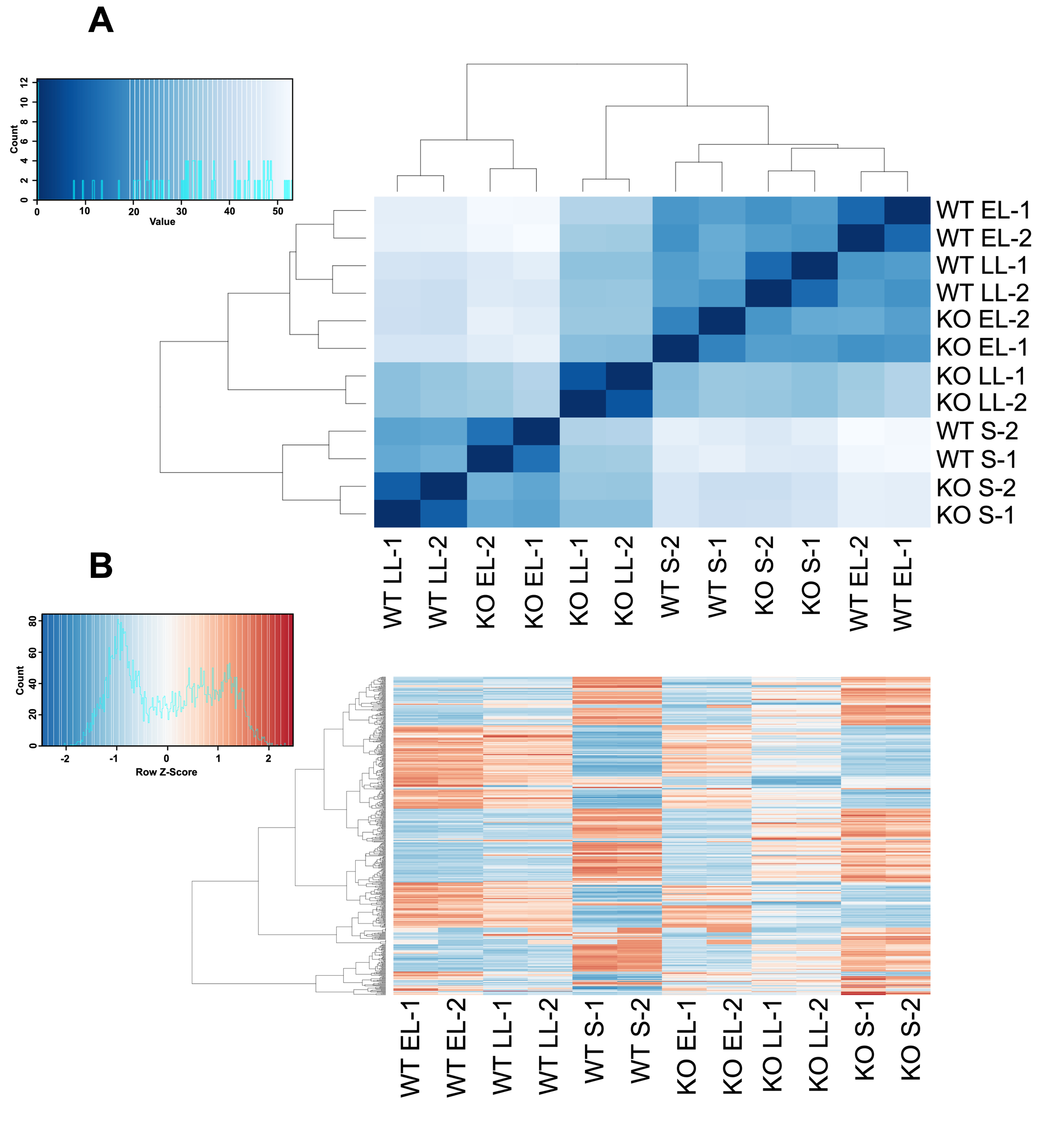

Supplement: S5 Fig — Genome wide correlation matrix with hierarchical clustering (A). Top 500 regulated genes across all RNA-seq samples with hierarchical clustering (B). (TIF) [file ppat.1009116.s008.tif]

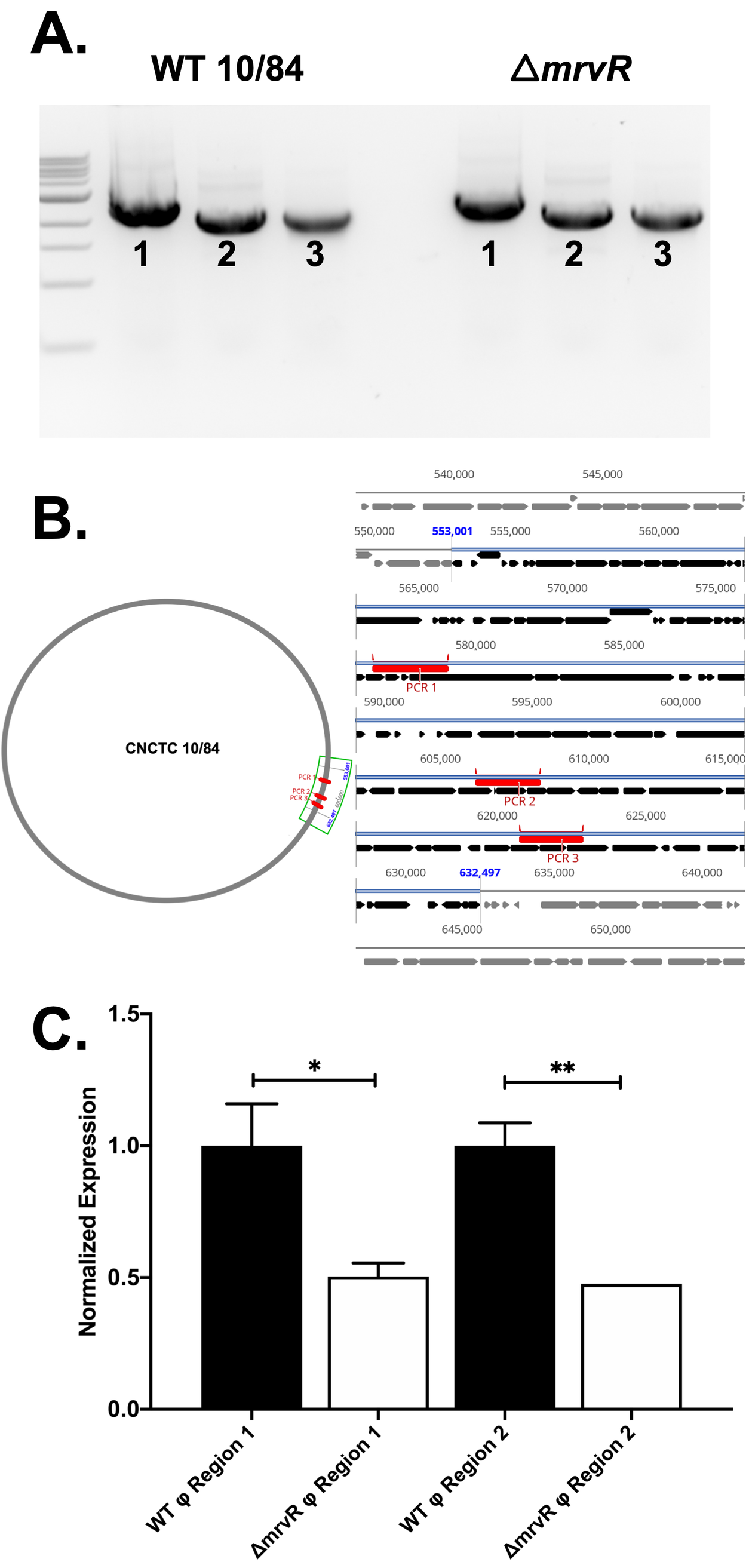

Supplement: S6 Fig — Three regions within the prophage island spanning gene loci W903_RS03075 through W903_RS03520 were amplified from wild type and mrvR knockout GBS genomic DNA. The PCR products from the two strains were the same size when assessed by gel electrophoresis (A). The schematic shows the three amplified regions of the chromosome in red (B). qPCR validation of RNA-seq data showing decreased expression of RNA from regions 1 and 2 in the mrvR knockout compared to wild type (C). (TIF) [file ppat.1009116.s009.tif]

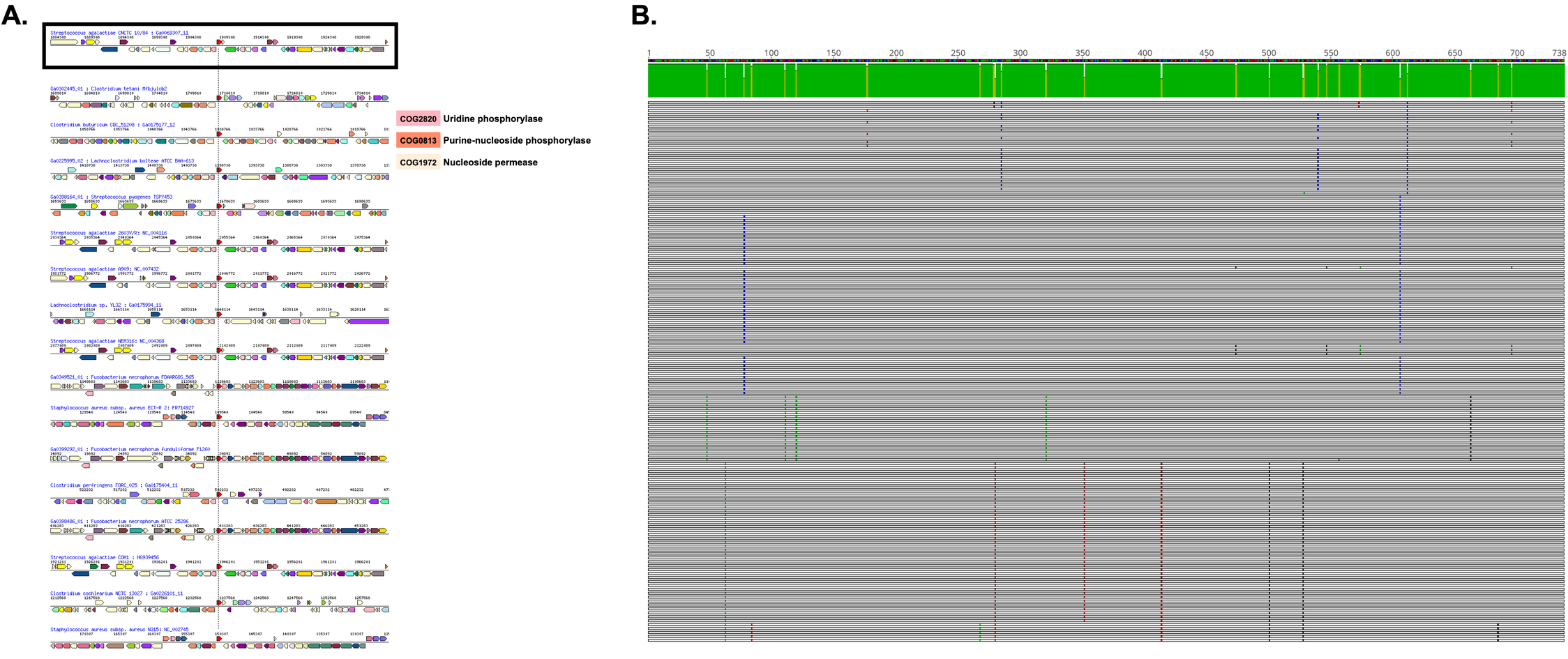

Supplement: S7 Fig — Gene synteny plot showing orthologs of the GBS mrvR gene in 16 other bacterial species. The plot was generated using the Integrated Microbial Genomes & Microbiomes server on the JGI genome portal (https://img.jgi.doe.gov). Genes are color coded by COG functional prediction. COG categories related to nucleotide metabolism present on the diagram are noted (A). Conservation of the mrvR coding sequence among 138 sequenced GBS genomes aligned through NCBI BLAST (B). The top of the panel shows percent conservation at each nucleotide sequence along the coding sequence. Mismatches in individual sequences (gray lines) are highlighted and color-coded for nucleotide identity. (TIF) [file ppat.1009116.s010.tif]
